# Effects of Pitavastatin on Coronary Artery Disease and Inflammatory Biomarkers in HIV: Mechanistic Substudy of the REPRIEVE Randomized Clinical Trial

**DOI:** 10.1001/jamacardio.2023.5661

**Published:** 2024-02-21

**Authors:** Michael T. Lu, Heather Ribaudo, Borek Foldyna, Markella V. Zanni, Thomas Mayrhofer, Julia Karady, Jana Taron, Kathleen V. Fitch, Sara McCallum, Tricia H. Burdo, Kayla Paradis, Sandeep S. Hedgire, Nandini M. Meyersohn, Christopher DeFilippi, Carlos D. Malvestutto, Audra Sturniolo, Marissa Diggs, Sue Siminski, Gerald S. Bloomfield, Beverly Alston-Smith, Patrice Desvigne-Nickens, Edgar T. Overton, Judith S. Currier, Judith A. Aberg, Carl J. Fichtenbaum, Udo Hoffmann, Pamela S. Douglas, Steven K. Grinspoon

**Affiliations:** 1 Cardiovascular Imaging Research Center, Department of Radiology, Massachusetts General Hospital, Harvard Medical School, Boston; 2 Center for Biostatistics in AIDS Research, Harvard T. H. Chan School of Public Health, Boston, Massachusetts; 3 Metabolism Unit, Massachusetts General Hospital, Harvard Medical School, Boston; 4 School of Business Studies, Stralsund University of Applied Sciences, Stralsund, Germany; 5 Cardiovascular Imaging Research Group, Heart and Vascular Center, Semmelweis University, Budapest, Hungary; 6 Department of Radiology, Medical Center University of Freiburg, Faculty of Medicine, University of Freiburg, Freiburg, Germany; 7 Department of Microbiology, Immunology, and Inflammation, Center for NeuroVirology and Gene Editing, Temple University Lewis Katz School of Medicine, Philadelphia, Pennsylvania; 8 Inova Heart and Vascular Institute, Falls Church, Virginia; 9 Division of Infectious Diseases, Ohio State University Medical Center, Columbus; 10 Frontier Science Foundation, Amherst, New York; 11 Department of Medicine, Duke Global Health Institute, Duke Clinical Research Institute, Duke University, Durham, North Carolina; 12 Division of AIDS, National Institute of Allergy and Infectious Diseases, National Institutes of Health, Bethesda, Maryland; 13 Division of Cardiovascular Sciences, National Heart, Lung, and Blood Institute, National Institutes of Health, Bethesda, Maryland; 14 Division of Infectious Diseases, University of Alabama at Birmingham; 15 ViiV Healthcare, Research Triangle Park, North Carolina; 16 Division of Infectious Diseases, David Geffen School of Medicine, University of California, Los Angeles; 17 Division of Infectious Diseases, Icahn School of Medicine at Mount Sinai, New York, New York; 18 Division of Infectious Diseases, University of Cincinnati College of Medicine, Cincinnati, Ohio; 19 Cleerly, Denver, Colorado; 20 Duke University Research Institute, Duke University School of Medicine, Durham, North Carolina

## Abstract

**Importance:**

Cardiovascular disease (CVD) is increased in people with HIV (PWH) and is characterized by premature noncalcified coronary plaque. In the Randomized Trial to Prevent Vascular Events in HIV (REPRIEVE), pitavastatin reduced major adverse cardiovascular events (MACE) by 35% over a median of 5.1 years.

**Objective:**

To investigate the effects of pitavastatin on noncalcified coronary artery plaque by coronary computed tomography angiography (CTA) and on inflammatory biomarkers as potential mechanisms for MACE prevention.

**Design, Setting, and Participants:**

This double-blind, placebo-controlled randomized clinical trial enrolled participants from April 2015 to February 2018 at 31 US clinical research sites.

PWH without known CVD who were taking antiretroviral therapy and had low to moderate 10-year CVD risk were included. Data were analyzed from April to November 2023.

**Intervention:**

Oral pitavastatin calcium, 4 mg per day.

**Main Outcomes and Measures:**

Coronary CTA and inflammatory biomarkers at baseline and 24 months. The primary outcomes were change in noncalcified coronary plaque volume and progression of noncalcified plaque.

**Results:**

Of 804 enrolled persons, 774 had at least 1 evaluable CTA. Plaque changes were assessed in 611 who completed both CT scans. Of 611 analyzed participants, 513 (84.0%) were male, the mean (SD) age was 51 (6) years, and the median (IQR) 10-year CVD risk was 4.5% (2.6-7.0). A total of 302 were included in the pitavastatin arm and 309 in the placebo arm. The mean noncalcified plaque volume decreased with pitavastatin compared with placebo (mean [SD] change, −1.7 [25.2] mm^3^ vs 2.6 [27.1] mm^3^; baseline adjusted difference, −4.3 mm^3^; 95% CI, −8.6 to −0.1; *P* = .04; 7% [95% CI, 1-12] greater reduction relative to placebo). A larger effect size was seen among the subgroup with plaque at baseline (−8.8 mm^3^ [95% CI, −17.9 to 0.4]). Progression of noncalcified plaque was 33% less likely with pitavastatin compared with placebo (relative risk, 0.67; 95% CI, 0.52-0.88; *P* = .003). Compared with placebo, the mean low-density lipoprotein cholesterol decreased with pitavastatin (mean change: pitavastatin, −28.5 mg/dL; 95% CI, −31.9 to −25.1; placebo, −0.8; 95% CI, −3.8 to 2.2). The pitavastatin arm had a reduction in both oxidized low-density lipoprotein (−29% [95% CI, −32 to −26] vs −13% [95% CI, −17 to −9]; *P* < .001) and lipoprotein-associated phospholipase A2 (−7% [95% CI, −11 to −4] vs 14% [95% CI, 10-18]; *P* < .001) compared with placebo at 24 months.

**Conclusions and Relevance:**

In PWH at low to moderate CVD risk, 24 months of pitavastatin reduced noncalcified plaque volume and progression as well as markers of lipid oxidation and arterial inflammation. These changes may contribute to the observed MACE reduction in REPRIEVE.

**Trial Registration:**

ClinicalTrials.gov Identifier: NCT02344290

## Key Points

**Question:**

Can pitavastatin reduce noncalcified coronary plaque and inflammatory biomarkers in people with HIV?

**Findings:**

In this substudy of the Randomized Trial to Prevent Vascular Events in HIV (REPRIEVE) including 611 people with HIV, compared with the placebo arm, the pitavastatin arm had a significant 7% relative reduction in noncalcified plaque. Pitavastatin use was associated with reductions in oxidized low-density lipoprotein cholesterol and lipoprotein-associated phospholipase A2.

**Meaning:**

In this study, among people with HIV at low to moderate cardiovascular risk, pitavastatin showed promise to reduce noncalcified coronary plaque as well as markers of lipid oxidation and arterial inflammation.

## Teaser

This substudy of a randomized clinical trial investigates the effects of pitavastatin on noncalcified coronary artery plaque using coronary computed tomography angiography and on inflammatory biomarkers as potential mechanisms for major adverse cardiovascular event prevention.

## Introduction

People with HIV (PWH) are at 1.5-fold to 2-fold risk of major adverse cardiovascular events (MACE), including myocardial infarction and stroke, and develop coronary artery disease (CAD) at an earlier age compared with people without HIV.^1,2^ Increased MACE in PWH is thought to be related, in part, to traditional mechanisms as well as to residual inflammation and immune activation.^3,4,5^ Premature CAD in PWH is characterized by increased noncalcified coronary plaque, which may contribute to increased cardiovascular events.^6^ As PWH are increasingly likely to die of non–HIV-related causes, including cardiovascular disease (CVD),^7^ it is important to develop a strategy to improve CAD and cardiovascular outcomes in PWH.

The Randomized Trial to Prevent Vascular Event in HIV (REPRIEVE) trial is a phase 3 multicenter randomized clinical trial of oral pitavastatin calcium, 4 mg per day, vs matched placebo for primary prevention of atherosclerotic CVD (ASCVD) events in PWH at low to moderate risk for ASCVD.^8^ In REPRIEVE, the pitavastatin arm had 35% less incident MACE compared with placebo over a median (IQR) of 5.1 (4.3-5.9) years, an effect beyond that anticipated from low-density lipoprotein (LDL) cholesterol reduction alone.^9^

The mechanistic substudy embedded within REPRIEVE was designed to determine potential statin effects to reduce noncalcified plaque volume and progression as assessed by entry and 2-year coronary computed tomography angiography (CTA). Moreover, the substudy sought to identify effects on lipid parameters and circulating biomarkers of inflammation.^10^

## Methods

### Trial Design and Oversight

The design of REPRIEVE and the mechanistic substudy have been previously described,^8,10^ and the trial protocol and statistical analysis plan are available in Supplement 1. In brief, 31 REPRIEVE sites, primarily from the AIDS Clinical Trial Group, were selected to participate in the mechanistic substudy based on capabilities for coronary CTA and blood biomarker acquisition. The trial was designed by study principal investigators in consultation with the National Heart, Lung, and Blood Institute, the National Institute of Allergy and Infectious Diseases, and the AIDS Clinical Trials Group. Institutional review board/ethics committee approval and any other applicable regulatory entity approvals were obtained from the Mass General Brigham Institutional Review Board and each clinical research site. Participants were provided with study information, including discussion of risks and benefits, and signed the approved declaration of informed consent. This study followed the Consolidated Standards of Reporting Trials (CONSORT) reporting guideline.

### Trial Population

For the main REPRIEVE trial, eligible individuals were aged 40 to 75 years, had documented HIV, were taking antiretroviral therapy for 180 days or more, had a CD4 cell count greater than 100 cells/mm^3^, estimated glomerular filtration rate of 60 mL/min/1.73 m^2^ or greater, had an alanine transaminase level 2.5-fold the upper limit of normal or lower, and low to moderate 10-year ASCVD risk as calculated by the 2013 Pooled Cohort Equation risk calculator, with LDL cholesterol less than threshold for a given risk score.^8^ Statin use in the 90 days before entry or prior history of CVD were exclusionary. Race and ethnicity were self-reported via a questionnaire; options included Asian, Black or African American, White, or other race, which included participants identifying as American Indian or Alaska Native, Native Hawaiian or Other Pacific Islander, or having more than 1 or unknown race. The REPRIEVE mechanistic substudy enrolled REPRIEVE participants without contraindications to coronary CTA. Detailed inclusion and exclusion criteria are available in the eMethods in Supplement 2.^10^

### Randomization

REPRIEVE participants were randomized 1:1 to pitavastatin calcium, 4 mg per day, and to matched placebo. Randomization was accomplished using a centralized computer system, stratified by sex, CD4 cell count, and participation in the mechanistic substudy. Study investigators and participants were blinded to randomization. Participants received information on heart-healthy lifestyle, diet, and smoking cessation.

### Coronary CTA and Plaque Assessment

Coronary CTA was performed on CT scanners using 64 slices or more according to Society of Cardiovascular CT guidelines.^11^ Deidentified coronary CTA images were transmitted to the central REPRIEVE CT core laboratory, as previously described.^10,12^ Coronary plaque volume and composition were quantified using semi-automated plaque analysis software (QAngio CT; Medis Medical Imaging).^13,14^ The coronary tree was automatically extracted. Plaque length was established visually with markers at the proximal and distal edges of plaque, and each participant’s CTs were read side-by-side blinded to scan order so that the same portion of the coronary artery was measured to minimize interscan variability. The inner and outer borders of the coronary wall were defined by the software and manually edited. Voxels between the outer wall of the coronary artery and the lumen were considered plaque; plaque composition was assigned based on voxel attenuation in Hounsfield units (HU; calcified plaque was defined as ≥350 HU, noncalcified plaque as <350 HU and low attenuation as <30 HU).^15,16^ Participants were randomly assigned to 1 of 3 experienced CT core laboratory readers (B. F., J. K., and J. T.), who read CTs blinded to clinical information, order of CT acquisition, and randomization. Interobserver reproducibility for the primary outcome, noncalcified coronary plaque volume, was excellent (intraclass correlation coefficient, 0.97; 95% CI, 0.95-0.99) in a subset of 40 baseline and follow-up CTs.

The primary outcomes were change in noncalcified coronary plaque volume and progression of noncalcified coronary plaque, defined as any increase in noncalcified plaque volume or new noncalcified plaque. Supportive outcomes included change in total plaque volume (including both noncalcified and calcified components) and change in the composition of plaque (ratio of noncalcified to total plaque volume). An additional exploratory plaque outcome was change in low-attenuation plaque volume.^17,18^

### Lipids and Biomarkers

Blood was obtained fasting at entry, month 4, and month 24 for assessment of lipids (LDL and non–high-density lipoprotein cholesterol), inflammatory (high-sensitivity C-reactive protein [hsCRP], lipoprotein-associated phospholipase A2 [LpPLA2], and oxidized low-density lipoprotein [oxLDL]), and immune activation biomarkers (monocyte chemoattractant protein 1, IL-6, IL-10, IL-1β, IL-18, soluble CD14, and soluble CD163). Lipids and hsCRP were measured from serum at Quest Diagnostics, Secaucus, New Jersey. Inflammatory and immune biomarkers were measured from plasma using enzyme-linked immunosorbent assay kits (R&D Systems) except for oxLDL (Mercodia) at Temple University, Philadelphia, Pennsylvania.

### Statistical Analysis

The statistical analysis plan is provided in Supplement 1. While the main trial (N = 7769) was powered for a reduction in cardiovascular events, target enrollment for the mechanistic substudy was 800 participants to provide 90% power to detect an approximate 6% relative difference between the pitavastatin and placebo arms in noncalcified plaque volume over 2 years and a 27% relative change in the risk of plaque progression, assuming 50% had noncalcified plaque at entry and 15% dropout.^19^ Given the expected mixed population of participants with and without noncalcified plaque at entry, power was estimated via a simulation study including a 5% type error with no adjustment for the dual primary outcomes.

**Figure 1.**
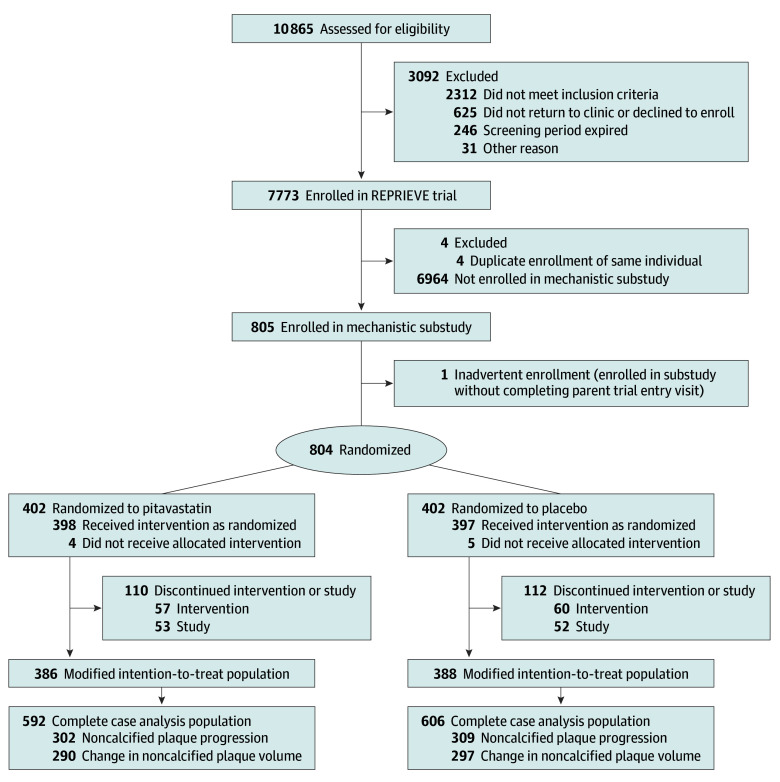
CONSORT Diagram REPRIEVE indicates Randomized Trial to Prevent Vascular Events in HIV.

Participants completing a parent trial entry visit and a separate substudy registration were considered enrolled (Figure 1). The modified intention-to-treat population for plaque outcomes was limited to participants who had at least 1 evaluable CT. Primary analyses included participants with complete case outcomes (eg, paired entry and year 2); sensitivity analyses used multiple imputation in the population with at least 1 evaluable CT (Statistical Analysis Plan in Supplement 1). A per-protocol analysis was conducted including only those participants who completed their 2-year course of pitavastatin or placebo. A planned secondary analysis was restricted to participants with quantified coronary plaque at entry (ie, excluding those with no plaque).

Data are presented as counts with percentages, means with SDs, and medians with IQRs. Changes from baseline in continuous outcomes are presented as means with 95% CIs. Treatment group differences in plaque volumes were based on differences in means adjusted for entry plaque volume using linear regression. Analyses adjusting for race and ethnicity, sex, and ASCVD risk score did not change the findings and are not presented. Analyses were conducted on the measured scale and with a log transformation to reflect both absolute and relative changes. Probability of noncalcified and total plaque volume progression was compared by treatment group using a χ^2^ test with the effect size presented as a relative risk (stratified on plaque presence at entry). Statin effects on lipids and markers of immune function and inflammation were assessed via Wilcoxon test for continuous variables.

Associations with changes in LDL cholesterol as well as immune and inflammatory biomarkers were assessed using linear regression for noncalcified coronary plaque volume and using log binomial regression for noncalcified plaque progression. These mechanistic analyses were limited to the per-protocol population with paired biomarkers and plaque outcomes (overall and the subpopulations with plaque at entry). The effects of biomarker changes on noncalcified coronary plaque volume at 4 and 24 months were examined to find the relationship of short-term and longer-term biomarkers changes to longer-term changes in noncalcified plaque volume. We also assessed whether adjusting for these biomarkers impacted the pitavastatin effect on plaque.

All inference used a 5% type I error with no adjustment for multiple comparisons, and all *P* values were 2-tailed. All analyses were conducted with SAS version 9.4M7 (SAS Institute).

## Results

### Study Population

A total of 804 participants from 31 US sites were enrolled from April 2015 to February 2018. Baseline characteristics of the enrolled population were previously reported and are compared with the parent REPRIEVE trial in eTable 1 in Supplement 2.^12^ Of the 804 enrolled participants, 774 had at least 1 evaluable CT (eTable 2 in Supplement 2). Of these, 611 had complete data for evaluation of noncalcified plaque progression, and 587 had complete data for change in noncalcified plaque volume. Among the 611 complete case participants (Table 1), 513 (84.0%) were male, the mean (SD) age was 51 (6) years, and the median (IQR) 10-year CVD risk was 4.5% (2.6-7.0). A total of 302 were included in the pitavastatin arm and 309 in the placebo arm. The median (IQR) HIV diagnosis duration at enrollment was 15 (9-22) years. The median (IQR) 10-year ASCVD risk score was 4.5% (2.6-7.0), with 338 (55.3%) having an ASCVD risk score less than 5%. The median (IQR) LDL cholesterol level was 108 (87-126) mg/dL (to convert to millimoles per liter, multiply by 0.0259). The median (IQR) body mass index (calculated as weight in kilograms divided by height in meters squared) was 26.9 (24.3-30.1). Participants had good virologic control, with a median (IQR) CD4 cell count of 609 (442-783) cells/mm^3^; 364 (59.6%) had been taking antiretroviral therapy for more than 10 years.

**Table 1.** Baseline Characteristics by Treatment Arm

Characteristic	No. (%)	
	Pitavastatin (n = 302)	Placebo (n = 309)
**Demographic characteristics**		
Age, y		
Mean (SD)	51 (6)	51 (6)
40-49	117 (39)	141 (46)
50-59	163 (54)	142 (46)
≥60	22 (7)	26 (8)
Natal sex		
Female	51 (17)	47 (15)
Male	251 (83)	262 (85)
Gender identity		
Cisgender	293 (97)	303 (98)
Transgender spectrum	7 (2)	5 (2)
Not reported	2 (1)	1 (<0.5)
Self-reported race^a^		
Asian	4 (1)	5 (2)
Black or African American	105 (35)	115 (37)
White	159 (53)	168 (54)
Other race	34 (11)	21 (7)
Self-reported ethnicity^b^		
Hispanic or Latino	79 (26)	71 (23)
Not Hispanic or Latino	221 (73)	234 (76)
Unknown	2 (1)	4 (1)
**Cardiovascular risk factors**		
ASCVD risk score, %		
Median (IQR)	4.4 (2.7-6.7)	4.6 (2.5-7.0)
0-<2.5	67 (22)	75 (24)
2.5-<5	100 (33)	96 (31)
5-10	116 (38)	117 (38)
>10	19 (6)	21 (7)
Coronary artery calcium score in Agatston units, No./total No. (%)		
0	194/290 (67)	191/293 (65)
1-100	67/290 (23)	74/293 (25)
101-400	25/290 (9)	20/293 (7)
>400	4/290 (1)	8/293 (3)
Smoking status, No./total No. (%)		
Current	74/301 (25)	71/308 (23)
Former	104/301 (35)	93/308 (30)
Never	123/301 (41)	144/308 (47)
Substance use, No./total No. (%)^c^		
Current	5/301 (2)	7/307 (2)
Former	146/301 (49)	148/307 (48)
Never	150/301 (50)	152/307 (50)
Use of antihypertensive medication	61 (20)	63 (20)
Systolic blood pressure, mean (SD), mm Hg	123 (13)	122 (13)
Cholesterol level, median (IQR), mg/dL^d^		
Total	186 (164-205)	180 (159-207)
HDL	49 (40-61)	48 (39-59)
LDL	108 (89-126)	106 (86-128)
Triglycerides, median (IQR), mg/dL^d^	114 (78-163)	111 (76-173)
Family history of premature CVD	77 (25)	58 (19)
BMI		
Mean (SD)	27.3 (4.3)	27.3 (4.3)
<25	97 (32)	107 (35)
25-29.9	132 (44)	118 (38)
≥30	73 (24)	84 (27)
Prior statin use^e^	21 (7)	25 (8)
Preexisting diabetes	1 (<0.5)	0
Use of antidiabetic medication	1 (<0.5)	0
Use of ACE inhibitors or ARBs	43 (14)	42 (14)
Use of antiplatelet therapy (including aspirin)^f^	24 (8)	26 (8)
Use of nonstatin lipid-lowering therapy	24 (8)	19 (6)
**HIV history**		
Time since HIV diagnosis, median (IQR), y	15 (9-21)	15 (9-22)
Nadir CD4 cell count, cells/mm^3^		
<50	63 (21)	70 (23)
50-199	88 (29)	90 (29)
200-349	86 (28)	78 (25)
≥350	55 (18)	63 (20)
Unknown	10 (3)	8 (3)
Total ART use, y		
<5	43 (14)	46 (15)
5-10	75 (25)	83 (27)
≥10	184 (61)	180 (58)
HIV-related health		
CD4 cell count, cells/mm^3^		
Mean (SD)	629 (269)	639 (286)
<350	44 (15)	46 (15)
350-499	56 (19)	59 (19)
≥500	202 (67)	204 (66)
HIV-1 RNA, No./total No. (%)		
<LLQ	263/300 (88)	264/303 (87)
LLQ -<400 copies/mL	29/300 (10)	32/303 (11)
≥400 copies/mL	8/300 (3)	7/303 (2)
Entry ART regimen class		
NRTI + INSTI	120 (40)	143 (46)
NRTI + non-NRTI	85 (28)	81 (26)
NRTI + PI	62 (21)	40 (13)
NRTI sparing	7 (2)	12 (4)
Other NRTI containing	28 (9)	33 (11)
Entry NRTI		
TDF	148 (49)	162 (52)
TAF	88 (29)	81 (26)
Abacavir	52 (17)	47 (15)
No NRTI	9 (3)	15 (5)
Other	5 (2)	4 (1)
Entry INSTI		
No INSTI	151 (50)	128 (41)
Dolutegravir	71 (24)	83 (27)
Elvitegravir	57 (19)	65 (21)
Raltegravir	23 (8)	33 (11)

Abbreviations: ACE, angiotensin-converting enzyme; ARB, angiotensin receptor blocker; ART, antiretroviral therapy; ASCVD, atherosclerotic cardiovascular disease; BMI, body mass index (calculated as weight in kilograms divided by height in meters squared); CVD, cardiovascular disease; HDL, high-density lipoprotein; INSTI, integrase strand inhibitor; LDL, low-density lipoprotein; LLQ, lower limit of quantification; NRTI, nucleoside reverse transcriptase inhibitor; PI, protease inhibitor; RNA, ribonucleic acid; TAF, tenofovir alafenamide; TDF, tenofovir disoproxil fumarate.

SI conversion factor: To convert cholesterol to mmol/L, multiply by 0.259; triglycerides to mmol/L, multiply by 0.0113.

^a^ The other race category included participants self-identifying as American Indian or Alaska Native, Native Hawaiian or Other Pacific Islander, or having more than 1 or unknown race.

^b^ Ethnicity is presented per National Institutes of Health definitions.

^c^ Substance use includes use of cocaine, methamphetamine, and intravenous drugs.

^d^ Screening lipids presented.

^e^ Prior statin use based on site report at entry.

^f^ Aspirin use in antiplatelet therapy is limited to long-term aspirin use, defined as more than 60 days.

### Primary Outcome: Noncalcified Plaque Volume

At baseline, the mean (SD) noncalcified plaque volume was 52.3 (193.5) mm^3^ in the pitavastatin arm and 57.7 (109.2) mm^3^ in the placebo arm (Table 2). Noncalcified plaque volume decreased in the pitavastatin arm compared with the placebo arm at month 24 (mean [SD] change, −1.7 [25.2] mm^3^ vs 2.6 [27.1] mm^3^; baseline adjusted difference, −4.3 mm^3^; 95% CI, −8.6 to −0.1; *P* = .04). Analyzed as a relative change from baseline, the treatment effect represented a 7% (95% CI, 1-12) greater decrease in noncalcified plaque volume compared with placebo (fold change, 0.93; 95% CI, 0.88-0.99; *P* = .02). Progression of noncalcified plaque was less likely in the pitavastatin arm (relative risk, 0.67; 95% CI, 0.52-0.88; *P* = .003). The sensitivity analysis using multiple imputation to account for missing data and the per-protocol analysis including only participants who completed treatment as randomized supported these findings (eTables 3 and 4 in Supplement 2).

**Table 2.** Coronary Artery Plaque Changes by Treatment Arm^a^

Outcome	Treatment arm		Treatment effect^b^	
	Pitavastatin (n = 302)	Placebo (n = 309)	Estimated difference adjusted for baseline (95% CI)	*P* value
**Primary outcomes**				
NCP volume, mean (SD), mm^3^^c^				
Baseline	52.3 (193.5)	57.7 (109.2)	NA	NA
Month 24	50.6 (192.8)	60.4 (113.7)	NA	NA
Change from baseline	−1.7 (25.2)	2.62 (27.1)	−4.3 (−8.6 to −0.1)	.04
Fold change from baseline (95% CI)	0.95 (0.91-1.00)	1.02 (0.99-1.05)	0.93 (0.88-0.99)	.02
Progression of NCP, No. (%)	53 (18)	85 (28)	0.67 (0.52-0.88)	.003
**Secondary outcomes**				
Total plaque volume, mean (SD), mm^3^				
Baseline	65.6 (211.7)	72.2 (135.9)	NA	NA
Month 24	67.9 (218.4)	79.1 (149.1)	NA	NA
Change from baseline	2.34 (28.7)	6.89 (32.3)	−4.3 (−9.2 to 0.62)	NA
Progression of total plaque, No. (%)	80 (28)	100 (34)	0.89 (0.74-1.08)	NA
Reduction in NCP percentage, mean (SD)	−6.6% (14.3)	−2.6% (9.21)	−4.1% (−7.0 to −1.3)	NA
**Exploratory outcomes**				
Low-attenuation plaque volume, mean (SD), mm^3^				
Baseline	3.32 (16.5)	4.25 (15.3)	NA	NA
Month 24	2.43 (9.91)	4.17 (12.8)	NA	NA
Change from baseline	−0.9 (9.97)	−0.1 (8.16)	−1.2 (−2.2 to −0.1)	NA

Abbreviations: NA, not applicable; NCP, noncalcified plaque.

^a^ NCP defined as plaque voxels with attenuation <350 Hounsfield units. Total plaque includes all plaque voxels (noncalcified + calcified). Low-attenuation plaque defined as <30 Hounsfield units.

^b^ For continuous outcomes, the treatment effect is given as the mean difference or relative fold change between treatment groups; for binary outcomes, it is a relative risk. All treatment effects and associated *P* values are adjusted for baseline values. Intention-to-treat analyses were conducted with the exception of reduction in NCP percentage, which is calculated in the subgroup with plaque present at baseline.

^c^ Change in NCP volume could be assessed in a subset of 587 participants (297 in the pitavastatin arm and 290 in the placebo arm).

In a preplanned subgroup analysis, a larger effect size was seen among the subgroup with plaque at baseline (−8.8 mm^3^; 95% CI, −17.9 to 0.4; eTable 5 in Supplement 2), representing a 12% greater decrease in noncalcified plaque in pitavastatin compared with placebo arms. There was similar reduction in progression of noncalcified plaque compared with placebo (relative risk, 0.71; 95% CI, 0.55-0.93). Example images of plaque progression and regression are provided in eFigure 1 in Supplement 2.

### Secondary and Exploratory Plaque Outcomes

There was a nonsignificantly smaller increase in total plaque volume (ie, noncalcified and calcified) with pitavastatin (mean [SD] change: pitavastatin, 2.3 [28.7] mm^3^; placebo, 6.9 [32.3] mm^3^; treatment effect, −4.3 mm^3^; 95% CI, −9.2 to 0.62). With respect to plaque composition, the percentage of noncalcified plaque decreased a mean (SD) of −6.6% (14.3) in the pitavastatin arm and −2.6% (9.2) in the placebo arm, for a treatment effect of −4.1% (95% CI, −7.0 to −1.3). Low-attenuation plaque volume also decreased on average in the pitavastatin arm compared with the placebo arm (mean [SD] change, −0.9 [10.0] mm^3^ vs −0.1 [8.2] mm^3^; baseline adjusted difference, −1.2 mm^3^; 95% CI, −2.2 to −0.1).

At baseline, the mean (SD) calcified plaque volume was 10.4 (30.6) mm^3^ in the pitavastatin arm and 13.1 (42.4) mm^3^ in the placebo arm. Calcified plaque volume increased in both the pitavastatin and placebo arms at month 24 (mean [SD] change, 4.1 [13.2] mm^3^ vs 4.2 [15.4] mm^3^).

### Effect on Lipids and Inflammatory Biomarkers and Relationship to Plaque

Entry and month 24 circulating lipids and measures of inflammation are provided in Table 3 and eTable 6 in Supplement 2. Available month 4 values are provided in eTable 7 in Supplement 2. As in the parent REPRIEVE trial, LDL cholesterol levels were similar at baseline and changed from a median (IQR) of 105 (88-124) mg/dL to 75 (59-93) mg/dL in the pitavastatin group and from 107 (90-129) mg/dL to 108 (87-128) mg/dL in the placebo group at year 2. Similar effects were seen for non–high-density lipoprotein cholesterol.

**Table 3.** Distributions of Inflammatory and Immune Biomarkers at Entry and Month 24

Measure^a^	Median (IQR)				
	Entry		Month 24		
	Pitavastatin (n = 402)	Placebo (n = 402)	Pitavastatin (n = 349)	Placebo (n = 350)	*P* value
**Inflammatory markers**					
LpPLA2					
Total, No.	398	391	335	333	NA
LpPLA2 level, ng/mL	126 (94.8-169)	131 (89.6-163)	119 (84.0-160)	143 (105-186)	<.001
Change from baseline	NA	NA	−9.77 (−33.6 to 16.8)	19.9 (−5.6 to 43.0)	<.001
Mean fold change (95% CI)	NA	NA	0.93 (0.89-0.96)	1.14 (1.10-1.18)	NA
oxLDL					
Total, No.	398	392	335	333	NA
oxLDL level, U/L	53.0 (42.2-69.8)	53.6 (41.7-69.9)	37.6 (30.0-47.3)	48.2 (38.9-58.4)	<.001
Change from baseline	NA	NA	−14.9 (−28.9 to −3.93)	−6.45 (−20.2 to 5.26)	<.001
Mean fold change (95% CI)	NA	NA	0.71 (0.68-0.74)	0.87 (0.83-0.91)	NA
hsCRP^b^					
Total, No.	397	391	343	335	NA
hsCRP level, mg/dL	0.18 (0.08-0.39)	0.18 (0.09-0.33)	0.17 (0.07-0.36)	0.19 (0.10-0.40)	.02
Change from baseline	NA	NA	−0.01 (−0.10 to 0.08)	0.01 (−0.09 to 0.11)	.09
Mean fold change (95% CI)	NA	NA	0.89 (0.79-1.01)	1.06 (0.94-1.20)	NA
**Immune markers**					
MCP-1					
Total, No.	398	390	335	330	NA
MCP-1 level, pg/mL	189 (148-245)	183 (144-238)	192 (157-235)	193 (154-247)	.77
Change from baseline	NA	NA	2.11 (−37.8 to 47.5)	3.91 (−33.3 to 53.2)	.46
Mean fold change (95% CI)	NA	NA	1.02 (0.98-1.06)	1.05 (1.01-1.09)	NA
Soluble CD14					
Total, No.	398	392	335	333	NA
Soluble CD14 level, ng/mL	1840 (1544-2176)	1810 (1496-2200)	1541 (1284-1809)	1534 (1324-1850)	.52
Change from baseline	NA	NA	−289 (−588 to −5.08)	−237 (−528 to 36.1)	.54
Mean fold change (95% CI)	NA	NA	0.83 (0.81-0.86)	0.86 (0.83-0.88)	NA
Soluble CD163					
Total, No.	398	391	335	333	NA
Soluble CD163 level, ng/mL	849 (651-1163)	845 (613-1074)	1013 (728-1419)	1003 (745-1384)	.65
Change from baseline	NA	NA	149 (−60.8 to 420)	143 (−46.0 to 453)	.88
Mean fold change (95% CI)	NA	NA	1.21 (1.16-1.27)	1.23 (1.18-1.29)	NA
IL-6					
Total, No.	398	391	335	333	NA
IL-6 level, pg/mL	1.64 (0.99-2.76)	1.46 (0.98-2.59)	1.64 (0.99-2.73)	1.53 (0.93-2.62)	.24
Change from baseline	NA	NA	0 (−0.95 to 0.77)	−0.12 (−0.81 to 0.59)	.46
Mean fold change (95% CI)	NA	NA	0.98 (0.89-1.08)	0.94 (0.86-1.03)	NA
IL-1β					
Total, No.	377	375	320	320	NA
IL-1β level, fg/mL	75.9 (43.9-151)	87.0 (44.9-168)	88.6 (50.2-164)	96.4 (52.6-191)	.09
Change from baseline	NA	NA	2.01 (−41.4 to 58.1)	11.4 (−28.9 to 69.2)	.06
Mean fold change (95% CI)	NA	NA	1.05 (0.93-1.17)	1.19 (1.07-1.33)	NA
IL-18					
Total, No.	377	375	320	320	NA
IL-18 level, pg/mL	245 (179-330)	232 (169-310)	236 (169-310)	230 (176-308)	.79
Change from baseline	NA	NA	−8.53 (−48.2 to 35.3)	−3.77 (−43.3 to 44.1)	.26
Mean fold change (95% CI)	NA	NA	0.96 (0.92-1.00)	1.01 (0.97-1.04)	NA
IL-10					
Total, No.	363	353	305	300	NA
IL-10 level, pg/mL	0.24 (0.17-0.37)	0.22 (0.16-0.35)	0.21 (0.15-0.34)	0.22 (0.16-0.33)	.53
Change from baseline	NA	NA	−0.02 (−0.12 to 0.06)	0 (−0.09 to 0.09)	.08
Mean fold change (95% CI)	NA	NA	0.96 (0.87-1.05)	0.99 (0.91-1.07)	NA
Caspase-1					
Total, No.	377	375	320	320	NA
Caspase-1 level, pg/mL	66.6 (47.0-93.8)	69.6 (50.1-107)	69.6 (48.6-102)	74.2 (52.6-113)	.08
Change from baseline	NA	NA	−3.41 (−22.2 to 26.2)	5.61 (−18.6 to 30.5)	.07
Mean fold change (95% CI)	NA	NA	1.02 (0.94-1.10)	1.07 (0.98-1.16)	NA

Abbreviations: hsCRP, high-sensitivity C-reactive protein; IL, interleukin; LpPLA2, lipoprotein-associated phospholipase A2; MCP-1, monocyte chemoattractant protein 1; NA, not applicable; oxLDL, oxidized low-density lipoprotein.

SI conversion factor: To convert hsCRP to mg/L, multiply by 10.

^a^ Data are reported for all enrolled participants with available biomarker results.

^b^ For hsCRP, censored values below the assay limit are randomly imputed based on a uniform distribution; otherwise, censored values below the assay limit are imputed as the result −0.01; values above the assay limit are imputed as result 0.1.

The pitavastatin arm demonstrated a greater reduction in oxLDL at year 2 (pitavastatin, −29%; 95% CI, −32 to −26; placebo, −13%; 95% CI, −17 to −9; *P* < .001). Pitavastatin also reduced LpPLA2 by −7% (95% CI, −11 to −4), while there was a 14% (95% CI, 10-18) increase in LpPLA2 in the placebo arm (*P* < .001) at year 2. There was a nonsignificant decrease in hsCRP (Table 3). No significant effects were seen on markers of immune activation.

**Figure 2.**
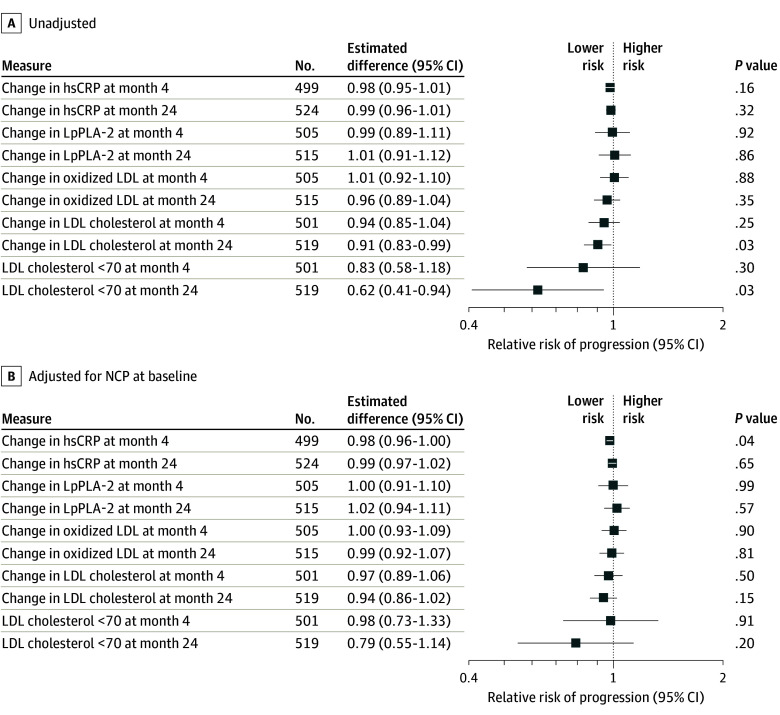
Effect of Biomarkers on Relative Risk of Progression of Noncalcified Plaque Effect size is per 25% decrease in the biomarker. The *P* value tests for a linearly increasing log relative risk. Mechanistic analyses were limited to the per-protocol population with paired biomarkers and plaque outcomes. hsCRP indicates high-sensitivity C-reactive protein; LDL, low-density lipoprotein; LpPLA-2, lipoprotein-associated phospholipase A2; NCP, noncalcified plaque.

There was no significant association between change in LDL cholesterol and change in continuous noncalcified plaque volume (eFigure 2 in Supplement 2). Change in LDL cholesterol and an achieved LDL cholesterol level less than 70 mg/dL at 24 months were associated with lower risk of noncalcified plaque progression, though these relationships were not significant after adjustment for baseline plaque volume (Figure 2). No significant associations between changes in oxLDL, hsCRP, or LpPLA2 and noncalcified plaque progression or change in noncalcified plaque volume were apparent. The association between pitavastatin and changes in noncalcified plaque did not change after adjustment for these biomarkers (eFigures 3 and 4 in Supplement 2).

## Discussion

REPRIEVE demonstrated that primary cardiovascular prevention with pitavastatin reduced the risk of MACE by 35% over a median (IQR) of 5.1 (4.3-5.9) years in PWH at low to moderate traditional cardiovascular risk,^9^ an effect beyond that anticipated from LDL cholesterol reduction alone. Within the embedded REPRIEVE mechanistic substudy, the pitavastatin arm demonstrated a relative reduction (treatment effect, −4.3 mm^3^; 95% CI, −8.6 to −0.1; a 7% [95% CI, 1-12] greater reduction relative to placebo) in noncalcified coronary plaque volume over 2 years, with an estimated 33% lower risk of plaque progression. The pitavastatin arm also had a reduction in LDL cholesterol and biomarkers of lipid oxidation (oxLDL) and arterial inflammation (LpPLA2), although there was not clear association with changes in noncalcified plaque volume. These findings provide insight into how pitavastatin may prevent MACE.

In designing the mechanistic substudy, we sought to assess the effects of a statin intervention on coronary plaque and inflammation in PWH. Coronary CTA allows for noninvasive quantification of changes in plaque in response to therapy.^20,21,22^ Here, we quantified noncalcified and low-attenuation coronary plaque, which are thought to be the biologically active component of plaque and are associated with MACE.^18,23^ Among PWH, increased noncalcified coronary plaque may be a mechanism of increased CVD.^12,24,25,26,27,28^ Noncalcified plaque was seen in a higher-than-expected percentage of participants at baseline in REPRIEVE, a relatively young primary prevention population with low to moderate traditional CVD risk.^12^ The observed reduction in plaque volume may therefore represent an important statin benefit potentially contributing to the reduced MACE observed with statin in REPRIEVE. Studies have shown that relatively small effects on atheroma volume of 1% can lead to large reductions in MACE.^29^ Other studies suggest decreases in noncalcified plaque with statin therapy may be due in part to conversion to calcified plaque, which may contribute to plaque stabilization.^30,31^ In this study, there were similar increases in calcified plaque volume between arms, while noncalcified plaque volume decreased more in the pitavastatin arm than the placebo arm. Indeed, there was a decrease in the proportion of noncalcified plaque by −4.1% (95% CI, −7.0 to −1.3) in the pitavastatin arm compared with the placebo arm, suggesting relatively greater effects of pitavastatin to reduce noncalcified plaque than to increase calcified plaque in this population with low to moderate ASCVD risk.

The observation of a reduction in noncalcified coronary plaque in the pitavastatin arm compared with the placebo arm in REPRIEVE builds on an earlier trial of atorvastatin in PWH,^19^ which found that atorvastatin reduced noncalcified plaque volume in 40 PWH with preexisting plaque. The amount of baseline noncalcified plaque and change in noncalcified plaque were relatively small but occurred in a population very different from that investigated in other trials of high-risk cohorts^20,21^ with more risk factors^32,33^ or with established CAD.^17,34^ Consistent with this, the effects of our statin intervention on plaque were larger among those with plaque at baseline in REPRIEVE. The reduction in noncalcified plaque volume among those with preexisting plaque in REPRIEVE was similar to that seen in a metanalysis of 3 CTA statin studies.^35^

While the focus was on the salutary effects of pitavastatin, the placebo arm also provides a valuable look at the natural history of CAD in PWH. In this group with low to moderate traditional CVD risk who would not normally qualify for statin, there was on average progression of noncalcified coronary plaque over 2 years. These findings support previous observations of accelerated CAD in PWH,^19,24,28,36^ which, along with the 35% reduction in MACE seen in the parent trial,^9^ build the case for primary prevention in this population.

The pitavastatin arm had a reduction in oxLDL and LpPLA2 compared with the placebo arm, in addition to LDL cholesterol. These changes are notable, as specific inflammatory pathways have been hypothesized to increase the risk of CVD in PWH.^4^ Prior studies have shown increased arterial inflammation using fludeoxyglucose-18 positron emission tomography in PWH compared with controls without HIV in the general population,^37^ linking arterial inflammation to noncalcified plaque among PWH.^38^ In this regard, LpPLA2 is a platelet-activating factor that hydrolyzes oxidized lipids, thought to play a proinflammatory role in atherogenesis and is a marker of arterial inflammation in PWH.^39,40^ In contrast, markers of generalized inflammation, such as CRP, may not be increased in well-treated PWH taking antiretroviral therapy, potentially explaining the more modest changes in CRP observed in our study. oxLDL is a marker of oxidative stress, which increases the atherogenic properties of LDL cholesterol and induces foam cell formation through localized macrophage activation and endothelial activation, key processes in plaque formation.^41^ In contrast, no significant effects on immune pathways were found.

Among studies in the general population, there is an overall association between achieved LDL cholesterol and reductions in coronary plaque volume, though considerable heterogeneity exists at the level of the individual study.^22^ Similarly, in REPRIEVE, there was a suggestion that achieved LDL cholesterol less than 70 mg/dL at 24 months was associated with a lower risk of noncalcified plaque progression in unadjusted analyses. Our population was unusual, as it was composed of PWH at low to moderate CVD risk with lower entry LDL cholesterol levels in contrast to other higher-risk populations with higher LDL cholesterol levels, in which statin effects on LDL may relate more strongly to changes in plaque. Decreases in systemic markers of lipid oxidation and arterial inflammation were not significantly associated with the changes in noncalcified plaque. However, effects of statins may modify inflammatory pathways through lipid effects occurring at the local plaque level, leading to reductions in inflammation in the plaque microenvironment as a mechanism of their anti-inflammatory effect.^42^ Moreover, changes in inflammatory pathways may be related to MACE, even if not related to changes in plaque volume, a hypothesis that will be further tested in the main REPRIEVE population. Further research is needed into the mechanism by which statins reduce noncalcified plaque among PWH, including potential effects related to atheroma formation, lipid uptake, and foam cell formation. Other effects of statins at the plaque surface to stabilize plaque, reduce inflammation within the plaque, and reduce plaque rupture, may be critical to preventing MACE in PWH. The data from REPRIEVE add to our growing understanding of the potential mechanisms of statin effects on CVD.^43^

### Limitations

Limitations of this study should be considered. Although our results are specific to pitavastatin, other statins may have similar effects. REPRIEVE was an international trial with 145 sites from 12 countries; the mechanistic substudy necessarily included a more limited population from 31 US sites and thus may not be representative of other regions. The substudy population was generally similar to the overall trial population in terms of ASCVD risk but included a higher proportion of male participants (eTable 1 in Supplement 2). A multiple imputation analysis addressed missing data and supported the overall conclusion of the study. The results may not be generalizable to higher-risk populations, with higher baseline LDL cholesterol and more plaque.

## Conclusions

In conclusion, in a primary prevention trial of PWH at low to moderate ASCVD risk, 2 years of pitavastatin reduced noncalcified coronary plaque volume and inflammatory biomarkers. These effects may contribute to the observed effects of pitavastatin on MACE in the parent REPRIEVE trial.

## Notes

Supplement 1. Trial Protocol and Statistical Analysis Plan

Supplement 2. **eAppendix.** List of REPRIEVE Mechanistic Substudy Site Investigators
eMethods.
**eTable 1.** Characteristics of Enrolled REPRIEVE Mechanistic Substudy and Overall REPRIEVE Trial Participants at Baseline* **eTable 2.** Baseline Characteristics of Participants Enrolled and With at Least 1 Evaluable Scan by Treatment Arm **eTable 3.** Sensitivity Analysis: Multiple Imputation of Changes in Coronary Artery Plaque by Treatment Arm **eTable 4.** Sensitivity Analysis: Per-Protocol Changes in Coronary Artery Plaque by Treatment Arm **eTable 5.** Coronary Artery Plaque Changes by Treatment Arm, Secondary Analysis in Subgroup With Plaque at Entry and Year 2 CT Available for Paired Analysis **eTable 6.** Distributions of Fasting Lipids (Entry, Month 4, and Month 24) **eTable 7.** Distributions of Inflammatory and Immune Biomarkers (Month 4) **eFigure 1.** Example Baseline and 2-Year Follow-Up Coronary CTA Demonstrating Plaque Progression on Placebo (Top Row) and Plaque Regression on Pitavastatin (Bottom Row) **eFigure 2.** Effect of Individual Biomarkers on Change in Noncalcified Plaque Volume **eFigure 3.** Effect of Treatment on Noncalcified Plaque Volume, Adjusted for Change in Individual Biomarkers **eFigure 4.** Effect of Treatment on Noncalcified Plaque Progression, Adjusted for Change in Individual Biomarkers
eReferences.

Supplement 3. **Group Information.** REPRIEVE Trial Writing Group

Supplement 4. Data Sharing Statement
