# Supplementary material for: Effects of Pitavastatin on Coronary Artery Disease and Inflammatory Biomarkers in HIV: Mechanistic Substudy of the REPRIEVE Randomized Clinical Trial
Source: JAMA Cardiol. 2024 Feb 21;9(4):323–34. doi: 10.1001/jamacardio.2023.5661 (PMC10882511; doi:10.1001/jamacardio.2023.5661)
Supplement: Supplement 1. — Trial Protocol and Statistical Analysis Plan [file jamacardiol-e235661-s001.pdf]

Protocol

Protocol for: Lu MT, Ribaudo H, Foldyna B, et al. Effects of Pitavastatin on Coronary Artery Disease and Inflammatory Biomarkers in HIV: Mechanistic Substudy of the Randomized Trial to Prevent Vascular Events in HIV (REPRIEVE)

This trial protocol has been provided by the authors to give readers additional information about the work.

The following are included in this document (1 – 6 below are hyperlinked)

1. Original REPRIEVE (A5332) protocol (Version 2.0, 19 Dec 2014)*
2. Original REPRIEVE Mechanistic Substudy (A5333s) protocol (Version 2.0, 19 Dec 2014)*
3. Final REPRIEVE (A5332) protocol (Version 6.0, 16 May 2022)
4. Final REPRIEVE Mechanistic Substudy (A5333s) protocol (Version 6.0, 16 May 2022)
5. Protocol summary of changes Versions 2.0 – 6.0
6. REPRIEVE Mechanistic Substudy Statistical Analysis Plan (Version 1.1, 4 Oct 2023)

*Protocol Version 2.0 was the initial protocol version distributed to sites.

Randomized Trial to Prevent Vascular Events in HIV – REPRIEVE (A5332)

**Principal Investigators:**	**Steven Grinspoon, MD**<br>**Pamela Douglas, MD**<br>**Udo Hoffmann, MD, MPH**<br>**Heather Ribaudo, PhD**
**AIDS Clinical Trials Group Investigators:**	**Turner Overton, MD**<br>**Carl Fichtenbaum, MD**<br>**Judith Aberg, MD**<br>**Markella Zanni, MD**
**Data Coordinating Center:**	**Harvard School of Public Health**

**Study Funders:**	**National Heart, Lung, and Blood Institute**<br>**National Institute of Allergy and Infectious Diseases**
**Industry Support:**	**Kowa Pharmaceuticals America**
**NHLBI Program and Medical Officer:**	**Monica R. Shah, MD**
**DAIDS Medical Officer:**	**Karin L. Klingman, MD**
**IND Sponsor:**	**Division of AIDS, NIAID, NIH**
**IND Number:**	**119127**

Final Version 2.0
December 19, 2014

CONTENTS

Page

SITES PARTICIPATING IN THE STUDY 4
PROTOCOL TEAM ROSTER 4
STUDY MANAGEMENT 7
GLOSSARY OF PROTOCOL-SPECIFIC TERMS 8
EXECUTIVE SUMMARY 9

1.0 HYPOTHESIS AND STUDY OBJECTIVES 10
1.1 Clinical Hypotheses 10
1.2 Clinical Objectives 10

2.0 INTRODUCTION 11
2.1 Background 11
2.2 Rationale 18

3.0 STUDY DESIGN 19

4.0 SELECTION AND ENROLLMENT OF SUBJECTS 19
4.1 Inclusion Criteria 19
4.2 Exclusion Criteria 21
4.3 Study Enrollment Procedures 23
4.4 Mechanistic Substudy of REPRIEVE (A5333s) Enrollment Procedures 24
4.5 Coenrollment Guidelines 24
4.6 Retention Procedures 24

5.0 STUDY TREATMENT 24
5.1 Regimens, Administration, and Duration 24
5.2 Study Product Formulation and Preparation 25
5.3 Pharmacy: Product Supply, Distribution, and Accountability 25
5.4 Concomitant Medications 26

6.0 CLINICAL AND LABORATORY EVALUATIONS 27
6.1 Schedule of Events 27
6.2 Timing of Evaluations 29
6.3 Instructions for Evaluations and Data Collection 30
6.4 Endpoint Assessments 36

7.0 CLINICAL MANAGEMENT ISSUES 37
7.1 Toxicity Management 38
7.2 Requirement for Precautionary or Prohibited Medications (see PSWP) 40
7.3 Pregnancy 40
7.4 Unblinding Procedures 41

8.0 CRITERIA FOR DISCONTINUATION 41
8.1 Premature and Permanent Treatment Discontinuation 41
8.2 Premature Study Discontinuation 42

9.0 STATISTICAL CONSIDERATIONS 42
9.1 General Design Issues 42
9.2 Outcome Measures 42
9.3 Randomization and Stratification 44
9.4 Sample Size and Accrual 44

9.5 Monitoring ... 47
9.6 Analyses ... 49

10.0 PHARMACOLOGY PLAN ... 52

11.0 DATA COLLECTION AND MONITORING AND ADVERSE EVENT REPORTING ... 52
11.1 Records to Be Kept ... 52
11.2 Role of Data Management ... 52
11.3 Clinical Site Monitoring and Record Availability ... 53
11.4 Expedited Adverse Event Reporting to DAIDS ... 53

12.0 HUMAN SUBJECTS ... 54
12.1 Institutional Review Board (IRB) Review and Informed Consent ... 54
12.2 Subject Confidentiality ... 54
12.3 Study Discontinuation ... 54
12.4 Women and Minorities ... 54

13.0 PUBLICATION OF RESEARCH FINDINGS ... 55

14.0 BIOHAZARD CONTAINMENT ... 55

15.0 STUDY GOVERNANCE ... 55

16.0 REFERENCES ... 56

APPENDIX I: SAMPLE INFORMED CONSENT FOR REPRIEVE (A5332)

APPENDIX II: THE MECHANISTIC SUBSTUDY OF REPRIEVE (A5333s)

APPENDIX III: SAMPLE INFORMED CONSENT FOR THE MECHANISTIC SUBSTUDY OF REPRIEVE (A5333s)

SITES PARTICIPATING IN THE STUDY

REPRIEVE (A5332) is a multicenter study open to US clinical research sites and selected ACTG international sites that have been approved for participation by the protocol team. Refer to the protocol-specific website (PSWP) for the list of eligible sites.

PROTOCOL TEAM ROSTER

Chair
Steve Grinspoon, MD
MGH Program in Nutritional Metabolism
Harvard Medical School
55 Fruit Street
Longfellow 5, Room 207
Boston, MA 02114
Phone: 617-724-9109
E-mail: sgrinspoon@partners.org

Co-Chairs
Pamela Douglas, MD
Duke University Medical Center
7022 North Pavilion
Durham, NC 27715
Phone: 919-681-2690
E-mail: pamela.douglas@duke.edu

Udo Hoffmann, MD, MPH
MGH Cardiac MR PET CT Program
Harvard Medical School
165 Cambridge Street, Suite 400
Boston, MA 02114
Phone: 617-643-0329
E-mail: uhoffmann@partners.org

Edgar Turner Overton, MD
Division of Infectious Diseases
University of Alabama
CCB 325
1530 3rd Ave S
Birmingham, AL 35294
Phone: 205-996-2373
E-mail: toverton@uab.edu

Vice Chairs
Judith Aberg, MD
Icahn School of Medicine at Mount Sinai
One Gustave L. Levy Place
Box 10190
New York, NY 10029
Phone: 212- 241-6741
E-Mail: Judith.aberg@mountsinai.org

Vice Chairs (Cont.)
Carl Fichtenbaum, MD
University of Cincinnati
200 Eden Avenue, Room 3114
Mail Location 405
Cincinnati, OH 45267
Phone: 513-584-6361
E-Mail: carl.fichtenbaum@uc.edu

Markella Zanni, MD
MGH Program in Nutritional Metabolism
Harvard Medical School
55 Fruit Street
Longfellow 5, Room 211
Boston, MA 02114
Phone: 617-724-6926
E-mail: mzanni@partners.org

Mechanistic Substudy
Michael Lu, MD
MGH Cardiac MR PET CT Program
Harvard Medical School
165 Cambridge Street, Suite 400
Boston, MA 02114
Phone: 617-643-0239
E-mail: mlu@mgh.harvard.edu

DAIDS Medical Officer
Karin L. Klingman, MD
HIV Research Branch
TRP, DAIDS, NIAID, NIH
5601 Fishers Lane, Room 9E40A
Rockville, MD 20852
HIV Research Branch
Phone: 240-627-3067
E-Mail: kklingman@niaid.nih.gov

NHLBI Program and Medical Officer
Monica R. Shah, MD, MSJ, MHS
National Heart, Lung, and Blood Institute
6701 Rockledge Drive, Room 8173
Bethesda, MD 20892
Phone: 301-594-1079
E-Mail: shahmr@nhlbi.nih.gov

Clinical Trials Specialist
Barbara Bastow, BSN
ACTG Network Coordinating Center
Social & Scientific Systems, Inc.
8757 Georgia Avenue, 12th Floor
Silver Spring, MD 20910-3714
Phone: 301-628-3315
E-Mail: bbastow@s-3.com

Statisticians
Heather Ribaudo, PhD
Center for Biostatistics in AIDS Research
Harvard School of Public Health
655 Huntington Avenue
Boston, MA 02115
Phone: 617-432-2897
E-mail: ribaudo@sdac.harvard.edu

Pawel Paczuski
Statistical & Data Analysis Center
Harvard School of Public Health
FXB Building, Room 535
Boston, MA 02115
Phone: 617-432-1161
E-Mail: ppaczusk@sdac.harvard.edu

Data Manager
Anthony Holguin, BA
Frontier Science and Technology Research Foundation
4033 Maple Road
Amherst, NY 14226
Phone: 716-834-0900 x7429
E-Mail: Holguin@fstrf.org

DAIDS Pharmacists
Oladapo Alli
5601 Fisher's Lane, Room 9E16 MSC #9832
Bethesda, MD 20852
Phone: 240-627-3593
E-mail: oladapo.alli@nih.gov

Ana Martinez, RPh
5601 Fisher's Lane, Room 9D32
Rockville, MD 20852
Phone: 301-496-8213
E-mail: amartinez@niaid.nih.gov

Investigator
Carlos Malvestutto, MD, MPH
Nationwide Children's Hospital
Section of Infectious Disease
700 Children's Drive
Columbus, OH 43205
Phone: 614-722-4457
E-mail: Malvestutto.1@osu.edu

Field Representatives
David Currin, RN, ACRN, CCRC
UNC AIDS CRS
BI Building, 2nd Floor, Room 2109D
Campus Box 7215
130 Mason Farm Road
Chapel Hill, NC 27599
Phone: 919-966-2624
E-Mail: dcurrin@med.unc.edu

Jayinthie (Jane) Govender, RN
Durban Adult HIV CRS
719 Umbilo Road
PO Box 4013
Durban, Kwa-Zulu Natal 4092
South Africa
Phone: 011-27-31-2604880
E-Mail: jgovender@ecarefoundation.com

Erin Elizabeth Hoffman, BS
UNC AIDS CRS
130 Mason Farm Road
Chapel Hill, NC 27599
Phone: 919-843-0720
E-Mail: erin_stephenson@med.unc.edu

Laboratory Technologists
Joan Dragavon, MLM
Virology Specialty Laboratory
University of Washington
Research & Training Building
300 9th Avenue, Room 725
Seattle, WA 98104
Phone: 206-897-5210
E-Mail: dragavon@u.washington.edu

Francoise Giguel
Division of Infectious Diseases
Partners AIDS Research Center
Massachusetts General Hospital
65 Landsdowne Street, Room 435
Cambridge. MA 02139
Phone: 617-768-8374
E-Mail: fgiguel@mgh.harvard.edu

Community Scientific Subcommittee (CSS) Representative
Ruben Vidales
4363 Palmero Drive
Los Angeles, CA 90065
Phone: 323-517-7363
E-mail: vidaruben@gmail.com

Site Specialist
Akbar Shahkolahi, PhD
ACTG Network Coordinating Center
Social & Scientific Systems, Inc.
8757 Georgia Avenue, 12th Floor
Silver Spring, MD 20910-3714
Phone: 301-628-3318
E-Mail: ashahkolahi@s-3.com

Laboratory Data Manager
Heather Sprenger, MS
Frontier Science and Technology Research Foundation
4033 Maple Road
Amherst, NY 14226
Phone: 716-834-0900 x7438
E-Mail: sprenger@fstrf.org

STUDY MANAGEMENT

This section provides important instructions on how to attempt to have questions about REPRIEVE (A5332) answered. Following these instructions will help you receive an answer as quickly as possible, generally within 24 hours. Sites are responsible for documenting phone calls made to team members.

Protocol E-mail Group

The protocol logon is the primary vehicle for distributing important information about the study to sites. It is the site's responsibility to add all relevant personnel to this e-mail group as soon as possible. Contact actg.user.support@fstrf.org to be added to the "actg.protA5332" e-mail group and, for sites participating in the mechanistic substudy, to the "actg.prota5333s" substudy e-mail group.

IND (Investigational New Drug) Number or Questions

E-mail Regulatory@tech-res.com.

To Request Study Product Package Inserts and/or Investigator Brochures

E-mail RIC@tech-res.com.

Study Drug Orders

Call the Clinical Research Products Management Center (CRPMC) at 1+ 301-294-0741.

Questions about Study Product, Dose, Supplies, Records, and Returns

Call protocol pharmacists Ana Martinez at 301-496-8213 or Oladapo Alli at 240-627-3593.

Protocol Registration Questions

E-mail Protocol@tech-res.com or call 1+ 301-897-1707.

Subject Registration and Randomization Issues and Study Identification Number (SID) Lists

E-mail rando.support@fstrf.org or call 1+ 716-834-0900 x7301.

Computer and Data Entry Screen Problems

E-mail actg.support@fstrf.org or call 1+ 716-834-0900 x7302 (US sites) or 1+ 716-834-0900 x7200 (non-US sites).

Data Management Questions

For nonclinical questions about electronic case report forms (eCRF), the eCRF schedule of events, and other data management issues, e-mail Anthony Holguin at Holguin@fstrf.org.

Expedited Adverse Event (EAE) Reporting/Questions

Contact DAIDS through the RSC Safety Office at DAIDSRSCSafetyOffice@tech-res.com or call 1-800-537-9979 or 301-897-1709; or fax 1-800-275-7619 or 301-897-1710.

Other Questions

- For all other questions about REPRIEVE (A5332), e-mail actg.corea5332@fstrf.org.
- For all questions about coronary computed tomography angiography (CCTA), e-mail actg.corea5333s@fstrf.org.

Protocol-Specific Web Page (PSWP)

Additional information about protocol management can be found on the PSWP at https://member.actgnetwork.org/study/53278#profile=0.

GLOSSARY OF PROTOCOL-SPECIFIC TERMS

ACC	American College of Cardiology
ACTG	AIDS Clinical Trials Group
AHA	American Heart Association
AMI	acute myocardial infarction
ART	antiretroviral therapy
ASCVD	atherosclerotic cardiovascular disease
CAD	coronary artery disease
CCTA	coronary computed tomography angiography
CEC	Clinical Event Committee
CVD	cardiovascular disease
DAIDS	Division of AIDS
DM	diabetes mellitus
ECG	electrocardiogram
FIB-4	Fibrosis 4 Score
HDL	high-density lipoprotein
IVUS	intravascular ultrasound
LDL	low-density lipoprotein
MACE	major adverse cardiovascular events
NAC	non-AIDS complications
NHLBI	National Heart Lung and Blood Institute
NIAID	National Institute of Allergy and Infectious Diseases
NCEP	National Cholesterol Education Panel
PAD	peripheral arterial disease
FDG-PET	fluorodeoxyglucose (FDG)-positron emission tomography (PET)
PSWP	protocol-specific Web page
TIA	transient ischemic attack

EXECUTIVE SUMMARY

Title	Randomized Trial to Prevent Vascular Events in HIV – REPRIEVE (A5332)
Indication	To study the efficacy of statins to reduce the risk of cardiovascular disease in HIV-infected patients.
Location	Multicenter trial conducted primarily at US trial sites.
Brief Rationale	HIV-infected persons face an increased risk of CVD morbidity and mortality, yet no preventive strategies for CVD risk reduction have been proven for this population. Among HIV-infected individuals, immune activation may contribute in unique ways to atherosclerosis and ensuing cardiovascular events. Statins affect both traditional CVD risk factors (LDL cholesterol) and have pleiotropic effects to reduce inflammation and immune activation. Thus, statins may target the unique mechanisms of cardiovascular disease in HIV.
Study Design and Duration	Prospective, double-blind, randomized, placebo-controlled, multicenter efficacy study in 6500 subjects, with individual subjects to be followed for up to 72 months.
Treatment	Pitavastatin 4 mg PO daily or placebo for pitavastatin.
Primary Objective	To determine the effects of pitavastatin as a primary prevention strategy for major adverse cardiovascular events (MACE) in HIV.
Key Secondary Objectives	1. The effects of pitavastatin on the components of MACE and all-cause mortality.<br>2. The effects of pitavastatin on LDL and non-HDL in relationship to MACE.<br>3. Whether baseline traditional risk factors and time updated HIV-specific immunological risk factors are predictive of MACE and pitavastatin effects on MACE.<br>4. The effects of pitavastatin on the incidence of serious non-CVD events.<br>5. The safety of pitavastatin in the HIV population.
Primary Endpoint	Major adverse cardiovascular events (MACE)
Secondary and Safety Endpoints	Primary components of MACE, all-cause mortality, LDL cholesterol, immune function, non-CVD events (malignancy, end stage liver and kidney disease, AIDS-defining events), and safety endpoints, including diabetes mellitus
Abbreviated Study Flow	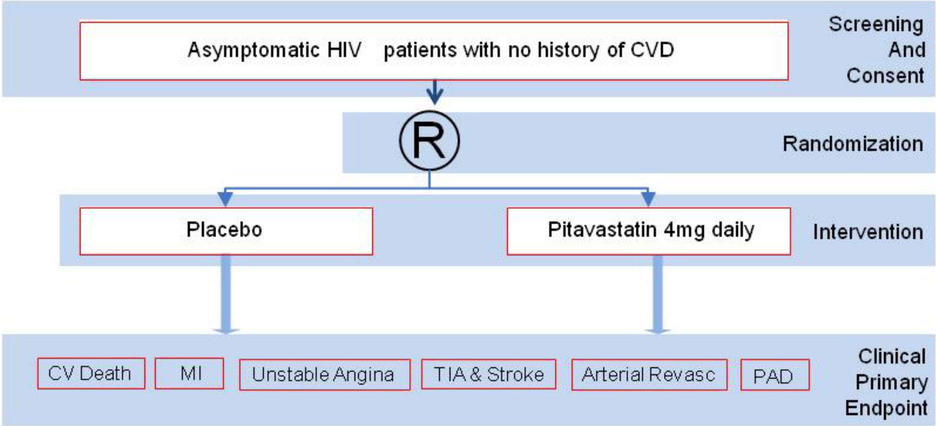

1.0 HYPOTHESIS AND STUDY OBJECTIVES

1.1 Clinical Hypotheses

1.1.1 Primary Clinical Hypothesis

Statin therapy will prevent atherosclerotic cardiovascular disease (ASCVD)-related MACE (major adverse cardiovascular events) in HIV-infected persons on antiretroviral therapy (ART) in whom traditional CVD risk is not significantly increased.

1.1.2 Secondary Clinical Hypotheses

1.1.2.1 Statin therapy will be associated with reductions in specific CVD-related events and all-cause mortality.

1.1.2.2 Decreases in LDL and non-HDL cholesterol levels associated with statin therapy will be predictive of reduction in CVD events.

1.1.2.3 Statin therapy will reduce serious non-cardiovascular events, including malignancies, end stage kidney or liver disease.

1.1.2.4 Statin therapy will be safe and well tolerated in the HIV-infected population.

1.2 Clinical Objectives

1.2.1 Primary Clinical Objective

To determine the effects of pitavastatin as a primary prevention strategy for MACE in HIV.

1.2.2 Secondary Clinical Objectives

1.2.2.1 To evaluate the effects of pitavastatin on each of the components of the primary composite MACE endpoint and all-cause mortality.

1.2.2.2 To determine the effects of pitavastatin on LDL and non-HDL cholesterol in the HIV population and assess the relationship of changes in LDL and non-HDL to the incidence of MACE.

1.2.2.3 To evaluate whether baseline traditional risk factors (including smoking, hypertension, dyslipidemia, glucose) and time updated HIV-specific (immunological and virological) risk factors are predictive of MACE and pitavastatin effects on MACE in the HIV population.

1.2.2.4 To evaluate whether baseline and time updated inflammatory and immune activation biomarkers are predictive of MACE and pitavastatin effects on MACE in the HIV population.

1.2.2.5 To determine the effects of pitavastatin on the incidence of serious non-cardiovascular events and AIDS-defining events.

1.2.2.6 To determine the safety of pitavastatin in the HIV population, including the development of diabetes mellitus (DM), liver dysfunction, and myopathy.

1.2.2.7 To collect blood to enable the evaluation of the relationship of host genetics to study endpoints in subsequent ancillary studies.

2.0 INTRODUCTION

2.1 Background

Over 34 million people worldwide, including 1.7 million people in the US, are chronically infected with HIV-1 [WHO 2012]. Due to the remarkable success of ART, HIV-infected patients are now living longer [Lewden 2007]. By the year 2015, greater than half of the US HIV-infected population is expected to be 50 or older [WHO 2012; Greene 2013]. Yet, even while ART has reduced AIDS-related deaths [Palella 2006], CVD and CVD-related deaths have increased in the HIV-infected population which has 1.5-2 times increased risk compared to HIV-negative individuals [Sackoff 2006; Gill 2010; Smith 2010]. Currently, there are no treatment strategies proven to prevent CVD in the HIV-infected population, despite the higher risk. A State of the Science Conference on CVD in HIV sponsored by the American Heart Association (AHA) highlighted the critical need to test preventive therapies for CVD in HIV and to develop HIV-specific guidelines [Grinspoon 2008]. Experts agree that existing strategies for CVD prevention in the general population are likely inadequate with increasing evidence suggesting HIV-associated immune activation accelerates the process of atherosclerosis and atherothrombosis [Hsue 2012; Zanni 2012] such that relatively young HIV-infected patients with modest traditional CVD risk factor scores still face high CVD risk. Most of these patients would not meet current guidelines for preventive CVD therapies. The imperative to identify a safe and efficacious strategy for CVD prevention in HIV is best met by a randomized controlled trial of an intervention addressing both traditional and immune CVD risk factors.

2.1.1 Increased Risk of CVD in HIV

CVD-related deaths - including sudden cardiac death - are increased in HIV-infected patients compared with HIV-negative persons [Gill 2010; Tseng 2012]. With respect to acute myocardial infarction (AMI), the most common cause of CVD-related deaths, studies show a 1.5-2 fold increased relative risk of this in HIV-infected individuals versus HIV-negative persons [Klein 2002; Currier 2003; Triant 2007; Obel 2007; Lang 2010; Durand 2011; Freiberg 2013]. Controlling for traditional CVD risk factors does not fully mitigate the heightened CVD risk among HIV-infected individuals [Triant 2007; Freiberg 2013], suggesting a unique pathobiology of atherosclerosis in HIV and highlighting the need for an effective, tailored primary CVD prevention strategy.

2.1.2 Unique Biology of CVD in HIV

2.1.2.1 Inadequate Explanation of CVD Risk in HIV by Traditional CVD Risk Factors

Early studies of CVD in HIV suggested that heightened risk stemmed, indirectly, from an effect of ART to exacerbate traditional CVD risk factors (eg, diabetes, hypertension, dyslipidemia, and abdominal fat accumulation). For example, in the Data Collection on Adverse Events of Anti-HIV Drugs (D:A:D) study, use of protease inhibitors was associated with increased AMI risk, and this risk was

partially attenuated after controlling for dyslipidemia [Friis-Moller 2003]. In contrast, recent studies demonstrate no independent association of ART with AMI and indicate that higher rates of AMI persist with HIV infection despite controlling for traditional CVD risk factors [Triant 2007; Freiberg 2013]. In large epidemiologic studies, immune dysfunction (low CD4 count) and degree of viremia have been identified as important independent contributors to CVD risk in HIV [Triant 2010]. Moreover, data from the Strategies for Management of Antiretroviral Therapy (SMART) study suggest a protective effect of continuous and intensive ART on CVD risk: In the SMART study, patients were randomized to continuous ART or to an ART conservation strategy based on a CD4-guided algorithm. CVD occurred 60% more often in the drug conservation group, highlighting a benefit from more continuous and suppressive ART [El-Sadr 2006]. Subsequent studies from the SMART group have focused on specific markers of inflammation and coagulation that are significantly increased with drug conservation, related to viremia, and independently associated with increased CVD rates [Kuller 2008]. Work from the SMART group thus reinforces the principle that chronic viral infections and the subsequent immune response contribute to CVD, independently of traditional risk pathways highlighting the need to consider a possible role for an immune modulating strategy to prevent CVD.

2.1.2.2 Relationship between HIV-Associated Systemic Immune Activation and CVD Risk

HIV infection induces a paradoxical state of both immune suppression (low CD4 count and increased risk of opportunistic diseases) and immune activation [Deeks 2011]. A paucity of CD4+ T regulatory cells in the gut mucosa enables heightened microbial translocation and ensuing activation of both the innate and adaptive arms of the immune system [Brenchley 2006]. Concomitantly, depletion of circulating CD4+ T regulatory cells, coupled with opportunistic co-infection (hepatitis, cytomegalovirus), results in further immune activation. The net result, in HIV-infected individuals, is persistent activation of circulating monocytes and T-cell subsets [Deeks 2011]. Such chronic immune activation translates into an exhausted T-cell phenotype and a pro-inflammatory milieu that is manifest by higher circulating levels of soluble inflammatory and immune activation markers in HIV-infected persons versus HIV-negative controls. Elevated inflammatory and immune activation biomarkers in HIV-infected patients include pro-inflammatory cytokines (eg, IL-6), acute phase proteins (eg, CRP), leukocyte adhesion molecules (eg, sICAM-1), and fibrin degradation products (eg, d-dimer) [Ross 2008; Dolan 2005; Ross 1999].

Immune activation in HIV is highly relevant to atherosclerosis [Hsue 2012; Zanni 2012] - an inflammatory disease [Libby 2002] resulting in AMI and sudden cardiac death. Atherosclerosis as a process features an intricate interplay between activated immune cells - particularly monocytes - and vascular endothelial cells [Libby 2002; Packard 2009; Koenen 2012; Libby 2011; Hulten 2009]: Circulating monocytes target the tunica intima of affected coronary arteries in response to chemokines and adhesion molecules produced by activated endothelial cells. There, monocytes transform to macrophages and then, upon internalization of oxidized LDL, to foam cells which form the lipid core of the developing atheroma. Plaque macrophages, along with resident T

cells, also secrete cytokines and matrix metalloproteinases, which can degrade the fibrous cap overlying an atheroma, thus precipitating plaque rupture/AMI [Libby 2002; Packard 2009; Koenen 2012; Libby 2011; Hulten 2009]. With HIV infection - a state of persistently activated circulating monocytes and T cells and increased circulating levels of pro-inflammatory cytokines - all stages of atherogenesis and atherothrombosis are likely exacerbated.

2.1.2.3 Novel Atherosclerotic Phenotype in HIV

Initial studies to radiographically characterize subclinical atherosclerosis in HIV focused on carotid intima media thickness (cIMT) [Hulten 2009], which has been found in this population to relate largely to traditional CVD risk factors and not to inflammatory indices [Stein 2013]. In contrast, studies employing coronary computed tomography angiography (CCTA) and cardiac fluorodeoxyglucose-positron emission tomography (FDG-PET) have shown HIV-infected patients to have 1) predominantly non-calcified coronary atherosclerotic plaque [Burdo 2011], 2) a higher prevalence of high risk morphology features (including low attenuation and positive remodeling) [Zanni 2013], and 3) vascular inflammation reflected in clustering of glucose-avid macrophages in the subendothelial matrix [Subramanian 2012; Yarasheski 2012]. The clinical relevance of these observations is that inflamed vulnerable plaque which has yet to calcify is more prone to rupture, resulting in AMI [Rominger 2009; Kitagawa 2009; Hou 2012]. Of note, in HIV-infected patients, non-calcified, vulnerable, and inflamed coronary atherosclerotic plaque has been found to relate to specific markers of monocyte activation, including soluble CD163, and has been observed even in those ART-treated HIV-infected patients with low traditional CVD risk factors [Burdo 2011; Zanni 2013; Subramanian 2012].

2.1.3 Rationale for Statin Therapy to Prevent Primary CVD in HIV

An ideal intervention to prevent CVD in HIV would affect both conventional lipid and HIV-specific immune mediators of CVD and would have minimal risk. Statin therapy uniquely meets these criteria.

2.1.3.1 LDL, Immunomodulatory, and Plaque Stabilizing Effects of Statins

Statins, which address both conventional and inflammatory mechanisms for atherosclerosis, may have unique utility for primary CVD prevention in HIV. In the general population, statins have long been known to potently reduce LDL cholesterol and to prevent CVD events [4S Study 1994; Sacks 1996; Shepherd 1995; Downs 1998]. In addition, statins are known to have pleiotropic anti-inflammatory and immunomodulatory characteristics, which may also contribute to cardio-protective effects [Greenwood 2007]. Indeed, in vitro, animal, and human studies have shown that statins decrease monocyte activation - reflected in a) decreased monocyte chemotaxis and endothelial adhesion [Montecucco 2009; Fujino 2006; Han 2005], b) reduced monocyte uptake of oxidized LDL cholesterol [Han 2004], and c) decreased monocyte secretion of cytokines/chemokines and matrix metalloproteinases [Guo 2009; Waehre 2003]. Moreover, statins decrease T-cell activation [Kwak 2000; Singh 2009; Bu 2010] while recruiting regulatory T cells [Mira 2008], suppress endothelial cell

activation [Zheng 2013; Romano 2000; Mulhaupt 2003; Veillard 2006; Zineh 2006], and decrease lipid oxidation [Aviram 1992; Giroux 1993; Vasankari 2001]. Further, data from cardiovascular imaging studies in HIV-negative subjects reveal that statins stabilize vulnerable coronary atherosclerotic plaque and even induce plaque regression. Specifically, statins have been shown to decrease atherosclerotic plaque vulnerability features on CCTA and intravascular ultrasound (IVUS) [Shimojima 2012; Inoue 2010; Kodama 2010; Nakamura 2008], to reduce atherosclerotic plaque inflammation on cardiac FDG-PET [Tawakol 2013], and to reduce non-calcified plaque volume [Burgstahler 2007; Hiro 2009]. Based on these data, statins may be uniquely tailored to address important mechanisms of CVD in HIV.

2.1.3.2 Statin Effects on Non-CVD Events in HIV

Due to the efficacy and widespread use of potent combination ART, mortality patterns among HIV patients have changed, with a decline in the proportion due to AIDS and a concomitant rise in the proportion due to non-AIDS-related diseases [Weber 2013; DAD 2010; Palella 2006; Wada 2013; Neuhaus 2010; ARTCC 2010; Marin 2009; French 2009; Lewden 2008]. In this regard, cardiovascular, end-stage liver and renal disease, and non-AIDS-related malignancies represent important causes of mortality among HIV patients in the current era of potent ART. Based on animal models and in-vitro and in-vivo data that statins may decrease the systemic inflammation that has been associated with many of these events, and epidemiologic data suggesting statins alter the risk of these events, including these key comorbidities in a secondary endpoint analysis of REPRIEVE (A5332) is warranted.

Beyond an excellent safety record in persons with liver disease, current data suggest that statins may improve various aspects of liver disease. In animal models of non-alcoholic fatty liver disease, several different statins, including pitavastatin, have improved hepatic steatosis and decreased fibrosis mediated by anti-inflammatory and antifibrotic effects [Tarantino 2012; Miyaki 2011; Hyogo 2012; Wang 2013]. Human trials have demonstrated similar improvements in steatosis and liver fibrosis [Gomez-Dominguez 2006; Foster 2011; Ekstedt 2007; Simon 2014].

Statins appear to have a beneficial effect on kidney function, particularly among persons with pre-existing microalbuminuria, a common finding among HIV-infected patients [Amarenco 2014; Colhoun 2009]. Meta-analyses have demonstrated either a modest benefit or uncertain effects of statins on renal function [Nikolic 2013; Douglas 2006; Palmer 2014]. The limited data in HIV patients indicated improvement in renal function with rosuvastatin that correlated with reductions in inflammatory biomarkers and T-cell activation [Longenecker 2014].

HIV infection remains associated with an increased risk of cancer [Crum-Cianflone 2009; Deeken 2012]. While there are numerous factors that contribute to the excess risk of cancer, persistent inflammation and alterations in the immune system, particularly altering normal tumor surveillance, are considered key contributors to the excess risk, even in the setting of viral suppression and partial immunologic reconstitution experienced with ART

[Borges 2014; Tenorio 2014]. Statins have been demonstrated to have anticancer activity which have been related to consequences of blocking the mevalonate pathway, including arresting cell cycle progression, inducing apoptosis, reducing oxidative stress, and decreasing systemic inflammation [Carlberg 1996; Wong 2002; Yasui, 2007]. While the data from observational studies and post-hoc analyses for cancers in the general population have failed to consistently demonstrate an anticancer effect for statins, data from studies including HIV-infected populations, while limited, suggest potential anticancer effects of statins due to the fact that persistent inflammation is likely an important driver [Galli 2014; Overton 2013].

Despite a declining incidence, AIDS events continue to occur and remain the leading cause of death among HIV-infected persons. Clearly, advanced immunosuppression and a maladaptive inflammatory response, both related to uncontrolled HIV infection, are important drivers of the underlying mechanisms of AIDS events. While there are limited data regarding the impact of statins on the risk of developing AIDS events, the effects of statins on markers of both cellular and soluble markers of inflammation indicate that there may be benefit to assess the effect of statins on AIDS events, in conjunction with end stage renal and liver disease, as well as cancers in REPRIEVE (A5332). These data are critical to obtain in order to understand the potential overall impact of statins on HIV-infected patients.

2.1.3.3 Efficacy of Statin Therapy in HIV

In HIV-infected individuals, statins safely and effectively lower LDL cholesterol [Silverberg 2009; Ganesan 2011; Calza 2012]. and also exert immunomodulatory effects to 1) decrease monocyte activation - reflected in decreased circulating levels of sCD14 and the macrophage-derived phospholipase, Lp-PLA2 [McComsey 2013] and 2) decrease T-cell activation in some studies [Ganesan 2011; DeWit 2011]. Statins are generally well tolerated among HIV-infected patients and introduce minimal risks balanced against their significant potential to prevent CVD in this population [Singh 2011]. In a large cohort study, Silverberg et al. demonstrated that statins can be safely administered to HIV-infected patients. Increased relative rates of grade III myositis (1.9% vs. 0.5%) and liver function test (LFT) abnormalities (1.1% vs. 0.3%) were seen in HIV-infected patients versus control subjects, but absolute rates were low [Silverberg 2009]. With respect to the potential to induce DM development with statin use in HIV-infected patients, non-randomized cohort studies recently reported contrasting results, with one study showing increased DM risk [Lichtenstein 2013] and two others suggesting no increased risk [Spagnuolo 2013; Overton 2013]. Unlike other agents with immune suppressant effects [Paton 2013], statins have been not been shown to have adverse effects on viral replication [Moncunill 2005; Negredo 2006]. Indeed, in vitro studies suggest numerous mechanisms through which statins may actually reduce viral replication although clinical data suggests little impact on measured changes in plasma HIV viral loads while on statin therapy [Nabatov 2007; Giguere 2004; Gilbert 2005; del Real 2004; Amet 2008].

2.1.3.4 Selection of Pitavastatin

Several statins are currently FDA approved and commercially available, with a limited number available in generic form; however the use of select statins in HIV-infected patients is complicated by, or in some cases contraindicated based on, complex interactions with antiretroviral agents [Aberg 2013; Aberg 2009; Ahmed 2012]. Most statins are primarily metabolized by the CYP3A4 system. HIV protease inhibitors inhibit CYP3A4 and thus markedly increase exposure to these statins, as reflected in the area under the plasma drug concentration-time curve (AUC) [Chauvin 2013]. As such, simvastatin and lovastatin are contraindicated in HIV-infected patients taking protease inhibitors. Atorvastatin is partially metabolized by the CYP3A4 system, and the exposure to this statin is moderately increased by protease inhibitor therapy. Pravastatin exposure may be decreased by co-administration with ritonavir, but increased with co-administration with ritonavir-boosted darunavir. Similarly, rosuvastatin exposure is increased by co-administration with ritonavir-boosted darunavir [Samineni 2012]. In contrast, no clinically significant effects of darunavir or lopinavir/ritonavir on pitavastatin were seen [Aberg 2013; Malvestutto 2014]. Other effects are also seen, for example between efavirenz, a widely prescribed ART, on the AUC of various common statins, including atorvastatin (-43% AUC), pravastatin (-40% AUC), and simvastatin (-58% AUC) [Gerber 2005], in contrast there was only an 11% change in pitavastatin AUC with efavirenz [Malvestutto 2014].

Pitavastatin is a relatively new statin with comparable efficacy in terms of LDL reduction and anti-inflammatory effects as other potent statins [Eriksson 2011]. It is an excellent choice for HIV-infected persons as it is not metabolized by the CYP3A4 system but is instead metabolized primarily by glucoronidation [Gotto 2010]. Consequently, there are minimal documented interactions between pitavastatin and antiretroviral agents, including minimal effects of pitavastatin on exposure to ritonavir (+8% AUC), darunavir (+3% AUC), atazanavir (+6% AUC), lopinavir (-9% AUC) [FDA package insert]. Similarly, relatively modest effects of individual antiretroviral drugs are seen with respect to pitavastatin AUC [FDA package insert]. Indeed, there is no contraindication or recommendation for dose adjustment when pitavastatin is used in concert with any specific antiretroviral agent. Importantly, pitavastatin 4 mg/day has been shown to effectively decrease LDL cholesterol among ART-treated HIV-infected persons: the INTREPID study randomized HIV-infected individuals to pitavastatin 4 mg/day versus pravastatin 40 mg/day [Sponseller 2013]. Results demonstrated that pitavastatin was superior to pravastatin with respect to LDL cholesterol lowering (-49.4 mg/dL (-31.1%) vs. -33.6 mg/dl (-20.9%)) over a 12-week period with results sustained over 52 weeks [Sponseller CROI 2014]. Moreover, relative to pravastatin, pitavastatin showed significantly greater ability to lower total cholesterol, non-HDL cholesterol, TC:HDL ratio, and Apo B lipoprotein levels. Tolerability and toxicity profiles were similarly benign for both agents: myalgias and LFT abnormalities were rare in both groups, no effects on HIV RNA or CD4 were seen. Of note, pitavastatin, like pravastatin, had a neutral effect on blood glucose and HgbA1c levels in the HIV-infected population [Aberg Endo 2013]. Pitavastatin has compared favorably to other statins in randomized trials among non-HIV infected patients as well [Eriksson 2011; Eriksson 2011; Gumprecht 2011; Maruyama 2011]. Additionally,

pitavastatin has demonstrated little impact on glucose metabolism and may even improve insulin resistance, an additional benefit when compared to other statins [Yokote 2009; Yamakawa 2008; Teramoto 2010; Aberg 2013].

2.1.4 Opportune Timing for Randomized Control Trial of Statin Therapy in HIV

The JUPITER trial demonstrated that among individuals in the general population with LDL cholesterol <130 mg/dL and moderate inflammation (defined as high sensitivity C-reactive protein >2 mg/L), statin therapy resulted in a 44% reduction in CVD events [Ridker 2008]. To date, there are no large scale randomized trials of statin therapy to prevent CVD among HIV-infected individuals. Non-randomized studies in the HIV-infected population demonstrate that use of statins is associated with reduced overall mortality [Moore 2011], especially among those diagnosed with co-morbidities [Rasmussen 2013]. Moore et al. showed that statin use was associated with a with a three-fold reduction in hazard ratio for all-cause mortality among HIV patients, adjusting for age, CD4, HIV-1 RNA, cholesterol levels, and prior ART use [Moore 2011]. Observational studies also show a trend toward reduction in non-AIDS complications among ART-treated HIV-infected patients on high potency statins [Dreschler 2013].

Despite the potential appeal of statins, their use is relatively low in HIV-infected patients - 19.6% in a 2013 survey commissioned from the ACTG. Another recent analysis reported significant clinical inertia for the utilization of statin therapy in well controlled HIV-infected persons highlighting that both patient and provider factors weigh into the decision whether to utilize statins [Willig 2008]. The low use of statins in the HIV-infected population reflects in part the relatively low prevalence of increased LDL cholesterol in this group [Freiberg 2013], but more importantly, the uncertain efficacy for CVD prevention and the potential side effects and ART interactions. These factors suggest equipoise and opportune timing for a large randomized trial to assess efficacy and safety of a statin therapy strategy for primary CVD prevention in HIV among those not meeting recommendations for statins under the 2013 ACC/AHA guidelines.

In 2013, the ACC/AHA released Blood Cholesterol Guidelines to replace the guidelines from the Third Report of the Expert Panel on Detection, Evaluation, and Treatment of High Blood Cholesterol in Adults (Adult Treatment Panel III). Novel aspects of the guidelines included 1) delineation of four statin benefit groups – patients age ≥21 with clinical ASCVD and/or with LDL cholesterol ≥190 mg/dL and patients age ≥40 and ≤75 with diabetes and/or with 10 year ASCVD risk estimated to be ≥7.5% by the Pooled Cohort Equations and 2) abandonment of LDL cholesterol and non-HDL cholesterol treatment goals. In addition, the new guidelines deemphasized the use of non-statin lipid lowering therapies and focused exclusively on the use of statins for primary CVD prevention [Stone 2013]. In this regard, pitavastatin is included as a recommended therapy in the new guidelines for primary prevention. Although these guidelines are now recommended for general use in clinical practice, they do not incorporate into risk prediction novel HIV-specific factors, including degree of systemic immune activation. REPRIEVE (A5332) provides a critical opportunity, in line with the hypothesis of the grant, to test whether patients without significant traditional risk, eg, those with ASCVD scores <7.5%, are an appropriate group for statin primary prevention. The currently proposed trial achieves equipoise by testing the efficacy and safety of statin therapy for primary CVD prevention in HIV among those not meeting recommendations for statins under the 2013 ACC/AHA guidelines and speaks directly to critical knowledge gaps identified by the recently published ACC/AHA guidelines for cholesterol management

and the AHA sponsored State of the Science Conference on CVD in HIV. In alignment with these guidelines, subjects aged 40-75 will be recruited for REPRIEVE (A5332). In order to be fully aligned with the 2013 ACC/AHA Guidelines, persons with diabetes and LDL <70mg/dL or ASCVD risk score ≥7.5% and LDL <70mg/dL are not recommended to initiate statins, thus these two groups will be included in the study.

Although we do not anticipate a change in the guidelines occurring during the REPRIEVE (A5332) study, we will address any future changes in the guidelines by obtaining Data Safety Monitoring Board (DSMB) review to determine whether equipoise has been disturbed and whether any changes to the study should be made as a result, updating the informed consent documents to inform participants about the change, and providing participants with a letter describing the new guidelines to take to their personal physicians to determine whether they should continue to participate in the study in light of the change.

2.2 Rationale

HIV-infected persons face an increased risk of CVD morbidity and mortality, yet no preventive strategies for CVD risk reduction have been proven for this population. Existing primary CVD preventive strategies for the general population cannot simply be extrapolated to HIV-infected individuals, in whom immune dysfunction/activation contributes in unique ways to atherosclerosis and ensuing cardiovascular events. The rationale of testing statins for primary CVD prevention in HIV is multifold: First, statins affect both traditional CVD risk factors (LDL cholesterol) and have beneficial pleiotropic “off-target” effects, ie, reduction in immune activation. The latter effect is critical, as immune activation and persistent inflammation in HIV are thought to contribute importantly to the development of non-calcified, vulnerable, and inflamed coronary atherosclerotic plaque even in relatively young HIV-infected patients on ART with low traditional CVD risk indices [Zanni 2013; Subramanian 2012; Yarasheski 2012]. In such patients, statin therapy may stabilize high-risk coronary atherosclerotic plaque, precluding rupture and AMI. Moreover, statins are generally safe in the HIV population, exerting anti-inflammatory properties without enhancing viremia [Silverberg 2009; McComsey 2013; Sponseller 2013; Negredo 2006; Nabatov 2007; Ganesan 2011].

Based on this rationale, the REPRIEVE (A5332) study was designed to assess the efficacy of statins as a primary prevention strategy for CVD events in HIV-infected persons on ART not meeting 2013 ACC/AHA guideline thresholds for recommended statin initiation. The study will also definitively determine the safety of statins in the HIV-infected population, including statin effects on non-CVD events such as incident diabetes, malignancies, kidney or liver failure, and AIDS-defining events in HIV-infected persons. In addition to enrolling patient in the main study, select sites will have the option of co-enrolling subjects in a Mechanistic Substudy of REPRIEVE (A5333s). As part of this substudy, described in detail in Appendix II, co-enrolled patients will undergo detailed coronary CT angiography and biochemical immunophenotyping. The Mechanistic Substudy of REPRIEVE (A5333s) will allow for determination of statin effect on non-calcified coronary atherosclerotic plaque burden and morphology, as well as lipid and immune parameters predictive of this effect. REPRIEVE (A5332) addresses an urgent national healthcare priority to prevent CVD among persons living with HIV infection.

3.0 STUDY DESIGN

REPRIEVE (A5332) is a prospective, double-blind, randomized, placebo-controlled, multicenter phase IV efficacy study that will examine the effects of 4 mg daily pitavastatin on cardiovascular-related events among HIV-1 infected adults who are currently on ART. The randomization in the study will be stratified by sex at birth, CD4+ T-cell counts (≤500 vs. >500 cells/mm^3), and by whether or not a subject has elected to participate in the Mechanistic Substudy of REPRIEVE (A5333s) (yes/no).

6500 HIV-1 infected men and women ≥40 and ≤75 years of age, on any ART regimen (ART not provided by the study) for at least 6 months prior to study entry, with any plasma HIV-1 RNA level, with CD4+ T-cell count >100 cells/mm^3 not meeting 2013 ACC/AHA guideline thresholds for recommended statin initiation (no known clinical ASCVD, LDL <190 mg/dL and 10-year ASCVD risk score estimated by Pooled Cohort Equations <7.5%) will be enrolled into this study.

At study entry, subjects will be randomized to one of the following arms:

<u>ARM A</u>: At Day 0, initiate pitavastatin at a daily dose of 4 mg.

<u>ARM B</u>: At Day 0, initiate placebo for pitavastatin daily.

Clinical assessments will be performed at month 1, month 4, and then every 4 months for the duration of the study. Total study duration will be approximately 72 months from the time of first participant enrolled.

4.0 SELECTION AND ENROLLMENT OF SUBJECTS

4.1 Inclusion Criteria

4.1.1 HIV-1 infection, documented by any licensed rapid HIV test or HIV enzyme or chemiluminescence immunoassay (E/CIA) test kit at any time prior to study entry and confirmed by a licensed Western blot or a second antibody test by a method other than the initial rapid HIV and/or E/CIA, or by HIV-1 antigen, plasma HIV-1 RNA viral load.

NOTE: The term “licensed” refers to a US FDA-approved kit, which is required for all IND studies. Non-US sites are encouraged to use FDA-approved methods. If an FDA-approved kit is not available, then each non-US site must use a kit that has been certified or licensed by an oversight body within that country and validated internally.

WHO (World Health Organization) and CDC (Centers for Disease Control and Prevention) guidelines mandate that confirmation of the initial test result must use a test that is different from the one used for the initial assessment. A reactive initial rapid test should be confirmed by either another type of rapid assay or an E/CIA that is based on a different antigen preparation and/or different test principle (eg, indirect versus competitive), or a Western blot or a plasma HIV-1 RNA viral load.

4.1.2 Combination antiretroviral therapy (ART) for at least 180 days prior to study entry.

NOTE: Treatment interruptions for up 14 days total in the last 180 days are permitted as long as the subject has been continuously on therapy for the 30 days prior to study entry.

4.1.3 CD4+ cell count >100 cells/mm^3 obtained from standard of care within 180 days prior to study entry at any US laboratory that has a Clinical Laboratory Improvement Amendments (CLIA) certification or its equivalent, or at any network-approved non-US laboratory that operates in accordance with Good Clinical Laboratory Practices and participates in appropriate external quality assurance programs.

4.1.4 Laboratory values drawn at screen and/or obtained from clinical care (as indicated in Table 6.1 Schedule of Events) within 90 days prior to study entry at any US laboratory that has a Clinical Laboratory Improvement Amendments (CLIA) certification or its equivalent, or at any network-approved non-US laboratory that operates in accordance with Good Clinical Laboratory Practices and participates in appropriate external quality assurance programs.

- Fasting LDL cholesterol <190 mg/dL
- Fasting triglycerides <500 mg/dL

NOTE: Fasting is defined as nothing to eat or drink except water and required prescription medications for at least 8 hours. Drinking black decaffeinated coffee without sweetener or creamer is permissible, but is not advised.

- Hemoglobin ≥8 g/dL for female subjects and ≥9 g/dL for male subjects
- Calculated creatinine clearance (CrCl) ≥60 mL/min, as estimated by the Cockcroft-Gault equation

NOTE: Refer to the calculator for the Cockcroft-Gault equation in the MOPS.

- ALT ≤2.5 x ULN

NOTE: Subjects co-infected with chronic active hepatitis B or C must have ALT ≤2 x ULN.

4.1.5 For persons with known chronic active hepatitis B or C, calculated FIB-4 score must be ≤3.25.

NOTE: Active is defined as hepatitis B surface antigen positive, hepatitis B DNA positive, or hepatitis C RNA positive.

NOTE: Refer to the calculator for the FIB-4 equation in the MOPS.

4.1.6 Female subjects of reproductive potential (defined as women who have not been post-menopausal for at least 24 consecutive months, ie, who have had menses within 24 months prior to study entry, and women who have not undergone surgical sterilization, specifically hysterectomy or bilateral oophorectomy) must have a negative serum or urine pregnancy test within 48 hours prior to entry by any US laboratory that has a CLIA certification or its equivalent, or at any network-approved non-US laboratory that operates in accordance with Good Clinical Laboratory Practices and participates in appropriate external quality assurance programs.

NOTE: Subject-reported history is considered acceptable documentation of hysterectomy, bilateral oophorectomy, tubal ligation, tubal micro-inserts, or menopause. Women are considered menopausal if they have not had a menses for at least 12 months and have a FSH (follicle stimulating hormone) of greater than 40 IU/L or, if FSH testing is not available, they have had amenorrhea for 24 consecutive months.

4.1.7 For women of child-bearing potential, willingness to use contraceptives as described in the product information for pitavastatin. Contraceptives must be used at least two weeks before initiation of study drug and must be continued 6 weeks after cessation of study drug.

If participating in sexual activity that could lead to pregnancy, women must use a form of contraceptive. At least one of the following methods must be used appropriately:

- Condoms (male or female) with spermicidal agent
- Diaphragm or cervical cap with spermicidal agent
- Intrauterine device (IUD)
- Hormone-based contraceptive

Women who are not of reproductive potential as defined above are eligible without the use of contraception.

4.1.8 Men and women age ≥40 and ≤75 years of age.

4.1.9 Ability and willingness of subject or legal representative to provide written informed consent.

4.2 Exclusion Criteria

4.2.1 Clinical ASCVD, as defined by 2013 ACC/AHA guidelines, including a previous diagnosis of any of the following:

- AMI
- Acute coronary syndromes
- Stable or unstable angina
- Coronary or other arterial revascularization
- Stroke
- TIA
- Peripheral arterial disease presumed to be of atherosclerotic origin

4.2.2 Current diabetes mellitus if LDL >70 mg/dL

NOTE: Current diabetes is defined by patient report of physician diagnosis. Subjects with a history of diabetes which has resolved and no longer requires therapy are not considered to have current diabetes, eg, women with a history of gestational diabetes, steroid-induced or medication-induced.

4.2.3 10-year ASCVD risk score estimated by Pooled Cohort Equations ≥7.5%

NOTE: The ACC/AHA 2013 Prevention Guidelines Calculator Tool link is located in section 6.3.4.

NOTE: Subjects with ASCVD risk score ≥7.5% who also have an LDL cholesterol level <70 mg/dl would, however, be permitted to enroll, in line with the 2013 ACC/AHA Cholesterol Guidelines.

4.2.4 Active cancer within 36 months prior to study entry.

NOTE: Subjects with successfully treated non-melanomatous skin cancer within 36 months prior to study entry are acceptable. Disseminated Kaposi Sarcoma (visceral organ involvement) within the past 36 months is exclusionary.

4.2.5 Known cirrhosis.

4.2.6 History of myositis or myopathy with active disease in the 180 days prior to study entry.

4.2.7 Known untreated symptomatic thyroid disease.

4.2.8 History of allergy or severe adverse reaction to statins.

4.2.9 Use of immunosuppressants or immunomodulatory agents including tacrolimus, sirolimus, rapamycin, mycophenolate, cyclosporine, TNF-alpha blockers or antagonists, azathioprine, interferon, growth factors, or intravenous immunoglobulin (IVIG) in the 30 days prior to study entry.

4.2.10 Current use of erythromycin, colchicine, or rifampin.

4.2.11 Use of any lipid lowering agents including statin drugs, fibrates, ezetimibe, red yeast rice, niacin or omega-3 fatty acids (>3 grams/day in standalone formulations) in the 90 days prior to study entry.

NOTE: Use of niacin as part of a multivitamin is not exclusionary.

4.2.12 Current use of an investigational new drug which would be contraindicated.

NOTE: Please contact the protocol core team via e-mail as described in the Study Management section for guidance on co-enrollment of subjects on investigational new drugs.

4.2.13 Serious illness or trauma requiring systemic treatment or hospitalization in the 30 days prior to study entry.

4.2.14 Known active or recent (not fully resolved within 30 days prior to study entry) systemic bacterial, fungal, parasitic, or viral infections (except HIV, HBV, human papillomavirus [HPV], or HCV).

4.2.15 Current breastfeeding.

4.2.16 Alcohol or drug use that, in the opinion of the site investigator, would interfere with completion of study procedures.

4.2.17 Other medical, psychiatric, or psychological condition that, in the opinion of the site investigator, would interfere with completion of study procedures and or adherence to study drug.

4.3 Study Enrollment Procedures

4.3.1 Prior to implementation of this protocol, and any subsequent full version amendments, each site must have the protocol and the protocol consent form(s) approved, as appropriate, by their local institutional review board (IRB)/ethics committee (EC) and any other applicable regulatory entity (RE). Upon receiving final approval, sites will submit all required protocol registration documents to the DAIDS Protocol Registration Office (DAIDS PRO) at the Regulatory Support Center (RSC). The DAIDS PRO will review the submitted protocol registration packet to ensure that all of the required documents have been received.

Initial site-specific informed consent forms (ICFs) will be reviewed and approved by the DAIDS PRO, and sites will receive an Initial Registration Notification from the DAIDS PRO that indicates successful completion of the protocol registration process. A copy of the Initial Registration Notification should be retained in the site's regulatory files.

Upon receiving final IRB/EC and any other applicable RE approvals for an amendment, sites should implement the amendment immediately. Sites are required to submit an amendment registration packet to the DAIDS PRO at the RSC. The DAIDS PRO will review the submitted protocol registration packet to ensure that all the required documents have been received. Site-specific ICF(s) WILL NOT be reviewed and approved by the DAIDS PRO, and sites will receive an Amendment Registration Notification when the DAIDS PRO receives a complete registration packet. A copy of the Amendment Registration Notification should be retained in the site's regulatory files.

For additional information on the protocol registration process and specific documents required for initial and amendment registrations, refer to the current version of the DAIDS Protocol Registration Manual: http://www.niaid.nih.gov/LabsAndResources/resources/DAIDSClinRsrch/Documents/prmanual.pdf.

Once a candidate for study entry has been identified, details including risks and benefits will be carefully discussed with the participant. The participant (or, when necessary, the legal representative if the participant is under guardianship) will be asked to read and sign the approved protocol consent form.

For participants from whom a signed informed consent has been obtained, an ACTG Screening Checklist must be entered through the Data Management Center (DMC) Participant Enrollment System.

4.3.2 Subject Randomization

Subjects will be randomized according to standard ACTG data management procedures. For subjects from whom informed consent has been obtained, but who are deemed ineligible or who do not enroll into the initial protocol step, an ACTG Screening Failure Results form must be completed and keyed into the database.

4.4 Mechanistic Substudy of REPRIEVE (A5333s) Enrollment Procedures

If applicable, the Mechanistic Substudy of REPRIEVE (A5333s) enrollment will occur at the same time as enrollment into REPRIEVE (A5332). Please see Appendix II section 4.3 for additional information regarding substudy enrollment procedures.

4.5 Coenrollment Guidelines

US ACTG sites are encouraged to coenroll subjects in A5128, "Plan for Obtaining Informed Consent to Use Stored Human Biological Materials (HBM) for Currently Unspecified Analyses." Non-US ACTG sites are encouraged to coenroll subjects in A5243, "Plan for Obtaining Human Biological Samples at Non-US Clinical Research Sites for Currently Unspecified Genetic Analyses." Coenrollment in A5128, observational studies, or studies that do not involve a random treatment assignment does not require permission from the REPRIEVE (A5332) protocol team.

For specific questions and approval for coenrollment in other studies (including HCV or HIV treatment trials and adjunctive treatment trials), sites should first check the PSWP or contact the protocol core team via e-mail as described in the Study Management section.

4.6 Retention Procedures

Subjects who miss visits should be contacted to make up the visit and to permit determination of whether a major event occurred. If a subject refuses to communicate with study staff in any context and avoids further participation in the study after repeated attempts or the site is unable to contact the subject by phone, mail, primary care physician, next of kin, home visits, or emergency contact numbers after repeated attempts, then subjects will be considered lost to study by the DMC when the Off-Study Form is entered indicating that the participant is lost. Participants who self-withdraw from study medications should be followed (if they agree) and evaluated; these patients will not be considered lost to study. See MOPS for procedures to contact patients after missed visits.

5.0 STUDY TREATMENT

Study treatment is defined as pitavastatin and placebo for pitavastatin, both of which will be provided by the study.

5.1 Regimens, Administration, and Duration

5.1.1 Regimens

At study entry (day 0), subjects will be randomized to one of the following arms:

ARM A: Pitavastatin 4 mg one tablet once daily taken orally with or without food.

ARM B: Placebo for pitavastatin one tablet once daily taken orally with or without food.

5.1.2 Administration

The appropriate dose of pitavastatin or placebo for pitavastatin can be administered at any time of the day, with or without food. The drug should be taken as close to the same time of day as possible. Subjects must begin treatment within 72 hours after randomization. For subjects coenrolled in the Mechanistic Substudy (A5333s), treatment should not begin until after the entry CCTA (see MOPS for more details).

Study product will be dispensed in accordance with the Drug Dispensation schedule as described in the MOPS. Subjects will be instructed to return any remaining study product at each study visit to ascertain adherence and in order that the dispensing schedule can be followed.

5.1.3 Treatment Duration

Subjects will remain on study treatment for approximately 42 to 72 months depending on the time they enrolled in the study.

5.2 Study Product Formulation and Preparation

Pitavastatin and placebo for pitavastatin should be stored at room temperature between 15–30°C (59–86°F). The product should be stored out of direct sunlight. The container should be kept tightly closed retaining the silica gel desiccant in the bottle. Dispense only in the original container.

5.3 Pharmacy: Product Supply, Distribution, and Accountability

5.3.1 Study Product Acquisition/Distribution

Pitavastatin and placebo for pitavastatin will be provided by Kowa Pharmaceuticals America, Inc. These study products will be available through the NIAID Clinical Research Products Management Center (CRPMC). The clinical research site (CRS) pharmacist can obtain the study products for this protocol by following the instructions in the manual *Pharmacy Guidelines and Instructions for Division of AIDS (DAIDS) Clinical Trials Networks*.

5.3.2 Study Product Accountability

The site pharmacist is required to maintain complete records of all study products received from the NIAID CRPMC and subsequently dispensed. All unused study products must be returned to the NIAID CRPMC (or as otherwise directed by the sponsor) after the study is completed or terminated. The procedures to be followed are provided in the manual *Pharmacy Guidelines and Instructions for DAIDS Clinical Trials Networks* in the section Study Product Management Responsibilities. At non-US CRSs, the site pharmacist must follow the instructions in the *Pharmacy Guidelines and Instructions for DAIDS Clinical Trials Networks* for the destruction of unused study products.

5.4 Concomitant Medications

Whenever a concomitant medication or study agent is initiated or a dose changed, investigators must review the concomitant medication's and study agent's most recent package insert, Investigator's Brochure, or updated information from DAIDS to obtain the most current information on drug interactions, contraindications, and precautions.

Additional drug information may be found on the updated ACTG Drug Interactions Database located at: http://tdm.pharm.buffalo.edu/home/di_search/Search.

5.4.1 Required Medications

- Combination ART for at least 180 days prior to study entry.

  NOTE: Treatment interruptions for up 14 days total in the last 180 days are permitted as long as subject has been continuously on therapy for the 30 days prior to study entry.

5.4.2 Recommended Medications

- *Pneumocystis jiroveci* pneumonia (PCP) prophylaxis for CD4+ T-cells <200 cells/mm^3 is strongly recommended.
- *Mycobacterium avium* prophylaxis for CD4+ T cells per US Department of Health & Human Services guidelines is strongly recommended.

5.4.3 Prohibited Medications

- Refer to the PSWP for a list of prohibited medications.

5.4.4 Precautionary Medications

- Refer to the PSWP for a list of precautionary medications.

6.0 CLINICAL AND LABORATORY EVALUATIONS

6.1 Schedule of Events

Evaluation	Screen Ing[2]	Entry Day[2] 0	Post-Entry Evaluations (*months*)[1] Visit Window ± 7 days for month 1; ± 14 days for all other visits																			Study Termination Visit[2]	Prem. Treatment Disc. Evals ± 14 days[2]	Prem. Study Disc. Evals[2]
			1	4	8	12[2]	16	20	24[2]	28	32	36[2]	40	44	48[2]	52	56	60[2]	64	68	72[2]			
Documentation of HIV-1	X																							
Medical history	X	X[3]	X	X	X	X	X	X	X	X	X	X	X	X	X	X	X	X	X	X	X	X	X	X
Medication History	X	X[3]	X	X	X	X	X	X	X	X	X	X	X	X	X	X	X	X	X	X	X	X	X	X
Myalgia Symptom Assessment		X	X	X	X	X	X	X	X	X	X	X	X	X	X	X	X	X	X	X	X	X	X	X
Calculate ASCVD Risk Score	X																							
CV risk factor assessment	X																							
REAP & DASI Questionnaires		X																			X	X	X	X
Screening physical exam	X																							
Targeted physical exam			X			X			X			X			X			X			X	X	X	X
Height	X																							
Weight	X					X			X			X			X			X			X	X	X	X
Waist circumference	X																					X	X	X
ECG[4]		X																						
Dispense lifestyle information		X				X			X			X			X			X			X	X	X	X
Labs captured from standard of care																								
CD4+/CD8+[5]	X					X			X			X			X			X			X	X	X	X
Plasma HIV-1 RNA[6]		X				X			X			X			X			X			X	X	X	X
CBC[7]	X																				X	X	X	X
Serum Creatinine[7]	X																				X	X	X	X
Calculated Creatinine Clearance	X																							
Labs to be collected (drawn)																								
Fasting lipid panel (local lab) [8]	X																							

Evaluation	Screen Ing[2]	Entry Day[2] 0	Post-Entry Evaluations (*months*)[1] Visit Window ± 7 days for month 1; ± 14 days for all other visits																			Study Termination Visit[2]	Prem. Treatment Disc. Evals ± 14 days[2]	Prem. Study Disc. Evals[2]
			1	4	8	12[2]	16	20	24[2]	28	32	36[2]	40	44	48[2]	52	56	60[2]	64	68	72[2]			
Fasting Lipid Panel (stored)[9]		X				X			X			X			X			X			X	X	X	X
ALT (local lab)	X[10]		X			X																		
AST (local lab)	X[10]																							
FIB-4[10]	X																							
Pregnancy testing	X	X	X	X	X	X	X	X	X	X	X	X	X	X	X	X	X	X	X	X	X	X	X	X
Fasting glucose[9]		X				X			X			X			X			X			X	X	X	X
Urine albumin/creatinine		X																						
Fasting plasma/serum for biomarkers – stored		X																			X	X	X	X
Whole blood for genetic testing[11]		X																						
Safety labs if indicated[12]			X	X	X	X	X	X	X	X	X	X	X	X	X	X	X	X	X	X	X	X	X	X
Dispense study drug		X		X	X	X	X	X	X	X	X	X	X	X	X	X	X	X	X	X				
Pill count for study drug			X	X	X	X	X	X	X	X	X	X	X	X	X	X	X	X	X	X	X	X	X	X
Patient self-adherence questionnaire			X	X	X	X	X	X	X	X	X	X	X	X	X	X	X	X	X	X	X	X	X	X
Vital Status Follow-up																					X	X	X	X
Endpoint Assessments			X	X	X	X	X	X	X	X	X	X	X	X	X	X	X	X	X	X	X	X	X	X

[1] For the purposes of the study 1 month equals 30 days.
[2] If a subject is not fasting for a visit for which fasting blood is obtained, their appointment may be rescheduled within 7 days of the original appointment date.
[3] Update any changes from the screening assessment (medical and medication history).
[4] ECG available to site PI, all ECGs will be sent for central read and storage until end of study, no specific ECG findings are exclusionary, except findings which in the judgment of PI render the patient clinically unstable as per clinical read, central readings will not be available to sites, see MOPS.
[5]At screening visit, CD4+/CD8+ will be obtained from standard of care within 180 days prior to entry, if not available or absolute CD4+ is not >100 cells/mm^3 this will be drawn as part of the study. For post entry CD4+/CD8+, the most recent values obtained since the last annual visit may be used.
[6] For the entry visit, an HIV viral load from standard of care from the prior 180 days will be used. For post-entry evaluations, the most recent viral load obtained from standard of care since the last annual study visit will be used.
[7]At screening visit, results of CBC and creatinine will be obtained from standard of care if available within 90 days prior to entry. At end of study visit, these results will be obtained from standard of care if available within 90 days prior to this visit. If not available, they will be drawn as part of the study.
[8] Direct LDL will be determined if triglyceride 400-500 mg/dL for screening labs.
[9] Fasting lipid panels and glucose from entry visit forward will be performed centrally and the results will be blinded until the end of the study.
[10]For persons with HCV and/or HBV, AST should also be performed at screening to facilitate calculation of FIB-4.
[11]Whole blood for genetic testing will be drawn at ACTG sites only.
[12]Additional safety labs will be checked at the discretion of site investigator based on subject symptoms (see section 7.1).

6.2 Timing of Evaluations

NOTE: For the purposes of the study, 1 month = 30 days.

6.2.1 Screening Evaluations

- Screening evaluations must occur prior to randomization and any study treatment or intervention.
- Screening evaluations to determine eligibility must be completed within 90 days prior to entry unless otherwise specified.
- In addition to data being collected on subjects who enroll into the study, demographic, clinical, and laboratory data on screening failures will be captured in a Screening Failure Results form and entered into the ACTG database.

6.2.2 Entry Evaluations

- Entry evaluations will normally occur at least 24 hours after screening evaluations unless otherwise specified. In certain circumstances, screening and entry visits may occur sequentially in real time as long as all screening criteria are confirmed prior to entry.
- Entry evaluations must occur after randomization and be completed before initiating study treatment.
- Subjects must begin treatment within 72 hours after randomization.

6.2.3 Post-Entry Evaluations

On-Study Evaluations
All on-study evaluations must be scheduled as per section 6.1 with a ± 7 day window for the month 1 visit and a ± 14 day window for all other visits.

Subjects who miss visits should be contacted to make up the visit and to permit determination of whether a major event occurred.

Return Fasting Visit (as needed)
This visit is only required for subjects who are in a non-fasting state for visits that require fasted assessments. These subjects should come back into the clinic within 7 days for a fasting evaluation.

Study Termination Evaluations
The study termination evaluations will be completed as the subject's final on-study visit.

Event-Driven Evaluations
Evaluations must be scheduled as per section 7.0.

6.2.4 Discontinuation Evaluations

Evaluations for Registered or Randomized Subjects Who Do Not Start Study Treatment
Subjects who withdraw from the study before initiating study treatment will follow the clinical assessment and laboratory schedules as per section 6.1. Every attempt will be

made to follow such subjects particularly with respect to assessment of the primary endpoint.

Premature Treatment Discontinuation Evaluations
Subjects who discontinue the study treatment before the end of the study will have the premature treatment discontinuation evaluations completed within 14 days after stopping the study drug. At this visit, study drug will not be dispensed but pill counts will be performed for these individuals. After completion of the premature treatment discontinuation evaluations and until the study termination visit, subjects will follow the clinical assessment and laboratory schedules as per section 6.1. Medication adherence by questionnaire will be assessed. More frequent clinical and laboratory evaluations may be clinically indicated.

Premature Study Discontinuation Evaluations
Subjects who prematurely discontinue from the study will have the premature study discontinuation evaluations performed as per section 6.1 prior to being taken off study.

NOTE: Sites will capture and document reasons for withdrawal of consent, eg, related to AE and whether withdrawal of consent pertains to consent for follow up and/or treatment.

In the event that a participant cannot be reached and is considered lost to follow-up, sites will attempt to obtain information regarding vital status (including date last seen alive, date of death and primary cause of death) from other sources (such as family members, other designated contacts or clinic records).

6.3 Instructions for Evaluations and Data Collection

All clinical and laboratory information required by this protocol is required to be maintained in the source documents. Sites must refer to the Source Document Guidelines on the DAIDS Web site for information about what must be included in the source document:
http://www.niaid.nih.gov/labsandresources/resources/daidsclinrsrch/documents/sourcedocappndx.pdf

All stated evaluations are to be recorded on the CRF and keyed into the database unless otherwise specified. This includes events that meet the International Conference on Harmonization (ICH) definitions for a serious adverse event.

- Results in death
- Life-threatening
- Requires inpatient hospitalization or prolongation of existing hospitalization
- Results in persistent or significant disability/incapacity
- Congenital anomaly/birth defect
- Other important medical event (may not be immediately life-threatening or result in death or hospitalization but may jeopardize the patient or may require intervention to prevent one of the events listed above).

To grade diagnoses, signs and symptoms, and laboratory results, sites must refer to the DAIDS Table for Grading the Severity of Adult and Pediatric Adverse Events (DAIDS AE Grading Table), Version 2.0, November 2014, which can be found on the DAIDS RSC Web site: http://rsc.tech-res.com/safetyandpharmacovigilance/gradingtables.aspx.

6.3.1 Documentation of HIV-1

Section 4.1.1 specifies assay requirements for HIV-1 documentation. HIV-1 documentation is not recorded on the CRF.

6.3.2 Medical History

The following diagnoses should be recorded at screening and updated at entry, regardless of when the diagnosis was made:

- Hypertension
- Diabetes mellitus
- AIDS-defining events
- Any malignancy (exclusive of basal/squamous cell skin cancer)
- Prior history of dialysis or renal transplantation
- Chronic active hepatitis C
- Chronic active hepatitis B
- Nadir CD4 cell count (verbal history accepted)

Any allergies to any medications and their formulations must also be documented.

6.3.3 Medication History

A limited medication history must be performed at screening and updated at entry and post entry visits as per the medication history table below. Record all modifications to ART, including subject-initiated modifications (more than 7 consecutive missed days), provider-initiated modifications, and permanent discontinuation. See Table and MOPS for more details.

Medication History Table

Medication Category	Complete History or Timeframe	Record on CRF (Yes/No)
Antiretroviral therapy	Cumulative duration of PI, thymidine analogs, abacavir, tenofovir, and overall ART duration	Yes
Current ART therapy	Current	Yes
Blinded study therapy (other than for REPRIEVE)	Current	Yes
Statin therapy	Any prior or current exposure	Yes
Nonstatin lipid lowering therapy	Current	Yes
Antidiabetic medications	Current	Yes
Aspirin therapy (ongoing regular therapy) and anticoagulant medications	Current	Yes
Antihypertensive medications	Current	Yes
Antihepatitis medications	Current	Yes
Hormonal contraceptives or hormone replacement therapy	Current	Yes
Testosterone therapy	Current	Yes

Study Treatment Modifications
The study drugs are pitavastatin and placebo for pitavastatin. Record all study drug modifications, subject-initiated and protocol-mandated modifications, inadvertent and deliberate interruptions of more than 7 consecutive days. Record any permanent discontinuation of treatment.

6.3.4 Clinical Assessments

Signs and Symptoms
At entry, all grades that occurred 30 days before entry must be recorded; post-entry, only signs and symptoms Grade ≥3 must be recorded. Record all signs and symptoms that led to a change in treatment (pitavastatin or placebo) regardless of grade. Further evaluation will be required for those events that meet EAE or ICH reporting requirements.

Diagnoses
All incident diagnoses listed in section 6.3.2 will be assessed at all visits post entry; refer to section 6.4 for requirements for intervening medical history review related to primary MACE and other secondary serious non-cardiovascular events.

Assessment of Potential Myalgia Toxicity
Potential statin effects on the liver will be assessed with ALT at 1 month and 12 month visits; potential effects on muscle will be assessed by myalgia symptom questionnaire at entry and onward.

Cardiovascular Risk Assessment Tool
The ACC/AHA 2013 10 year ASCVD risk score should be performed as part of screening. The calculator is located at the following website: http://my.americanheart.org/professional/StatementsGuidelines/PreventionGuidelines/PreventionGuidelines_UCM_457698_SubHomePage.jsp.

NOTE: For the purposes of calculation of the 10-year ASCVD risk score Using the Pooled Cohort Equations

- For subjects whose values of HDL cholesterol, total cholesterol, and/or systolic blood pressure fall below or above the acceptable calculator bounds for those parameters, values at the lower or upper bounds, respectively, will be entered
- Subjects of mixed race will be asked to identify themselves as predominantly African American or predominantly other and the race of predominant identification will be entered; as per calculator guidelines, non-African American race is entered as White or other race
- Subjects will be asked to report sex at birth and this sex will be entered
- *Only* subjects currently on 1 or more antihypertensive medications will be counted as undergoing treatment for high blood pressure.
- *Only* subjects who report current active smoking will be counted as smokers.

Cardiovascular Risk Factor Assessment
An assessment of general cardiovascular risk factors, current smoking, alcohol use, substance use, family history of premature CVD, hormonal history will be assessed at screen. Diet (Rapid Eating and Activity Assessment for Patients [(REAP] questionnaire), and functional capacity (Duke Activity Status Index [DASI] questionnaire) will be completed at entry and final visits.

Screening Physical Exam
A screening physical examination is to include auscultation of the chest; cardiac exam; and examination of the lower extremities for edema. The screening physical exam will also include vital signs.

Targeted Physical Exam
A targeted physical examination is to include vital signs, and is to be driven by any previously identified or new signs or symptoms, including muscle aches, pains, tenderness, weakness, malaise, or fever. A targeted physical exam will also be driven by any diagnoses that the subject has experienced within the 30 days prior to the last visit. The targeted physical exam will occur at month 1 and each annual visit.

Height
Measurement of height will occur at screening.

Weight
Measurement of weight will occur at screening and each annual visit.

Waist Circumference
Measurement of iliac waist circumference will occur at screening and final visit. Please see MOPS for instructions on waist circumference measurement.

ECG
Resting 12-lead ECG results, including heart rate will be performed at entry. Please see MOPS for instructions.

6.3.5 Lifestyle Information

Subjects will be provided information regarding healthy diet and activities in the Lipid-Lowering Diet, Activity Guide, and Smoking Cessation located on the REPRIEVE (A5332) PSWP. In addition, subjects will be reminded to adhere to prescribed antiretroviral regimen and study medication. This is to be provided at entry and annual visits.

6.3.6 Laboratory Evaluations

At screening and entry, all laboratory values must be recorded. For post-entry assessments, record all Grade ≥3 laboratory values. All laboratory values that led to a change in treatment or that are performed as part of toxicity management (for example all CK assessments performed as part of management of myalgias and myopathy), must be recorded regardless of grade. Further evaluation will be required for those events that meet EAE or ICH reporting requirements. Subjects must be evaluated in a fasting state for those evaluations indicated as “fasting.” For screening labs captured from clinical care, these should be within 90 days of start of study unless otherwise indicated. Laboratory testing must be performed in a CLIA or equivalent certified laboratory.

NOTE: When labs are being captured from clinical care, the most recent lab value should be recorded.

CD4+/CD8+

- Screen: Absolute CD4+/CD8+ count and percentages will be obtained from standard of care within 180 days prior to entry as part of study at a CLIA-certified or equivalent laboratory.
- Post-entry evaluations: only a CD4+/CD8+ obtained since the last study visit may be used.

NOTE: If CD8+ cell count is not available, CD4+ cell count is acceptable.

Plasma HIV-1 RNA

At all visits indicated on the Schedule of Events, record on the CRF the results of plasma HIV-1 RNA determinations that are obtained through routine clinical care at a CLIA-certified or equivalent laboratory. For the entry visit, a viral load from the prior 180 days should be recorded. For all post-entry evaluations, the most recent viral load obtained since the last study visit should be recorded.

Hematology

Hemoglobin, hematocrit, white blood cell count (WBC) and platelets will be performed as part of the study at screening and end of study visit if not available as part of routine clinical care. At screening, results must be within 90 days prior to entry. At end of study visit, results must be within 90 days prior to that visit.

Serum creatinine and calculated creatinine clearance as estimated by the Cockcroft-Gault equation: at screening, creatinine result will be obtained from standard care within 90 days prior to entry. At end of study visit, results must be obtained within 90 days prior to that visit.

NOTE: If results are not available they may be drawn as part of the study.

NOTE: Please refer to the MOPS for the calculator for the Cockcroft-Gault equation.

Fasting Lipid Panel

Total cholesterol, HDL cholesterol, calculated LDL cholesterol (if triglycerides <400 mg/dL) or direct LDL if triglycerides are >400 mg/dL and <500 mg/dL, and triglycerides will be performed at the local laboratory at screening; specimens from entry onward will be stored to be tested centrally and results will be blinded until the end of the study. The screening lipid panel will be reviewed with the potential subject and shared with his or her medical provider along with the study rationale to confirm support of subject participation.

NOTE: Fasting is defined as nothing to eat or drink except water and required prescription medications for at least 8 hours. Drinking black decaffeinated coffee without sweetener or creamer is permissible, but is not advised. Subjects will be asked whether they have fasted, and if not, they should be scheduled to return in a fasting state within 7 days to complete the fasting evaluations.

Subjects should be instructed with the exact time beyond which they are to be fasting, such as: “Your visit is scheduled for 8:00 a.m. You should not have any food or drink by mouth except water and medication after 12 a.m.”

In order to minimize diurnal variation, fasting samples for individual subjects should be obtained consistently in the morning, if possible. Subjects will be encouraged to take the study drug consistently at the same time and this information will be collected in the medication adherence questionnaire.

Liver Function Tests
ALT (SGPT) will be performed as part of the study at screening, month 1, and month 12. Persons with HCV or HBV should also have AST (SGOT) done at screening to facilitate calculation of fibrosis by the FIB-4 equation.

NOTE: Refer to MOPS for link to the FIB-4 calculator.

Pregnancy Testing
For women with reproductive potential: serum or urine β-HCG (urine test must have a sensitivity of 15-25 mIU/mL) will be performed as part of the study at each visit and whenever pregnancy is suspected. Refer to the MOPS for guidance on recording pregnancy outcomes.

Fasting glucose at entry and onward will be performed as part of the study. Specimens will be tested centrally and results will be blinded until the end of the study.

NOTE: Fasting is defined as above (see 6.3.6 for Fasting Lipid Panel).

Urine Albumin/Creatinine Ratio
Urine will be collected for urine albumin/creatinine ratio as part of the study at entry. Specimens will be batched and tested centrally.

6.3.7 Immunologic and Biomarker Studies

Plasma/Serum for Biomarkers - Stored
Additional serum and plasma will be batched and stored in the ACTG Specimen Repository for analyses by a central core laboratory. Refer to the Laboratory Processing Chart (LPC) for details.

6.3.8 Stored Whole Blood for Future Genetic Studies

A single whole blood sample will be obtained from all study volunteers for human genotyping of selected polymorphisms that may predispose to CVD or alter pitavastatin levels or effectiveness. This sample will be drawn at ACTG sites only and will be collected in addition to any samples collected for subjects in A5128 or A5243.

6.3.9 Medication Adherence Assessment

Site personnel will perform an assessment of adherence to study medication, including a pill count, at every visit after the entry visit.

6.3.10 Vital Status Follow-up

Site personnel will attempt to obtain information regarding vital status (including date last seen alive, date of death and primary cause of death) from other sources (such as family members, other designated contacts, or clinic records).

6.4 Endpoint Assessments

At entry and onward, a comprehensive review of the intervening medical history of the subject will be performed to capture the data for adjudication of the primary and secondary endpoints, including diagnoses and any hospitalization. Subjects will be specifically asked about hospitalization, emergency room visits, urgent care visits, physician visits, and any symptoms that are suggestive of cardiovascular disease. Information regarding potential events will be captured on CRFs and via source documents and prepared for transmission after de-identification to the Clinical Event Committee (CEC) for adjudication. Subjects who miss visits will be contacted to make up the visit and to permit determination of whether major event occurred. Events will be reviewed and categorized by a CEC unaware of the treatment assignment based on established definitions described below.

6.4.1 Definitions of Major Adverse Cardiovascular Events (MACE) Components

The primary endpoint of Major Adverse Cardiovascular Events (MACE) includes the composite of Cardiovascular Death, Myocardial Infarction, Hospitalization for Unstable Angina, Coronary, Carotid or Peripheral Arterial Revascularization, Transient Ischemic Attack, Stroke, and Peripheral Arterial Ischemia.

Formal definitions for each of these components and additional secondary and exploratory endpoints are contained within the CEC charter, and are based on the Standardized Definitions for Cardiovascular and Stroke End Point Events in Clinical Trials [Hicks 2012].

General principles guiding these definitions are described below.

<u>CVD death</u>: includes death resulting from an acute myocardial infarction (MI), sudden cardiac death, death due to heart failure (HF), death due to stroke, death due to cardiovascular (CV) procedures, death due to CV hemorrhage, and death due to other CV causes.

<u>Myocardial infarction</u>: The diagnosis of MI requires the combination of: evidence of myocardial necrosis (either changes in cardiac biomarkers or post mortem pathological findings); and supporting information derived from the clinical presentation, electrocardiographic changes, or the results of myocardial or coronary artery imaging suggesting an event consistent with coronary ischemia.

The totality of the clinical, electrocardiographic, and cardiac biomarker information will be considered to determine whether or not an MI has occurred. Specifically, timing and trends in cardiac biomarkers and electrocardiographic information will be included whenever possible, but the diagnosis can still be determined if these results are not available.

<u>Unstable angina hospitalization</u>: ischemic discomfort or equivalent requiring hospitalization within 24 hours with objective signs of coronary ischemia in absence of MI. ECG, angiographic and imaging criteria will be considered.

<u>Coronary, carotid, or peripheral arterial revascularization</u>: invasive percutaneous or

surgical procedure intended to restore or improve blood flow in a coronary or peripheral artery including but not limited to angioplasty, stent, stent graft, or bypass graft.

Stroke: an acute episode of focal or global neurological dysfunction caused by brain, spinal cord, or retinal vascular injury as a result of hemorrhage or infarction.

Transient Ischemic Attack: a transient episode of focal neurological dysfunction caused by brain, spinal cord, or retinal ischemia, without stroke.

Peripheral Arterial Ischemia (PAD): Peripheral arterial ischemia hospitalization: Urgent hospitalization for insufficiency of the peripheral arterial circulation, including but not limited to, acute limb ischemia, chronic limb ischemia, amputation, or other vascular abnormality of an ischemic and noninfectious nature.

6.4.2 Causes of Death

In addition to CVD death which is a component of the primary endpoint, all deaths will be adjudicated by the CEC to determine likely cause.

6.4.3 Cardiac Events to be adjudicated but not included in REPRIEVE (A5332) MACE Definition:

- Heart Failure (see CEC Charter)

6.4.4 Non-CVD events

Clinical assessments will also include assessing the subject regarding the occurrence of the following diagnoses:

- AIDS-defining events
- Non AIDS defining cancers (except squamous/basal cell of the skin)
- End stage kidney disease, requiring initiation of dialysis or renal transplantation
- End stage liver disease (cirrhosis or hepatic decompensation requiring hospitalization)
- Incident diabetes mellitus requiring use of diabetes medications

For diagnostic criteria for these non-CVD events, refer to the MOPS.

7.0 CLINICAL MANAGEMENT ISSUES

Only toxicities related to the study drug (pitavastatin and placebo for pitavastatin) are subject to the guidelines outlined in the toxicity management section. The grading system is located in the DAIDS Table for Grading the Severity of Adult and Pediatric Adverse Events (DAIDS AE Grading Table), Version 2.0, November 2014, which can be found on the DAIDS RSC Web site: http://rsc.tech-res.com/safetyandpharmacovigilance/gradingtables.aspx.
Please refer all questions to the REPRIEVE (A5332) protocol team via email, as described in the Study Management section.

In the case of multiple toxicities or adverse events (AEs), the guidelines pertaining to the most severe event should take precedence.

7.1 Toxicity Management

7.1.1 General Reactions

Grade 1 or 2
Subjects who develop a Grade 1 or 2 toxicity may continue study treatment.

Grade 3
Subjects who develop a Grade 3 toxicity that is judged by the site investigator to be study drug-related should have the study drug held and the study team should be consulted. The subject should be followed closely and if the toxicity does not return to Grade ≤2 within 2 weeks, the study drug must be permanently discontinued with subject evaluations as per section 6.2.4.

If the study drug is resumed and the same Grade 3 toxicity recurs within 4 weeks of reintroduction, and the site investigator considers this AE related to the study drug, the drug must be permanently discontinued.

With a Grade 3 toxicity that is judged not related to the study drug by the site investigator, the study drug may be continued at the discretion of the site investigator in consultation with the study team.

Grade 4
Subjects who develop a Grade 4 toxicity will have the study drug permanently discontinued, with clinical assessments and laboratory testing as described for Grade 3 toxicity. If the investigator feels that the toxicity is clearly related to another cause and that the toxicity is not caused by the study drug, and after consultation with the study team, dosing may continue when the toxicity has resolved to Grade 2 or less.

Subjects experiencing Grade 4 toxicities should be followed closely with additional clinical assessments and laboratory testing as clinically indicated in consultation with the study team.

NOTE: Direct and indirect bilirubin elevations that reach Grade 4 elevations according to the DAIDS toxicity schema and are related to atazanavir are excluded from reporting.

7.1.2 ALT elevations

ALT levels will be routinely evaluated at visits at month 1 and month 12. All other evaluations of ALT will be performed at the discretion of the site investigator based on subject symptoms.

Grade 3
Subjects who develop *asymptomatic* >5 x ULN ALT elevations (Grade 3), study drug should be held for 1 week and the individual should be re-evaluated 1 week after drug discontinuation. If at that time the ALT elevation is ≤5 x ULN and subjects remain asymptomatic, the subjects are eligible to continue on study treatment at the discretion of the site investigator. If the ALT does not return to ≤5 x ULN within the 1 week period, the study drug must be permanently discontinued unless the ALT elevations are deemed not related to study drug upon further assessment as per the discretion of the PCP (ie, acute hepatitis A or other clear causation).

For any *symptomatic* (eg, fatigue, nausea and vomiting, right upper quadrant pain, rash or eosinophilia) ALT >5 x ULN (Grade 3), study drug should be held. Subjects should be asked to return to the research site for repeat testing 1 week later. If repeat ALT is ≤3 x ULN and the subject is no longer symptomatic, study drug can be resumed.

<u>Grade 4</u>
For any ALT >10 x ULN (Grade 4), study drug should be discontinued. The subject should be brought back for repeat testing every 1 week until the ALT ≤5 x ULN.

NOTE: If the Grade 3 or 4 elevation is clearly related to another cause and not related to study drug (eg, acute hepatitis A infection), the subject should be brought back for repeat testing every 2 weeks for Grade 3 or every 1 week for Grade 4 until the ALT ≤3 x ULN. The local site investigators should contact the study team for approval before resuming study drug. Subjects who permanently discontinue study drug will be followed on study, off treatment through the study termination visit with subject evaluations as per section 6.2.4.

NOTE: For those subjects with ALT >3 x ULN upon repeat testing, whether symptomatic or not, AST, alkaline phosphatase, and total bilirubin or INR, should also be performed as part of the study to help determine etiology of increased LFTs. For subjects on atazanavir, performance of INR rather than bilirubin is preferred. Other labs including hepatitis serologies may also be indicated and performed in the context of clinical care by PCP.

NOTE: If the subject has recurrent elevations of ALT >3 x ULN but the site investigator deems the elevation not related to study drug, the site investigator must contact the study team to discuss continuation of study drug.

Abnormal ALT determinations occurring in the course of clinical care should be repeated by the treating clinician. Persistently abnormal values which would trigger the toxicity guidelines above should be reported to the REPRIEVE (A5332) team. If the ALT abnormality is not due to another cause, eg, acute hepatitis, the REPRIEVE (A5332) site PI will follow the toxicity algorithm above.

7.1.3 Myalgias and Myopathy

Persons who present with significant myalgias (Grade ≥3, ie, muscle pain causing inability to perform usual social and functional activities) should be evaluated with a clinical assessment that includes an evaluation of CK, serum creatinine, potassium, and urinalysis. Myopathy is defined as muscle aches, soreness, tenderness, or weakness with creatinine kinase (CK) >10 x ULN not related to exercise or other causes, including trauma. If the symptoms are associated with Grade ≥3 elevation in CK (10 x ULN) (see table below) <u>that is not related to exercise or other cause</u>, study medications should be permanently discontinued. Subjects will be followed on study, off treatment through the study termination visit with subject evaluations as per section 6.2.4.

NOTE: Mitochondrial toxicity related to nucleoside therapy and not related to study medication is a possibility, and evaluations for lactic acidosis should be considered by the subject’s primary care provider.

Serum CK Toxicity Grading*

Toxicity Grade	Value
Grade 1	3 – <6 x ULN
Grade 2	6 – <10 x ULN
Grade 3	10 – <20 x ULN
Grade 4	≥20 x ULN

* Not related to exercise or other cause

7.1.4 Rhabdomyolysis

Rhabdomyolysis is defined as the presence of myopathy as per section 7.1.3 plus one or more of the following:

- Hematuria on urine dipstick in the absence of microscopic hematuria (myoglobinuria)
- Grade ≥2 hyperkalemia
- Grade ≥2 creatinine elevation.

If rhabdomyolysis occurs, study medications should be permanently discontinued. The team should be consulted. Subjects will be followed on study, off treatment through the study termination visit with subject evaluations as per section 6.2.4. In addition, CK will be added to the laboratory evaluations performed until it has declined to ≤1 x ULN.

7.2 Requirement for Precautionary or Prohibited Medications (see PSWP)

Subjects who need to initiate therapy with erythromycin, colchicine, cyclosporine, or rifampin should be asked to hold study drug. If use of one of these precautionary medications is anticipated to be short-term, the site investigator may consider restarting study drug after use of prohibited medication is discontinued.

Subjects who begin a statin medication provided through clinical care should discontinue study drug. Taking two statins can increase the risk of toxicity.

Subjects who temporarily or permanently discontinue study treatment will be followed on study, off treatment through the study termination visit with subject evaluations as per section 6.2.4.

7.3 Pregnancy

If the pregnancy test is positive at entry, then the subject should not start study treatment. No further evaluations are necessary, provided that the subject did not initiate study drug.

Subjects who become pregnant after study entry must discontinue study treatment immediately. These subjects should be seen for a premature treatment discontinuation evaluation within 7 days. Subjects will be followed on study, off treatment through the study termination visit with subject evaluations as per section 6.2.4. The core team must be notified of any pregnancies that occur in subjects on study. Management of the background ART is at the discretion of the site investigator.

All pregnancies should be followed until the final outcome can be determined. In the event that the pregnancy has not been completed by the final study visit, the site should contact the subject through monthly phone calls and review of medical records, if possible, until the

pregnancy outcome can be ascertained. See the MOPS for guidance on documenting pregnancy outcomes.

Pregnancies that occur on study should be reported prospectively to The Antiretroviral Pregnancy Registry. More information is available at www.apregistry.com. Phone: 800-258-4263; Fax: 800-800-1052 (Non-US sites: Fax: 44-1628-789-666 or 910-246-0637; phone: 910-679-1598.)

7.4 Unblinding Procedures

For unblinding requests, including emergency unblinding, refer to the ACTG Unblinding Subjects Standard Operating Procedure (SOP) 123 at https://member.actgnetwork.org/cms/folder/6184.

Please note: unblinding is rarely allowed as study medications can most often be withdrawn in a participant experiencing adverse effects without the need for unblinding. Any decision on unblinding should be made with reference to the ACTG SOP.

In the event that emergency disclosure of treatment assignment is thought to be required, the site investigator must follow the ACTG Unblinding Subjects SOP 123: https://member.actgnetwork.org/cms/folder/6184.

The protocol chairs and DAIDS medical officer will be notified of such a request through the ACTG DMC unblinding program. All site e-mails to the team should be carefully worded to prevent unblinding the team, if possible.

Unblinding of all study subjects will take place after the last subject has completed the study, all data have been entered into the database and cleaned for primary and secondary endpoints, and MACE endpoint verification is complete. The time necessary to finalize the data can be up to 3 months or more after study closure.

8.0 CRITERIA FOR DISCONTINUATION

8.1 Premature and Permanent Treatment Discontinuation

- Drug-related toxicity per section 7.0.
- Clinical reasons believed life threatening by the physician, even if not addressed in section 7.0.
- Pregnancy or breast-feeding.
- Use of prohibited and some precautionary medications per section 7.0. See PSWP for precautionary and prohibited medication list for further information. Please contact the study team if you have questions.

NOTE: Subjects who temporarily or permanently discontinue study treatment will be followed on study, off treatment through the study termination visit with subject evaluations per section 6.2.4.

8.2 Premature Study Discontinuation

- Refusal by the subject of further study follow up.
- Request by the subject to withdraw consent.
- Request of the primary care provider if s/he thinks the study is no longer in the best interest of the subject.
- Subject judged by the investigator to be at significant risk of failing to comply with the provisions of the protocol as to cause harm to self or seriously interfere with the validity of the study results. If a subject misses clinic visits for >1 year, it is at the discretion of the site PI whether or not to discontinue the subject from study participation.
- At the discretion of the ACTG, IRB/EC, Food and Drug Administration (FDA), Office for Human Research Protections (OHRP), NHLBI, NIAID, other government agencies as part of their duties, investigator, or pharmaceutical supporter.

Subjects who prematurely discontinue from the study will have the premature study discontinuation evaluations performed as per section 6.1 and then be taken off study.

In the event that a participant cannot be reached and is considered lost to follow-up, sites will attempt to obtain information regarding vital status from other sources (such as family members, other designated contacts, or clinic records) per section 6.2.4.

9.0 STATISTICAL CONSIDERATIONS

9.1 General Design Issues

REPRIEVE (A5332) is a prospective, randomized, double-blind, placebo-controlled, phase IV study of the effect of pitavastatin on major cardiovascular events in HIV-1 Infected Individuals who do not meet current guidelines for statin therapy. The study will enroll 6500 HIV-infected subjects who are on stable ART and not eligible for statins as per the 2013 ACC/AHA guidelines. Enrollment is anticipated to take 30 months; study follow-up will continue for approximately 72 months after the enrollment of the first subject.

All primary analyses will be performed as intention-to-treat and include all subjects as randomized.

A complete firewall will be maintained to ensure that investigators have no access to the data. All data will be kept on secure systems at Frontier Science and Technology Research Foundation (FSTRF). A complete description of the firewall procedures, data organization, and security will be included in the final statistical analysis plan. The plans and procedures will be consistent with NHLBI and NIAID policy with respect to maintenance of data integrity.

9.2 Outcome Measures

For all time to event study endpoints, time will be measured from the date of randomization to the onset date of the event of interest.

9.2.1 Primary Endpoint

The primary endpoint will be time to the first event of a composite of major cardiovascular events including:

- Atherosclerotic or other CVD death
- Nonfatal myocardial infarction
- Unstable angina hospitalization
- Coronary or peripheral arterial revascularization
- Nonfatal stroke or TIA
- Urgent PAD ischemic event (acute or chronic limb ischemia, amputation, etc.)

All primary events will be prospectively determined and adjudicated by an expert Clinical Events Committee based on standardized criteria used in prior cardiovascular trials and developed by consensus groups and the FDA. [Hicks 2012] (see section 6.4.1).

Subjects lost to follow-up without experiencing the event will be considered censored at the time of their contact at which an assessment for primary endpoints was made; deaths from non CVD causes will be treated as competing risk events in the primary analysis. See section 9.6 for additional supportive analyses.

9.2.2 Supportive and Secondary Endpoints

9.2.2.1 Time to the first of each individual component of the primary endpoint.

For each event, subjects lost to follow-up without experiencing the event will be considered censored; deaths from other causes will be considered competing risk events.

9.2.2.2 Time to death (all-cause mortality)

Based on independent review, death will be classified as cardiovascular event or non-cardiovascular event. Non cardiovascular events will be further characterized as HIV-associated clinical diagnosis, non-AIDS malignancy, accidental, suicide, homicide, other sudden death of unknown etiology, or other. See SAP for further details.

9.2.2.3 Time to death (all-cause mortality) and/or MACE

9.2.2.4 Time to any (composite) or each (individual) of the following clinical diagnoses (including recurrent diagnoses as appropriate)

- Non AIDS-defining cancers (excluding basal cell and squamous cell carcinomas of the skin)
- AIDS-defining events (based on CDC 2014 classification)
- Initiation of dialysis or renal transplantation.
- Cirrhosis, or hepatic decompensation requiring hospitalization

For specific case definitions see MOPS.

For each event, subjects lost to follow-up without experiencing each event will be treated as censored; deaths from other causes will be treated as competing risk events.

9.2.2.5 Calculated fasting LDL and non-HDL cholesterol level at study entry and annually thereafter as well as change from baseline expressed as absolute change and as a percentage of baseline. For subjects with triglycerides >400 mg/dL and <500 mg/dL, direct LDL will be determined and use in the statistical analysis.

9.2.2.6 Time to any of the following adverse events (including recurrent events as appropriate)

- Serious adverse event as defined by ICH criteria
- Incident Diabetes mellitus (DM)
- Grade 3 or 4 ALT
- Grade 3 or 4 myopathy

All events will be included regardless of relationship to treatment as determined by sites.

Grading will be defined per the DAIDS Table for Grading the Severity of Adult and Pediatric Adverse Events, Version 2.0, November 2014. See section 7.0 for link to the document.

9.2.3 Exploratory Endpoints

9.2.3.1 Fasting Total and HDL cholesterol and LDL-C/HDL-C ratio at study entry and annually thereafter.

9.2.3.2 Time to heart failure

9.3 Randomization and Stratification

At study entry, subjects will be assigned with equal probability to one of the two treatment arms. Randomization will use permuted blocks with stratification by sex (male/female) and screening CD4+ T-cell counts (≤500 vs. >500 cells/mm^3) Additionally, to ensure balanced treatment allocation for the Mechanistic Substudy of REPRIEVE (A5333s), randomization will also be stratified by whether or not a subject has elected to participate in the mechanistic substudy (yes/no).

It is anticipated that the study will take 30 months to fully enroll.

9.4 Sample Size and Accrual

The target sample size of REPRIEVE (A5332) is 6500 individuals. Sample size considerations for REPRIEVE (A5332) are provided below.

The sample size for the main study was determined to provide 90% power to detect a 30% reduction in the composite CVD endpoint with statins (statin effectiveness equating to Hazard Ratio (HR) of 0.70). This desired effect size equates to a 5-year number needed to treat of 47. The effect size is more than the 22% based on LDL reduction alone seen in the CTT

collaboration [CTT Collaboration 2010], consistent with the hypothesis of REPRIEVE (A5332), that statins will have an effect beyond LDL lowering. The HR is a more conservative effect size than was observed in JUPITER (a primary CVD prevention trial in non HIV patients) - a HR of 0.56 or 44% reduction in the composite endpoint [Ridker 2008]. This more modest clinical effect is felt justified based on the higher absolute risk of CVD outcomes in the HIV-infected population as well as some anticipated cross-over between the two study groups.

Assuming a fixed sample size of 6500 individuals and total follow-up of 6 years, Table 9.1 shows the power under a range of alternative scenarios. For example, under the baseline assumption of an event rate of 15/1000 PY and accrual duration of 2.5 years, the sample size of 6500 subjects will ensure 90% power to detect a 30% reduction in event rates (HR = 0.70; 5 year NNT = 47). However, with slower accrual (2.75 yrs) and lower event rates (14/1000 PY) REPRIEVE (A5332) will still have adequate power at 89% and 88%, respectively. Moreover, while power will be reduced to 83% in the case of severely reduced event rates of 12/1000 PY and a slower accrual rate of 2.75 years, contingencies to extend REPRIEVE (A5332) for a total follow up of 6.5 years while remaining within the scope of the current budget, will assure a reasonable 85% power.

Table 9.1: Power to detect given hazard ratio (HR) with total sample size of 6500 based on 2.5 years of accrual and 6 years maximal follow-up

Design Assumptions								Power to detect given HR		
Control rate of MACE	5y event rates for given HR				5y NNT for given HR					
	Control	Statin								
		0.65	0.70	0.75	0.65	0.70	0.75	0.65	0.70	0.75
18/1000 PY	8.7%	5.7%	6.1%	6.5%	34	39	48	99%	94%	83%
15/1000 PY	7.3%	4.8%	5.1%	5.5%	41	47	57	97%	90%	76%
12/1000 PY	5.8%	3.8%	4.1%	4.4%	50	58	70	93%	83%	67%

The assumptions and justification underpinning the REPRIEVE (A5332) study sample size are as follows:

A composite CVD event rate of 15/1000 PY in the absence of statin therapy: Event rates for a similar composite endpoint were queried in the Partners Research Patient Data Registry (RPDR) for individuals ≥ 40 years of age, and determined for 3,213 thousand HIV-infected and 26,309 thousand non-HIV patients matched on age/gender/race followed over 10 years from 2000-2009 (14,942 person years for HIV-infected and 106,853 person years for non-HIV). Event rates were 21/1000 PY in the HIV-infected and 15/1000 PY in the non-HIV group. Further refining the Partners database query for incident MI, stroke, angina, revascularization among HIV-infected individuals ≥ 40 years, without diabetes mellitus and no recent history of statin use demonstrated (in alignment with the LDL independent definition of potentially eligible subjects for REPRIEVE (A5332)) a MACE rate of 13/1000 PY. Since CVD death could not be obtained as part of this query, this rate likely underestimates the rate that could be expected in REPRIEVE (A5332). In JUPITER, CVD death represented 20% of all MACE. A similar adjustment would bring the HIV CVD event rate in Partners to 16.2/1000 PY. The placebo rate for MACE in JUPITER, among non HIV-infected patients was 13.6/1000 PY. The assumed event rate of 15/1000 PY for REPRIEVE (A5332) was felt to be a reasonable compromise between these rates.

Enrollment will be completed in 30 months: Given the total study duration of 6 years after enrollment has begun, individual subject follow-up will range from 3.5 to 6 years, with a median follow-up of 4.75 years. REPRIEVE (A5332) will be conducted at approximately 100-130 sites

including the majority of all domestic ACTG sites, and selected non-US ACTG sites as well as protocol specific sites. The assumed enrollment period of 30 months requires an average enrollment rate of 2.2 participants per site per month. Given a staggered rate of site activation, actual site enrollment will need to be between 3 to 4 participants per site per month. This enrollment rate is consistent with enrollment rates observed in recent large ACTG trials and with the expectations of the study sites based on a site survey. Further, a site survey conducted in early 2014 has suggested broad availability of the target study population. As of May 29, 2014, of sites queried, a total of 153 sites have expressed an interest in participating in REPRIEVE (A5332) and estimated being able to enroll 10,551 out of an estimated pool of 46,395 eligible subjects.

An annual 5% loss to follow up rate: Since 1999, the ACTG has actively followed a large cohort of individuals on a limited visit schedule. During that time, the annual rate of lost to follow up was observed to be around 5.6%. We believe that we will be able to achieve a rate lower than this in REPRIEVE (A5332) since participants may also be receiving active treatment. Indeed, while this conservatively high estimate maintains power of study even in the event of 25% rate of loss over the duration of the study, it is desired that the observed rate will be no more than 15% over 6 years.

Estimated treatment cross-over rates of 10%: REPRIEVE (A5332) is powered to detect a statin effectiveness equating to a HR of 0.70. The influence of treatment switching (crossover) on the effectiveness of statin treatment for the prevention of MACE and impact of this crossover on the power of the REPRIEVE (A5332) study to detect a statin benefit as currently designed was broadly assessed via a simulation study. Briefly, clinical trials were simulated according to the REPRIEVE (A5332) design and analysis considerations. Simulated MACE times were accelerated and decelerated for discontinuation or initiation of statin treatment (for active and placebo groups respectively) over a range of crossover rates that were varied according to underlying MACE risk – simulated cases in the placebo group of higher underlying CVD risk as well as those in the statin group with the lowest CVD risk were assumed to have the highest rate of crossover. Average statin effectiveness over 6 years was estimated based on uncensored follow-up over >3,000,000 simulated cases. Under the base case, an overall rate of crossover of 10% resulted in an estimated statin effectiveness at the target of 0.70 for an underlying statin efficacy of 0.66. For the same statin efficacy, a 16% overall rate of crossover was associated with an estimated statin effectiveness of 0.74.

9.4.1 Power Considerations for non-MACE Clinical Endpoints

Since the composite rate of serious non-MACE of interest (see section 9.2.2.4) in the absence of statin is expected to exceed the rate of MACE, REPRIEVE (A5332) will be well powered to detect statin effectiveness equating to a 25% reduction in non-MACE or higher.

If the incidence of any specific class of non-MACE in the absence of statin therapy is 5/1000 PY or higher, REPRIEVE (A5332) will have 90% power to detect a statin effect equating to a 50% reduction in the non-MACE event rate or higher. Published data from the ALLRT cohort [Overton 2013] suggest that the incidence of each of these events of interest will exceed this 5/1000 PY threshold.

9.5 Monitoring

The following is a summary of the data and safety monitoring plan for REPRIEVE (A5332). Prior to enrollment of the first subject, a detailed study monitoring and analysis plan document will be prepared that will more fully describe these data monitoring aspects including timelines and responsibilities for preparation.

Summaries of accrual rates, deaths, SAEs, and targeted AEs across regimens as well as study conduct (in terms of off-study rates, and completeness of study visits) will be reviewed on a regular basis by the protocol core team with all data pooled across study arms. Further, in line with the NHLBI Accrual Guidelines of observed against target accrual benchmarks will be reviewed by NHLBI program staff at a minimum of 25%, 50%, and 75% of the anticipated accrual period. Additional accrual reporting to NHLBI will be provided as requested. The timing and anticipated enrollment by 25%, 50%, and 75% of the accrual period are shown in Table 9.2.

Table 9.2: Accrual benchmarks at 25%, 50%, and 75% of the accrual period

Recruitment Period	Time after first patient enrollment	Projected Accrual
25%	7.5 months	767
50%	15 months	2740
75%	22.5 months	4635

The study will undergo at least annual review by an NIH appointed Data Safety Monitoring Board (DSMB) for study conduct, continued feasibility, safety, and efficacy.

Unless otherwise noted, the unblinded REPRIEVE (A5332) statisticians at the ACTG Statistics and Data Analysis Center (SDAC) will be responsible for all data analysis and report preparation to the DSMB. The DSMB will be appointed by NHLBI in consultation with DAIDS. For each review the statisticians will prepare 3 summary reports: a closed report containing all information broken down by masked treatment group distributed only to the DSMB; a report for REPRIEVE (A5332) PIs and NIH (NHLBI and DAIDS) Medical Officers that will include study conduct and safety information pooled over both treatment groups; and an open administrative report with pooled administrative information pooled over treatment groups. Specific contents of reports will be discussed with the DSMB prior to the first review. An outline of the focus of each review is provided in Table 9.3 below; further details and rationale are provide in the text below. These details are provided in broader detail in the Statistical Analysis Plan.

Table 9.3: Overview of DSMB Monitoring Focus

Focus		Timeframe
Feasibility and conduct	Site activation, enrollment, data and visit completeness, rates of loss to follow-up and cross-over	At all interim reviews occurring at least annually
Safety	Rates of adverse events by treatment group	At all interim reviews occurring at least annually
Event rate evaluation	Pooled rates of events observed to date; predicted confidence interval of the pooled event rate under a range of realistic scenarios*	At all interim reviews occurring at least annually

Focus		Timeframe
Formal efficacy and futility review	Treatment group comparison for the primary endpoint utilizing group sequential methods	At least annually starting once the adequacy of the sample size has been established. A total of 4 interim looks for efficacy are planned.
** To be conducted if the pooled rate falls below a specified target (see Statistical Analysis Plan).*		

The first feasibility review will occur approximately one year after the accrual of the first subject with a focus on site activation and patient accrual and retention as well as rates of treatment crossover. Benchmarks for these aspects are provided in detail in the Statistical Analysis Plan that will be reviewed and agreed upon by the DSMB.

In addition to these aspects, pooled rates of events observed to date will be reviewed by the DSMB at all reviews to evaluate the adequacy of the sample size assumptions. Given the expected rate of accumulation of events it is anticipated that sufficient events will have been accrued to the study by the time of reviews occurring 2-2.5 years after enrollment of the first subject to allow reasonable determination of whether the underlying rate of event accumulation is inconsistent with observing the required total number of events to achieve 90% power to detect a HR of 0.70 at the study conclusion. This is illustrated by the anticipated rate of event accumulation dependent on the interim review timing shown in Table 9.4.

Table 9.4: Anticipated data and event accumulation dependent of interim review timing

Timing of review (y)	Number of subjects enrolled*	Accumulated person years of follow-up*	Expected total accumulation of events under given event rate in control group (all cases assume the target HR of 0.7)		
			15/1000	12/1000	10/1000
1	508	127	2	1	1
1.5	1944	972	12	10	7
2	3609	2707	34	28	21
2.5	5414	5685	72	58	43
3	6500	8125	103	82	62
3.5	6500	11375	143	115	86
**Assumes data freeze 4 months prior to DSMB review and enrollment benchmarks agreed with NHLBI.*					

The <u>event rate evaluation</u> will be performed by a blinded REPRIEVE (A5332) statistician at the ACTG Statistics and Data Analysis Center. In the event that the observed number of events falls short of the predetermined benchmarks providing in the statistical analysis plan, a predicted confidence interval analysis will be performed for the total expected number of MACE at trial conclusion based on accumulated data to date and a range of scenarios for accumulation of future data. These will include but not be limited to continued accumulation as observed and, under the target effect size of HR = 0.70, control MACE rate of 12/1000 PY and 15/1000 PY. Unless otherwise requested by the DSMB, these assessments along with recommendations for any study design changes will be presented only in the closed study report, and thus available only to members of the DSMB. Release of the information to the REPRIEVE (A5332) Executive Committee will be at the discretion of the DSMB.

If any of these assessments suggest that the anticipated total number of events appear substantially smaller than the rate assumed for sample size considerations or if accrual is below anticipated, consideration will be given to modifying the design of the study. Such considerations may include (but will not be limited to) extending the study duration,

increasing the target sample size, and broadening the study entry criteria to include individuals with low/moderate traditional CVD risk who are willing to be randomized to statin therapy or placebo, for example subjects who have an ASCVD risk score of ≥7.5%.

Unless there are emerging feasibility or safety concerns, guidelines for stopping or modifying the trial will be guided by formal efficacy review to occur once sample size adequacy for the study has been established. Subsequent to this time, these reviews will occur annually utilizing Lan and DeMet's implementation of the O'Brien-Fleming sequential stopping boundary with information measured on the cumulative number of confirmed composite MACE endpoints. This implementation permits early stopping only for very strong positive or negative effects and maintains most of the nominal power for final analysis; the ultimate recommendation of all reviews – irrespective of whether boundary p-values have been achieved - will be at the discretion of the DSMB. Four interim looks at the data are planned, tentatively occurring at 20%, 40%, 60%, and 80% information.

9.6 Analyses

9.6.1 General Analysis Considerations

The following sections provide a brief overview of the analysis considerations for REPRIEVE (A5332). Prior to the start of enrollment, a detailed Statistical Analysis Plan will be prepared that fully delineates all planned statistical analyses. This plan will be finalized prior to the first interim review of the study data.

All major treatment comparisons between the randomized groups will be performed according to the principle of "intention-to-treat;" that is, subjects will be analyzed (and endpoints attributed) according to the randomized treatment assigned regardless of subsequent changes to that treatment; as-treated analyses will also be performed. Descriptive summaries of the distribution of continuous baseline variables will be presented in terms of percentiles (eg, median, 25th and 75th percentiles), while discrete variables will be summarized in terms of frequencies and percentages. Statistical comparisons will be performed using two-sided significance tests with a 5% Type I error.

Unless otherwise noted, comparison of time to event outcomes will use methods for competing risks, notably Cox proportional hazards models for estimation of cause-specific hazard ratios and Gray's test for comparison of cumulative incidence curves. Treatment group comparisons of continuous outcomes will use t-tests with transformation as needed. In the event of non-Normal distributions even after transformation, Wilcoxon rank sum tests will be preferred. With respect to discrete outcomes, Wilcoxon rank sum tests with be used for ordinal outcomes; Chi-squared test will be used otherwise; Fisher's exact test will be preferred in the event of small cell numbers. All testing will be stratified by sex and CD4 cell count per randomization: while the primary analyses will not be further stratified by enrollment in the Mechanistic Substudy of REPRIEVE (A5333s), sensitivity analyses will be performed including this stratification. Additional perspective regarding the interpretation of the data will be provided through extensive use of confidence intervals and graphical displays.

Given the strong plans for subject follow-up as part of the study, it is anticipated that missing data will be minimized. Unless examination of the data suggest otherwise, missing data will be assumed to be ignorable; subjects lost to follow-up before experiencing a prior endpoint will be considered non-informatively censored. For all

subjects lost to follow-up, cardiovascular risk factors at the time of their final study visit will be described.

With the primary clinical and mechanistic hypotheses and the various secondary endpoints that have been outlined, it is recognized that there is a multiplicity of analyses to be performed, which leads to an increased probability that at least one of the comparisons could be "significant" by chance. Although the overall level of significance for all treatment comparisons will be 0.05, we will be conservative in the interpretation of our supporting analyses, taking into account the degree of significance, and looking for consistency across endpoints.

Assessment will be made for any differences in major disease indices between participants in the Mechanistic Substudy of REPRIEVE (A5333s) and REPRIEVE (A5332). Such differences are unlikely, given that all patients in the Mechanistic Substudy will be eligible for REPRIEVE (A5332) and vice versa. Moreover, a subset of sites will be performing both and there will be overlap in the capacity to do both studies at selected sites. Nonetheless, we will analyze for any differences in the main and Mechanistic Substudy populations and account for any such differences in our interpretation of the Mechanistic Substudy and main study results.

9.6.2 Primary and Supportive Analyses

The primary comparison of study arms for the primary composite endpoint will be time to event analyses and therefore based on the time from randomization to the first of any of the components of the primary composite endpoint. Deaths from non-CVD causes will be treated as competing risk events and participants completing follow-up without experiencing the event will be considered censored at the time of their last contact at which an assessment for primary endpoints was made.

A Stratified Cox proportional hazards model will be the primary analytic methods used for assessing outcome differences between the two treatment groups with stratification by sex and CD4 cell count at screening as previously noted. The relative cause specific hazard of pitavastatin versus placebo for MACE will be estimated with a 95% confidence interval and compared via a Wald test; modification of the statin effect over time (non-proportional hazards) will be evaluated with treatment by time interaction. In supportive analyses, the cumulative incidence of MACE will be estimated over time by treatment group and compared via a stratified Gray test.

To complement the primary analyses, the same analytic approach will be used for evaluation of individual components of the primary MACE endpoint. In the absence of a competing risk event, treatment comparisons of all-cause mortality and a composite of MACE and all-cause mortality will use a stratified log-rank test.

Poisson regression with robust variance estimates will also be used to incorporate multiple and repeated events in evaluation of event incidence rates by treatment group and rate ratios. Sensitivity analyses will be performed that censor individuals for whom critically significant CAD was identified as a result of the Mechanistic Substudy of REPRIEVE (A5333s) CCTA evaluation; censoring will occur at the date of the apparent CCTA study findings.

9.6.3 Secondary Analyses

Analyses of targeted serious clinical diagnoses (see section 9.2.2.4) will use the same methods as described for the primary MACE endpoint for the composite and individual outcomes. Analyses of a further composite outcome including the primary MACE outcomes (the START endpoint) will also be performed.

Summary statistics (means and quantile distributions) of the distributions of LDL and non-HDL cholesterol from study entry and 12 month intervals over time will be estimated with the mean difference in levels between treatment groups 12, 24, 36, 48, and 60 months estimated with 95% confidence intervals and compared at each week using Students t-tests stratified for sex and screening CD4 cell count; the same analyses will be applied for other lipid fractions.

Prognostic Factors of MACE: Important secondary aims of REPRIEVE (A5332) are to evaluate whether baseline traditional risk factors and time updated HIV-specific risk factors are predictive of MACE and pitavastatin effects on MACE in the HIV population.

Targeted risk factors of interest at (or prior to) study entry and time-updated (as indicated) are as follows:

<u>At study entry/screening</u>:

- Age, sex, race, ethnicity
- HIV-1 RNA level
- CD4 cells count
- Nadir CD4 cell count
- Duration of ART exposure and any exposure to thymidine analogs, protease inhibitors, or abacavir
- Weight, BMI, waist circumference
- Fasting lipid (TC, HDL-C and LDL-C, HDL:LDL ratio, TG) and glucose
- Smoking status
- Systolic and diastolic BP and use of anti-hypertensive agents
- Presence of metabolic syndrome defined according to current guidelines at the time of the analysis.
- Self-reported level of physical activity
- Family history of heart disease

<u>Time-updated (annually unless otherwise noted)</u>:

- HIV-1 RNA level
- CD4 cells count
- Fasting lipid (TC, HDL-C and LDL-C, HDL:LDL ratio, TG) and glucose and their changes expressed as absolute change and as percent of baseline

Analyses will use stratified Cox proportional hazards models to estimate the cause-specific hazard of MACE with respect to the risk factors of interest, including the selected biomarkers representing surrogates from plaque progression as described above. In the full cohort we will include the baseline risk factors and evaluate modification of the statin effect by key subgroups (ie, race/ethnicity sex, and CD4 cell count at screening as well as HIV-related and CV risk factors including age, hypertension, LDL and non-HDL cholesterol at entry, BMI, metabolic syndrome (as

defined by current NCEP guidelines at the time of the analysis) and smoking) by interaction terms in these models.

This approach will investigate associations with LDL and non-HDL levels and changes from baseline as a time-updated covariate. The same approach to analysis will be used to assess the effects of other longitudinal outcomes. With respect to HIV-1 RNA levels over time, it is of particular interest to examine whether there is evidence of the modification of the effect of statins according to whether subjects maintain full suppression of HIV-1 RNA levels. This will be examined in a 12-month landmarked Cox proportional hazards models assessing an interaction between continued HIV-1 RNA suppression (<200 copies/mL) over the first 12 months of the study as well as time-updated analysis. The analysis approach for assessment of effect of selected biomarkers for MACE will be determined based on the sampling approach to biomarker testing. Depending on power consideration, sampling may involve testing of the entire REPRIEVE (A5332) study population or a restricted sampling approach such as a case-cohort sampling. In this case, the same analytic approach as described above would be utilized with appropriate weighting for the sampling fractions; an alternative would be case-control sampling with analyses performed using logistic regression.

The frequency with which serious adverse events (excluding study defined clinical endpoints) occur will be tabulated and descriptively summarized. Additional targeted and pre-specified adverse events that will be summarized will include incident diabetes, elevated liver function tests, and myositis. Statistical comparisons of the randomized arms with respect to adverse events will use chi-square or other appropriate two-sample methods depending on the nature of the event, interpreting such comparisons in the context of differences between the two randomized arms in the primary and major secondary clinical endpoints.

10.0 PHARMACOLOGY PLAN

Not applicable.

11.0 DATA COLLECTION AND MONITORING AND ADVERSE EVENT REPORTING

11.1 Records to Be Kept

Case report forms (CRF) will be provided for each subject. Subjects must not be identified by name on any CRFs. Subjects will be identified by the patient identification number (PID) and study identification number (SID) provided by the ACTG DMC upon randomization.

11.2 Role of Data Management

- Instructions concerning the recording of study data on CRFs will be provided by the ACTG DMC. Each CRS is responsible for keying the data in a timely fashion.
- It is the responsibility of the ACTG DMC to assure the quality of computerized data for each ACTG study. This role extends from protocol development to generation of the final study databases.

11.3 Clinical Site Monitoring and Record Availability

- Site monitors under contract to the NIAID will visit participating clinical research sites to review the individual subject records, including consent forms, CRFs, supporting data, laboratory specimen records, and medical records (physicians' progress notes, nurses' notes, individuals' hospital charts), to ensure protection of study subjects, adherence with the protocol, and accuracy and completeness of records. The monitors also will inspect sites' regulatory files to ensure that regulatory requirements are being followed and sites' pharmacies to review product storage and management.
- The site investigator will make study documents (eg, consent forms, drug distribution forms, CRFs) and pertinent hospital or clinic records readily available for inspection by the local IRB/EC, the site monitors, the FDA, the NIAID, the OHRP, and the industry supporter or designee for confirmation of the study data.

11.4 Expedited Adverse Event Reporting to DAIDS

11.4.1 Adverse Event Reporting to DAIDS

- Requirements, definitions, and methods for expedited reporting of AEs are outlined in Version 2.0 of the DAIDS EAE Manual, which is available on the RSC website at http://rsc.tech-res.com/safetyandpharmacovigilance/.
- The DAIDS Adverse Events Reporting System (DAERS), an internet-based reporting system, must be used for expedited AE reporting to DAIDS. In the event of system outages or technical difficulties, expedited AEs may be submitted via the DAIDS EAE Form. For questions about DAERS, please contact DAIDS-ES at DAIDS-ESSupport@niaid.nih.gov. Site queries may also be sent from within the DAERS application itself.
- Sites where DAERS has not been implemented will submit expedited AEs by documenting the information on the current DAIDS EAE Form. This form is available on the RSC website: http://rsc.tech-res.com/safetyandpharmacovigilance/. For questions about EAE reporting, please contact the RSC (DAIDSRSCSafetyOffice@tech-res.com).

11.4.2 Reporting Requirements for this Study

The SAE Reporting Category, as defined in Version 2.0 of the DAIDS EAE Manual, will be used for this study with certain exceptions as noted below. Unless the site investigator considers the following events to be study drug-related, DO NOT REPORT them as EAEs:

- AIDS-defining events (see MOPS for a listing)
- REPRIEVE MACE (see section 9.2.1 for complete listing) and related CVD events: CVD death, MI, stroke, TIA, unstable angina, peripheral ischemia, coronary or peripheral reperfusion procedures, heart failure (because they are components of the primary endpoint or additional adjudicated CVD endpoint). See MOPS for details about reporting CVD Endpoints).

The study agents for which expedited reporting are required are pitavastatin and placebo for pitavastatin.

11.4.3 Grading Severity of Events

The Division of AIDS Table for Grading the Severity of Adult and Pediatric Adverse Events (DAIDS AE Grading Table), Version 2.0, November 2014, must be used and is available on the DAIDS RSC Web site at http://rsc.tech-res.com/safetyandpharmacovigilance/gradingtables.aspx.

11.4.4 Expedited AE Reporting Period

The expedited AE reporting period for this study is the entire study duration for an individual subject (from study enrollment until study completion or discontinuation of the subject from study participation for any reason).

After the protocol-defined AE reporting period, unless otherwise noted, only SUSARs (suspected unexpected serious adverse reactions) as defined in Version 2.0 of the EAE Manual, will be reported to DAIDS if the study staff become aware of the events on a passive basis (from publicly available information).

12.0 HUMAN SUBJECTS

12.1 Institutional Review Board (IRB) Review and Informed Consent

This protocol and the informed consent document (Appendix I) and any subsequent modifications will be reviewed and approved by the IRB/EC responsible for oversight of the study. A signed consent form will be obtained from the subject (or legal representative). The consent form will describe the purpose of the study, the procedures to be followed, and the risks and benefits of participation. A copy of the consent form will be given to the subject or legal representative, and this fact will be documented in the subject's record. Risks, including potential risks of pitavastatin, and protection against risk are described in the accompanying sample informed consent form.

12.2 Subject Confidentiality

All laboratory specimens, evaluation forms, reports, and other records that leave the site will be identified by coded number only to maintain subject confidentiality. All records will be kept locked. All computer entry and networking programs will be done with coded numbers only. Clinical information will not be released without written permission of the subject, except as necessary for monitoring by the ACTG, IRB/EC, FDA, NHLBI, NIAID, OHRP, and other government agencies as part of their duties, or the industry supporter or designee.

12.3 Study Discontinuation

The study may be discontinued at any time by the ACTG, IRB/EC, FDA, NIAID, NHLBI, OHRP, or the industry supporter, or other government agencies as part of their duties to ensure that research subjects are protected.

12.4 Women and Minorities

REPRIEVE (A5332) will aim to recruit women and minority subjects commensurate with the population demographic for HIV in the US. The prevalence of HIV among women in the US is

approximately 22%, while over half of people living with HIV in the US are minorities (CDC 2014).

13.0 PUBLICATION OF RESEARCH FINDINGS

Publication of the results of this trial will be governed by NHLBI and NIAID policies.

14.0 BIOHAZARD CONTAINMENT

All dangerous goods and materials, including diagnostic specimens and infectious substances, must be transported using packaging mandated by CFR 42 Part 72. Please refer to instructions detailed in the International Air Transport Association (IATA) Dangerous Goods Regulations.

15.0 STUDY GOVERNANCE

- There will be an Executive Committee, a DSMB, and External Advisory Committee.
- There will also be various other operational committees to ensure that the trial progresses smoothly.
- A Clinical Events Committee (CEC) charter will be developed.

16.0 REFERENCES

Aberg JA, et al. Primary care guidelines for the management of persons infected with HIV: 2013 update by the HIV Medicine Association of the Infectious Diseases Society of America. Clin Infect Dis 2014;58(1):1-10. PMID: 2434358.

Aberg JA, et al. HIV Medicine Association of the Infectious Diseases Society of America. Primary care guidelines for the management of persons infected with human immunodeficiency virus: 2009 update by the HIV Medicine Association of the Infectious Diseases Society of America. Clin Infect Dis 2009;49(5):651-81. PMID: 19640227.

Aberg JA, et al. Neutral effects of pitavastatin 4 mg and pravastatin 40 mg on blood glucose and HbA1c levels over 12 weeks: prespecified safety analysis from INTREPID (HIV-infected Patients and Treatment with Pitavastatin vs Pravastatin for Dyslipidemia), a Phase 4 trial. Endocrine Society of America 95th Annual Meeting and Expo; 2013; San Francisco, CA.

Ahmed MH, et al. The safety and effectiveness of statins as treatment for HIV-dyslipidemia: the evidence so far and the future challenges. Expert Opin Pharmacother 2012;13(13):1901-9. PMID: 22770622.

Amarenco P, et al. Effect of high-dose atorvastatin on renal function in subjects with stroke or transient ischemic attack in the SPARCL Trial. Stroke 2014;45(10):2974-82. Epub 2014 Aug 21.

Amet T, et al. Statin-induced inhibition of HIV-1 release from latently infected U1 cells reveals a critical role for protein prenylation in HIV-1 replication. Microbes Infect 2008;10(5):471-80.

Antiretroviral Therapy Cohort Collaboration. Causes of death in HIV-1-infected patients treated with antiretroviral therapy, 1996-2006: collaborative analysis of 13 HIV cohort studies. Clin Infect Dis 2010;50(10):1387-96.

Aviram M, et al. Lovastatin inhibits low-density lipoprotein oxidation and alters its fluidity and uptake by macrophages: in vitro and in vivo studies. Metabolism 1992;41(3):229-35.

Brenchley JM, et al. Microbial translocation is a cause of systemic immune activation in chronic HIV infection. Nat Med 2006;12(12):1365-71.

Bu DX, et al. Statin-induced Kruppel-like factor 2 expression in human and mouse T cells reduces inflammatory and pathogenic responses. J Clin Invest 2010;120(6):1961-70.

Burdo TH, et al. Soluble CD163, a novel marker of activated macrophages, is elevated and associated with noncalcified coronary plaque in HIV-infected patients. J Infect Dis 2011;204(8):1227-36.

Borges AH, et al. Factors contributing to risk for cancer among HIV-infected individuals, and evidence that earlier combination antiretroviral therapy will alter this risk. Curr Opin HIV AIDS 2014;9(1):34-40.

Burgstahler C, et al. Influence of a lipid-lowering therapy on calcified and noncalcified coronary plaques monitored by multislice detector computed tomography: results of the New Age II Pilot Study. Invest Radiol 2007;42(3):189-95.

Calza L, et al. Statin therapy decreases serum levels of high-sensitivity C-reactive protein and tumor necrosis factor-α in HIV-infected patients treated with ritonavir-boosted protease inhibitors. HIV Clin Trials 2012;13(3):153-61. PMID: 22592095.

Carlberg M, et al. Mevalonic acid is limiting for N-linked glycosylation and translocation of the insulin-like growth factor-1 receptor to the cell surface. Evidence for a new link between 3-hydroxy-3-methylglutaryl-coenzyme a reductase and cell growth. J Biol Chem 1996;271(29):17453-62.

Centers for Disease Control and Prevention. Who’s at Risk for HIV. Accessed September 8, 2014 from http://www.cdc.gov/hiv/risk/.

Chauvin B, et al. Drug-drug interactions between HMG-CoA reductase inhibitors (statins) and antiviral protease inhibitors. Clin Pharmacokinet 2013;52(10):815-31. PMID: 23703578.

Cholesterol Treatment Trialists’ (CTT) Collaboration. Baigent C, et al. Efficacy and safety of more intensive lowering of LDL cholesterol: a meta-analysis of data from 170,000 participants in 26 randomised trials. Lancet 2010;376(9753):1670-81. PMID: 21067804.

Colhoun HM, et al. Effects of atorvastatin on kidney outcomes and cardiovascular disease in patients with diabetes: an analysis from the Collaborative Atorvastatin Diabetes Study (CARDS). Am J Kidney Dis 2009;54(5):810-9.

Crum-Cianflone N, et al. Trends in the incidence of cancers among HIV-infected persons and the impact of antiretroviral therapy: a 20-year cohort study. AIDS 2009;23(1):41-50.

Currier JS, et al. Coronary heart disease in HIV-infected individuals. J Acquir Immune Defic Syndr 2003;33(4):506-12.

Data Collection on Adverse Events of Anti-HIV Drugs (D:A:D) Study Group. Smith C, et al. Factors associated with specific causes of death amongst HIV-positive individuals in the D:A:D Study. AIDS 2010;24(10):1537-48.

De Wit S, et al. Downregulation of CD38 activation markers by atorvastatin in HIV patients with undetectable viral load. AIDS 2011;25(10):1332-3.

Deeken JF, et al. The rising challenge of non-AIDS-defining cancers in HIV-infected patients. Clin Infect Dis 2012;55(9):1228-35.

Deeks SG. HIV infection, inflammation, immunosenescence, and aging. Ann Rev Med 2011;62:141-55.

del Real G, et al. Statins inhibit HIV-1 infection by down-regulating Rho activity. J Exp Med 2004;200(4):541-7.

Dolan SE, et al. Increased cardiovascular disease risk indices in HIV-infected women. J Acquir Immune Defic Syndr 2005;39(1):44-54.

Douglas K, et al. Meta-analysis: the effect of statins on albuminuria. Ann Intern Med 2006;145(2):117-24.

Downs JR, et al. Primary prevention of acute coronary events with lovastatin in men and women with average cholesterol levels: results of AFCAPS/TexCAPS. Air Force/Texas Coronary Atherosclerosis Prevention Study. JAMA 1998;279(20):1615-22.

Dreschler H, Impact of statin exposure on mortality and non-AIDS complications in HIV patients on HAART [abstract 765]. 20th Conference on Retroviruses and Opportunistic Infections; March 3-6, 2013; Atlanta, GA.

Durand M, et al. Association between HIV infection, antiretroviral therapy, and risk of acute myocardial infarction: a cohort and nested case-control study using Quebec's public health insurance database. J Acquir Immune Defic Syndr 2011;57(3):245-53.

Ekstedt M, et al. Statins in non-alcoholic fatty liver disease and chronically elevated liver enzymes: a histopathological follow-up study. J Hepatol 2007;47(1):135-41.

El-Sadr WM, et al. CD4+ count-guided interruption of antiretroviral treatment. N Engl J Med 2006;355(22):2283-96.

Eriksson M, et al. Comparative efficacy of pitavastatin and simvastatin in high-risk patients: a randomized controlled trial. Adv Ther 2011;28(9):811-23. PMID: 2187453.

Eriksson M, et al. Long-term efficacy of pitavastatin versus simvastatin. Adv Ther 2011;28(9):799-810. PMID: 21874537.

Foster T, et al. Atorvastatin and antioxidants for the treatment of nonalcoholic fatty liver disease: the St Francis Heart Study randomized clinical trial. Am J Gastroenterol 2011;106(1):71-7. PMID: 20842109.

Freiberg MS, et al. HIV infection and the risk of acute myocardial infarction. JAMA Intern Med 2013;173(8):614-22.

French AL, et al. Trends in mortality and causes of death among women with HIV in the United States: a 10-year study. J Acquir Immune Defic Syndr 2009;51(4):399-406.

Friis-Moller N, et al. Combination antiretroviral therapy and the risk of myocardial infarction. N Engl J Med 2003;349(21):1993-2003.

Fujino M, et al. Pitavastatin-induced downregulation of CCR2 and CCR5 in monocytes is associated with the arrest of cell-cycle in S phase. Atherosclerosis 2006;187(2):301-8.

Galli L, et al. Use of statins and risk of AIDS-defining and non-AIDS defining malignancies among HIV-1 infected patients on antiretroviral therapy. AIDS 2014;28(16):2407-15. PMID: 25160933.

Ganesan A, et al. High dose atorvastatin decreases cellular markers of immune activation without affecting HIV-1 RNA levels: results of a double-blind randomized placebo controlled clinical trial. J Infect Dis 2011;203(6):756-64.

Gerber JG, et al. Effect of efavirenz on the pharmacokinetics of simvastatin, atorvastatin, and pravastatin: results of AIDS Clinical Trials Group 5108 Study. J Acquir Immune Defic Syndr 2005;39(3):307-12. PMID: 15980690.

Giguere JF and Tremblay MJ. Statin compounds reduce human immunodeficiency virus type 1 replication by preventing the interaction between virion-associated host intercellular adhesion molecule 1 and its natural cell surface ligand LFA-1. J Virol 2004;78(21):12062-5.

Gilbert C, et al. Statins could be used to control replication of some viruses, including HIV-1. Viral Immunol 2005;18(3):474-89.

Gill J, et al. Antiretroviral Therapy Cohort Collaboration. Causes of death in HIV-1-infected patients treated with antiretroviral therapy, 1996-2006: collaborative analysis of 13 HIV cohort studies. Clin Infect Dis 2010;50(10):1387-96.

Giroux LM, et al. Simvastatin inhibits the oxidation of low-density lipoproteins by activated human monocyte-derived macrophages. Biochim Biophys Acta 1993;1165(3):335-8.

Gómez-Domínguez, et al. A pilot study of atorvastatin treatment in dyslipid, non-alcoholic fatty liver patients. Aliment Pharmacol Ther 2006;23(11):1643-7.

Gotto AM Jr and Moon J. Pitavastatin for the treatment of primary hyperlipidemia and mixed dyslipidemia. Expert Rev Cardiovasc Ther 2010;8(8):1079-90. PMID: 20670185.

Greene M, et al. Management of human immunodeficiency virus infection in advanced age. JAMA 2013;309(13):1397-405.

Greenwood J and Mason JC. Statins and the vascular endothelial inflammatory response. Trends Immunol 2007;28(2):88-98. PMID: 17197237.

Grinspoon SK, et al. State of the science conference: Initiative to decrease cardiovascular risk and increase quality of care for patients living with HIV/AIDS: executive summary. Circulation 2008;118(2):198-210.

Gumprecht J, et al. Comparative long-term efficacy and tolerability of pitavastatin 4 mg and atorvastatin 20-40 mg in patients with type 2 diabetes mellitus and combined (mixed) dyslipidaemia. Diabetes Obes Metab 2011;13(11):1047-55. PMID: 21812889.

Guo H, et al. Rosuvastatin inhibits MMP-2 expression and limits the progression of atherosclerosis in LDLR-deficient mice. Arch Med Res 2009;40(5):345-51.

Han J, et al. Pitavastatin downregulates expression of the macrophage type B scavenger receptor, CD36. Circulation 2004;109(6):790-6.

Han KH, et al. HMG-CoA reductase inhibition reduces monocyte CC chemokine receptor 2 expression and monocyte chemoattractant protein-1-mediated monocyte recruitment in vivo. Circulation 2005;111(11):1439-47.

Hiro T, et al. Effect of intensive statin therapy on regression of coronary atherosclerosis in patients with acute coronary syndrome: a multicenter randomized trial evaluated by volumetric intravascular ultrasound using pitavastatin versus atorvastatin (JAPAN-ACS [Japan assessment of pitavastatin and atorvastatin in acute coronary syndrome] study). J Am Coll Cardiol 2009;54(4):293-302.

Hicks, KA, et al. Standardized definitions for end point events in cardiovascular trials. On behalf of the Standardized Data Collection for Cardiovascular Trials Initiative. Draft November 2012.

Hou ZH, et al. Prognostic value of coronary CT angiography and calcium score for major adverse cardiac events in outpatients. JACC Cardiovasc Imaging 2012;5(10):990-9.

Hsue PY, et al. Immunologic basis of cardiovascular disease in HIV-infected adults. J Infect Dis 2012;205 Suppl 3:S375-82.

Hulten E, et al. HIV positivity, protease inhibitor exposure and subclinical atherosclerosis: a systematic review and meta-analysis of observational studies. Heart 2009;95(22):1826-35.

Hyogo H, et al. Atorvastatin improves disease activity of nonalcoholic steatohepatitis partly through its tumor necrosis factor-α-lowering property. Dig Liver Dis 2012;44(6):492-6.

Inoue K, et al. Serial coronary CT angiography-verified changes in plaque characteristics as an end point: evaluation of effect of statin intervention. JACC Cardiovasc Imaging 2010;3(7):691-8.

Kitagawa T, et al. Characterization of noncalcified coronary plaques and identification of culprit lesions in patients with acute coronary syndrome by 64-slice computed tomography. JACC Cardiovasc Imaging 2009;2(2):153-60.

Klein D, et al. Do protease inhibitors increase the risk for coronary heart disease in patients with HIV-1 infection? J Acquir Immune Defic Syndr 2002;30(5):471-7.

Kodama K, et al. Stabilization and regression of coronary plaques treated with pitavastatin proven by angioscopy and intravascular ultrasound--the TOGETHAR trial. Circ J 2010;74(9):1922-8.

Koenen RR, Weber C. Therapeutic targeting of chemokine interactions in atherosclerosis. Nat Rev Drug Discov 2010;9(2):141-53.

Kuller LH, et al. Inflammatory and coagulation biomarkers and mortality in patients with HIV infection. PLoS Med 2008;5(10):e203.

Kwak B, et al. Statins as a newly recognized type of immunomodulator. Nat Med 2000;6(12):1399-402.

Lang S, et al. Increased risk of myocardial infarction in HIV-infected patients in France, relative to the general population. AIDS 2010;24(8):1228-30.

Lewden C, et al. Changes in causes of death among adults infected by HIV between 2000 and 2005: The "Mortalité 2000 and 2005" surveys (ANRS EN19 and Mortavic). J Acquir Immune Defic Syndr 2008;48(5):590-8.

Libby P, et al. Inflammation and atherosclerosis. Circulation 2002;105(9):1135-43.

Libby P, et al. Progress and challenges in translating the biology of atherosclerosis. Nature 2011;473(7347):317-25.

Lichtenstein K, et al. and HIV Outpatient Study Investigators. Statin use is associated with incident diabetes mellitus among patients in the HIV Outpatient Study [abstract 767]. 20th Conference on Retroviruses and Opportunistic Infections; March 3-6, 2013; Atlanta, GA.

Longenecker CT, et al. Rosuvastatin preserves renal function and lowers cystatin C in HIV-infected subjects on antiretroviral therapy: The SATURN-HIV Trial. Clin Infect Dis 2014;59(8):1148-56.

Malvestutto CD, et al. Lack of pharmacokinetic interactions between pitavastatin and efavirenz or darunavir/ritonavir. J Acquir Immune Defic Syndr 2014; Sep 8. [Epub ahead of print].

Marin B, et al. Non-AIDS-defining deaths and immunodeficiency in the era of combination antiretroviral therapy. AIDS 2009;23(13):1743-53.

Maruyama T, et al. Comparison of preventive effect on cardiovascular events with different statins. The CIRCLE study. Circ J 2011;75(8):1951-9. PMID: 21673458.

McComsey GA, et al. Effect of statins on immune activation and inflammation in HIV+ subjects on ART: a randomized placebo controlled trial [abstract 186LB]. 20th Conference on Retroviruses and Opportunistic Infections; March 3-6, 2013; Atlanta, GA.

Mira E, et al. Statins induce regulatory T cell recruitment via a CCL1 dependent pathway. J Immunol 2008;181(5):3524-34.

Miyaki T, et al. Pitavastatin inhibits hepatic steatosis and fibrosis in non-alcoholic steatohepatitis model rats. Hepatol Res 2011;41(4):375-85.

Moncunill G, et al. Evaluation of the anti-HIV activity of statins. AIDS 2005;19(15):1697-700.

Montecucco F, et al. Statins inhibit C-reactive protein-induced chemokine secretion, ICAM-1 upregulation and chemotaxis in adherent human monocytes. Rheumatology (Oxford) 2009;48(3):233-42.

Moore RD, et al. Association between use of HMG CoA reductase inhibitors and mortality in HIV-infected patients. PLoS ONE 2011;6(7):e21843.

Mulhaupt F, et al. Statins (HMG-CoA reductase inhibitors) reduce CD40 expression in human vascular cells. Cardiovasc Res 2003;59(3):755-66.

Nabatov AA, et al. Statins disrupt CCR5 and RANTES expression levels in CD4(+) T lymphocytes in vitro and preferentially decrease infection of R5 versus X4 HIV-1. PLoS ONE 2007;2(5):e470.

Nakamura T, et al. Rapid stabilization of vulnerable carotid plaque within 1 month of pitavastatin treatment in patients with acute coronary syndrome. J Cardiovasc Pharmacol 2008;51(4):365-71.

Negredo E, et al. The effect of atorvastatin treatment on HIV-1-infected patients interrupting antiretroviral therapy. AIDS 2006;20(4):619-21.

Neuhaus J, et al. INSIGHT SMART and ESPRIT study groups. Risk of all-cause mortality associated with nonfatal AIDS and serious non-AIDS events among adults infected with HIV. AIDS 2010;24(5):697-706.

Nikolic D, et al. A meta-analysis of the role of statins on renal outcomes in patients with chronic kidney disease. Is the duration of therapy important? Int J Cardiol 2013;168(6):5437-47.

Obel N, et al. Ischemic heart disease in HIV-infected and HIV-uninfected individuals: a population-based cohort study. Clin Infect Dis 2007;44(12):1625-31.

Overton ET, et al. Effect of statin therapy in reducing the risk of serious non-AIDS-defining events and nonaccidental death. Clin Infect Dis 2013;56(10):1471-9.

Packard RR, et al. Innate and adaptive immunity in atherosclerosis. Semin Immunopathol 2009;31(1):5-22.

Palella FJ, Jr., et al. Mortality in the highly active antiretroviral therapy era: changing causes of death and disease in the HIV outpatient study. J Acquir Immune Defic Syndr 2006;43(1):27-34.

Palmer SC, et al. HMG CoA reductase inhibitors (statins) for people with chronic kidney disease not requiring dialysis. Cochrane Database Syst Rev 2014;5:CD007784.

Paton NI, et al. Effects of hydroxychloroquine on immune activation and disease progression among HIV-infected patients not receiving antiretroviral therapy: a randomized controlled trial. JAMA 2012;308(4):353-61.

Randomised trial of cholesterol lowering in 4444 patients with coronary heart disease: the Scandinavian Simvastatin Survival Study (4S). Lancet 1994;344(8934):1383-9.

Rasmussen LD, et al. Statin therapy and mortality in HIV-infected individuals; a Danish nationwide population-based cohort study. PLoS ONE 2013;8(3):e52828.

Ridker PM, et al. Rosuvastatin to prevent vascular events in men and women with elevated C-reactive protein. N Engl J Med 2008;359(21):2195-207.

Romano M, et al. Inhibition of monocyte chemotactic protein-1 synthesis by statins. Lab Invest 2000;80(7):1095-100.

Rominger A, et al. 18F-FDG PET/CT identifies patients at risk for future vascular events in an otherwise asymptomatic cohort with neoplastic disease. J Nucl Med 2009;50(10):1611-20.

Ross AC, et al. Endothelial activation markers are linked to HIV status and are independent of antiretroviral therapy and lipoatrophy. J Acquir Immune Defic Syndr 2008;49(5):499-506.

Ross R. Atherosclerosis--an inflammatory disease. N Engl J Med 1999;340(2):115-26.

Sackoff JE, et al. Causes of death among persons with AIDS in the era of highly active antiretroviral therapy: New York City. Ann Intern Med 2006;145(6):397-406.

Sacks FM, et al. The effect of pravastatin on coronary events after myocardial infarction in patients with average cholesterol levels. Cholesterol and Recurrent Events Trial investigators. N Engl J Med 1996;335(14):1001-9.

Samineni D, et al. Steady-state pharmacokinetic interactions of darunavir/ritonavir with lipid-lowering agent rosuvastatin. J Clin Pharmacol 2012;52(6):922-31. PMID: 21712498.

Shepherd J. The West of Scotland Coronary Prevention Study: a trial of cholesterol reduction in Scottish men. Am J Cardiol 1995;76(9):113C-117C.

Shimojima M, et al. Rapid changes in plaque composition and morphology after intensive lipid lowering therapy: study with serial coronary CT angiography. Am J Cardiovasc Dis 2012;2(2):84-8.

Silverberg MJ, et al. Response to newly prescribed lipid-lowering therapy in patients with and without HIV infection. Ann Intern Med 2009;150(5):301-13.

Simon TG, et al. Statin use is associated with a reduced risk of fibrosis progression in chronic hepatitis C. J Hepatol 2014; Aug 15; Epub ahead of print.

Singh P, et al. Influence of statins on MHC class I expression. Ann N Y Acad Sci 2009;1173:746-51.

Singh S, et al. Comparative effectiveness and toxicity of statins among HIV-infected patients. Clin Infect Dis 2011;52(3):387-95. PMID: 21189273.

Smith C, et al. Factors associated with specific causes of death amongst HIV-positive individuals in the D:A:D Study. AIDS 2010;24(10):1537-48.

Spagnuolo V, et al. Association between statin use and type-2 diabetes mellitus occurrence among HIV-1+ patients receiving ART [abstract 766]. 20th Conference on Retroviruses and Opportunistic Infections; March 3-6, 2013; Atlanta, GA.

REFERENCES (Cont'd)

Sponseller CA, et al. After 52 weeks, pitavastatin is superior to pravastatin for LDL-C lowering in patients with HIV [abstract 751LB]. 20th Conference on Retroviruses and Opportunistic Infections; March 3-6, 2013; Atlanta, GA.

Stein JH, et al. Ultrasonographic measures of cardiovascular disease risk in antiretroviral treatment-naive individuals with HIV infection. AIDS 2013;27(6):929-37.

Stone NJ, et al. 2013 ACC/AHA Guidelines on the treatment of blood cholesterol to reduce atherosclerotic cardiovascular risk in adults: a report of the American College of Cardiology/American Heart Association Task Force on Practice Guidelines. J Am Coll Cardiol 2014;63(25 Pt B):2889-934.

Subramanian S, et al. Arterial inflammation in patients with HIV. JAMA 2012;308(4):379-86.

Tarantino G, et al. Are hepatic steatosis and carotid intima media thickness associated in obese patients with normal or slightly elevated gamma-glutamyl-transferase? J Transl Med 2012;10:50.

Tawakol A, et al. Intensification of statin therapy results in a rapid reduction in atherosclerotic inflammation: results of a multicenter fluorodeoxyglucose-positron emission tomography/computed tomography feasibility study. J Am Coll Cardiol 2013;62(10):909-17.

Tenorio AR, et al. Soluble markers of inflammation and coagulation but not T-cell activation predict non-AIDS-defining morbid events during suppressive antiretroviral treatment. J Infect Dis 2014;210(8):1248-59.

Teramoto T, et al. New evidence on pitavastatin: efficacy and safety in clinical studies. Expert Opin Pharmacother 2010;11(5):817-28.

Triant VA, et al. Increased acute myocardial infarction rates and cardiovascular risk factors among patients with human immunodeficiency virus disease. J Clin Endocrinol Metab 2007;92(7):2506-12.

Triant VA, et al. Association of immunologic and virologic factors with myocardial infarction rates in a US healthcare system. J Acquir Immune Defic Syndr 2010;55(5):615-9.

Tseng ZH, et al. Sudden cardiac death in patients with human immunodeficiency virus infection. J Am Coll Cardiol 2012;59(21):1891-6.

Vasankari T, et al. Oxidized LDL and thickness of carotid intima-media are associated with coronary atherosclerosis in middle-aged men: lower levels of oxidized LDL with statin therapy. Atherosclerosis 2001;155(2):403-12.

Veillard, NR, et al. Simvastatin modulates chemokine and chemokine receptor expression by geranylgeranyl isoprenoid pathway in human endothelial cells and macrophages. Atherosclerosis 2006; 188(1):51-8.

Wada N, et al. Cause-specific life expectancies after 35 years of age for human immunodeficiency syndrome-infected and human immunodeficiency syndrome-negative individuals followed simultaneously in long-term cohort studies, 1984-2008. Am J Epidemiol 2013;177(2):116-25.

Waehre T, et al. Hydroxymethylglutaryl coenzyme a reductase inhibitors down-regulate chemokines and chemokine receptors in patients with coronary artery disease. J Am Coll Cardiol 2003;41:1460-7.

Wang W, et al. Simvastatin ameliorates liver fibrosis via mediating nitric oxide synthase in rats with non-alcoholic steatohepatitis-related liver fibrosis. PLoS One 2013;8(10):e76538.

Weber R, et al. Swiss HIV Cohort Study (SHCS). Decreasing mortality and changing patterns of causes of death in the Swiss HIV Cohort Study. HIV Med 2013;14(4):195-207.

Willig JH, et al. Clinical inertia in the management of low-density lipoprotein abnormalities in an HIV clinic. Clin Infect Dis 2008;46(8):1315-8.

Wong WW, et al. HMG-CoA reductase inhibitors and the malignant cell: the statin family of drugs as triggers of tumor-specific apoptosis. Leukemia 2002;16(4):508-19.

REFERENCES (Cont'd)

World Health Organization. HIV Epidemiology 2012. http://www.who.int/hiv/data/en/. Site accessed June 30, 2013.

Yamakawa T, et al. Influence of pitavastatin on glucose tolerance in patients with type 2 diabetes mellitus. J Atheroscler Thromb 2008;15(5):269-75.

Yarasheski KE, et al. 18FDG PET-CT imaging detects arterial inflammation and early atherosclerosis in HIV-infected adults with cardiovascular disease risk factors. J Inflamm (Lond) 2012;9(1):26.

Yasui Y, et al. A lipophilic statin, pitavastatin, suppresses inflammation-associated mouse colon carcinogenesis. Int J Cancer 2007;121(10):2331-9. PMID: 17657716.

Yokote K, et al. CHIBA study investigators. Influence of statins on glucose tolerance in patients with type 2 diabetes mellitus: subanalysis of the collaborative study on hypercholesterolemia drug intervention and their benefits for atherosclerosis prevention (CHIBA study). J Atheroscler Thromb 2009;16(3):297-98.

Zanni MV and Grinspoon SK. HIV-specific immune dysregulation and atherosclerosis. Curr HIV/AIDS Rep 2012;9(3):200-205.

Zanni MV, et al. Increased coronary atherosclerotic plaque vulnerability by coronary computed tomography angiography in HIV-infected men. AIDS 2013;27(8):1263-72.

Zheng C, et al. Statins suppress apolipoprotein CIII-induced vascular endothelial cell activation and monocyte adhesion. Eur Heart J 2013;34(8):615-24.

Zineh I, et al. Modulatory effects of atorvastatin on endothelial cell-derived chemokines, cytokines, and angiogenic factors. Pharmacotherapy 2006;26(3):333-40.

APPENDIX I: SAMPLE INFORMED CONSENT

For the REPRIEVE (A5332) Protocol, FINAL Version 2.0, 12/19/14

Randomized Trial to Prevent Vascular Events in HIV (The REPRIEVE Study)

INTRODUCTION

You are being asked to take part in this research study because you are infected with the human immunodeficiency virus (HIV), the virus that causes AIDS, and you are taking HIV medications.

This study is sponsored by the National Institutes of Health (NIH). The doctor in charge of this study at this site is: (insert name of Principal Investigator). Before you decide if you want to be a part of this study, we want you to know about the study.

WHY IS THIS STUDY BEING DONE?

Since people started taking HIV medications, illness from AIDS has decreased, but other serious diseases, like heart disease, have increased. HIV causes inflammation (irritation) inside the body that cannot be felt but can be measured. These tests will be described later in this consent form. Inflammation may contribute to diseases such as heart disease that have become some of the leading causes of death in people with HIV. HIV medications can lower inflammation somewhat, however sometimes the levels of inflammation can remain higher compared to people who are not infected with HIV.

Statins are a group of medicines used to lower the levels of cholesterol and triglycerides (fat in the blood) that people make and to prevent heart-related disease events such as heart attacks in persons with high risk for heart attacks. Studies have shown that statins may have other benefits. For example, by decreasing levels of inflammation, statins may have an effect to protect against heart disease and its related events. In addition, statins may have some beneficial effects on some other diseases like some cancers or kidney problems.

The most recent guidelines from the American College of Cardiology and the American Heart Association recommend the use of statins if someone is at risk of heart-related disease based on many different factors. People living with HIV may not be considered at high-risk for heart disease using the current guidelines. However, HIV infection, HIV medications, and chronic inflammation may put you at higher risk for these diseases, although we do not know if you would benefit from taking a statin. You are eligible for this study because you are not recommended to take statins using the current guidelines. Your participation in this study will help us determine if the use of statins can prevent heart-related disease among people with HIV infection. The results of this study may help to create guidelines for the prevention of heart disease in HIV infection.

Pitavastatin is a statin that, along with a diet, has been approved by the US Food and Drug Administration for the treatment of high cholesterol. It also lowers triglyceride levels in the blood. It has not been studied to see if it reduces heart-related disease or death. Pitavastatin was chosen because there are thought to be few interactions between pitavastatin and commonly used HIV medications.

The main purpose of this clinical trial is to see if pitavastatin can prevent heart disease and heart-related deaths in people with HIV infection who are taking HIV medications. We will also study the safety of pitavastatin.

HOW MANY PEOPLE WILL BE IN THIS STUDY?

About 6500 people will take part in this study.

WHAT DO I HAVE TO DO IF I AM IN THIS STUDY?

Study visits
If you enter the study, you will be seen in the clinic about 6 times the first year. After that, the study visits are every 4 months for the next 3 ½-5 ¾ years. This means that you will be in the study for about 3 ½ - 6 years, depending on when you enter the study. The study staff will tell you about how long each visit will be. More details about the visits and procedures are below.

If you do not enter the study
If you decide not to take part in this study after signing the consent form, or if you do not meet the eligibility requirements, we will still use some of your information. As part of this screening visit, some demographic (for example, age, gender, race), clinical (for example, disease condition, diagnosis), and laboratory (for example, safety tests) information is being collected from you so that AIDS Clinical Trials Group (ACTG) researchers may help determine whether there are patterns or common reasons why people do not join a study.

Study drugs
If you enter the study, you will be randomly assigned (as if by the toss of a coin) to get either pitavastatin or a placebo for pitavastatin. The placebo is a tablet that looks just like pitavastatin but does not contain any active medication. Therefore, there is a chance that if you are randomized to the placebo you will receive no treatment during your participation in the study. We use placebos in clinical studies to learn if the effects seen in the trial are truly from the study medicine or from other reasons. Neither you nor the study staff will know your assignment. You will not find out your assignment until after the entire study is over and the results of the study are known. You and your doctor can be told of the assignment at any point if it is necessary for your health.

You will take the study medicine (either pitavastatin or the placebo for pitavastatin) once a day, every day, throughout the study period, with or without food. The dose is 4 mg. We recommend that you take the study medicine at the same time each day. These drugs are provided by the study. It is very important that you take your medicines as directed. Antiretroviral drugs (treatment for HIV) will not be provided by the study.

Study procedures
The study staff can answer any questions you have about individual study visits and the procedures. The table below can be used as a quick reference, along with the explanations that follow.

Procedure	Screening[1]	Entry[2]	Month 1	Visits every 4 months (starting at month 4)	Annual visits (starting at month 12)	Final visit
Physical exam	X		X		X	X
Heart disease risk assessment	X					
Heart disease risk factors	X					
Diet and exercise questions		X				X
Dispense lifestyle information		X			X	X
Health and medicine questions	X	X	X	X	X	X
Blood collected	X	X	X		X	X
Urine collected		X				
Fasting blood tests	X	X			X	X
Pregnancy test	X	X	X	X	X	X
Electrocardiogram		X				
Pill count questionnaire			X	X	X	X
Pills dispensed and pills counted		Pills dispensed only	Pill count only	X	X	Pill count only

[1] Screening visit: before you can enter the study, you will need to come to the clinic to have evaluations done to make sure that you can take part in the study.
[2] Entry visit: if you meet the entry requirements, you will enroll in the study.

If you leave the study early, or have to stop taking the study medication before the study is over, you will have the procedures listed in the table below.

Procedure	Stopping the study or the study treatment early
Physical exam	X
Health and medicine questions	X
Pregnancy test	X
Blood collected	X
Fasting blood tests	X
Pills counted	X
Pill count questionnaire	X

Explanation of study procedures

<u>Physical exam</u>
You will have a physical exam at screening. At other visits after entry, the extent of the exam will depend on how you are feeling at that visit. You will have vital signs taken, including, blood pressure and pulse. You will have measurements taken of your waist and height and weight. You will be asked questions about your health and medicines.

Heart disease risk assessment

At screening we will ask specific questions to assess eligibility based on cardiovascular disease risk. At screening you will also be asked about cardiovascular risk factors including your family history, smoking, alcohol use, substance use, diet, and exercise.

Lifestyle /risk reduction counseling

If you join the study, you will be given information about a healthy diet and the importance of exercise, smoking cessation, and taking your antiretroviral therapy and study medication as prescribed. We will provide this information at all annual visits.

ECG

An electrocardiogram, or ECG, will be done at entry. An ECG is an electrical tracing of your heart that can show how hard it is working. You will have to lie very still for up to 10 minutes while the ECG is being done.

Blood collected

Blood will be collected from you for different tests if they are not available as part of your routine medical care or for safety reasons. These include routine tests to evaluate your blood counts, liver, and kidney function.

At screen, month 1, and month 12 we will collect blood from you to evaluate your liver function. This test is required as part of your participation in the study. Approximately 1 teaspoon of blood at each of these visits will be collected for this test.

At screen and the end of study visit we will use the results of your CBC (blood count) and kidney function done as part of routine care by your medical provider.

At screen and annual visits we will use the results of your CD4 T-cell count (how many infection fighting cells are in your blood) done as part of routine care by your medical provider.

At entry and annual visits we will use the results of your HIV viral load (how much HIV is in your blood) done as part of your routine care by your medical provider.

At screen we will check your cholesterol (fat found in your blood) levels.

You will be told the results of these routine tests.

At entry, all annual visits, and the end of study visit, some blood will be collected and stored for tests that will be done later on in the study or after the study is over. These tests will measure the levels of fat and sugar in your blood. Some of these tests will be used for metabolic blood tests (measures how your body uses the food that you eat). You do not need to agree to store this blood to join the study and you may change your mind about storing your blood at any time. Your blood may be stored (with usual protectors of identity) for an indefinite length of time. You will not be told of the results of the research done on your blood.

At each of these visits, approximately 2 teaspoons of your blood will be collected and stored for these purposes.

Do you agree to let us store your samples for tests to measure the levels of fat and sugar in your blood?

________ YES ________ NO _______Initials

At entry, all annual visits, and the end of study visit, some of your blood will be collected and stored use for future ACTG-approved research on conditions including cardiovascular disease, HIV, inflammation, cancer or statin medications. You do not need to agree to store this blood to join the study and you may change your mind about storing your blood at any time. Your blood may be stored (with the usual protectors of identity) for an indefinite length of time. You will not be told of the results of the research done on your blood.

Approximately 4-8 teaspoons of blood will be collected at the entry and each annual visit for these purposes.

Do you agree to let us store your samples for future research on conditions including cardiovascular disease, HIV, inflammation, cancer, and statin medications?

________ YES ________ NO _______Initials

For ACTG sites: Approximately 1 teaspoon of blood will be collected to look at genes that may affect your risk for cardiovascular disease and how statins work in your body. Genetic testing is a laboratory test that looks at differences in people’s genes. Your body, like all living things, is made up of cells, and cells contain deoxyribonucleic acid, also known as “DNA.” DNA is like a string of information put together in a certain order. Parts of the string make up “genes.” Genes contain instructions on how to make your body work and fight disease. The testing in this study will focus on certain genes that are known to have an effect on cardiovascular disease and how your body uses statins. New genes of interest may be identified in the future and may also be looked at.

Your body’s genetic makeup is unique to you, so there is a risk with genetic research that even with all of the security measures in place, someone using your samples or genetic information may still find out which information is yours. However, this risk today is very small, but it may increase with time since science and technology are developing rapidly.

For US ACTG sites: In the event that your genetic information becomes linked to your name, the US federal law called the Genetic Information Nondiscrimination Act (GINA) helps protect you. This law prohibits health insurance companies, group health plans, and most employers from denying services based on your genetic information. However, GINA does not protect against discrimination by companies that sell life insurance, disability insurance, or long-term care insurance.

We would like to use some of the blood we collect to look at your genes (DNA). Do you agree to this genotyping?

________ YES ________ NO _______Initials

If at a later date you change your mind and want your samples destroyed, contact the research staff. There are two ways to withdraw your permission. You could allow researchers to remove all your personal identifiers from your samples, so that they are not linked to you anymore. These samples will then become anonymous. Or, you can ask researchers to destroy your samples, so that they cannot be

used for future research. However, in either case, researchers will not be able to destroy samples or information from research that is already underway.

For Non-US sites: Your samples may be shipped and stored outside of your country and may be used by researchers outside of your country.

Urine collected
Urine will be collected at entry to check for protein in your urine. The results from this test will not be known immediately; therefore we cannot make sure that you will be told the results of this test.

Fasting blood tests
Before the screen, entry and all annual visits you should not eat or drink anything, including food, beverages, candy, or gum for 8 hours before your visit. You are encouraged to drink water before your visits. If you are not fasting we will ask you to return while fasting to have your blood drawn within 7 days of the study visit.

Study drugs given to you
Study drugs will be given to you at entry and every 4 months. No study drugs will be given to you at your final study visit.

Pill count
After you start the study the study staff will give ask you to bring in your pill bottles at every visit. They will count the number of pills left over.

Questionnaires
You will be asked questions about your diet and exercise at entry and will be repeated the final study visit.

HOW LONG WILL I BE IN THIS STUDY?

You will be in this study about 3 ½ - 6 years (42 – 72 months) depending on when you join. As soon as the first person who joined this study completes 72 months, the study will be over.

WHY WOULD THE DOCTOR TAKE ME OFF THIS STUDY EARLY?

The study doctor may need to take you off the study early without your permission if:

- the doctor thinks it is in your best interest
- the study is cancelled
- you are not able to attend the study visits as required by the study

The study doctor may also need to take you off the study drug without your permission if:

- you are not able to take the study drug as required by the study
- continuing the study drug may be harmful to you
- you need a treatment that you may not take while on the study.
- you become pregnant

If you must stop taking the study drug before the study is over, we will ask you to continue to be part of the study and return for some study visits and procedures.

<u>If you have to permanently stop taking the study drug, or if you leave the study, how would pitavastatin be provided?</u>

During the study:

If you must permanently stop taking study-provided pitavastatin before your study participation is over, the study staff will discuss other options that may be of benefit to you.

After the study:

After you have completed your study participation, the study will not be able to continue to provide you with the pitavastatin you received on the study. If continuing to take this or a similar drug would be of benefit to you, the study staff will discuss how you may be able to obtain the drug.

WHAT ARE THE RISKS OF THE STUDY?

The drug used in this study may have side effects, some of which are listed below. Please note that these lists do not include all the side effects seen with this drug. These lists include the more serious or common side effects with a known or possible relationship. If you have questions concerning the additional study drug side effects please ask the medical staff at your site.

There is a risk of serious or life-threatening side effects when non-study medications are taken with the study drug. For your safety, you must tell the study doctor or nurse about all medications you are taking before you start the study and also before starting any new medications while on the study. Also, you must tell the study doctor or nurse before enrolling in any other clinical trials while on this study.

<u>Risks of Pitavastatin</u>

- Muscle problems. Pitavastatin can occasionally cause serious muscle problems that can lead to kidney problems, including kidney failure and rarely, death.
- Liver problems. Pitavastatin can occasionally cause liver problems that may rarely be serious or cause death. Your study nurse or doctor will do blood tests to check your liver before you start taking pitavastatin and while you take it.
- Be sure to let your doctor or study nurse know immediately if you have any of these problems:
    - Muscle problems like weakness, tenderness, or pains that happen without a good reason, especially if you also have a fever or feel more tired than usual.
    - Nausea and vomiting.
    - Passing brown or dark-colored urine.
    - Feeling more tired than usual.
    - Noticing the skin and whites of your eyes become yellow.
    - Having stomach pain.

Other problems that have been caused by pitavastatin include headaches, rash (which rarely may be severe or fatal), severe allergic reaction or swelling, constipation, gas, diarrhea, pain or numbness in arms or legs, tendon rupture, urinary tract infection, dizziness, memory impairment, and depression. All of these problems are uncommon to rare.

<u>Risks of drawing blood</u>

Taking blood may cause some discomfort, lightheadedness, bleeding, swelling, or bruising where the needle enters the body, and in rare cases, fainting, or infection.

Risks of fasting
Some people find fasting and not smoking or consuming caffeine to be bothersome. It may make some individuals feel anxious, irritable, or hungry. Patients who are required to take their morning medications with food should wait until after the visit has been completed to take their medications.

Risks of ECG
You may experience mild irritation, slight redness and itching on your skin where the electrodes from the electrocardiogram machine are placed.

For ACTG sites: Genetic Testing
The results of your genetic tests are for research purposes only and no individual results will be given back to you. The results of the genetic studies will never become a part of your medical record. We will protect your confidentiality to the fullest extent. Blood samples for genetic studies will be identified in a way in order to maintain your confidentiality.

Research study results will not be given to your family members, insurance companies, employers, or third parties without your written permission and approval of the Institutional Review Board at _________.

Unknown risks
Other side effects that are not known at this time could happen during the study. All drugs have a possible risk of an allergic reaction, which if not treated right away, could become life-threatening. During the study, you will be told about any new information that may affect your decision to stay in the study. If you decide to stay in the study, you will be asked to sign an updated consent form. If you decide to leave the study early, the study staff will talk with you about your treatment options.

ARE THERE RISKS RELATED TO PREGNANCY?

Pitavastatin is unsafe for unborn babies. The risks to the unborn baby include birth defects, premature delivery, or death. If you are having sex that could lead to pregnancy, you must agree not to become pregnant.

If you can become pregnant, you must have a pregnancy test before you enter this study and at every visit (1 teaspoon of blood or a urine specimen will be collected) and at any time that pregnancy is suspected. This test must show that you are not pregnant. If you become pregnant or think you may be pregnant at any time during the study, tell your study staff right away. The study staff will talk to you about your choices.

Because of the risk involved, you and your partner must use at least one accepted form of birth control that you discuss with the study staff. You must start an accepted form of birth control at least two weeks before you start study drug and continue to use an accepted form of birth control until at least 6 weeks after you stop the study drug. If you are having sex that could lead to pregnancy, and do not use an accepted form of birth control, your study doctor will take you off of the study drug. You may choose from the birth control methods listed below:

- condoms, with a spermicidal agent
- a diaphragm or cervical cap with spermicide
- an IUD (intrauterine device)
- tubal ligation
- hormone-based contraceptive

If you become pregnant while on study, the study staff would like to obtain information from you about the outcome of the pregnancy (even if it is after your participation in the study ends). If you are taking anti-HIV drugs when you become pregnant, your pregnancy will be reported to an international database that collects information about pregnancies in women taking anti-HIV drugs. This report will not use your name or other information that could be used to identify you.

Breastfeeding

It is not known whether the study drug pass through the breast milk and may cause harm to your infant. Women who start breastfeeding must stop taking the provided study drug.

ARE THERE BENEFITS TO TAKING PART IN THIS STUDY?

Studies have shown statins to provide a benefit in terms of preventing heart disease in HIV uninfected patients with inflammation, but the effects of statins to prevent heart disease in HIV-infected patients is not known. If you take part in this study, there may be a direct benefit to you, but no guarantee can be made. You may benefit from learning about your risk of a cardiovascular event, but it is also possible that you may receive no benefit from being in this study either because the drug may not work or because you are assigned to placebo. Information learned from this study may help others who have HIV and are at risk of cardiovascular disease.

WHAT OTHER CHOICES DO I HAVE BESIDES THIS STUDY?

Instead of being in this study you have the choice of:

- treatment with prescription drugs available to you
- treatment with experimental drugs, if you qualify
- no treatment
- continue routine medical care from your primary care provider
- joining another trial if you qualify
- not getting medical care

Please talk to your study doctor about these and other choices available to you. Your study doctor will explain the risks and benefits of these choices.

WHAT ABOUT CONFIDENTIALITY?

For US sites: We will do everything we can to protect your privacy. In addition to the efforts of the study staff to help keep your personal information private, we have gotten a Certificate of Confidentiality from the US Federal Government. This certificate means that researchers cannot be forced to tell people who are not connected with this study, such as the court system, about your participation. Also, any publication of this study will not use your name or identify you personally.

People who may review your records include the AIDS Clinical Trials Group (ACTG), Office for Human Research Protections (OHRP) or other government agencies as part of their duties, Food and Drug Administration (FDA) (insert name of site) IRB/EC (a group that protects the rights and well-being of people in research), National Institutes of Health (NIH), study staff, study monitors, the drug company supporting this study, and their designees. Having a Certificate of Confidentiality does not prevent you from releasing information about yourself and your participation in the study.

Even with the Certificate of Confidentiality, if the study staff learns of possible child abuse and/or neglect or a risk of harm to yourself or others, we will be required to tell the proper authorities.

OR

For Non-US sites: Efforts will be made to keep your personal information confidential. We cannot guarantee absolute confidentiality. Your personal information may be disclosed if required by law. Any publication of this study will not use your name or identify you personally.

Your records may be reviewed by the ACTG, OHRP, FDA, (insert name of site) IRB/EC, National Institutes of Health (NIH), national regulatory/health agencies, study staff, study monitors, and the drug company supporting this study and its designees.

A description of this clinical trial will be available on www.ClinicalTrials.gov, as required by US law. This web site will not include information that can identify you. At most, the web site will include a summary of the results. You can search this web site at any time.

WHAT IF WE CAN NO LONGER REACH YOU DURING YOUR STUDY PARTICIPATION?

In the event you cannot be reached after multiple attempts to contact you, study staff may try to contact you through alternate phone numbers of family, friends, case manager, or acquaintances obtained at screening and updated at each visit. If you are unable to be reached through the alternate contacts we will attempt to obtain information about you from other sources such as family members, other designated contacts, or clinic records. The purpose of obtaining this information is to determine if you have died and the cause of death since last contact.

Contacting Your Health Care Providers

Sites to modify per local requirements for obtaining health care records: With your permission, for which you would need to sign a waiver, study staff may contact your health care providers regarding any clinical diagnoses you may develop during the study, including heart related diagnoses and other diagnoses, such as HIV, kidney, liver or cancer diagnoses. Will you allow us to contact your health care providers regarding these clinical diagnoses?

________ YES ________ NO _______Initials

WHAT ARE THE COSTS TO ME?

There will be no cost to you for the study drugs, the study visits, physical examinations, laboratory tests or other tests required by the study. You or your insurance company, or your health care system will be responsible for the costs of your regular medical care as well as for the costs of drugs not given by the study.

Taking part in this study may lead to added costs to you and your insurance company. In some cases it is possible that your insurance company will not pay for these costs because you are taking part in a research study.

WILL I RECEIVE ANY PAYMENT?

You will be paid ________ at the entry visit, the month 1 visit, and annual visits thereafter for participation in the study. *(The team recommends compensation to participants of $25 at the entry visit, month 1, month 12, month 24, month 36, month 48*, *month 6, and month 72). Sites will be reimbursed for the expense.)*

WHAT HAPPENS IF I AM INJURED?

If you are injured as a result of being in this study, you will be given immediate treatment for your injuries. The cost for this treatment will be charged to you or your insurance company. There is no program for compensation either through this institution or the National Institutes of Health. You will not be giving up any of your legal rights by signing this consent form.

WHAT ARE MY RIGHTS AS A RESEARCH PARTICIPANT?

Taking part in this study is completely voluntary. You may choose not to take part in this study or leave this study at any time. Your decision will not have any impact on your participation in other studies conducted by NIH and will not result in any penalty or loss of benefits to which you are otherwise entitled.

We will tell you about new information from this or other studies that may affect your health, welfare, or willingness to stay in this study. If you want the results of the study, let the study staff know.

WHAT DO I DO IF I HAVE QUESTIONS OR PROBLEMS?

For questions about this study or a research-related injury, contact:

- name of the investigator or other study staff
- telephone number of above

For questions about your rights as a research participant, contact:

- name or title of person on the Institutional Review Board (IRB/EC) or other organization appropriate for the site
- telephone number of above

SIGNATURE PAGE

If you have read this consent form (or had it explained to you), all your questions have been answered and you agree to take part in this study, please sign your name below.

______________________ Subject's Name (print)	______________________ Subject's Signature and Date
______________________ Subject's Legal Representative (print) (As appropriate)	______________________ Legal Representative's Signature and Date
______________________ Study Staff Conducting Consent Discussion (print)	______________________ Study Staff's Signature and Date
______________________ Witness's Name (print) (As appropriate)	______________________ Witness's Signature and Date

APPENDIX II: THE MECHANISTIC SUBSTUDY OF REPRIEVE (A5333s)

Effects of Pitavastatin on Coronary Artery Disease and Inflammatory Biomarkers: Mechanistic Substudy of REPRIEVE

Principal Investigators: Steven Grinspoon, MD
Pamela Douglas, MD
Udo Hoffmann, MD, MPH
Heather Ribaudo, PhD

AIDS Clinical Trials Group Investigators: Turner Overton, MD
Carl Fichtenbaum, MD
Judith Aberg, MD
Markella Zanni, MD

Data Coordinating Center: Harvard School of Public Health

Study Funders: National Heart, Lung, and Blood Institute
National Institute of Allergy and Infectious Diseases

Industry Support: Kowa Pharmaceuticals America

NHLBI Program and Medical Officer: Monica R. Shah, MD

DAIDS Medical Officer: Karin L. Klingman, MD

IND Sponsor: Division of AIDS, NIAID, NIH

IND Number: 119127

FINAL Version 2.0
December 19, 2014

SITES PARTICIPATING IN THE MECHANISTIC SUBSTUDY of REPRIEVE (A5333s)

The Mechanistic Substudy of REPRIEVE (A5333s) is open to selected sites that are participating in the REPRIEVE (A5332) study. A list of these sites can be found on the Mechanistic Substudy (A5333s) PSWP.

EXECUTIVE SUMMARY

Title	Effects of Pitavastatin on Coronary Artery Disease and Inflammatory Biomarkers: Mechanistic Substudy of REPRIEVE (A5333s)
Indication	Assess the effects of statins on critical plaque and inflammatory characteristics to understand mechanism of action in HIV
Location	Selected sites of the REPRIEVE (A5332) study
Brief Rationale	The Mechanistic Substudy of REPRIEVE (A5333s) will determine, among HIV-infected persons, potential statin effects to halt progression of non-calcified atherosclerotic plaque and to stabilize morphologic features of plaque vulnerability. Moreover, the study will identify biological factors mediating these changes – be it lipid parameters, such as LDL cholesterol, or markers of inflammation and immune activation.
Study Design and Duration	Randomized, placebo-controlled multicenter substudy of REPRIEVE (A5332) in 800 subjects, with individual subjects participating in the substudy for 2 years.
Treatment	Pitavastatin 4 mg PO daily or placebo for pitavastatin.
Primary Objective	To determine the effects of pitavastatin on the morphology and composition of non-calcified coronary atherosclerotic plaque (NCP), including the progression of plaque volume and whether these effects are modulated by markers of inflammation and immune activation.
Key Secondary Objectives	1. The effects of pitavastatin on the progression of high risk plaque features including low attenuation plaque and positive remodeling.<br>2. The effects of pitavastatin on detailed markers of immune activation, immune activation, inflammation, coagulation, and traditional CVD risk indices including detailed parameters of glucose homeostasis (insulin, glucose and related indices of insulin resistance such as HOMA-IR, HgbA1c).<br>3. The relative contributions of baseline and pitavastatin induced changes in HIV-specific immune activation and traditional risk factors, including LDL, on the presence and progression of coronary plaque and high risk morphological features in HIV.
Primary Endpoint	Noncalcified plaque volume on coronary computed tomography angiography (CCTA)
Secondary Endpoints	1. High risk plaque features on CCTA<br>2. Detailed immune phenotyping measures and inflammatory and coagulation indices as well as detailed measures of glucose homeostasis
Abbreviated Study Flow	Subjects will be co-enrolled at specific sites participating in the Mechanistic Substudy of REPRIEVE (A5333s). Enrollment and treatment will be identical as in REPRIEVE (A5332), but endpoints will be collected over 2 year study duration for each enrolled patient.

1.0 HYPOTHESIS AND STUDY OBJECTIVES

1.1 Primary Mechanistic Hypothesis

Statin therapy will reduce progression of non-calcified coronary atherosclerotic plaque volume over two years as measured by serial coronary computed tomography angiography (CCTA) as compared to placebo in HIV-infected patients on ART in whom traditional CVD risk is not significantly increased. The mechanisms underlying the effect of statins will include a) reduction in non-calcified coronary atherosclerotic plaque, b) reduction in vulnerability features of non-calcified coronary atherosclerotic plaque, and c) improvement in critical indices of immune activation and inflammation.

1.2 Secondary Mechanistic Hypotheses

1.2.1 Decreases in LDL cholesterol levels associated with statin therapy will be predictive of improvement in non-calcified coronary atherosclerotic plaque burden and/or vulnerability features.

1.2.2 Statin therapy will reduce indices of general inflammation, coagulation, monocyte activation, and arterial inflammation.

1.2.3 Statin therapy will reduce levels of pro-inflammatory monocyte populations.

1.2.4 Statin therapy will reduce levels of T-cell activation and exhaustion.

1.2.5 Changes in levels of immune activation and inflammatory markers will be associated with changes in morphology and composition of non-calcified coronary atherosclerotic plaque.

1.2.6 Statin therapy will not have a clinically significant effect on glucose and insulin resistance.

1.3 Primary Mechanistic Objective

To determine the effects of pitavastatin on the morphology and composition of non-calcified coronary atherosclerotic plaque (NCP), including the progression of plaque volume and whether these effects are modulated by markers of inflammation and immune activation.

1.4 Secondary Mechanistic Objectives

1.4.1 To determine the effects of pitavastatin on the progression of high risk plaque features including low attenuation plaque and positive remodeling.

1.4.2 To determine the effects of pitavastatin on detailed markers of immune activation, including immune function (CD4, viral load), immune activation (%CD14+CD16+ monocytes, sCD163, sCD14, MCP-1 and T-cell markers), inflammation (Lp-PLA2, hsCRP, IL-6), coagulation (D-Dimer and tissue factor) and traditional CVD risk indices including detailed parameters of glucose homeostasis (insulin, glucose and related indices of insulin resistance such as HOMA-IR, HgbA1c).

1.4.3 To determine the relative contributions of baseline and pitavastatin induced changes in HIV-specific immune activation and traditional risk factors, including LDL, on the presence and progression of coronary plaque and high risk morphological features in HIV.

1.4.4 To collect blood to enable the evaluation of the relationship of host genetics to study endpoints in subsequent ancillary studies.

2.0 INTRODUCTION

2.1 Background

HIV-infected persons face a 1.5 to 2 fold risk of myocardial infarction (MI), even after controlling for increased prevalence of traditional CVD risk factors [Triant 2007; Freiberg 2013]. This increased risk is thought to be mediated, in large part, by immune activation and inflammation as emerging major contributors to the markedly increased prevalence of high risk coronary atherosclerosis in the HIV-infected population [Hsue 2012; Zanni 2012], such that young patients without known CVD and with relatively low Framingham risk scores may still be high-risk. Traditional CVD prevention paradigms tested in the HIV-negative population fail to adequately assess and specifically target HIV-associated immune-mediated CVD risk. The proposed randomized trial of statin therapy in HIV-infected individuals with minimal traditional cardiovascular risk leverages the LDL-lowering [Silverberg 2009] and immunomodulatory properties of statins [Kwak 2000], targeting both traditional CVD risk factors (dyslipidemia), and HIV-specific immune activation. Embedded in the larger events trial is a mechanistic sub-study in which co-enrolled participants will undergo coronary CT angiography for determination of atherosclerotic plaque morphology and composition, as well as detailed profiling of lipid and inflammatory/immune parameters. The mechanistic sub-study will provide several key scientific insights of crucial relevance to predicting and preventing CVD in HIV. Data from the mechanistic sub-study will highlight whether CCTA-based plaque morphology relates to CVD events in HIV, independent of lipid and inflammatory/immune parameters, and whether potential effects of statins to stabilize atherosclerotic plaque morphology – rendering plaques less likely to rupture and cause acute myocardial infarction – are mediated primarily through lipid-lowering or through immunomodulatory effects.

2.1.1 HIV-infected patients have a novel phenotype of atherosclerotic plaque linked to immune activation

HIV-infected individuals without known CVD have been shown to have more non-calcified coronary atherosclerotic plaque relative to HIV-negative controls matched on traditional cardiovascular risk factors [Burdo 2011]. The significance of non-calcified plaque – namely, the higher likelihood it has to rupture, relative to calcified plaque – is highlighted by the MACE trial of over 5000 HIV-negative outpatients. This study showed that over 3 years of follow-up, 22.7% of patients with non-calcified plaque experienced a major adverse cardiac event compared with 5.5% of patients with calcified plaque [Hou 2012]. HIV-infected individuals without known CVD also have more vulnerable atherosclerotic plaque on coronary CTA relative to HIV-negative controls matched on traditional CVD risk factors. Features used to characterize vulnerability include low CT attenuation (correlating with necrotic lipid core) and positive remodeling (reflecting eccentric plaque extension) [Zanni 2013]. Among HIV-negative individuals with suspected CVD, low attenuation and positively remodeling have been demonstrated to

prospectively predict the development of acute coronary syndrome (ACS). In a large study of over 1000 patients, Motoyama et al. demonstrated that in patients without significant CAD the occurrence of either low CT attenuation or positive remodeling was accompanied by a 22 fold increase in relative risk for suffering from an adverse cardiovascular event [Motoyama 2009]. These results were confirmed in another study that demonstrated that those persons with atherosclerotic plaques characterized by two vulnerability features were far more likely than individuals with atherosclerotic plaques absent of vulnerability features to develop ACS (22.2% vs. 0.5%, respectively) [Kitagawa 2009; Motoyama 2009]. Importantly, non-calcified and vulnerable plaque in HIV-infected individuals relates to levels of soluble CD163, a monocyte/macrophage activation marker [Burdo 2011; Zanni 2013]. This latter finding suggests that immune activation in HIV may be contributing to the development of a novel atherosclerotic plaque phenotype which is prone to rupture and result in acute MI. Numerous other studies have linked immune activation markers – including monocyte activation markers LPS, sCD14, sMCP-1, and sCD163, as well as T-cell activation markers – to subclinical atherosclerosis, cardiovascular events, and mortality [Merlini 2012; Kelesidis 2012; Sandler 2011; Kaplan 2011; Liu 1997; Hunt 2011; Giorgi 1999; Burdo 2011; Zanni 2013; Subramanian 2012].

2.1.2 Statin Effects

2.1.2.1 Statins lower LDL cholesterol and dampen immune activation

In the general population, statins have long been known to potently reduce LDL cholesterol and to prevent CVD events [Sacks 1996; Shepherd 1995; Downs 1998]. In addition, statins have pleiotropic anti-inflammatory and immunomodulatory characteristics, which may also contribute to cardio-protective effects. Indeed, *in vitro,* animal, and human studies have shown that statins decrease monocyte activation – reflected in a) decreased monocyte chemotaxis and endothelial adhesion [Montecucco 2009; Fujino 2006; Han 2005] b) reduced monocyte uptake of oxidized LDL cholesterol [Han 2004]), and c) decreased monocyte secretion of cytokines/chemokines and matrix metalloproteinases [Guo 2009; Waehre 2003]. Moreover, statins decrease T-cell activation [Kwak 2000; Singh 2009; Bu 2010] while recruiting regulatory T cells [Mira 2008], and statins also suppress endothelial cell activation [Zheng 2013; Romano 2000; Mulhaupt 2003; Veillard 2006; Zineh 2006]. In HIV-infected persons, statins, and specifically pitavastatin, effectively lower LDL cholesterol [Silverberg 2009; Sponseller 2013; Aberg 2013]. In HIV-infected persons, statins also exert immunomodulatory effects to 1) decrease monocyte activation – reflected in decreased circulating levels of sCD14 and the macrophage-derived phospholipase, Lp-PLA2 [McComsey 2013] and 2) reduce T-cell activation [Ganesan 2011; De Wit 2011].

2.1.2.2 Statins may stabilize rupture-prone coronary atherosclerotic plaque

Findings from observational CT imaging studies and large randomized IVUS trials demonstrate that statin therapy results in regression of NCP volume and potentially stabilizes coronary atherosclerotic plaque morphology in HIV-negative populations [Shimojima 2012; Inoue 2010; Kodama 2010; Nakamura 2008]. In addition, statins have been demonstrated to reduce atherosclerotic plaque inflammation on cardiac FDG-PET [Takawol 2013], and to reduce non-calcified plaque volume [Burgstahler 2007; Hiro 2009].

2.1.2.3 The effect of statins to stabilize rupture-prone atherosclerotic plaque may be mediated through LDL lowering and/or through the exertion of anti-inflammatory effects

The JUPITER trial was a landmark study in that it showed a significant effect of statin therapy to prevent CVD events among non-HIV patients without known heart disease, with relatively low LDL cholesterol (<130 mg/dl), and with evidence of generalized inflammation (CRP >2 mg/L) [Ridker 2008]. The CVD preventive benefits seen in JUPITER, surprisingly, were found to be proportional to reductions in CRP. This observation suggests that in the general population, anti-inflammatory pleiotropy may contribute significantly to the cardioprotective effects exerted by statins [Ridker 2009]. However, event reduction was also consistent with the known magnitude of expected effect from LDL lowering. Thus, although the JUPITER trial demonstrated a marked reduction in CVD events with statins among patients with relatively low LDL cholesterol but generalized inflammation, the lack of mechanistic assessment precluded determination of how this effect was achieved. In a study of the MESA cohort, a population similar to the JUPITER trial cohort, major adverse cardiovascular events correlated better with high risk coronary atherosclerotic features than inflammatory markers such as hsCRP [Blaha 2011]. This data highlights the importance of performing assessments of plaque volume and morphology to characterize the mechanisms by which statins have their effect. Hence, the currently proposed study will determine, in the HIV-infected population, the effects of statins to prevent CVD events and, importantly, mechanisms through which statins may achieve this effect including a reduction of these high risk morphologic features.

2.2 Rationale

The proposed REPRIEVE (A5332) study is a multi-center, prospective randomized placebo controlled trial testing the effect of statin primary preventive therapy on CVD events in HIV-infected patients on ART without significantly increased traditional cardiovascular disease risk and no prior history of CVD. The trial described in this appendix is a mechanistic substudy embedded in the larger REPRIEVE (A5332) clinical events trial. In the Mechanistic Substudy of REPRIEVE (A5333s), co-enrolled participants will undergo coronary computed tomography angiography (CCTA) for determination of atherosclerotic plaque morphology, as well as detailed profiling of lipid and inflammatory/immune parameters.

The Mechanistic Substudy of REPRIEVE (A5333s) will determine, among HIV-infected persons, potential statin effects to halt progression of non-calcified atherosclerotic plaque and to stabilize morphologic features of plaque vulnerability. Moreover, the study will identify biological factors mediating these changes – be it lipid parameters, such as LDL cholesterol, or markers of inflammation and immune activation. Finally, the study will demonstrate whether presence and morphology of subclinical atherosclerotic plaque at baseline relates to CVD events independently of traditional CVD risk factors and markers of HIV-specific immune activation.

Findings from the study will have implications for predicting CVD risk in HIV-infected individuals, for whom traditional risk prediction paradigms such as those used in the 2013 ACC/AHA guidelines may fall short (for failing to factor in the contributions of HIV-specific immune activation) [Stone 2013]. Moreover, findings from the mechanistic substudy will have

implications for the development of targeted CVD preventive strategies in the HIV-infected population: If statins stabilize coronary atherosclerotic plaques and prevent CVD events primarily via LDL lowering, then further LDL lowering strategies may be indicated. On the other hand, if statins stabilize coronary atherosclerotic plaques and prevent CVD events primarily through immunomodulation, then complementary immune-suppressant therapies in HIV-infected patients – such as inhibitors of monocyte activation – will need to be further explored. Overall, the mechanistic sub-study will provide new, critical knowledge about the biology of atherosclerosis in HIV.

3.0 STUDY DESIGN

A5333s is an optional mechanistic substudy of REPRIEVE (A5332). Approximately 800 HIV-infected males and females who are enrolled in REPRIEVE (A5332) will coenroll in the Mechanistic Substudy of REPRIEVE (A5333s). To ensure treatment balance in treatment assignment in the Mechanistic Substudy of REPRIEVE (A5333s), randomization in REPRIEVE (A5332) will be stratified by anticipated substudy participation. Participants will be followed for 24 months in the Mechanistic Substudy of REPRIEVE (A5333s).

4.0 SELECTION AND ENROLLMENT OF SUBJECTS

4.1 Inclusion Criteria

4.1.1 Enrollment in REPRIEVE (A5332).

4.1.2 Willingness to complete procedures required for the study.

4.1.3 Signed informed consent.

4.1.4 Calculated creatinine clearance (CrCl) ≥60 mL/min, as estimated by the Cockcroft-Gault equation as per REPRIEVE (A5332)

NOTE: Results of creatinine must be obtained and creatinine clearance as estimated by the Cockcroft-Gault must be calculated within 14 days prior to CCTA. The estimated CrCl must be >60 mL/min for the subject to proceed with CCTA both at entry and at month 24.

4.2 Exclusion Criteria

4.2.1 Known allergy to iodinated contrast agent.

4.2.2 Currently symptomatic asthma.

4.2.3 Allergy to beta blockers.

4.2.4 Contraindication to beta blockers (ie, taking daily asthma medications).

4.2.5 Positive pregnancy test within 24 hours prior to study entry.

NOTE: Female subjects of reproductive potential (defined as women who have not been post-menopausal for at least 24 consecutive months, ie, who have had menses within 24 months prior to study entry, and women who have not undergone surgical sterilization, specifically hysterectomy or bilateral oophorectomy) must have a negative serum or urine pregnancy test within 24 hours prior to CCTA by any US laboratory that has a CLIA certification or its equivalent, or at any network-approved non-US laboratory that operates in accordance with Good Clinical Laboratory Practices and participates in appropriate external quality assurance programs.

NOTE: Subject reported history is considered acceptable documentation of hysterectomy, bilateral oophorectomy, tubal ligation, tubal micro-inserts, or menopause. Women are considered menopausal if they have not had a menses for at least 12 months and have a FSH (follicle stimulating hormone) of greater than 40 IU/L or, if FSH testing is not available, they have had amenorrhea for 24 consecutive months.

4.2.6 Any condition that prohibits the individual from completing the CCTA.

4.2.7 Body mass index (BMI) ≥40 kg/m^2.

NOTE: Refer to the MOPS for the link to the BMI calculator.

4.2.8 Cardiac arrhythmia at enrollment precluding CCTA; such as atrial fibrillation with heart rate >80 beats per minute or frequent ectopic beats. Please see MOPs for scanner-specific details.

4.3 Enrollment Procedures

The Mechanistic Substudy of REPRIEVE (A5333s) will be limited to select sites participating in REPRIEVE (A5332). Prior to implementation of the substudy, each site must have the protocol and the protocol consent form approved, as appropriate, by their local IRB/EC and any other applicable regulatory entity (RE).

Site inclusion criteria for the Mechanistic Substudy of REPRIEVE (A5333s) will include the following:

- Site participating in REPRIEVE (A5332).
- Site radiology or cardiology department/facility must conduct >1000 CT/per year.
- MD oversight/supervision of CT scans.
- CT scanner must be at least 2nd generation 64-slice.
- The facility must utilize level III readers.
- The radiology/cardiology facility must be capable of all of the following
    - prospective triggering/gating
    - using low KV
    - have capacity to premedicate subjects

Once a candidate for entry has been identified, details will be carefully discussed with the subject. The subject (or when necessary, the legal representative) will be asked to read and sign the approved Mechanistic Substudy of REPRIEVE (A5333s) consent form.

Enrollment into the Mechanistic Substudy of REPRIEVE (A5333s) will occur concurrently with enrollment and randomization into REPRIEVE (A5332).

4.4 Subject Registration

Subjects who meet enrollment criteria will be registered to the substudy according to standard ACTG data management procedures.

5.0 STUDY TREATMENT

No medications are provided by the Mechanistic Substudy of REPRIEVE (A5333s). Study treatment will be distributed and administered as per REPRIEVE (A5332). See section 5.0 of REPRIEVE (A5332) for study treatment requirements and concomitant medications.

6.0 EVALUATIONS

6.1 Schedule of Events for the Mechanistic Substudy of REPRIEVE (A5333s)

Evaluation[1]	Entry	Month 4 ± 14 days	Month 24 ± 28 days	Premature Study Disc. Evaluations[2]
Calculate BMI[3]	X		X	
REAP and DASI Questionnaires			X	X
QOL assessment	X		X	X
Pregnancy Testing	X[4]		X[4]	X
Serum Creatinine	X[5]		X[5]	X
CBC with differential	X		X	X
CD4+/CD8+	X[5]		X[5]	X
Plasma HIV-1 RNA	X[5]		X[5]	X
Fasting Lipid Panel		X[6]		
Fasting Plasma/Serum for Biomarkers – Planned Analysis[7]	X	X	X	X
Cryopreserved PBMCs for Flow Cytometry – Planned Analysis	X		X	X
Whole blood for RNA	X		X	X
CCTA	X		X	X

[1]Subjects in the mechanistic substudy will have all evaluations as per REPRIEVE (A5332), see Table 6.1 REPRIEVE (A5332), additional testing and/or unique processing instructions specific to the mechanistic substudy are listed here in Table 6.1 of the mechanistic substudy.
[2] Premature Study Discontinuation Visits should be performed for the substudy if 6 months have passed since the last visit on REPRIEVE (A5332).
[3]For BMI calculation at entry, use height and weight obtained at screen from REPRIEVE (A5332). For month 24 BMI calculation, use height obtained at screen and weight obtained at month 24 from REPRIEVE (A5332).
[4]Pregnancy testing *must* be performed and reviewed prior to CCTA for mechanistic substudy participants at entry and month 24 to ensure subject eligibility.
[5] CD4+/CD8+, HIV viral load, and serum creatinine at entry and month 24 will be performed as part of substudy. Serum creatinine results *must* be drawn and creatinine clearance as estimated by the Cockcroft-Gault must be reviewed within 14 days prior to CCTA to ensure subject eligibility. If drawn more than 14 days before CCTA, repeat creatinine must be drawn.
[6]Fasting lipid panel from month 4 will be performed centrally and the results will be blinded until the end of the study.
[7] HgbA1c, Lp-PLA2, sCD163, sCD14, MCP-1, IL-6, D-dimer (will not be collected at month 4), hsCRP, troponin, insulin, glucose. Related indices of insulin resistance such as HOMA-IR will be calculated.

6.2 Timing of the Evaluations

6.2.1 Entry

Registration to the Mechanistic Substudy of REPRIEVE (A5333s) will occur at the entry visit and is concurrent with enrollment to the main study. Substudy entry evaluations (excluding CCTA evaluations) must be completed prior to the initiation of REPRIEVE (A5332) study medications. CCTA scan should be completed within 14 days after the initiation of REPRIEVE (A5332) study medications.

6.2.2 Post-entry

Month 4 substudy evaluations must be performed ± 14 days; month 24 substudy evaluations must be performed ± 28 days.

6.2.3 Discontinuation Evaluations

Premature Discontinuation of REPRIEVE (A5332) Study Treatment
Subjects who prematurely discontinue REPRIEVE (A5332) study treatment may continue participation in the substudy with all evaluations performed as per section 6.0.

Premature Discontinuation of REPRIEVE (A5332) Study Participation
Subjects who prematurely discontinue participation in the REPRIEVE (A5332) study will be discontinued from the Mechanistic Substudy of REPRIEVE (A5333s).

Premature Discontinuation of the Mechanistic Substudy of REPRIEVE (A5333s) Participation
Subjects who discontinue participation in the Mechanistic Substudy of REPRIEVE (A5333s) may continue participation in REPRIEVE (A5332).

Subjects who prematurely discontinue participation in the Mechanistic Substudy (A5333s) should complete the Premature Discontinuation of Substudy evaluations at the next scheduled REPRIEVE (A5332) visit.

Subjects who become pregnant while on the Mechanistic Substudy of REPRIEVE (A5333s) must immediately be discontinued from the substudy without any further evaluations performed.

6.3 Instructions for Evaluations

6.3.1 Clinical Assessments

Targeted Physical Exam
A targeted physical examination as described in REPRIEVE (A5332) will be performed as per Table 6.1 and findings will be recorded on the REPRIEVE (A5332) CRFs. Blood pressure and pulse should also be taken. Blood pressure should be recorded on the REPRIEVE (A5332) CRFs at all substudy visits. Record any changes that occur after entry on the REPRIEVE (A5332) CRF.

Calculation for Body Mass Index will be performed prior to entry and month 24 CCTA. See MOPS for link to calculator.

Cardiovascular Risk Factor Assessment
Diet, using the Rapid Eating and Activity Assessment for Patients (REAP) questionnaire and functional capacity using the Duke Activity Status Index (DASI) questionnaire will be performed at the month 24 visit.

Quality of Life Assessment using the SF-36v2 questionnaire will be performed at entry and month 24.

6.3.2 Laboratory Evaluations

Pregnancy Testing
For women with reproductive potential: serum or urine beta-HCG (urine test must have a sensitivity of 15-25 mIU/mL). A negative pregnancy test result must be obtained before the CCTA.

Serum Creatinine
For entry and month 24, serum creatinine will be performed as part of the Mechanistic Substudy of REPRIEVE (A5333s). Serum creatinine must be drawn and creatinine clearance as estimated by the Cockcroft-Gault must be calculated within 14 days prior to CCTA. The estimated CrCl must be >60 mL/min for the subject to proceed with CCTA both at entry and at month 24.

CBC with Differential
For entry and month 24, a CBC with differential will be performed as part of the Mechanistic Substudy of REPRIEVE (A5333s).

CD4+/CD8+ T-cell Counts
CD4+/CD8+ T-cell count and percentage assays at entry and month 24 must be performed as part of the substudy at a CLIA-certified or equivalent laboratory. In addition, on-study CD4+/CD8+ assays must be performed by a laboratory that is certified for protocol testing by the DAIDS Immunology Quality Assurance (IQA).

Plasma HIV-1 RNA
HIV-1 RNA must be performed at entry and month 24 by a laboratory that possesses a CLIA certification or equivalent.

Fasting Lipids
Serum lipid and lipoproteins (total cholesterol, HDL cholesterol, LDL cholesterol, non HDL, triglycerides, particle size, lipid subfractions, oxidized LDL) are collected only for the Mechanistic Substudy of REPRIEVE (A5333s) at month 4. These samples will be batched and stored in the ACTG Specimen Repository for analyses by a central laboratory. Results will be blinded until the end of study.

Fasting insulin
Fasting insulin samples will be batched and stored in the ACTG specimen repository for analyses by a central laboratory. HOMA-IR will be calculated from fasting insulin determined from the Mechanistic Substudy of REPRIEVE (A5333s) and fasting glucose determined from REPRIEVE (A5332).

<u>HgbA1C</u>
Samples for HgbA1C will be batched and stored in the ACTG Specimen Repository for analyses by a central laboratory.

6.3.3 Immunologic and Biomarker Studies

<u>Fasting Plasma/Serum for Biomarkers – Planned Analysis</u>
The following primary biomarker assays will be performed using frozen samples: Lp-PLA2, sCD163, sCD14, MCP-1, IL-6, D-dimer, hsCRP, troponin. These samples will be batched and stored in the ACTG Specimen Repository for analyses by a central core laboratory. Refer to the Laboratory Processing Chart (LPC) for details. Additional biomarker and coagulation marker assays may be performed.

<u>PBMCs for Monocyte and T Lymphocyte Activation – Planned Analysis</u>
PBMCs for pre-specified flow cytometric analysis on monocytes and T lymphocytes will be batched and stored in the ACTG Specimen Repository for analyses by a central core laboratory. Refer to the LPC for details.

<u>Whole blood RNA</u>
Whole blood will be obtained and processed for RNA. Samples will be batched and stored to enable the evaluation of ancillary studies to assess RNA changes.

6.3.4 CT Angiogram and Risks of Radiation Dose

Coronary computed tomography angiography (CCTA) is performed in accordance with best practice standards as delineated in the imaging guidelines of the Society of Cardiovascular Computed Tomography [Abbara 2009] by competent and appropriately credentialed physicians. This includes the optimization of the scan protocol to limit radiation dose. Specific protocols for each CT scanner, manufacturer, and model will be provided and performed in the MOPS. In general, both retrospective and prospective ECG gated protocols are permitted. The Core Lab will conduct an initial quality assurance screen to ensure correct imaging parameters (kVp, mAs, FOV, slice thickness, slice interval, reconstruction kernel). When a data set is received by the CT core lab, quality analysis (QA) will be performed and if accepted, a case acceptance notice will be sent to the site. If the data set does not pass QA the site will be sent a query to resolve the issue. The CRA will determine if the data set requires resubmission or if the correspondence with the site can resolve the query. If the site does not respond to the query the CRA will follow the query escalation plan. When the query is resolved the site will be sent a case acceptance notification and the data set will be moved to the reader work list. Prior to the follow up CCTA, the Core Lab will send the site a reminder including the baseline scan parameters with a request to perform the follow up as closely as possible resembling the baseline protocol. Refer to the Mechanistic Substudy of REPRIEVE (A5333s) MOPS for detailed instructions.

Risks of Radiation Exposure from CT: CT scanning results in a measurable radiation exposure. For most patients participation in this study will be associated with an estimated cumulative radiation exposure of approximately 11 mSv with a maximum dose of about 16 mSv over two years (median radiation exposure per exam: 5.6 mSv, range 3-8 mSv).

By comparison, radiation exposure from a single stress nuclear myocardial perfusion imaging, another test commonly used to detect heart disease, is about 14 mSv. The average annual radiation exposure from natural background sources in the US is between 2 and 3.6 mSv.

The radiation exposure from this study is only 10% of the maximally allowed annual radiation exposure to radiation workers such as radiology technologists, radiologists, or workers in nuclear plants (50 mSv per year). Overall, each CT scan adds a very small theoretical risk of less than 0.05% to the 7% lifetime risk of lung cancer for men and women and to the 12% lifetime risk of breast cancer for women.

Pregnant subjects must not have a CCTA scan.

7.0 TOXICITY MANAGEMENT

See section 7.0 in protocol REPRIEVE (A5332). For specific CCTA related events, these events should be handled as per standard treatment guidelines, eg, for acute contrast related events.

8.0 CRITERIA FOR SUBSTUDY DISCONTINUATION

- Subjects who discontinue participation in REPRIEVE (A5332). Discontinuation of REPRIEVE (A5332) at A5332 study closure does not constitute premature discontinuation.
- The subject refuses further participation.
- The site investigator determines further participation would be detrimental to the subject's health or well-being.
- The subject fails to comply with the Mechanistic Substudy of REPRIEVE (A5333s) requirements, so as to cause harm to self or seriously interfere with the validity of the substudy results.
- The subject becomes pregnant.
- The subject fails to complete the entry CCTA.
- Subject develops impaired renal function defined as confirmed CrCl <60 mL/min using the calculation for the Cockcroft-Gault equation at month 24 visit.
- The subject develops new onset clinically significant asthma.
- The subject develops new onset/previously undiagnosed allergy to IV contrast.
- Body mass index (BMI) is ≥40 kg/m2 at month 24 visit.

9.0 STATISTICAL CONSIDERATION

General Design Considerations

The overarching aim of the Mechanistic Substudy of REPRIEVE (A5333s) is to better understand the modulation of critical features of coronary plaque morphology with statin therapy, including the progression of non-calcified coronary atherosclerotic plaque (NCP) volume in HIV and the biological factors mediating these effects during statin treatment in HIV.

9.1 Endpoints

9.1.1 Primary Endpoints

9.1.1.1 Evidence of non-calcified coronary atherosclerotic plaque (NCP) at study entry and 2 years.

9.1.1.2 Volume of non-calcified coronary atherosclerotic plaque (NCP) at study entry and change in NCP over 2 years (expressed as absolute change and as a percentage of baseline).

9.1.1.3 Progression of NCP where progression will be defined as follows:

a) Among subjects with evidence of NCP at entry, any progression/increase in NCP volume
b) Among subjects without evidence of NCP at entry, incident NCP.

9.1.2 Secondary and Supportive Endpoints

9.1.2.1 Number of segments with NCP

9.1.2.2 Presence and number of each of the following high risk plaque features

- Low Hounsfield Unit attenuation by CT assessment
- Positive remodeling

9.1.2.3 Levels at study entry, 4 months, and 2 years, and changes from study entry to 4 months and 2 years in the following biomarkers

- Markers of HIV-1 disease: CD4 cell count, HIV-1 RNA level (entry to 2 years only)
- Soluble markers of monocyte activation: sCD163, sCD14, MCP-1
- Various monocyte populations including %CD14+CD16+ monocytes (entry to 2 years only)
- Markers of T-cell activation and exhaustion (entry to 2 years only)
- Markers of inflammation: Lp-PLA2, hsCRP, IL6, troponin
- Markers of coagulation: D-Dimer and tissue factor (excluding D-Dimer at month 4; D-Dimer will be available at entry and month 24 only)

9.1.2.4 Fasting lipid fractions (Total, non-HDL, and HDL cholesterol) and LDL:HDL ratio at study entry, month 4 and 2 years.

9.1.2.5 Fasting insulin, HgbA1c, and HOMA-IR at study entry, month 4, and 2 years (excluding HgbA1c at month 4; HgbA1c will be available at entry and month 24 only).

9.1.2.6 Time to the first major cardiovascular events as defined in section 9.2.1 of REPRIEVE (A5332).

9.2 Sample Size

The target sample size for the Mechanistic Substudy of REPRIEVE (A5333s) is 800 participants that will be approximately equally distributed between the study arms.

This sample size was determined to have high power to detect clinically relevant differences between the two study groups both with respect to plaque progression (among those with plaque at study entry) and rates of incident plaque (among those plaque-free at entry). Specifically, the total sample size of 800 subjects will provide 90% power to detect a 6% difference between the study groups in the percent change in NCP volume over 2 years among those with plaque at entry and 90% power to detect 13 percentage point difference in the probability of plaque development over 2 years. These effect sizes translate to a combined estimated 14 percentage point difference in the probability of NCP progression over two years and are based on the following assumptions:

- 50% of study participants will have evidence of NCP at study entry [Lo 2010]
- A SD of 20% for the percent change over 2 years among participants with evidence of plaque at entry
- An annual rate of incident plaque development of 12% among participants without plaque at entry
- 15% of participants entering the substudy will not be evaluable for study entry or 2 year NCP volume.

Together, the effects of statins acting in these two groups (those with and without evidence of NCP at study entry) will provide for a 7% lower prevalence of NCP after 2-years of statin treatment. Expectations for NCP prevalence and progression both as a whole and according to whether NCP was present at study entry are illustrated in the table below. These estimates are based on a simulation that further assumed the following:

- Average volume NCP 250mm3 (SD=200) among participants with NCP at entry.
- Average volume NCP 40mm3 (SD=20) at 2 years among participants without NCP at entry without statin treatment.
- Average volume NCP 20mm3 (SD=20) at 2 years among participants without NCP at entry with statin treatment.
- NCP distributions were also assumed to follow a gamma distribution with size and shape parameters determine to provide the desire mean and standard deviations.

Cells constituting the primary targeted group comparisons are shown in bold.

		At study entry			At two 2 years						
		NCP (%)	NCP volume (mm3)		NCP (%)	NCP volume (mm3)		Change in NCP volume (mm3)		Percent change in NCP volume (%)[2]	
			Mean (SD)	[P5, P95]		Mean (SD)	[P5, P95]	Mean (SD)	[P5, P95]	Mean (SD)	[P5, P95]
Control	Overall[1]	50%	121 (186)	[0, 509]	59%	140 (210)	[0, 573]	19 (51)	[-21, 103]	17% (33%)	[-10%, 100%]
	No plaque at entry		-	-	21%	9 (19)	[0, 51]	9 (19)	[0, 51]	21% (41%)	[0%, 100%]
	No plaque at 2 years					-	-	-	-	-	-
	Plaque at 2 years					40 (20)	[17, 79]	40 (20)	[17, 79]	100% (0%)	[100%, 100%]
	Plaque at entry		251 (198)	[26, 637]		281 (231)	[28, 730]	30 (68)	[-38, 153]	12% (20%)	[-14%, 49%]
Statin	Overall[1]	50%	120 (185)	[0, 507]	52%	127 (198)	[0, 536]	7 (45)	[-42, 76]	7% (24%)	[-17%, 60%]
	No plaque at entry		-	-	8%	2 (8)	[0, 14]	2 (8)	[0, 14]	8% (27%)	[0%, 100%]
	No plaque at 2 years					-	-	-	-	-	-
	Plaque at 2 years					23 (19)	[2,60]	23 (19)	[2,60]	100% (0%)	[100%, 100%]
	Plaque at entry		250 (197)	[26, 633]		262 (216)	[26, 682]	12 (64)	[-66, 123]	5% (20%)	[-21%, 43%]

[1] In estimation of overall means, participants without evidence of NCP are assigned a value of 0 for volume and change.
[2] In estimation of percentage change, participants without evidence of NCP at study entry with NCP at 2 years are assigned a value of 100%.

These desired statin effects on NCP volume are similar to those seen in intravascular ultrasound (IVUS) studies [Nissen 2006; Nicholls 2005]. Although no data exists to directly inform the clinical relevance of these differences, randomized comparisons of high vs. low dose statin therapy among non HIV-infected patients have demonstrated favorable effects on

atherosclerotic plaque of a similar magnitude as well as on adverse cardiac events, among non HIV patients [Nissen 2006]. Further, data from the "Coronary CT Angiography Evaluation for Clinical Outcomes (CONFIRM) registry suggest that the presence of non-obstructive CAD is predictive major adverse cardiac events independent of traditional risk factors, degree of stenosis and coronary artery calcification (HR 2-5) [Hulton 2013]. The proposed mechanistic study will further add to this body of evidence investigating, for the first time in HIV-infected population, the association of NCP (both the presence and magnitude) as well as changes in volume relate to events. For this exploratory analysis, a larger sample size will be beneficial, in order to accrue CVD events to relate to plaque morphology.

9.3 Randomization/Registration

Participants will be enrolled into the Mechanistic Substudy of REPRIEVE (A5333s) via the ACTG enrollment system at the same time as they are randomized into REPRIEVE (A5332). To ensure balanced treatment allocation in the mechanistic study, REPRIEVE (A5332) randomization will be stratified by planned mechanistic substudy participation.

9.4 Monitoring

Ongoing monitoring of accumulating data for the Mechanistic Substudy of REPRIEVE (A5333s) by the study team (pooled by treatment group) will occur for study conduct and data completeness. Since scans will be read and biomarkers tested in batch at the end of the study, data completeness focus will be for tracking of scan and specimen completeness.

Such issues will be monitored by the jointly appointed NIAID/NHLBI DSMB at the time of DSMB review of REPRIEVE (A5332).

9.5 Analysis Plan

9.5.1 General analysis considerations

The presence and extent of coronary artery disease (CAD) in this population will be described including non-obstructive or obstructive CAD, volume of NCP and calcified plaque, and Agatston score, as well as the prevalence of high risk plaque features such as positive remodeling and low CT attenuation, and levels of inflammatory, and immunomodulatory biomarkers. 95% confidence intervals will be provided for CAD characteristic for the mechanistic study subgroup as whole and by randomization.

All treatment group comparisons will be performed ITT using a 5% type error. Unless otherwise noted, analyses will be performed by subgroups defined according to the presence of NCP at study entry. Since CCTA will not be available at the time of randomization, balance by treatment arm cannot be guaranteed within these subgroups, but given the large sample size and stratification according to sex and HIV-1 disease severity relative balance between the group sample sizes is expected.

9.5.2 Analysis plan

<u>Statin effects on coronary plaque morphology</u>: Among participants with plaque at entry, descriptive statistics for the change and percentage change in NCP volume over 2 years will be provided by treatment group with group comparisons made with stratified t-test. Among those without NCP at entry the prevalence of incident NCP over 2 years will be

compared with stratified chi-squared test. To assess the mechanistic study population as a whole patients will be classified as progressors (any progression/increase in NCP volume OR incident NCP) or non-progressors (no progression in NCP volume OR no incident NCP); the probability of progression over two years will be compared by treatment group using a stratified chi-squared test.

The statin effect on high risk plaque features, including low HU attenuation and positive remodeling, will be assessed by comparing differences in the 2 year prevalence of high risk plaque morphology features between treatment groups using chi-squared (or Fisher's exact) tests as appropriate; these analyses will be performed overall and by subgroups defined by the presence of NCP at study entry. Exploratory analysis will be performed for additional high risk plaque features that have been described in CCTA and IVUS studies including the Napkin Ring Sign, Minimal luminal area, plaque burden, and segments with NCP. The analytic approach will be similar as described above.

Statin effects on blood biomarkers: Statin effects on the distributions of blood biomarkers belonging to distinct pathways (ie, monocyte activation, generalized inflammation, and coagulation) will be assessed via treatment group comparisons of these respective markers via t-tests; modification of statin effect on these markers by HIV-1 and traditional risk factors (including sex, age, screening CD4, duration of suppressive ART, and presence of NCP at study entry). Since the hypothesized mechanism is that sustained high levels of immune activation and inflammation precede and contribute to progression of NCP volume and high risk plaque features, these analyses will relate short term changes in these biomarkers (over 4 months) to longer term changes in NCP volume and morphology after two years.

LDL and blood biomarkers as mediators for plaque progression: In the event that both statin effects on NCP progression and biomarker changes are apparent, the association between changes in LDL and these biomarkers and NCP progression will be examined using graphical techniques and normal errors and logistic regression (for the subpopulations with and without NCP at entry respectively. A mediating effect of these biological factors will be evaluated by examination of changes in the estimated statin effect on NCP upon adjustment for these biological factors. Those biomarkers with the strongest mediating effect on plaque progression will be measured in the entire REPRIEVE (A5332) cohort to determine their association with MACE.

10.0 DATA COLLECTION AND MONITORING AND ADVERSE EVENT REPORTING

Serious adverse events (SAEs) that occur as part of participation in the Mechanistic Substudy (A5333s) are reported as expedited adverse events (EAEs) through REPRIEVE (A5332). These SAEs are reported on a CRF for the Mechanistic Substudy (A5333s); they are not reported on the CRF for REPRIEVE (A5332). There are additional non-serious AEs (ie, non-serious contrast reactions) that may occur as part of the Mechanistic Substudy (A5333s) that will also be recorded on the CRF. Please see the Mechanistic Substudy (A5333s) MOPS for more details regarding AE reporting for A5333s.

11.0 HUMAN SUBJECTS

IRB/EC review, subject confidentiality, and study discontinuation procedures will be the same as in REPRIEVE (A5332). Subjects must sign a separate informed consent form for the Mechanistic Substudy of REPRIEVE (A5333s). Risks, including potential risks of CCTA, and protection against risk are described in the accompanying sample informed consent form.

12.0 PUBLICATION OF RESEARCH FINDINGS

Publication of the results of the Mechanistic Substudy of REPRIEVE (A5333s) will be governed by NIH policies.

13.0 BIOHAZARD CONTAINMENT

Precautions and procedures will be as in REPRIEVE (A5332).

14.0 REFERENCES

Abbara S, et al. SCCT guidelines for performance of coronary computed tomographic angiography: a report of the Society of Cardiovascular Computed Tomography Guidelines Committee. J Cardiovasc Comput Tomogr 2009;3(3):190–204.

Aberg JA, et al. Neutral effects of pitavastatin 4 mg and pravastatin 40 mg on blood glucose and HbA1c levels over 12 weeks: prespecified safety analysis from INTREPID (HIV-infected patients and treatment with pitavastatin vs pravastatin for dyslipidemia), a phase 4 trial. Endocrine Society of America 95th Annual Meeting and Expo, 2013, San Francisco, CA.

Blaha MJ, et al. Associations between C-reactive protein, coronary artery calcium, and cardiovascular events: implications for the JUPITER population from MESA, a population-based cohort study. Lancet 2011;378(9792):684-92. PMID: 21856482.

Bu DX, et al. Statin-induced Kruppel-like factor 2 expression in human and mouse T cells reduces inflammatory and pathogenic responses. J Clin Invest 2010;120(6):1961-70.

Burdo TH, et al. Soluble CD163, a novel marker of activated macrophages, is elevated and associated with noncalcified coronary plaque in HIV-infected patients. J Infect Dis 2011;204(8):1227-36. PMID: 21917896.

Burgstahler C, et al. Influence of a lipid-lowering therapy on calcified and noncalcified coronary plaques monitored by multislice detector computed tomography: results of the New Age II Pilot Study. Invest Radiol 2007;42(3):189-95.

De Wit S, et al. Downregulation of CD38 activation markers by atorvastatin in HIV patients with undetectable viral load. AIDS 2011;25(10):1332-3.

Downs JR, et al. Primary prevention of acute coronary events with lovastatin in men and women with average cholesterol levels: results of AFCAPS/TexCAPS. Air Force/Texas Coronary Atherosclerosis Prevention Study. JAMA 1998;279(20):1615-22. PMID: 9613910.

Freiberg MS, et al. HIV infection and the risk of acute myocardial infarction. JAMA Intern Med 2013;173(8):614-22. PMID: 23459863.

Fujino M, et al. Pitavastatin-induced downregulation of CCR2 and CCR5 in monocytes is associated with the arrest of cell-cycle in S phase. Atherosclerosis 2006;187(2):301-8. PMID: 16289073.

Ganesan A, et al. High dose atorvastatin decreases cellular markers of immune activation without affecting HIV-1 RNA levels: results of a double-blind randomized placebo controlled clinical trial. J Infect Dis 2011;203(6):756-64.

Giorgi JV, et al. Shorter survival in advanced human immunodeficiency virus type 1 infection is more closely associated with T lymphocyte activation than with plasma virus burden or virus chemokine coreceptor usage. J Infect Dis 1999;179(4):859-70. PMID: 10068581.

Guo H, et al. Rosuvastatin inhibits MMP-2 expression and limits the progression of atherosclerosis in LDLR-deficient mice. Arch Med Res 2009;40(5):345-51.

Han KH, et al. HMG-CoA reductase inhibition reduces monocyte CC chemokine receptor 2 expression and monocyte chemoattractant protein-1-mediated monocyte recruitment in vivo. Circulation 2005;111(11):1439-47.

Han J, et al. Pitavastatin downregulates expression of the macrophage type B scavenger receptor, CD36. Circulation 2004;109(6):790-6.

Hiro T, et al. Effect of intensive statin therapy on regression of coronary atherosclerosis in patients with acute coronary syndrome: a multicenter randomized trial evaluated by volumetric intravascular ultrasound using pitavastatin versus atorvastatin (JAPAN-ACS [Japan assessment of pitavastatin and atorvastatin in acute coronary syndrome] study). J Am Coll Cardiol 2009;54(4):293-302.

Hou ZH, et al. Prognostic value of coronary CT angiography and calcium score for major adverse cardiac events in outpatients. JACC Cardiovasc Imaging 2012;5(10):990-9. PMID: 23058065.

Hsue PY, et al. Immunologic basis of cardiovascular disease in HIV-infected adults. J Infect Dis 2012;205 Suppl 3:S375-82. PMID: 2577211.

Hulten E, et al. Usefulness of coronary computed tomography angiography to predict mortality and myocardial infarction among Caucasian, African and East Asian ethnicities (from the CONFIRM [Coronary CT Angiography Evaluation for Clinical Outcomes: An International Multicenter] Registry). Am J Cardiol 2013;111(4):479-85. PMID: 23211358.

Hunt PW, et al. Impact of CD8+ T-cell activation on CD4+ T-cell recovery and mortality in HIV-infected Ugandans initiating antiretroviral therapy. AIDS 2011;25(17):2123-31. PMID: 21881481.

Inoue K, et al. Serial coronary CT angiography-verified changes in plaque characteristics as an end point: evaluation of effect of statin intervention. JACC Cardiovasc Imaging 2010;3(7):691-8.

Kaplan RC, et al. T cell activation and senescence predict subclinical carotid artery disease in HIV-infected women. J Infect Dis 2011;203(4):452-63. PMID: 21220772.

Kelesidis T, et al. Biomarkers of microbial translocation and macrophage activation: association with progression of subclinical atherosclerosis in HIV-1 infection. J Infect Dis 2012;206(10):1558-67. PMID: 23066162.

Kitagawa T, et al. Characterization of noncalcified coronary plaques and identification of culprit lesions in patients with acute coronary syndrome by 64-slice computed tomography. JACC Cardiovasc Imaging 2009;2(2):153-60. PMID: 19356549.

Kodama K, et al. Stabilization and regression of coronary plaques treated with pitavastatin proven by angioscopy and intravascular ultrasound--the TOGETHAR trial. Circ J 2010;74(9):1922-8.

Kwak B, et al. Statins as a newly recognized type of immunomodulator. Nat Med 2000;6(12):1399-402. PMID: 11100127.

Lo J, et al. Increased epicardial adipose tissue volume in HIV-infected men and relationships to body composition and metabolic parameters. AIDS 2010;24(13):2127-30. PMID: 20588167.

Liu Z, et al. Elevated CD38 antigen expression on CD8+ T cells is a stronger marker for the risk of chronic HIV disease progression to AIDS and death in the Multicenter AIDS Cohort Study than CD4+

cell count, soluble immune activation markers, or combinations of HLA-DR and CD38 expression. J Acquir Immune Defic Syndr Hum Retrovirol 1997;16(2):83-92. PMID: 9358102.

McComsey GA, et al. Effect of statins on immune activation and inflammation in HIV+ subjects on ART: a randomized placebo controlled trial. 20th Conference on Retroviruses and Opportunistic Infections; March 3-6, 2013, Atlanta, GA.

Merlini E, et al. T-cell phenotypes, apoptosis and inflammation in HIV+ patients on virologically effective cART with early atherosclerosis. PLoS One 2012;7(9):e46073. PMID: 23029393.

Mira E, et al. Statins induce regulatory T cell recruitment via a CCL1 dependent pathway. J Immunol 2008;181(5):3524-34.

Montecucco F, et al. Statins inhibit C-reactive protein-induced chemokine secretion, ICAM-1 upregulation and chemotaxis in adherent human monocytes. Rheumatology (Oxford) 2009;48(3):233-42. PMID: 19151033.

Motoyama S, et al. Computed tomographic angiography characteristics of atherosclerotic plaques subsequently resulting in acute coronary syndrome. J Am Coll Cardiol 2009;54(1):49-57. PMID: 19555840.

Mulhaupt F, et al. Statins (HMG-CoA reductase inhibitors) reduce CD40 expression in human vascular cells. Cardiovasc Res 2003;59(3):755-66. PMID: 14499877.

Nakamura T, et al. Rapid stabilization of vulnerable carotid plaque within 1 month of pitavastatin treatment in patients with acute coronary syndrome. J Cardiovasc Pharmacol 2008;51(4):365-71.

Nicholls SJ, et al. Effect of atorvastatin (80 mg/day) versus pravastatin (40 mg/day) on arterial remodeling at coronary branch points (from the REVERSAL study). Am J Cardiol 2005;96(12):1636-9. PMID: 16360349.

Nissen SE, et al. ASTEROID Investigators. Effect of very high-intensity statin therapy on regression of coronary atherosclerosis: the ASTEROID trial. JAMA 2006;295(13):1556-65. PMID: 16533939.

Ridker PM, et al. JUPITER Study Group. Rosuvastatin to prevent vascular events in men and women with elevated C-reactive protein. N Engl J Med 2008;359(21):2195-207. PMID: 18997196.

Ridker PM, et al. Reduction in C-reactive protein and LDL cholesterol and cardiovascular event rates after initiation of rosuvastatin: a prospective study of the JUPITER trial. Lancet 2009;373(9670):1175-82.

Romano M, et al. Inhibition of monocyte chemotactic protein-1 synthesis by statins. Lab Invest 2000;80(7):1095-100. PMID: 10908155.

Sacks FM, et al. The effect of pravastatin on coronary events after myocardial infarction in patients with average cholesterol levels. Cholesterol and Recurrent Events Trial investigators. N Engl J Med 1996;335(14):1001-9. PMID: 8801446.

Sandler NG, et al. INSIGHT SMART Study Group. Plasma levels of soluble CD14 independently predict mortality in HIV infection. J Infect Dis 2011;203(6):780-90. PMID: 21252259.

Shepherd J. The West of Scotland Coronary Prevention Study: a trial of cholesterol reduction in Scottish men. Am J Cardiol 1995;76(9):113C-117C. PMID: 7572679.

Shimojima, M, et al. Rapid changes in plaque composition and morphology after intensive lipid lowering therapy: study with serial coronary CT angiography. Am J Cardiovasc Dis 2012;2(2): 84-8.

Silverberg MJ, et al. Response to newly prescribed lipid-lowering therapy in patients with and without HIV infection. Ann Intern Med 2009;150(5):301-13. PMID: 19258558.

Singh P, et al. Influence of statins on MHC class I expression. Ann N Y Acad Sci 2009;1173:746-51.

Sponseller CA, et al. Pitavastatin 4 mg provides greater LDL-C reduction compared to pravastatin 40 mg over 12 weeks of treatment in HIV-infected adults with dyslipidemia. 20th Conference on Retroviruses and Opportunistic Infections; March 3-6, 2013; Atlanta, GA.

Stone NJ, et al. 2013 ACC/AHA guidelines on the treatment of blood cholesterol to reduce atherosclerotic cardiovascular risk in adults: A Report of the American College of Cardiology/American Heart Association Task Force on Practice Guidelines. J Am Coll Cardiol 2014;63(25 Pt B):2889-934.

Subramanian S, et al. Arterial inflammation in patients with HIV. JAMA 2012;308(4):379-86. PMID: 22820791.

Tawakol A, et al. Intensification of statin therapy results in a rapid reduction in atherosclerotic inflammation: results of a multi-center fluorodeoxyglucose-positron emission tomography/computed tomography feasibility study. J Am Coll Cardiol 2013;62(10):909-17.

Triant VA, et al. Increased acute myocardial infarction rates and cardiovascular risk factors among patients with human immunodeficiency virus disease. J Clin Endocrinol Metab 2007;92(7):2506-12. PMID: 17456578.

Veillard NR, et al. Simvastatin modulates chemokine and chemokine receptor expression by geranylgeranyl isoprenoid pathway in human endothelial cells and macrophages. Atherosclerosis 2006;188(1):51-8. PMID: 16321392.

Waehre T, et al. Hydroxymethylglutaryl coenzyme a reductase inhibitors down-regulate chemokines and chemokine receptors in patients with coronary artery disease. J Am Coll Cardiol 2003;41(9):1460-7.

Zanni MV, et al. HIV-specific immune dysregulation and atherosclerosis. Curr HIV/AIDS Rep 2012;9(3):200-5. PMID: 22638983.

Zanni MV, et al. Increased coronary atherosclerotic plaque vulnerability by coronary computed tomography angiography in HIV-infected men. AIDS 2013;27(8):1263-72. PMID: 23324657.

Zheng C, et al. Statins suppress apolipoprotein CIII-induced vascular endothelial cell activation and monocyte adhesion. Eur Heart J 2013;34(8):615-24.

Zineh I, et al. Modulatory effects of atorvastatin on endothelial cell-derived chemokines, cytokines, and angiogenic factors. Pharmacotherapy 2006;26(3):333-40. PMID: 1650371.

APPENDIX III: SAMPLE INFORMED CONSENT

For The Mechanistic Substudy of REPRIEVE (A5333s), FINAL Version 2.0, 12/19/14

Effects of Pitavastatin on Coronary Artery Disease and Inflammatory Biomarkers: Mechanistic Substudy of REPRIEVE

SHORT TITLE FOR THE STUDY: The REPRIEVE Mechanistic Substudy

INTRODUCTION

You are being asked to take part in this research substudy because you will be taking pitavastatin or the placebo for pitavastatin for REPRIEVE (A5332). This study is sponsored by the National Institutes of Health (NIH). The doctor in charge of this substudy at this site is: (insert name of Principal Investigator). Before you decide if you want to be a part of this substudy, we want you to know about the substudy.

This is a consent form. It gives you information about this substudy. The substudy staff will talk with you about this information. You are free to ask questions about this substudy at any time. If you agree to take part in this substudy, you will be asked to sign this consent form. You will get a copy to keep.

WHY IS THIS SUBSTUDY BEING DONE?

The purpose of this substudy is to learn about the effects of pitavastatin on the vessels that supply your heart with blood “coronary arteries” and the atherosclerotic plaque within the wall of these vessels (known as “hardening of the arteries”), as well as inflammatory biomarkers (blood tests that indicate the body’s immune system is active) among people infected with HIV.

HOW MANY PEOPLE WILL BE IN THIS SUBSTUDY?

About 800 people will take part in this study.

WHAT DO I HAVE TO DO IF I AM IN THIS SUBSTUDY?

If you agree to be in this substudy and sign this consent form, you will be asked to come in for 3 visits. Each visit will last about 2-3 hours and will occur at the same time as your main study visits whenever possible. The visits are at entry (when you join the substudy), month 4, and month 24.

Before all visits for the substudy you should not eat or drink anything, including food, beverages, candy, or gum for 8 hours before your visit. You are encouraged to drink water before your visits. If you are not fasting we will ask you to return to have your blood drawn within 7 days of the study visit.

The procedures described below will be done *in addition* to your participation in the REPRIEVE (A5332) study.

Explanation of study procedures
You will be asked to fill out a questionnaire about your quality of life at entry and month 24.

Study staff will ask you questions about your diet and physical activity at month 24.

For women capable of having children, a pregnancy test will be done immediately before the CT of your heart. This test is required as part of your participation in this study. You will be told the results of the pregnancy test. You must notify the research staff if you are pregnant, think you may be pregnant, or if you are trying to become pregnant. If do become pregnant while on the substudy, you will be taken off the substudy and will not have any more substudy tests.

At entry and month 24 we will check your kidney function, complete blood count (CBC), CD4 T-cell count (how many infection fighting cells are in your blood), and HIV viral load (how much HIV is in your blood). The test to check your kidney function is required as part of your participation in this research study. Approximately 3 teaspoons of blood will be collected at each the entry and month 24 visits for these tests.

You will be told the results of these tests.

You will have about 1-4 tablespoons of blood drawn in addition to the blood drawn for REPRIEVE (A5332) at the entry, month 4 and month 24 visits. This blood will be collected and stored for tests that will be done later on in the study or after the study is over. These tests will measure various substances in your blood related to cholesterol (fat in your blood), blood sugar, metabolic tests (how your body processes food), inflammation, and immune function (how your body reacts to infection). You do not need to agree to store this blood to join the study and you may change your mind about storing your blood at any time. You will not be told of the results of the research done on your blood.

Do you agree to let us store your samples for these tests?

________ YES ________ NO _______Initials

Approximately 1 teaspoon of blood collected will be used to look at genes that may affect your risk for cardiovascular disease. Genetic testing is a laboratory test that looks at differences in people's genes. Your body, like all living things, is made up of cells, and cells contain deoxyribonucleic acid, also known as "DNA." DNA is like a string of information put together in a certain order. Parts of the string make up "genes." For the substudy we will do a test to look at your RNA. RNA is made from DNA and is short for ribonucleic acid. RNA is a genetic material that has a major role in making proteins. Proteins are the building blocks of your body, cells, and organs. Genes contain instructions on how to make your body work and fight disease. The testing in this study will focus on certain RNA's that are known to be related to cardiovascular disease and effects of statins. New RNA's of interest may be identified in the future and may also be looked at. You do not need to agree to store this blood to join the study and you may change your mind about storing your blood at any time. You will not be told of the results of the research done on your blood.

Your body's genetic makeup is unique to you, so there is a risk with genetic research that even with all the security measures in place, someone using your samples or genetic information may still find out which information is yours. However, this risk today is very small, but it may increase with time since science and technology are developing rapidly.

In the event that your genetic information becomes linked to your name, the US federal law called the Genetic Information Nondiscrimination Act (GINA) helps protect you. This law prohibits health insurance companies, group health plans, and most employers from denying services based on your genetic information. However, GINA does not protect against discrimination by companies that sell life insurance, disability insurance, or long-term care insurance.

We would like to use some of the blood we collect to look at your genes (RNA). Do you agree to this genotyping?

________ YES ________ NO _______Initials

If at a later date you change your mind and want your samples destroyed, contact the research staff. There are two ways to withdraw your permission. You could allow researchers to remove all your personal identifiers from your samples, so that they are not linked to you anymore. These samples will then become anonymous. Or, you can ask researchers to destroy your samples, so that they cannot be used for future research. However, in either case, researchers will not be able to destroy samples or information from research that is already underway.

For Non-US sites: Your samples may be shipped and stored outside of your country and may be used by researchers outside of your country.

You will have a computed tomography (CT) scan (“Cat” scan) of your heart at entry and month 24. A CT scan is a special kind of x-ray that takes pictures of the inside of the body using a small amount of radiation. A small amount of dye (intravenous contrast) will be injected into your arm during the CT scan to better see the vessels that supply your heart with blood. If your heart rate is more than 65 beats per minute, we may inject a drug called a beta-blocker into your arm via the intravenous line. A beta-blocker is used to slow down your heart rate. A low heart rate is needed in order to make the best pictures of your heart and coronary arteries. In addition, a drug called nitroglycerin will be given to you by mouth in order to obtain better images of the blood vessels of the heart. We will also check your heart rhythm with an electrocardiogram (ECG). To do this, wires with sticky pads attached will be placed on your chest before the scan.

You will be asked to lie quietly while your body is moved inside a large machine and the x-ray is taken. The CT scan takes about 15 minutes. For women, a pregnancy test will be performed prior to CT scanning. Pregnant women will not be allowed to undergo CT scanning. For all patients, a blood test for kidney function will be performed before the CT scan and patients with abnormal kidney function will not undergo CT scanning.

Because the test results are being used for research only, the results created by this study will not become part of your hospital record unless we discover an unexpected medical problem that must be communicated to the study doctors or your primary care physician. If you are found to have a critical blockage of the vessels supplying your heart with blood or another important non-cardiac abnormality that may affect your health, we will provide the results of the CT to your study doctor. The cost of any additional testing will not be covered by the study.

<u>Other Information</u>

You may withdraw from this substudy at any time and still remain on the main study. If you decide to withdraw from the substudy early (before month 24) or stop taking the study treatment in the main study

or if you decide to withdraw from the main study you will be asked to return to the clinic to have the procedures listed in the table below

Procedure	Stopping the study or the study treatment early
Fasting Blood	X
Blood Collected	X
Pregnancy Testing	X
Computed Tomography of your Heart	X
Quality-of-Life Assessment	X

HOW LONG WILL I BE IN THIS SUBSTUDY?

You will be in this substudy for about 2 years.

WHY WOULD THE DOCTOR TAKE ME OFF THIS STUDY EARLY?

The study doctor may need to take you off the study early without your permission if:

- the doctor thinks it is in your best interest
- the study is cancelled
- you are not able to attend the study visits as required by the study
- you are unable to complete the computed tomography of your heart at the entry visit
- you become pregnant
- you have to stop participating in the main study
- your kidney function becomes abnormal during your study participation
- you develop asthma during your study participation
- you develop an allergy to the contrast dye during your study participation
- your body mass index (a measure of body fat based on your height and weight) is greater or equal to 40

WHAT ARE THE RISKS OF THE SUBSTUDY?

The scanning on CT machines will not cause any physical discomfort other than from having to lie still on the table for the duration of the test.

Risks of Radiation Dose from CT
You will have two CTs, one at entry and one at month 24. CT results in a measurable radiation dose. For most people the dose from each CT will be approximately twice the amount you get from natural background radiation (sun and earth) each year. Your dose may be higher or lower based on your size, your heartbeat, and the CT scanner. To put the total dose from the two CTs in further context, it is similar to the radiation dose from a cardiac imaging stress test (another test commonly used to detect heart disease) and much less (approximately 10%) of the maximum allowed exposure for radiation workers such as medical technologists, radiologists, or nuclear plant technicians.

The lifetime risk of lung cancer for men and women is 7%. The lifetime risk of breast cancer in women is 12%. Each CT scan adds a very small theoretical risk of 0.05%.

Radiation Dose

Cardiac CT scanning results in a measurable radiation dose. In order to minimize risk for participants in this study we have implemented the following measures:

- Exclude subjects <40 years of age
- Exclude pregnant and breast feeding women
- Application of cardiac CT protocols to minimize radiation dose
- Review and approval of these CT protocols by the IRB/EC

Risks of Intravenous Contrast Dye

You will receive intravenous contrast dye as part of the CT scan and there is a small risk (2 out of 1,000) of an allergic reaction. More than 90% of such adverse reactions are very mild and allergic-like (itching, *rash*) and can be effectively treated with available drugs (ie, antihistamine). Severe reactions occur in 2 out of 10,000 and one death occurred in approximately 60,000 contrast dye injections.

Contrast-Induced nephropathy (CIN)

CIN is a kidney injury caused by contrast and is usually reversible. CIN almost always occurs in people who already have abnormal kidney function. To be enrolled in this study you must have normal kidney function, and for this reason CIN is very unlikely (less than 5 out of 1,000).

Risks of IV Needle Placement

- Hemorrhage (bruise at the injection site)
- Infection (catheter related infection) at the injection site (very rare)
- Leaking of contrast agent outside of the vein at the place where the IV is inserted.
- Minor discomfort
- Bleeding
- Infection
- Bruising

Risks of Beta-blockers and Nitroglycerin

Beta-blockers and nitroglycerin are used by millions of Americans and are generally considered safe. These drugs are routinely administered prior to cardiac CT to improve the quality and interpretability of the study.

The risk of beta-blockers includes slow heart rate (bradycardia), low blood pressure (hypotension), and wheezing (bronchospasm). Allergic reactions to beta-blockers are rate. Persons with asthma treated with inhalers should not receive beta-blockers. Study staff will assess this and other reasons for you not to have beta-blockers with you prior to the CT.

The side-effects and risks of nitroglycerin are generally mild and of short duration and include low blood pressure (hypotension), high heart rate and abnormal rhythm (tachyarrhythmia), headache, lightheadedness, and visual disturbance. Persons who take erectile dysfunction medications such as Viagra, Cialis, or Levitra (sildenafil, tadalafil, or vardenafil) will need to stop these drugs at least 5 days prior to receiving nitroglycerin on the day of the cardiac CT scan. Nitroglycerin should not be given to persons with a low blood pressure. Study staff will assess this and other reasons for you not to have nitroglycerin with you prior to the CT.

Other Risks/ Additional Risks of CT Scans
• Discomfort
• Claustrophobia

Risks of Drawing Blood
Having your blood drawn may cause discomfort, bleeding, and bruising where the blood is drawn. Occasionally, there is swelling in the area where the needle enters the body and there is a small risk of infection. There is also a risk of lightheadedness, fainting, and blood clots.

Risks of Fasting
Some people find fasting and not smoking or consuming caffeine to be bothersome. It may make some individuals feel anxious, irritable, or hungry. Patients who are required to take their morning medications with food should wait until after the visit has been completed to take their medications.

Genetic Testing
The results of your genetic tests are for research purposes only and no individual results will be given back to you. The results of the genetics studies will never become a part of your medical record. We will protect your confidentiality to the fullest extent. Blood samples for genetic studies will be identified in a way in order to maintain your confidentiality.

Research study results will not be given to your family members, insurance companies, employers, or third parties without your written permission and approval of the Institutional Review Board at _________.

Additional Risks
The CT scan of your heart is being done to answer research questions, not to examine you medically. This scan is not a substitute for one your doctor would order. If the radiologist thinks that there may be an abnormality in your scan, we will contact you and will help you get medical follow-up for the problem. If you have a primary care doctor, we can contact your doctor, with your permission, and help him or her get the right follow-up for you. It is possible that you could be unnecessarily worried if a problem were suspected, but not actually found.

ARE THERE BENEFITS TO TAKING PART IN THIS SUBSTUDY?

If you take part in this substudy, there may be a direct benefit to you, but no guarantee can be made. It is also possible that you may receive no benefit from being in this study. Information learned from this study may help others who have HIV.

WHAT OTHER CHOICES DO I HAVE BESIDES THIS STUDY?

Instead of being in this study you have the choice of:
- participating in REPRIEVE (A5332) only
- not participating

Please talk to your doctor about these and other choices available to you. Your doctor will explain the risks and benefits of these choices.

WHAT ABOUT CONFIDENTIALITY?

We will do everything we can to protect your privacy. In addition to the efforts of the study staff to help keep your personal information private, we have gotten a Certificate of Confidentiality from the U.S. Federal Government. This certificate means that researchers cannot be forced to tell people who are not connected with this study, such as the court system, about your participation. Also, any publication of this study will not use your name or identify you personally.

People who may review your records include the AIDS Clinical Trials Group (ACTG), OHRP, (insert name of site) IRB/EC, government agencies such as the National Institutes of Health (NIH) and Food and Drug Administration (FDA), study staff, study monitors, the drug company supporting this study, and its designee. Having a Certificate of Confidentiality does not prevent you from releasing information about yourself and your participation in the study.

Even with the Certificate of Confidentiality, if the study staff learns of possible child abuse and/or neglect or a risk of harm to yourself or others, we will be required to tell the proper authorities.

A description of this clinical trial will be available on www.ClinicalTrials.gov. This web site will not include information that can identify you. At most, the web site will include a summary of the results. You can search this Web site at any time.

WHAT IF WE CAN NO LONGER REACH YOU DURING YOUR STUDY PARTICIPATION?

In the event you cannot be reached after multiple attempts to contact you, study staff may try to contact you through alternate phone numbers of family, friends, case manager, or acquaintances obtained at screening and updated at each visit. If you are unable to be reached through the alternate contacts we will attempt to obtain information about you from other sources such as family members, other designated contacts, or clinic records. The purpose of obtaining this information is to determine if you have died and the cause of death since last contact.

WHAT ARE THE COSTS TO ME?

Taking part in this substudy may lead to added costs to you and your insurance company. In some cases it is possible that your insurance company will not pay for these costs because you are taking part in a research study.

WILL I RECEIVE ANY PAYMENT?

You will be paid ________ per visit for participation in the substudy. *(The team recommends compensation to participants of $25 at the entry and the month 24 visits. Sites will be reimbursed for the expense.)*

WHAT HAPPENS IF I AM INJURED?

If you are injured as a result of being in this study, you will be given immediate treatment for your injuries. The cost for this treatment will be charged to you or your insurance company. There is no

program for compensation either through this institution or the National Institutes of Health. You will not be giving up any of your legal rights by signing this consent form.

WHAT ARE MY RIGHTS AS A RESEARCH PARTICIPANT?

Taking part in this study is completely voluntary. You may choose not to take part in this study or leave this study at any time. Your decision will not have any impact on your participation in other studies conducted by NIH and will not result in any penalty or loss of benefits to which you are otherwise entitled.

We will tell you about new information from this or other studies that may affect your health, welfare, or willingness to stay in this study. If you want the results of the study, let the study staff know.

WHAT DO I DO IF I HAVE QUESTIONS OR PROBLEMS?

For questions about this study or a research-related injury, contact:

- name of the investigator or other study staff
- telephone number of above

For questions about your rights as a research participant, contact:

- name or title of person on the Institutional Review Board (IRB/EC) or other organization appropriate for the site
- telephone number of above

OTHER

All other information that is contained in the main study REPRIEVE (A5332) consent that you signed also applies to this substudy consent. A copy of the signed main study consent will be provided for you as a reference at the time you consent to participate in the substudy.

SIGNATURE PAGE

If you have read this consent form (or had it explained to you), all your questions have been answered and you agree to take part in this study, please sign your name below.

_______________________	_______________________
Subject's Name (print)	Subject's Signature and Date
_______________________	_______________________
Participant's Legal Representative (print)<br>(As appropriate)	Legal Representative's Signature and Date
_______________________	_______________________
Study Staff Conducting<br>Consent Discussion (print)	Study Staff's Signature and Date
_______________________	_______________________
Witness's Name (print)<br>(As appropriate)	Witness's Signature and Date

Randomized Trial to Prevent Vascular Events in HIV – REPRIEVE (A5332)

Principal Investigators:	Steven Grinspoon, MD<br>Pamela Douglas, MD<br>Michael Lu, MD, MPH<br>Heather Ribaudo, PhD
AIDS Clinical Trials Group Investigators:	Carlos Malvestutto, MD, MPH<br>Carl Fichtenbaum, MD<br>Judith Aberg, MD<br>Markella Zanni, MD
Data Coordinating Center:	Harvard School of Public Health

Study Funders:	National Heart, Lung, and Blood Institute<br>National Institute of Allergy and Infectious Diseases<br>National Institute of Diabetes and Digestive and Kidney Diseases<br>Office of AIDS Research, National Institutes of Health
Industry Support:	Kowa Pharmaceuticals America<br>Gilead Sciences<br>ViiV Healthcare
NHLBI Program and Medical Officer:	Patrice M. Desvigne-Nickens, MD
DAIDS Medical Officer:	Christine Chiou, MD
IND Sponsor:	Division of AIDS, NIAID, NIH
IND Number:	119127

FINAL Version 6.0
May 16, 2022

ACTG
AIDS CLINICAL TRIALS GROUP

Randomized Trial to Prevent Vascular Events in HIV – REPRIEVE (A5332)

SIGNATURE PAGE

I will conduct the study in accordance with the provisions of this protocol and all applicable protocol-related documents. I agree to conduct this study in compliance with United States (US) Health and Human Service regulations (45 CFR 46); applicable US Food and Drug Administration regulations; standards of the International Conference on Harmonization Guideline for Good Clinical Practice (E6); Institutional Review Board/Ethics Committee determinations; all applicable in-country, state, and local laws and regulations; and other applicable requirements (eg, US National Institutes of Health, Division of AIDS) and institutional policies.

Principal Investigator: ____
Print/Type

Signed: ________________________________ Date: _______________
Name/Title

TABLE OF CONTENTS

Page

SIGNATURE PAGE ... 2

SITES PARTICIPATING IN THE STUDY ... 5

PROTOCOL TEAM ROSTER ... 6

STUDY MANAGEMENT ... 9

GLOSSARY OF PROTOCOL-SPECIFIC TERMS ... 11

EXECUTIVE SUMMARY ... 12

1.0 HYPOTHESES AND STUDY OBJECTIVES ... 14
1.1 Clinical Hypotheses ... 14
1.2 Clinical Objectives ... 14

2.0 INTRODUCTION ... 15
2.1 Background ... 15
2.2 Rationale ... 27

3.0 STUDY DESIGN ... 28

4.0 SELECTION AND ENROLLMENT OF PARTICIPANTS ... 29
4.1 Inclusion Criteria ... 29
4.2 Exclusion Criteria ... 31
4.3 Study Enrollment Procedures ... 33
4.4 Mechanistic Substudy of REPRIEVE (A5333s) Enrollment Procedures ... 34
4.5 Coenrollment Guidelines ... 34
4.6 Retention Procedures ... 35

5.0 STUDY TREATMENT ... 35
5.1 Regimens, Administration, and Duration ... 35
5.2 Study Product Formulation and Preparation ... 36
5.3 Pharmacy: Product Supply, Distribution, and Accountability ... 36
5.4 Concomitant Medications ... 36

6.0 CLINICAL AND LABORATORY EVALUATIONS ... 38
6.1 Schedule of Evaluations ... 38
6.2 Timing of Evaluations ... 44
6.3 Instructions for Evaluations and Data Collection ... 45
6.4 Endpoint Assessments ... 53

7.0 CLINICAL MANAGEMENT ISSUES ... 55
7.1 Toxicity Management ... 56
7.2 Requirement for Precautionary or Prohibited Medications (see PSWP) ... 58
7.3 Pregnancy ... 59
7.4 Unblinding Procedures ... 59

CONTENTS (Cont'd)

Page

8.0 CRITERIA FOR DISCONTINUATION.......60
8.1 Premature and Permanent Treatment Discontinuation.......60
8.2 Premature Study Discontinuation.......60

9.0 STATISTICAL CONSIDERATIONS.......61
9.1 General Design Issues.......61
9.2 Outcome Measures.......62
9.3 Randomization and Stratification.......64
9.4 Sample Size and Accrual.......64
9.5 Monitoring.......68
9.6 Analyses.......71

10.0 PHARMACOLOGY PLAN.......76

11.0 DATA COLLECTION AND MONITORING AND ADVERSE EVENT REPORTING.......76
11.1 Records to Be Kept.......76
11.2 Role of Data Management.......76
11.3 Clinical Site Monitoring and Record Availability.......76
11.4 Expedited Adverse Event Reporting to DAIDS.......77

12.0 PARTICIPANTS.......78
12.1 Institutional Review Board (IRB) Review and Informed Consent.......78
12.2 Participant Confidentiality.......78
12.3 Study Discontinuation.......79
12.4 Women and Minorities.......79

13.0 PUBLICATION OF RESEARCH FINDINGS.......79

14.0 BIOHAZARD CONTAINMENT.......79

15.0 STUDY GOVERNANCE.......79

16.0 REFERENCES.......80

APPENDIX I: SAMPLE INFORMED CONSENT.......91

APPENDIX IA: SAMPLE INFORMED CONSENT ADDENDUM.......106

APPENDIX II: THE MECHANISTIC SUBSTUDY OF REPRIEVE (A5333s).......109

APPENDIX III: SAMPLE INFORMED CONSENT.......133

APPENDIX IV: REPRIEVE OBJECTIVES TO DECIPHER SEX-SPECIFIC MECHANISMS OF CVD RISK AND RISK REDUCTION.......143

APPENDIX V: REPRIEVE ANCILLARY STUDY: EFFECT OF PITAVASTATIN ON KIDNEY FUNCTION IN HIV-INFECTED PERSONS.......150

SITES PARTICIPATING IN THE STUDY

REPRIEVE (A5332) is a multicenter study open to US clinical research sites and select international sites that have been approved for participation by the protocol team. Refer to the Site tab on the protocol-specific Web page on the ACTG Member website for the list of eligible sites.

PROTOCOL TEAM ROSTER

Chair
Steve Grinspoon, MD
Metabolism Unit
Harvard Medical School
55 Fruit Street
Longfellow 5, Room 207
Boston, MA 02114
Phone: 617-724-9109
E-mail: sgrinspoon@partners.org

Co-Chair
Pamela Douglas, MD
Duke University Medical Center
7022 North Pavilion
Durham, NC 27715
Phone: 919-681-2690
E-mail: pamela.douglas@duke.edu

Vice Chairs
Judith Aberg, MD
Icahn School of Medicine at Mount Sinai
One Gustave L. Levy Place, Box 10190
New York, NY 10029
Phone: 212- 241-6741
E-mail: Judith.aberg@mountsinai.org

Carl Fichtenbaum, MD
University of Cincinnati
200 Eden Avenue, Room 3114
Mail Location 405
Cincinnati, OH 45267
Phone: 513-584-6361
E-mail: fichtecj@ucmail.uc.edu

Carlos Malvestutto, MD, MPH
Ohio State University Medical Center
410 West 10th Avenue
Columbus, OH 43205
Phone: 614-366-5405
E-mail: carlos.malvestutto@osumc.edu

Vice Chairs (Cont'd)
Markella V. Zanni, MD
Metabolism Unit
Harvard Medical School,
55 Fruit Street, Longfellow 5, Room 209
Boston, MA 02114
Phone: 617-724-6926
E-mail: mzanni@mgh.harvard.edu

Vice-Chair and Co-Project Manager
Kathleen V. Fitch, MSN
Metabolism Unit
Harvard Medical School
55 Fruit Street
Longfellow 5, Room 207
Boston, MA 02114
Phone: 617-724-8015
E-mail: kfitch@mgh.harvard.edu

Mechanistic Substudy
Michael T. Lu, MD, MPH
MGH **Cardiovascular Imaging Research Center,** Harvard Medical School
165 Cambridge Street, Suite 400
Boston, MA 02114
Phone: 617-726-1255
E-mail: mlu@mgh.harvard.edu

DAIDS Medical Officer
Christine Chiou, MD
HIV Research Branch
TRP, DAIDS, NIAID, NIH
5601 Fishers Lane, Room 9F41
Rockville, MD 20852
Phone: 240-507-9611
E-mail: cchiou@niaid.nih.gov

NHLBI Program and Medical Officer
Patrice M. Desvigne-Nickens, MD
Heart Failure and Arrhythmias Branch
DCS, NHLBI, NIH
6705 Rockledge Drive, Room **312-B2**
Bethesda, MD 20817
Phone: 301-435-0504
E-mail: desvignp@nhlbi.nih.gov

Clinical Trials Specialists
Laura E. Moran, MPH
ACTG Network Coordinating Center
Social & Scientific Systems**, Inc.,**
A DLH Holdings Company
8757 Georgia Avenue, 12th Floor
Silver Spring, MD 20910-3714
Phone: 301-628-3373
E-mail: **laura**.moran@**dlhcorp**.com

Nada Saleh, MS
ACTG Network Coordinating Center
Social & Scientific Systems, Inc.,
A DLH Holdings Company
8757 Georgia Avenue, 12th Floor
Silver Spring, MD 20910-3714
Phone: 301-628-0204
E-mail: nada.saleh@dlhcorp.com

Statisticians
Amy Kantor
Center for Biostatistics in AIDS Research
Harvard School of Public Health
655 Huntington Avenue
Boston, MA 02115
Phone: 617-432-7129
E-mail: akantor@sdac.harvard.edu

Jorge Tomas Leon-Cruz
Center for Biostatistics in AIDS Research
Harvard School of Public Health
655 Huntington Avenue
Boston, MA 02115
Phone: 617-432-7469
E-mail: jleoncru@sdac.harvard.edu

Heather Ribaudo, PhD
Center for Biostatistics in AIDS Research
Harvard School of Public Health
655 Huntington Avenue
Boston, MA 02115
Phone: 617-432-2897
E-mail: ribaudo@sdac.harvard.edu

Statisticians (Cont'd)
Triin Umbleja, MSc
Statistical and Data Analysis Center
Harvard School of Public Health
FXB Building, Room 512A
651 Huntington Avenue
Boston, MA 02115
Phone: 617-432-0118
E-mail: tumbleja@sdac.harvard.edu

Project Manager
Kayla Paradis, BA
Cardiovascular Imaging Research Center
Department of Radiology, Massachusetts General Hospital
165 Cambridge Street, Suite 400
Boston, MA 02114
Phone: 978-870-4535
E-mail: kparadis@mgh.harvard.edu

Data Manager**s**
Mark Byroads
Frontier Science & Technology Research Foundation
4033 Maple Road
Amherst, NY 14226
Phone: 716-834-0900 x7341
E-mail: byroads@frontierscience.org

Kenneth Wood, MA
Frontier Science & Technology Research Foundation
4033 Maple Road
Amherst, NY 14226
Phone: 716-834-0900 x7235
E-mail: wood@**frontierscience.org**

DAIDS Pharmacist
Oladapo Alli
5601 Fishers Lane
Room **9E18**, MSC #**9829**
Rockville, MD 20852
Phone: 240-627-3593
E-mail: oladapo.alli@nih.gov

PROTOCOL TEAM ROSTER (Cont'd)

Investigator
Gerald Bloomfield, MD
Associate Professor of Medicine
Duke University School of Medicine
200 Morris Street
Durham, NC 27701
Phone: 919-668-8702
E-mail: gerald.bloomfield@duke.edu

Field Representative
Erin Elizabeth Hoffman, BS
UNC AIDS CRS
130 Mason Farm Road
Chapel Hill, NC 27599
Phone: 919-843-0720
E-mail: Erin_Hoffman@med.unc.edu

Laboratory Technologist
Francoise Giguel
Division of Infectious Diseases
Partners AIDS Research Center
Massachusetts General Hospital
65 Landsdowne Street, Room 435
Cambridge. MA 02139
Phone: 617-768-8374
E-mail: fgiguel@mgh.harvard.edu

Community Scientific Subcommittee (CSS) Representatives
Karl Shaw, BBA
19140 Ohio Street
Detroit, MI 48221
Phone: 313-861-1979
E-mail: kedshaw@hotmail.com

Kate Starr, BS
Ohio State University CRS
6285 Rygate Drive
Reynoldsburg, OH 43068
Phone: 614-353-2944
E-mail: borloglou@gmail.com

International Program Specialist
Akbar Shahkolahi, PhD
ACTG Network Coordinating Center
Social & Scientific Systems**, Inc., A DLH Holdings Company**
8757 Georgia Avenue, 12th Floor
Silver Spring, MD 20910-3714
Phone: 301-628-3318
E-mail: akbar.shahkolahi@dlhcorp.com

Industry Representatives
Kathy Melbourne, PharmD
Gilead Sciences
333 Lakeside Drive
Foster City, CA 94404
Phone: 401-261-7360
E-mail: kathy.melbourne@gilead.com

James Rooney, MD
Gilead Sciences, 333 Lakeside Drive
Foster City, CA 94404
Phone: 650-522-5708
E-mail: jim.rooney@gilead.com

Craig Sponseller, MD
Kowa Pharmaceuticals America, Inc.
530 Industrial Park Boulevard
Montgomery, AL 36117
Phone: 334-288-1288 x127
E-mail: csponseller@kowapharma.com

Laboratory Data Manager**s**
Frederic Bone
Frontier Science and Technology Research Foundation. 4033 Maple Road
Amherst, NY 14226
Phone: 716-834-0900 x7306
E-mail: bone@frontierscience.org

David Vlieg
Frontier Science and Technology Research Foundation
4033 Maple Road
Amherst, NY 14226
Phone: 716-834-0900 x7333
Email: vlieg@frontierscience.org

STUDY MANAGEMENT

This section provides important instructions on how to attempt to have questions about REPRIEVE (A5332) answered. Following these instructions will help you receive an answer as quickly as possible, generally within 24 hours. Sites are responsible for documenting phone calls made to team members.

Protocol E-mail Group
The protocol logon is the primary vehicle for distributing important information about the study to sites. It is the site's responsibility to add all relevant personnel to this e-mail group as soon as possible. Contact actg.user.support@fstrf.org to be added to the "actg.protA5332" e-mail group and, for sites participating in the mechanistic substudy, to the "actg.prota5333s" substudy e-mail group.

IND (Investigational New Drug) Number or Questions
E-mail Regulatory@tech-res.com.

To Request Study Product Package Inserts and/or Investigator Brochures
E-mail RIC@tech-res.com.

Study Drug Orders
Call the Clinical Research Products Management Center (CRPMC) at 1+ 301-294-0741.

Questions about Study Product, Dose, Supplies, Records, and Returns
E-mail protocol pharmacist Oladapo Alli at PABREPRIEVEPEP@mail.nih.gov.

Protocol Registration Questions
E-mail Protocol@tech-res.com or call 1+ 301-897-1707.

Participant Registration and Randomization Issues and Study Identification Number (SID) Lists
E-mail rando.support@fstrf.org or call 1+ 716-834-0900 x7301.

FSTRF Portal Problems and Data Management Questions
- E-mail actg.support@fstrf.org or call 1+ 716-834-0900 x7302 (US sites) or 1+ 716-834-0900 x7200 (non-US sites).
- For nonclinical questions about randomization/registration, inclusion/exclusion criteria, OpenClinica, electronic case report forms (eCRFs), transfers, and other data management issues, e-mail reprieve.dmc@fstrf.org.

Expedited Adverse Event (EAE) Reporting/Questions
Contact DAIDS through the RSC Safety Office at DAIDSRSCSafetyOffice@tech-res.com or call 1-800-537-9979 or 1+ 301-897-1709; or fax 1-800-275-7619 or 301-897-1710.

Other Questions

- For all other questions about REPRIEVE (A5332), e-mail actg.corea5332@fstrf.org.
- For all questions about the Mechanistic Substudy A5333s, including coronary computed tomography angiography (CCTA), e-mail actg.corea5333s@fstrf.org.

Protocol-Specific Web Page (PSWP)

Additional information about protocol management can be found on these PSWPs:

- A5332: https://member.mis.s-3.net/cms/study/53278/10260
- A5333s: https://member.mis.s-3.net/cms/study/53279/10264

GLOSSARY OF PROTOCOL-SPECIFIC TERMS

ACC	American College of Cardiology
ACTG	AIDS Clinical Trials Group
AHA	American Heart Association
AMI	acute myocardial infarction
ART	antiretroviral therapy
ASCVD	atherosclerotic cardiovascular disease
CAD	coronary artery disease
CCTA	coronary computed tomography angiography
CEC	Clinical Events Committee
CKD	chronic kidney disease
CVD	cardiovascular disease
DAIDS	Division of AIDS
DM	diabetes mellitus
DSMB	Data and Safety Monitoring Board
ECG	electrocardiogram
EDC	electronic data capture system
FDG-PET	fluorodeoxyglucose (FDG)-positron emission tomography (PET)
FIB-4	Fibrosis 4 Score
HDL	high-density lipoprotein
IVUS	intravascular ultrasound
LDL	low-density lipoprotein
MACE	major adverse cardiovascular events
NAC	non-AIDS complications
NCEP	National Cholesterol Education Panel
NHLBI	National Heart, Lung, and Blood Institute
NIAID	National Institute of Allergy and Infectious Diseases
PAB	Pharmaceutical Affairs Branch (DAIDS)
PAD	peripheral arterial disease
PSWP	protocol-specific web page
**PWH**	**people with HIV**
RCT	randomized controlled trial
SAP	Statistical Analysis Plan
TIA	transient ischemic attack

EXECUTIVE SUMMARY

Title	Randomized Trial to Prevent Vascular Events in HIV – REPRIEVE (A5332)
Indication	To study the efficacy of statins to reduce the risk of cardiovascular disease (CVD) in **people with HIV (PWH)**.
Location	Multicenter trial conducted at US trial sites and select international sites
Brief Rationale	**PWH** face an increased risk of CVD morbidity and mortality, yet no preventive strategies for CVD risk reduction have been proven for this population. Among **PWH**, immune activation may contribute in unique ways to atherosclerosis and ensuing cardiovascular events. Statins affect both traditional CVD risk factors (LDL cholesterol) and have pleiotropic effects to reduce inflammation and immune activation. Thus, statins may target the unique mechanisms of cardiovascular disease in HIV.
Study Design and Duration	Prospective, double-blind, randomized, placebo-controlled, multicenter efficacy study in 6500 participants, with individual participants to be followed for up to 72 months. The sample size was increased to approximately 7500 participants with follow-up for up to 84 months per the December 2017 Data and Safety Monitoring Board (DSMB) recommendations. Follow-up was further increased to 96 months, which was endorsed at the December 2018 DSMB meeting**. Follow-up is now planned to continue, up to Month 120, until the study reaches its target of 288 primary MACE endpoints or is otherwise recommended for closure by the DSMB.**
Treatment	Pitavastatin 4 mg PO daily or placebo for pitavastatin.
Primary Objective	To determine the effects of pitavastatin as a primary prevention strategy for major adverse cardiovascular events (MACE) in HIV.
Key Secondary Objectives	1. The effects of pitavastatin on the components of MACE and all-cause mortality.<br>2. The effects of pitavastatin on LDL and non-HDL in relationship to MACE.<br>3. Whether baseline traditional risk factors and time updated HIV-specific immunological risk factors are predictive of MACE and pitavastatin effects on MACE.<br>4. The effects of pitavastatin on the incidence of serious non-CVD events.<br>5. The safety of pitavastatin in the HIV population.
Ancillary Objectives	1. To assess the influence of sex/reproductive aging status on immune activation and statin induced immunomodulation in relation to clinical CVD events.<br>2. To determine the effects of pitavastatin on the incidence of changes in kidney function.
Primary Endpoint	Major adverse cardiovascular events (MACE)
Secondary and Safety Endpoints	Primary components of MACE, all-cause mortality, LDL cholesterol, immune function, non-CVD events (malignancy, end stage liver and kidney disease, AIDS-defining events), and safety endpoints, including diabetes mellitus

Abbreviated Study Flow	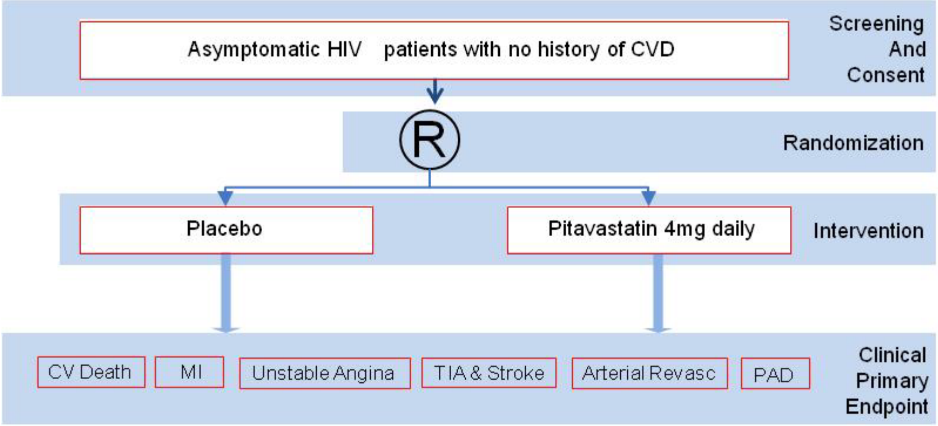

1.0 HYPOTHESES AND STUDY OBJECTIVES

1.1 Clinical Hypotheses

1.1.1 Primary Clinical Hypothesis

Statin therapy will prevent atherosclerotic cardiovascular disease (ASCVD)-related MACE (major adverse cardiovascular events) in **PWH** on antiretroviral therapy (ART) in whom traditional **cardiovascular disease (CVD)** risk is not significantly increased.

1.1.2 Secondary Clinical Hypotheses

1.1.2.1 Statin therapy will be associated with reductions in specific CVD-related events and all-cause mortality.

1.1.2.2 Decreases in LDL and non-HDL cholesterol levels associated with statin therapy will be predictive of reduction in CVD events.

1.1.2.3 Statin therapy will reduce serious non-cardiovascular events, including malignancies, end stage kidney or liver disease.

1.1.2.4 Statin therapy will be safe and well tolerated in PWH.

1.2 Clinical Objectives

1.2.1 Primary Clinical Objective

To determine the effects of pitavastatin as a primary prevention strategy for MACE in HIV.

1.2.2 Secondary Clinical Objectives

1.2.2.1 To evaluate the effects of pitavastatin on each of the components of the primary composite MACE endpoint and all-cause mortality.

1.2.2.2 To determine the effects of pitavastatin on LDL and non-HDL cholesterol in the HIV population and assess the relationship of changes in LDL and non-HDL to the incidence of MACE.

1.2.2.3 To evaluate whether baseline traditional risk factors (including smoking, hypertension, dyslipidemia, glucose) and time updated HIV-specific (immunological and virological) risk factors are predictive of MACE and pitavastatin effects on MACE in the HIV population.

1.2.2.4 To evaluate whether baseline and time updated inflammatory and immune activation biomarkers are predictive of MACE and pitavastatin effects on MACE in the HIV population.

1.2.2.5 To determine the effects of pitavastatin on the incidence of serious non-cardiovascular events and AIDS-defining events.

1.2.2.6 To determine the safety of pitavastatin in the HIV population, including the development of diabetes mellitus (DM), liver dysfunction, and myopathy.

1.2.2.7 To collect blood to enable the evaluation of the relationship of host genetics to study endpoints in subsequent ancillary studies.

1.2.2.8 **Characterize the epidemiology and pathobiology of SARS-CoV-2 infection, particularly with respect to CVD, among individuals aging with HIV globally.**

1.2.2.9 **Identify which host factors influence risk with respect to SARS-CoV-2 infection and COVID-19-related CVD outcomes.**

1.2.2.10 **Determine whether statin therapy protects against SARS-CoV-2 infection and/or mitigates the severity of COVID-19-related CVD complications**.

1.2.3 Ancillary Objectives

1.2.3.1 To assess the influence of sex/reproductive aging status on immune activation and statin induced immunomodulation in relation to clinical CVD events.

1.2.3.2 To determine the effects of pitavastatin on the incidence of changes in kidney function.

2.0 INTRODUCTION

2.1 Background

Over 34 million people worldwide, including 1.7 million people in the US, are chronically infected with HIV-1 [WHO 2012]. Due to the remarkable success of ART, **PWH** are now living longer [Lewden 2007]. By the year 2015, greater than half of the **population living with HIV in the US** is expected to be 50 or older [WHO 2012; Greene 2013]. Yet, even while ART has reduced AIDS-related deaths [Palella 2006], CVD and CVD-related deaths have increased in **PWH,** who have 1.5-2 times increased risk compared with HIV-negative individuals [**Antiretroviral Therapy Cohort Collaboration** 2010; **Data Collection on Adverse Events of Anti-HIV drugs (D:A:D) Study Group** 2010; Sackoff 2006]. Currently, there are no treatment strategies proven to prevent CVD in **PWH**,

despite the higher risk. A State of the Science Conference on CVD in HIV sponsored by the American Heart Association (AHA) highlighted the critical need to test preventive therapies for CVD in HIV and to develop HIV-specific guidelines [Grinspoon 2008]. Experts agree that existing strategies for CVD prevention in the general population are likely inadequate with increasing evidence suggesting HIV-associated immune activation accelerates the process of atherosclerosis and atherothrombosis [Hsue 2012; Zanni 2012] such that relatively young **PWH** with modest traditional CVD risk factor scores still face high CVD risk. Most of these patients would not meet current guidelines for preventive CVD therapies. The imperative to identify a safe and efficacious strategy for CVD prevention in HIV is best met by a randomized controlled trial **(RCT)** of an intervention addressing both traditional and immune CVD risk factors.

2.1.1 Increased Risk of CVD in HIV

CVD-related deaths—including sudden cardiac death—are increased in **PWH** compared with **people without** HIV [**Antiretroviral Therapy Cohort Collaboration** 2010; Tseng 2012]. With respect to acute myocardial infarction (AMI), the most common cause of CVD-related deaths, studies show a 1.5- to 2-fold increased relative risk of this in **PWH** versus **people without HIV** [Currier 2003; Durand 2011; Freiberg 2013; Klein 2002; Lang 2010; Obel 2007; Triant 2007]. Controlling for traditional CVD risk factors does not fully mitigate the heightened CVD risk among **PWH** [Freiberg 2013; Triant 2007], suggesting a unique pathobiology of atherosclerosis in HIV and highlighting the need for an effective, tailored primary CVD prevention strategy.

2.1.2 Unique Biology of CVD in HIV

2.1.2.1 Inadequate Explanation of CVD Risk in HIV by Traditional CVD Risk Factors

Early studies of CVD in HIV suggested that heightened risk stemmed, indirectly, from an effect of ART to exacerbate traditional CVD risk factors (eg, diabetes, hypertension, dyslipidemia, and abdominal fat accumulation). For example, in the Data Collection on Adverse Events of Anti-HIV Drugs (D:A:D) study, use of protease inhibitors was associated with increased AMI risk, and this risk was partially attenuated after controlling for dyslipidemia [Friis-Moller 2003]. In contrast, recent studies demonstrate no independent association of ART with AMI and indicate that higher rates of AMI persist with HIV infection despite controlling for traditional CVD risk factors [Freiberg 2013; Triant 2007]. In large epidemiologic studies, immune dysfunction (low CD4 count) and degree of viremia have been identified as important independent contributors to CVD risk in HIV [Triant 2010]. Moreover, data from the Strategies for Management of Antiretroviral Therapy (SMART) study suggest a protective effect of continuous and intensive ART on CVD risk: In the SMART study, patients were randomized to continuous ART or to an ART conservation strategy based on a CD4-guided algorithm.

CVD occurred 60% more often in the drug conservation group, highlighting a benefit from more continuous and suppressive ART [**Strategies for Management of Antiretroviral Therapy (SMART) Study Group** 2006]. Subsequent studies from the SMART group have focused on specific markers of inflammation and coagulation that are significantly increased with drug conservation, related to viremia, and independently associated with increased CVD rates [Kuller 2008]. Work from the SMART group thus reinforces the principle that chronic viral infections and the subsequent immune response contribute to CVD, independently of traditional risk pathways highlighting the need to consider a possible role for an immune modulating strategy to prevent CVD.

2.1.2.2 Relationship between HIV-Associated Systemic Immune Activation and CVD Risk

HIV infection induces a paradoxical state of both immune suppression (low CD4 count and increased risk of opportunistic diseases) and immune activation [Deeks 2011]. A paucity of CD4+ T regulatory cells in the gut mucosa enables heightened microbial translocation and ensuing activation of both the innate and adaptive arms of the immune system [Brenchley 2006]. Concomitantly, depletion of circulating CD4+ T regulatory cells, coupled with opportunistic co-infection (hepatitis, cytomegalovirus), results in further immune activation. The net result, in **PWH**, is persistent activation of circulating monocytes and T-cell subsets [Deeks 2011]. Such chronic immune activation translates into an exhausted T-cell phenotype and a pro-inflammatory milieu that is manifest by higher circulating levels of soluble inflammatory and immune activation markers in **PWH** versus HIV-negative controls. Elevated inflammatory and immune activation biomarkers in **PWH** include pro-inflammatory cytokines (eg, IL-6), acute phase proteins (eg, CRP), leukocyte adhesion molecules (eg, sICAM-1), and fibrin degradation products (eg, d-dimer) [Dolan 2005; Ross 1999; Ross 2008].

Immune activation in HIV is highly relevant to atherosclerosis [Hsue 2012; Zanni 2012]—an inflammatory disease [Libby 2002] resulting in AMI and sudden cardiac death. Atherosclerosis as a process features an intricate interplay between activated immune cells—particularly monocytes—and vascular endothelial cells [Hulten 2009; Koenen 2010; Libby 2002; Libby 2011; Packard 2009]: Circulating monocytes target the tunica intima of affected coronary arteries in response to chemokines and adhesion molecules produced by activated endothelial cells. There, monocytes transform to macrophages and then, upon internalization of oxidized LDL, to foam cells which form the lipid core of the developing atheroma. Plaque macrophages, along with resident T cells, also secrete cytokines and matrix metalloproteinases, which can

degrade the fibrous cap overlying an atheroma, thus precipitating plaque rupture/AMI [Hulten 2009; Koenen 2010; Libby 2002; Libby 2011; Packard 2009]. With HIV infection—a state of persistently activated circulating monocytes and T cells and increased circulating levels of pro-inflammatory cytokines—all stages of atherogenesis and atherothrombosis are likely exacerbated.

2.1.2.3 Novel Atherosclerotic Phenotype in HIV

Initial studies to radiographically characterize subclinical atherosclerosis in HIV focused on carotid intima media thickness (cIMT) [Hulten 2009], which has been found in this population to relate largely to traditional CVD risk factors and not to inflammatory indices [Stein 2013]. In contrast, studies employing coronary computed tomography angiography (CCTA) and cardiac fluorodeoxyglucose-positron emission tomography (FDG-PET) have shown **PWH** to have 1) predominantly non-calcified coronary atherosclerotic plaque [Burdo 2011], 2) a higher prevalence of high-risk morphology features (including low attenuation and positive remodeling) [Zanni 2013], and 3) vascular inflammation reflected in clustering of glucose-avid macrophages in the subendothelial matrix [Subramanian 2012; Yarasheski 2012]. The clinical relevance of these observations is that inflamed vulnerable plaque which has yet to calcify is more prone to rupture, resulting in AMI [Hou 2012; Kitagawa 2009; Rominger 2009]. Of note, in **PWH**, non-calcified, vulnerable, and inflamed coronary atherosclerotic plaque has been found to relate to specific markers of monocyte activation, including soluble CD163, and has been observed even in those ART-treated **PWH** with low traditional CVD risk factors [Burdo 2011; Subramanian 2012; Zanni 2013].

2.1.3 Rationale for Statin Therapy to Prevent Primary CVD in HIV

An ideal intervention to prevent CVD in HIV would affect both conventional lipid and HIV-specific immune mediators of CVD and would have minimal risk. Statin therapy uniquely meets these criteria.

2.1.3.1 LDL, Immunomodulatory, and Plaque Stabilizing Effects of Statins

Statins, which address both conventional and inflammatory mechanisms for atherosclerosis, may have unique utility for primary CVD prevention in HIV. In the general population, statins have long been known to potently reduce LDL cholesterol and to prevent CVD events [Downs 1998; Sacks 1996; **Scandinavian Simvastatin Survival Study (4S)** 1994; Shepherd 1995]. In addition, statins are known to have pleiotropic anti-inflammatory and immunomodulatory characteristics, which may also contribute to cardio-protective effects [Greenwood 2007]. Indeed, in vitro, animal, and human studies have shown that statins decrease monocyte activation - reflected in a) decreased monocyte chemotaxis

and endothelial adhesion [Fujino 2006; Han 2005; Montecucco 2009], b) reduced monocyte uptake of oxidized LDL cholesterol [Han 2004], and c) decreased monocyte secretion of cytokines/chemokines and matrix metalloproteinases [Guo 2009; Waehre 2003]. Moreover, statins decrease T-cell activation [Bu 2010; Kwak 2000; Singh 2009] while recruiting regulatory T cells [Mira 2008], suppress endothelial cell activation [Mulhaupt 2003; Romano 2000; Veillard 2006; Zheng 2013; Zineh 2006], and decrease lipid oxidation [Aviram 1992; Giroux 1993; Vasankari 2001]. Further, data from cardiovascular imaging studies in HIV-negative participants reveal that statins stabilize vulnerable coronary atherosclerotic plaque and even induce plaque regression. Specifically, statins have been shown to decrease atherosclerotic plaque vulnerability features on CCTA and intravascular ultrasound (IVUS) [Inoue 2010; Kodama 2010; Nakamura 2008; Shimojima 2012], to reduce atherosclerotic plaque inflammation on cardiac FDG-PET [Tawakol 2013], and to reduce non-calcified plaque volume [Burgstahler 2007; Hiro 2009]. Based on these data, statins may be uniquely tailored to address important mechanisms of CVD in HIV.

2.1.3.2 Statin Effects on Non-CVD Events in HIV

Due to the efficacy and widespread use of potent combination ART, mortality patterns among HIV patients have changed, with a decline in the proportion due to AIDS and a concomitant rise in the proportion due to non-AIDS-related diseases [ARTCC 2010; DAD 2010; French 2009; Lewden 2008; Marin 2009; Neuhaus 2010; Palella 2006; Wada 2013; Weber 2013]. In this regard, cardiovascular, end-stage liver and renal disease, and non-AIDS-related malignancies represent important causes of mortality among HIV patients in the current era of potent ART. Based on animal models and in-vitro and in-vivo data that statins may decrease the systemic inflammation that has been associated with many of these events, and epidemiologic data suggesting statins alter the risk of these events, including these key comorbidities in a secondary endpoint analysis of REPRIEVE (A5332) is warranted.

Beyond an excellent safety record in persons with liver disease, current data suggest that statins may improve various aspects of liver disease. In animal models of non-alcoholic fatty liver disease, several different statins, including pitavastatin, have improved hepatic steatosis and decreased fibrosis mediated by anti-inflammatory and antifibrotic effects [Hyogo 2012; Miyaki 2011; Tarantino 2012; Wang 2013]. Human trials have demonstrated similar improvements in steatosis and liver fibrosis [Ekstedt 2007; Foster 2011; Gomez-Dominguez 2006; Simon 2015].

Statins appear to have a beneficial effect on kidney function, particularly among persons with pre-existing microalbuminuria, a common finding among **PWH** [Amarenco 2014; Colhoun 2009]. Meta-analyses have

demonstrated either a modest benefit or uncertain effects of statins on renal function [Douglas 2006; Nikolic 2013; Palmer 2014]. The limited data in HIV patients indicated improvement in renal function with rosuvastatin that correlated with reductions in inflammatory biomarkers and T-cell activation [Longenecker 2014].

HIV infection remains associated with an increased risk of cancer [Crum-Cianflone 2009; Deeken 2012]. While there are numerous factors that contribute to the excess risk of cancer, persistent inflammation and alterations in the immune system, particularly altering normal tumor surveillance, are considered key contributors to the excess risk, even in the setting of viral suppression and partial immunologic reconstitution experienced with ART [Borges 2014; Tenorio 2014]. Statins have been demonstrated to have anticancer activity which have been related to consequences of blocking the mevalonate pathway, including arresting cell cycle progression, inducing apoptosis, reducing oxidative stress, and decreasing systemic inflammation [Carlberg 1996; Wong 2002; Yasui 2007]. While the data from observational studies and post-hoc analyses for cancers in the general population have failed to consistently demonstrate an anticancer effect for statins, data from studies including **PWH**, while limited, suggest potential anticancer effects of statins due to the fact that persistent inflammation is likely an important driver [Galli 2014; Overton 2013].

Despite a declining incidence, AIDS events continue to occur and remain the leading cause of death among **PWH**. Clearly, advanced immunosuppression and a maladaptive inflammatory response, both related to uncontrolled HIV infection, are important drivers of the underlying mechanisms of AIDS events. While there are limited data regarding the impact of statins on the risk of developing AIDS events, the effects of statins on markers of both cellular and soluble markers of inflammation indicate that there may be benefit to assess the effect of statins on AIDS events, in conjunction with end stage renal and liver disease, as well as cancers in REPRIEVE (A5332). These data are critical to obtain in order to understand the potential overall impact of statins on **PWH**.

2.1.3.3 Efficacy of Statin Therapy in HIV

In **PWH**, statins safely and effectively lower LDL cholesterol [Calza 2012; Ganesan 2011; Silverberg 2009]. and also exert immunomodulatory effects to 1) decrease monocyte activation - reflected in decreased circulating levels of sCD14 and the macrophage-derived phospholipase, Lp-PLA2 [Eckard 2014; Funderburg 2014, 2015] and 2) decrease T-cell activation in some studies [De Wit 2011; Ganesan 2011]. Statins are generally well tolerated among **PWH** and introduce minimal risks balanced against their significant potential to prevent CVD in this population [Singh 2011]. In a large cohort study, Silverberg et al. demonstrated that statins can be safely administered to **PWH**. Increased relative rates of grade III myositis (1.9% vs. 0.5%) and liver function test (LFT) abnormalities (1.1% vs. 0.3%) were seen in **PWH** versus control participants, but absolute rates were low [Silverberg 2009]. With respect to the potential to induce DM development with statin use in **PWH**, non-randomized cohort studies recently reported contrasting results, with one study showing increased DM risk [Lichtenstein 2015] and two others suggesting no increased risk [Overton 2013; Spagnuolo 2017]. Unlike other agents with immune suppressant effects [Paton 2012], statins have not been shown to have adverse effects on viral replication [Moncunill 2005; Negredo 2006]. Indeed, in vitro studies suggest numerous mechanisms through which statins may actually reduce viral replication although clinical data suggests little impact on measured changes in plasma HIV viral loads while on statin therapy [Amet 2008; del Real 2004; Giguere 2004; Gilbert 2005; Nabatov 2007].

2.1.3.4 Selection of Pitavastatin

Several statins are currently FDA approved and commercially available, with a limited number available in generic form; however**,** the use of select statins in **PWH** is complicated by, or in some cases contraindicated based on, complex interactions with antiretroviral agents [Aberg 2017; Ahmed 2012; Eckard 2014; Funderburg 2014, 2015]. Most statins are primarily metabolized by the CYP3A4 system. HIV protease inhibitors inhibit CYP3A4 and thus markedly increase exposure to these statins, as reflected in the area under the plasma drug concentration-time curve (AUC) [Chauvin 2013]. As such, simvastatin and lovastatin are contraindicated in **PWH** taking protease inhibitors. Atorvastatin is partially metabolized by the CYP3A4 system, and the exposure to this statin is moderately increased by protease inhibitor therapy. Pravastatin exposure may be decreased by co-administration with ritonavir, but increased with co-administration with ritonavir-boosted darunavir. Similarly, rosuvastatin exposure is increased by co-administration with ritonavir-boosted darunavir [Samineni 2012]. In contrast, no clinically significant effects of darunavir or lopinavir/ritonavir on pitavastatin were

seen [Aberg 2017; Eckard 2014; Funderburg 2014, 2015; Malvestutto 2014]. Other effects are also seen, for example between efavirenz, a widely prescribed ART, on the AUC of various common statins, including atorvastatin (-43% AUC), pravastatin (-40% AUC), and simvastatin (-58% AUC) [Gerber 2005], in contrast there was only an 11% change in pitavastatin AUC with efavirenz [Malvestutto 2014].

Pitavastatin is a relatively new statin with comparable efficacy in terms of LDL reduction and anti-inflammatory effects as other potent statins [Eriksson 2011**a**]. It is an excellent choice for **PWH** as it is not metabolized by the CYP3A4 system but is instead metabolized primarily by glucuronidation [Gotto 2010]. Consequently, there are minimal documented interactions between pitavastatin and antiretroviral agents, including minimal effects of pitavastatin on exposure to ritonavir (+8% AUC), darunavir (+3% AUC), atazanavir (+6% AUC), lopinavir (-9% AUC) [FDA package insert]. Similarly, relatively modest effects of individual antiretroviral drugs are seen with respect to pitavastatin AUC [FDA package insert]. Indeed, there is no contraindication or recommendation for dose adjustment when pitavastatin is used in concert with any specific antiretroviral agent. Importantly, pitavastatin 4 mg/day has been shown to effectively decrease LDL cholesterol among ART-treated **PWH**: the INTREPID study randomized **PWH** to pitavastatin 4 mg/day versus pravastatin 40 mg/day [Aberg 2017]. Results demonstrated that pitavastatin was superior to pravastatin with respect to LDL cholesterol lowering (-49.4 mg/dL (-31.1%) vs. -33.6 mg/dl (-20.9%)) over a 12-week period with results sustained over 52 weeks [Aberg 2017]. Moreover, relative to pravastatin, pitavastatin showed significantly greater ability to lower total cholesterol, non-HDL cholesterol, TC:HDL ratio, and Apo B lipoprotein levels. Tolerability and toxicity profiles were similarly benign for both agents: myalgias and LFT abnormalities were rare in both groups; no effects on HIV RNA or CD4 were seen. Of note, pitavastatin, like pravastatin, had a neutral effect on blood glucose and HgbA1c levels in **PWH** [Aberg 2017]. Pitavastatin has compared favorably to other statins in randomized trials among **people without HIV** as well [Eriksson 2011**a**; Eriksson 2011**b**; Gumprecht 2011; Maruyama 2011]. Additionally, pitavastatin has demonstrated little impact on glucose metabolism and may even improve insulin resistance, an additional benefit when compared with other statins [Aberg 2017; Eckard 2014; Funderburg 2014, 2015; Teramoto 2010; Yamakawa 2008; Yokote 2009].

2.1.4 Opportune Timing for Randomized Control Trial of Statin Therapy in HIV

The JUPITER trial demonstrated that among individuals in the general population with LDL cholesterol <130 mg/dL and moderate inflammation (defined as high sensitivity C-reactive protein >2 mg/L), statin therapy resulted in a 44% reduction in CVD events [Ridker 2008]. To date, there are no large scale randomized trials

of statin therapy to prevent CVD among **PWH**. Non-randomized studies in **PWH** demonstrate that use of statins is associated with reduced overall mortality [Moore 2011], especially among those diagnosed with co-morbidities [Rasmussen 2013]. Moore et al. showed that statin use was associated with a with a three-fold reduction in hazard ratio for all-cause mortality among **PWH**, adjusting for age, CD4, HIV-1 RNA, cholesterol levels, and prior ART use [Moore 2011]. Observational studies also show a trend toward reduction in non-AIDS complications among ART-treated **PWH** on high potency statins [Drechsler 2017].

Despite the potential appeal of statins, their use is relatively low in **PWH**—19.6% in a 2013 survey commissioned from the ACTG. Another recent analysis reported significant clinical inertia for the utilization of statin therapy in well controlled **people living with HIV** highlighting that both patient and provider factors weigh into the decision whether to utilize statins [Willig 2008]. The low use of statins in **PWH** reflects in part the relatively low prevalence of increased LDL cholesterol in this group [Freiberg 2013], but more importantly, the uncertain efficacy for CVD prevention and the potential side effects and ART interactions. These factors suggest equipoise and opportune timing for a large randomized trial to assess efficacy and safety of a statin therapy strategy for primary CVD prevention in HIV among those not meeting recommendations for statins under the 2013 American College of Cardiology/American Heart Association (ACC/AHA) guidelines.

In 2013, the ACC/AHA released Blood Cholesterol Guidelines to replace the guidelines from the Third Report of the Expert Panel on Detection, Evaluation, and Treatment of High Blood Cholesterol in Adults (Adult Treatment Panel III). Novel aspects of the guidelines included 1) delineation of four statin benefit groups—patients age ≥21 with clinical ASCVD and/or with LDL cholesterol ≥190 mg/dL and patients age ≥40 and ≤75 with diabetes and/or with 10-year ASCVD risk estimated to be ≥7.5% by the Pooled Cohort Equations and 2) abandonment of LDL cholesterol and non-HDL cholesterol treatment goals. In addition, the new guidelines deemphasized the use of non-statin lipid lowering therapies and focused exclusively on the use of statins for primary CVD prevention [Stone 2014]. In this regard, pitavastatin is included as a recommended therapy in the new guidelines for primary prevention. Although these guidelines are now recommended for general use in clinical practice, they do not incorporate into risk prediction novel HIV-specific factors, including degree of systemic immune activation. REPRIEVE (A5332) provides a critical opportunity, in line with the hypothesis of the grant, to test whether patients without significant traditional risk, eg, those with ASCVD scores <7.5%, are an appropriate group for statin primary prevention. The currently proposed trial achieves equipoise by testing the efficacy and safety of statin therapy for primary CVD prevention in HIV among those not meeting recommendations for statins under the 2013 ACC/AHA guidelines and speaks directly to critical knowledge gaps identified by the recently published ACC/AHA guidelines for cholesterol management and the AHA sponsored State of the Science Conference on CVD in HIV. In alignment

with these guidelines, individuals aged 40-75 will be recruited for REPRIEVE (A5332). In order to be fully aligned with the 2013 ACC/AHA Guidelines, persons with diabetes and LDL <70mg/dL or ASCVD risk score ≥7.5% and LDL <70mg/dL are not recommended to initiate statins, thus these two groups will be included in the study.

Although we do not anticipate a change in the guidelines occurring during the REPRIEVE (A5332) study, we will address any future changes in the guidelines by obtaining Data and Safety Monitoring Board (DSMB) review to determine whether equipoise has been disturbed and whether any changes to the study should be made as a result, updating the informed consent documents to inform participants about the change, and providing participants with a letter describing the new guidelines to take to their personal physicians to determine whether they should continue to participate in the study in light of the change.

2.1.5 Rationale for the Change in 10-Year ASCVD Risk Score

Following the launch of REPRIEVE (A5332), the team found that a number of candidates were being excluded from the trial in the moderate risk category with an ASCVD risk score ≤10%. These individuals were not otherwise being offered statins clinically, and equipoise exists for their entry into this randomized, placebo-controlled trial. Moreover, it was noted by the team that certain demographic and risk factor groups were being excluded from the trial disproportionately based on higher ASCVD risk scores, despite relatively low LDL cholesterol levels. For example, black/African American men and smokers were often excluded at very young ages, despite uncertainty about the efficacy of statins among HIV+ individuals with such risk factors. The exclusion from the study of certain demographic groups overrepresented in the US HIV epidemic reduces the generalizability of the study and precludes from study a large number of individuals with moderate risk for whom the question of statin use for primary CVD prevention was critical.

During a DSMB meeting on June 8, 2015, the REPRIEVE (A5332) team leadership reported these findings and proposed a change in the ASCVD risk score entry criteria in order to improve the generalizability of the study results and the enrollment of participants for whom statins are not generally offered. The DSMB agreed with the study team's assessment and proposal to raise the upper bound for the enrollment ASCVD risk score from <7.5% to ≤10%, if those individuals between 7.5% and 10% had an LDL <160 mg/dL. The rationale for having a lower threshold for the LDL cholesterol for persons with an ASCVD risk score between 7.5% and 10% was to focus study enrollment on those persons with low or moderate risk who were unlikely to be prescribed statin therapy. The comments from the DSMB affirm their agreement with this assessment and proposal and noted: "1) there are no trial data validating the ASCVD risk prediction equations in **PWH**; 2) there are no definitive data for **PWH** with ASCVD risk scores of 7.5%-10% regarding the use of statins; 3) the use of statins in this group is not mandated by clinical experience or guidelines, allowing for the necessary equipoise for inclusion

of this group in REPRIEVE (A5332); 4) the consent should ensure the opportunity for an informed discussion between patients and providers with ASCVD risk scores between 7.5%-10% and patients/providers could elect not to pursue the trial; and 5) raising the ASCVD risk score might permit enrollment of study populations, including smokers and minority patients, that would be more representative of the actual HIV population in the United States." The DSMB further agreed that the 2013 guidelines were designed to encourage a discussion of the relative risk and benefits of statins for persons with an ASCVD risk score between 7.5%-15% (considered the moderate risk category), and that prescription of statins was not mandatory in this group, especially in the case of **PWH** for whom there were no trial data. Furthermore, the requirement to limit trial participants in the range of 7.5%-10% ASCVD risk category to those with an LDL cholesterol <160 mg/dL would help to ensure that those with relatively higher ASCVD risk and elevated LDL levels were not enrolled into the trial. This level of LDL cholesterol was selected based on the recently updated National Lipid Association Recommendations that suggest consideration for initiating statin therapy for moderate risk individuals (ie, risk score <15%) [Jacobson 2014].

During a DSMB meeting on June 24, 2016, the REPRIEVE (A5332) team proposed a further change in the 10-year ASCVD risk score entry criteria after finding that a partial increase in the risk score cutoff from 7.5% to 10% only slightly increased the median risk of the study population. Approximately 6 months after the change, the median risk remained low at 3.6%. Moreover, persons of black race tended to be disproportionately excluded as the scoring is weighted such that, for this racial group, the ASCVD risk score is higher for a given set of parameters. The net result of the use of the score at the current level has been:

- Enrollment of a low-risk population with fewer black participants and smokers than would be representative of the relevant at-risk population.
- Enrollment of a non-representative, low-risk population for whom prevention is less relevant.
- An adverse impact on enrollment and perhaps a reduction in event rates to a level that was not envisioned.

REPRIEVE (A5332) is the first prospective study to assess the accuracy of the ASCVD scoring system in HIV+ individuals, but will be limited in its utility if participants are clustered at the very low end of this risk scale. Moreover, the 2013 ACC/AHA Prevention Guidelines are designed to assess 10-year and lifetime risk, and it is not yet known how well it will predict rates over 3 to 5 years in a trial. To rectify this situation, the team proposed, and the DSMB approved, an increase in the threshold for exclusion from ≤10% to ≤15%, requiring an LDL <130 mg/dL for those with a risk score >10% and ≤15%. Recent data [Nayor 2016] highlight the uncertainty of the correct threshold for statin initiation to be used with the ASCVD criteria, and this change is in line with the recommendation of at least one major guideline, from Canada.

In alignment with the guidelines, this change in the risk score threshold will facilitate enrollment of the intended population of intermediate risk participants for whom uncertainty and equipoise exist, and will permit a more generalizable and

relevant study population, representative of the at-risk HIV population. Enrollment of participants with a broader range of scores will also better permit REPRIEVE (A5332) to assess the relative utility of the ASCVD score in the HIV population, leveraging the study for a critical purpose. The graded LDL requirement will further ensure equipoise, and will help to prevent any significant crossover rate.

2.1.6 Rationale for the Enrollment Cap for 10-Year ASCVD Risk Score <2.5%

Based on recommendations of the REPRIEVE (A5332) DSMB following their December 2017 review, the REPRIEVE team has capped enrollment of persons with a very low cardiovascular risk (10-year ASCVD risk score <2.5%) at approximately 2000 participants to ensure that final study population reflects the targeted low-to-moderate-risk population that the study is intending to enroll. Despite the previous expansion of the upper bound of the allowable entry criteria to allow enrollment of persons with 10-year ASCVD risk score ≤15%, the DSMB noted that median ASCVD risk score for the entire cohort remained low at 3.4% with approximately 2000 participants with a 10-year ASCVD risk score <2.5%.

The cessation of enrollment of persons with very low 10-year ASCVD score will ensure that the study enrolls a study cohort that is more reflective of the entire HIV population at risk for ASCVD and ensures that the study maintains adequate power to determine the efficacy of pitavastatin for primary ASCVD prevention in the setting of HIV infection over a broad range of scenarios for the rate of events of interest.

2.1.7 Rationale for the Enrollment Cap for 10-Year ASCVD Risk Score <5%

Based on a recommendation from the National Institutes of Health (NIH) (the National Heart, Lung, and Blood Institute [NHLBI] and the Division of AIDS [DAIDS] at the National Institute of Allergy and Infectious Diseases [NIAID]) in April 2018, the REPRIEVE team is capping enrollment of persons with low cardiovascular risk (10-year ASCVD risk score <5%) at approximately 4200 participants to ensure that the final study population reflects the targeted low-to-moderate-risk population.

2.1.8 **Rationale for the Study of SARS-CoV-2**

The COVID-19 pandemic gripped the globe in 2020 and continues to have significant impact, with SARS-CoV-2 predicted to infect a large percentage of the global population and cause significant morbidity and mortality [Walker 2020]. Cardiovascular (CV) involvement is common, including myocarditis, heart failure, ischemic events due to thromboembolism and supply/demand mismatch, and arrhythmias [Madjid 2020; Zheng 2020]. The impact of COVID-19 on the large global population of persons with HIV who have excess risk for CV disease is unknown. REPRIEVE is uniquely positioned to serve as a platform to understand the epidemiology, genetics, and pathobiology of

COVID-19-related CVD in HIV, as well as the effects of statins on COVID-19 in a large, global RCT.

COVID-19 may increase CVD through immunological pathways that have been characterized as part of the cytokine release syndrome [Saghazadeh 2020]. Even without such advanced disease presentations, the HIV population is particularly important to study, given that PWH concomitantly experience reduced immune function and increased innate immune activation [Lederman 2013]. Investigation in this population may provide insights into COVID-19 of relevance to the general population.

REPRIEVE will be used to identify host factors influencing risk for SARS-CoV-2 infection, COVID-19 disease, and CV complications of COVID-19.

The randomized design of REPRIEVE is ideal to study whether statins' pleotropic immune-modulatory actions and known protection against virally-mediated myocardial injury [Guan 2010] and acute lung injury [Brett 2011] impact COVID-19 infection. Statins have been shown to reduce hyperinflammatory subphenotype of acute respiratory distress syndrome, increasing 28-day survival [Calfee 2018], and thus may be particularly useful to reduce inflammatory conditions in other systems, including CVD. Moreover, ongoing use of statins, rather than as acute therapy, may be protective and help to prevent such disease before it becomes advanced and difficult to treat. Pitavastatin therapy—the statin used in REPRIEVE—has the most potent immune modulatory effects in PWH [Toribio 2017], affects multiple potentially relevant immune pathways in this population [deFilippi 2020], and has inhibitory activity against SARS-CoV-2 proteins [Xu 2020]. In this regard, we will test whether the therapy in REPRIEVE will ameliorate COVID-19-related hospitalization and ensuing COVID-19-related cardiovascular events including myocardial infarction and/or heart failure.

2.2 Rationale

PWH face an increased risk of CVD morbidity and mortality, yet no preventive strategies for CVD risk reduction have been proven for this population. Existing primary CVD preventive strategies for the general population cannot simply be extrapolated to **PWH**, in whom immune dysfunction/activation contributes in unique ways to atherosclerosis and ensuing cardiovascular events. The rationale of testing statins for primary CVD prevention in HIV is multifold: First, statins affect both traditional CVD risk factors (LDL cholesterol) and have beneficial pleiotropic "off-target" effects, i.e., reduction in immune activation. The latter effect is critical, as immune activation and persistent inflammation in HIV are thought to contribute importantly to the development of non-calcified, vulnerable, and inflamed coronary atherosclerotic plaque even in relatively young **PWH** on ART with low traditional CVD risk indices [Subramanian 2012; Yarasheski 2012; Zanni 2013]. In such patients, statin therapy may stabilize high-risk coronary atherosclerotic plaque, precluding rupture and AMI. Moreover, statins are generally safe in the HIV population, exerting anti-inflammatory properties without enhancing viremia

[Aberg 2017; Eckard 2014; Funderburg 2014, 2015; Ganesan 2011; Nabatov 2007; Negredo 2006; Silverberg 2009]. Based on this rationale, the REPRIEVE (A5332) study was designed to assess the efficacy of statins as a primary prevention strategy for CVD events in **PWH** on ART not meeting 2013 ACC/AHA guideline thresholds for recommended statin initiation. The study will also definitively determine the safety of statins in **PWH**, including statin effects on non-CVD events such as incident diabetes, malignancies, kidney or liver failure, and AIDS-defining events in **PWH**. In addition to enrolling **participants** in the main study, select sites will have the option of coenrolling participants in a Mechanistic Substudy of REPRIEVE (A5333s). As part of this substudy, described in detail in Appendix II, coenrolled patients will undergo detailed coronary CT angiography and biochemical immunophenotyping. The Mechanistic Substudy of REPRIEVE (A5333s) will allow for determination of statin effect on non-calcified coronary atherosclerotic plaque burden and morphology, as well as lipid and immune parameters predictive of this effect. REPRIEVE (A5332) addresses an urgent national healthcare priority to prevent CVD among persons living with HIV.

REPRIEVE will address critical knowledge gaps regarding SARS-CoV-2 infection and COVID-related CV complications among PWH. We will focus on three interrelated but independent key topics: epidemiology and scope of COVID-19-related CVD in HIV, host factors, and protective strategies. Uniform study-wide eCRF assessment tools to ascertain symptoms (including long-term COVID-19 effects) and antibody testing will allow for an objective and unbiased determination of epidemiology of COVID-19, its effects on CVD in HIV, and statin effects across the globe for the first time in HIV. The results of this work will provide critical information on COVID-19-related CVD in HIV, and critical effects of statins to mitigate effects of the SARS-CoV-2 virus-related infection, disease severity, and MACE. These results will be broadly generalizable to the large population of PWH simultaneously at risk for COVID-19 and CVD and also to other populations at risk for CVD experiencing COVID-19 infection.

3.0 STUDY DESIGN

REPRIEVE (A5332) is a prospective, double-blind, randomized, placebo-controlled, multicenter phase III efficacy study that will examine the effects of 4 mg daily pitavastatin on cardiovascular-related events among **adults with HIV** who are currently on ART. The randomization in the study will be stratified by sex at birth, CD4+ T-cell counts (≤500 vs. >500 cells/mm^3), and by whether or not a participant has elected to participate in the Mechanistic Substudy of REPRIEVE (A5333s) (yes/no).

Men and women ≥40 and ≤75 years of age, on any ART regimen (ART not provided by the study) for at least 6 months prior to study entry, with any plasma HIV-1 RNA level, with CD4+ T-cell count >100 cells/mm^3 considered low-to-moderate risk using the 2013 ACC/AHA guideline thresholds for recommended statin initiation will be enrolled into this study. Per the December 2017 DSMB recommendations, the study sample size was increased to approximately 7500 participants, follow-up was extended to approximately 84 months from the time the first participant enrolled, and on 01/19/18, total enrollment of individuals with very low cardiovascular risk (10-year ASCVD risk score <2.5%) was capped at approximately 2000 participants to ensure that final study population reflects

the targeted low-to-moderate-risk population. Subsequent to a new recommendation from the NIH, the total enrollment of individuals with low cardiovascular risk (10-year ASCVD risk score <5.0%) was capped at approximately 4200 participants to ensure that the final study population reflects the targeted low-to-moderate-risk population. At this same time the NIH recommended to increase follow-up to 96 months. This increase was endorsed by the DSMB at the December 2018 meeting. **Follow-up is now up to 120 months in order to continue until the study reaches its target of 288 primary MACE endpoints or is otherwise recommended for closure by the DSMB (see section 9.1 for more details).**

At study entry, participants will be randomized to one of the following arms:
ARM A: At Day 0, initiate pitavastatin at a daily dose of 4 mg.
ARM B: At Day 0, initiate placebo for pitavastatin daily.

Clinical assessments will be performed at month 1, month 4, and then every 4 months for the duration of the study.

4.0 SELECTION AND ENROLLMENT OF PARTICIPANTS

4.1 Inclusion Criteria

4.1.1 Documentation of HIV-1 infection by means of any one of the following:

- Documentation of HIV diagnosis in the medical record by a licensed health care provider;
- OR HIV-1 RNA detection by a licensed HIV-1 RNA assay demonstrating >1000 RNA copies/mL;
- OR any licensed HIV screening antibody and/or HIV antibody/antigen combination assay confirmed by a second licensed HIV assay such as a HIV-1 Western blot confirmation or HIV rapid Multispot antibody differentiation assay.

NOTE: A “licensed” assay refers to a US FDA-approved assay, which is required for all IND studies. Non-US sites are encouraged to use FDA-approved methods; if not available, then each non-US site must use an assay that has been certified or licensed by an oversight body within that country and validated internally.

WHO (World Health Organization) and CDC (Centers for Disease Control and Prevention) guidelines mandate that confirmation of the initial test result must use a test that is different from the one used for the initial assessment.

4.1.2 Combination antiretroviral therapy (ART) for at least 180 days prior to study entry.

NOTE: Treatment interruptions for up to 30 days total in the last 180 days are permitted as long as the participant has been continuously on therapy for the 30 days prior to study entry.

4.1.3 CD4+ cell count >100 cells/mm^3 obtained within 180 days prior to study entry at any US laboratory that has a Clinical Laboratory Improvement Amendments (CLIA) certification or its equivalent, or at any network-approved non-US laboratory that operates in accordance with Good Clinical Laboratory Practices and participates in appropriate external quality assurance programs.

4.1.4 Laboratory values drawn at screen and/or obtained from clinical care (as indicated in section 6.1 Schedule of Evaluations) within 90 days prior to study entry at any US laboratory that has a Clinical Laboratory Improvement Amendments (CLIA) certification or its equivalent, or at any network-approved non-US laboratory that operates in accordance with Good Clinical Laboratory Practices and participates in appropriate external quality assurance programs.

- Fasting LDL cholesterol as follows:
    - If ASCVD risk score <7.5%, LDL cholesterol must be <190 mg/dL
    - If ASCVD risk score ≥7.5% and ≤10%, LDL must be <160 mg/dL
    - If ASCVD risk score >10% and ≤15%, LDL must be <130 mg/dL

  NOTE: If LDL <70 mg/dL, participant is eligible regardless of 10-year ASCVD risk score in line with the ACC/AHA 2013 Prevention Guidelines.

- Fasting triglycerides <500 mg/dL
- Hemoglobin ≥8 g/dL for female participants and ≥9 g/dL for male participants
- Glomerular filtration rate (GFR) ≥60 mL/min/1.73m^2 or creatinine clearance (CrCl) ≥60 mL/min
  NOTE: See the A5332 Manual of Procedures (MOPS) for links to GFR and CrCl calculators.
- ALT ≤2.5 x ULN
  NOTE: Participants co-infected with chronic active hepatitis B or C must have ALT ≤2 x ULN.

4.1.5 For persons with known chronic active hepatitis B or C, calculated FIB-4 score must be ≤3.25.

NOTE: Active is defined as hepatitis B surface antigen positive, hepatitis B DNA positive, or hepatitis C RNA positive.

NOTE: Refer to the calculator for the FIB-4 equation in the MOPS.

4.1.6 Female participants of reproductive potential (defined as women who have not been post-menopausal for at least 24 consecutive months, ie, who have had menses within 24 months prior to study entry, and women who have not undergone surgical sterilization, specifically hysterectomy or bilateral oophorectomy) must have a negative serum or urine pregnancy test within 48 hours prior to entry by any US laboratory or clinic that has a CLIA certification or its equivalent, or is using a point-of-care (POC)/CLIA-waived test, or at any network-approved non-US laboratory or clinic that operates in accordance with

Good Clinical Laboratory Practices and participates in appropriate external quality assurance programs.

NOTE: Participant-reported history is considered acceptable documentation of hysterectomy, bilateral oophorectomy, and menopause. Women are considered menopausal if they have not had a menses for at least 12 months and have a FSH (follicle stimulating hormone) of greater than 40 IU/L or, if FSH testing is not available, they have had amenorrhea for 24 consecutive months.

4.1.7 For women of reproductive potential, willingness to use contraceptives as described in the product information for pitavastatin. Contraceptives must be used at least two weeks before initiation of study drug and must be continued 6 weeks after cessation of study drug.
If participating in sexual activity that could lead to pregnancy, women must use a form of contraceptive. At least one of the following methods must be used appropriately:

- Condoms (male or female) with or without spermicidal agent
- Diaphragm or cervical cap with spermicidal agent
- Intrauterine device (IUD)
- Hormone-based contraceptive
- Tubal ligation
- Tubal micro-inserts

Women who are not of reproductive potential as defined above are eligible without the use of contraception.

4.1.8 Men and women age ≥40 and ≤75 years of age.

4.1.9 Ability and willingness of participant or legal representative to provide written informed consent.

4.2 Exclusion Criteria

4.2.1 Clinical ASCVD, as defined by 2013 ACC/AHA guidelines, including a previous diagnosis of any of the following:

- AMI
- Acute coronary syndromes
- Stable or unstable angina
- Coronary or other arterial revascularization
- Stroke
- TIA
- Peripheral arterial disease presumed to be of atherosclerotic origin

4.2.2 Current diabetes mellitus if LDL ≥70 mg/dL

NOTE: Current diabetes is defined by patient report of physician diagnosis. Participants with a history of diabetes that has resolved and no longer requires

therapy are not considered to have current diabetes, eg, women with a history of gestational diabetes, steroid-induced or medication-induced.

4.2.3 10-year ASCVD risk score estimated by Pooled Cohort Equations >15%

NOTES:

- If LDL <70 mg/dL, participant is eligible regardless of risk score in line with the ACC/AHA 2013 Prevention Guidelines.
- See inclusion criterion 4.1.4 for LDL requirements by risk score.
- See section 6.3.4, Cardiovascular Risk Assessment Tool, for detailed instructions concerning access and use of the 10-year ASCVD risk score calculator.

4.2.4 Active cancer within 12 months prior to study entry.

NOTE: Exceptions:

- Successfully treated non-melanomatous skin cancer
- Kaposi sarcoma without visceral organ involvement

4.2.5 Known decompensated cirrhosis.

4.2.6 History of myositis or myopathy with active disease in the 180 days prior to study entry.

4.2.7 Known untreated symptomatic thyroid disease.

4.2.8 History of allergy or severe adverse reaction to statins.

4.2.9 Use of specific immunosuppressants or immunomodulatory agents, including but not limited to tacrolimus, sirolimus, rapamycin, mycophenolate, cyclosporine, TNF-alpha blockers or antagonists, azathioprine, interferon, growth factors, or intravenous immunoglobulin (IVIG), in the 30 days prior to study entry.

NOTE: Use of oral prednisone ≤10 mg/day or equivalent dosage is allowed.

NOTE: Refer to the MOPS for clarification regarding medications in these categories.

4.2.10 Current use of erythromycin, colchicine, or rifampin.

4.2.11 Use of any statin drugs, gemfibrozil, or PCSK9 inhibitors in the 90 days prior to study entry.

4.2.12 Current use of an investigational new drug that would be contraindicated.

NOTE: Please contact the protocol core team via e-mail as described in the Study Management section for guidance on coenrollment of participants on investigational new drugs.

4.2.13 Serious illness or trauma requiring systemic treatment or hospitalization in the 30 days prior to study entry.

4.2.14 Known active or recent (not fully resolved within 30 days prior to study entry) systemic bacterial, fungal, parasitic, or viral infections (except HIV, HBV, human papillomavirus [HPV], or HCV).

4.2.15 Current breastfeeding.

4.2.16 Alcohol or drug use that, in the opinion of the site investigator, would interfere with completion of study procedures.

4.2.17 Other medical, psychiatric, or psychological condition that, in the opinion of the site investigator, would interfere with completion of study procedures and or adherence to study drug.

4.3 Study Enrollment Procedures

4.3.1 Prior to implementation of this protocol, and any subsequent full version amendments, each site must have the protocol and the protocol consent form(s) approved, as appropriate, by their local institutional review board (IRB)/ethics committee (EC) and any other applicable regulatory entity (RE). Upon receiving final approval, sites will submit all required protocol registration documents to the DAIDS Protocol Registration Office (DAIDS PRO) at the Regulatory Support Center (RSC). The DAIDS PRO will review the submitted protocol registration packet to ensure that all of the required documents have been received.

Initial site-specific informed consent forms (ICFs) will be reviewed and approved by the DAIDS PRO, and sites will receive an Initial Registration Notification from the DAIDS PRO that indicates successful completion of the protocol registration process. A copy of the Initial Registration Notification should be retained in the site's regulatory files.

Upon receiving final IRB/EC and any other applicable RE approvals for an amendment, sites should implement the amendment immediately. Sites are required to submit an amendment registration packet to the DAIDS PRO at the RSC. The DAIDS PRO will review the submitted protocol registration packet to ensure that all the required documents have been received. Site-specific ICF(s) WILL NOT be reviewed and approved by the DAIDS PRO, and sites will receive an Amendment Registration Notification when the DAIDS PRO receives a complete registration packet. A copy of the Amendment Registration Notification should be retained in the site's regulatory files.

For additional information on the protocol registration process and specific documents required for initial and amendment registrations, refer to the current version of the DAIDS Protocol Registration Manual https://rsc.niaid.nih.gov/clinical-research-sites/daids-protocol-registration-policy-and-procedures-manual.

Once a candidate for study entry has been identified, details including risks and benefits will be carefully discussed with the participant. The participant (or, when necessary, the legal representative if the participant is under guardianship) will be asked to read and sign the approved protocol consent form.

For participants from whom a signed informed consent has been obtained, a REPRIEVE Screening Checklist must be entered through the Data Management Center (DMC) Participant Enrollment System.

4.3.2 Participant Randomization

Participants will be randomized according to standard data management procedures. For participants from whom informed consent has been obtained, but who are deemed ineligible or who are not randomized into REPRIEVE (A5332), a Screening Outcome form must be recorded in the Electronic Data Capture system (EDC).

4.4 Mechanistic Substudy of REPRIEVE (A5333s) Enrollment Procedures

If applicable, the Mechanistic Substudy of REPRIEVE (A5333s) enrollment will occur at the same time as enrollment into REPRIEVE (A5332), ie, completion of enrollment into A5333s should occur no later than one business day after enrollment into REPRIEVE (A5332). Please see Appendix II for additional information regarding substudy enrollment procedures.
NOTE: The Mechanistic Substudy of REPRIEVE (A5333s) closed to accrual on 02/06/18.

4.5 Coenrollment Guidelines

US ACTG sites are encouraged to coenroll participants in A5128, “Plan for Obtaining Informed Consent to Use Stored Human Biological Materials (HBM) for Currently Unspecified Analyses.” Non-US ACTG sites are encouraged to coenroll participants in A5243, “Plan for Obtaining Human Biological Samples at Non-US Clinical Research Sites for Currently Unspecified Genetic Analyses.” Coenrollment in A5128, observational studies, or studies that do not involve a random treatment assignment does not require permission from the REPRIEVE (A5332) protocol team.

For specific questions and approval for coenrollment in other studies (including HCV or HIV treatment trials and adjunctive treatment trials), sites should first check the PSWP or contact the protocol core team via e-mail as described in the Study Management section.

4.6 Retention Procedures

Participants who miss visits should be contacted to make up the visit and to permit determination of whether a major event occurred. If a participant refuses to communicate with study staff in any context and avoids further participation in the study after repeated attempts or the site is unable to contact the participant by phone, mail, primary care physician, next of kin, home visits, or emergency contact numbers after repeated attempts, then the participant should be taken off study.

Participants who self-withdraw from study medications should be followed (if they agree) and evaluated.

See MOPS for recruitment and retention procedures, including contact and data collection for participants in the event of missed visits.

5.0 STUDY TREATMENT

Study treatment is defined as pitavastatin and placebo for pitavastatin, both of which will be provided by the study.

5.1 Regimens, Administration, and Duration

5.1.1 Regimens

At study entry (day 0), participants will be randomized to one of the following arms:

ARM A: Pitavastatin 4 mg one tablet once daily taken orally with or without food.
ARM B: Placebo for pitavastatin one tablet once daily taken orally with or without food.

5.1.2 Administration

The appropriate dose of pitavastatin or placebo for pitavastatin can be administered at any time of the day, with or without food. The drug should be taken as close to the same time of day as possible. With the exception of participants enrolled in the Mechanistic Substudy (A5333s) or select other approved REPRIEVE (A5332) ancillary studies, participants must begin treatment within 72 hours after randomization.

NOTE: For participants coenrolled in the Mechanistic Substudy (A5333s) or other specified approved REPRIEVE (A5332) ancillary studies with ancillary study procedures, the initiation of treatment must be held until after the entry CCTA or relevant ancillary study procedure (see the A5332 MOPS for more details), even if the CCTA or relevant ancillary study procedure occurs more than 72 hours

after randomization but not more than 14 days after randomization. See the A5332 MOPS for more details regarding the specified ancillary studies.

Study product will be dispensed in accordance with the Drug Dispensation schedule as described in the MOPS.

NOTE: The drug dispensation schedule described in the MOPS is a preferred guidance schema. Sites should notify the DAIDS Pharmaceutical Affairs Branch (PAB) at PABREPRIEVEPEP@mail.nih.gov of any deviation from this schema and document such occurrences in dispensation/accountability logs.

5.1.3 Treatment Duration

Participants will remain on study treatment for approximately **120** months depending on the time they enrolled in the study.

5.2 Study Product Formulation and Preparation

Pitavastatin and placebo for pitavastatin should be stored at room temperature between 15–30°C (59–86°F). The product should be stored out of direct sunlight. The container should be kept tightly closed retaining the silica gel desiccant in the bottle. Dispense only in the original container.

5.3 Pharmacy: Product Supply, Distribution, and Accountability

5.3.1 Study Product Acquisition/Distribution

Pitavastatin and placebo for pitavastatin will be provided by Kowa Pharmaceuticals America, Inc. These study products will be available through the NIAID Clinical Research Products Management Center (CRPMC). The clinical research site (CRS) pharmacist can obtain the study products for this protocol by following the instructions in the manual *Pharmacy Guidelines and Instructions for Division of AIDS (DAIDS) Clinical Trials Networks*.

5.3.2 Study Product Accountability

The site pharmacist is required to maintain complete records of all study products received from the NIAID CRPMC and subsequently dispensed. All unused study products must be returned to the NIAID CRPMC (or as otherwise directed by the sponsor) after the study is completed or terminated. The procedures to be followed are provided in the manual *Pharmacy Guidelines and Instructions for DAIDS Clinical Trials Networks* in the section Study Product Management Responsibilities. At non-US CRSs, the site pharmacist must follow the instructions in the *Pharmacy Guidelines and Instructions for DAIDS Clinical Trials Networks* for the destruction of unused study products.

5.4 Concomitant Medications

Whenever a concomitant medication or study agent is initiated or a dose changed, investigators must review the concomitant medication's and study agent's most recent package insert, Investigator's Brochure, or updated information from DAIDS to obtain the most current information on drug interactions, contraindications, and precautions. Site staff are encouraged to contact the core protocol team via e-mail with any questions about required, prohibited, or precautionary medications. Refer to MOPS section 5.0 for additional information.

Additional drug information may be found on the updated ACTG Precautionary and Prohibited Medications Database located at http://tprc.pharm.buffalo.edu/home/di_search. This database was created to assist investigators and research staff to access the most current pharmacokinetic data on drug interactions with antiretrovirals. You do not need to register or login to use this resource.

5.4.1 Required Medications

Combination ART for at least 180 days prior to study entry.

NOTE: Treatment interruptions for up to 30 days total in the last 180 days are permitted as long as participant has been continuously on therapy for the 30 days prior to study entry.

5.4.2 Recommended Medications

Prophylaxis for HIV-related opportunistic infections should be administered as per the "Guidelines for the Prevention and Treatment of Opportunistic Infections in **Adults** and Adolescents **with HIV**: Recommendations from the Centers for Disease Control and Prevention, the National Institutes of Health, and the HIV Medicine Association of the Infectious Diseases Society of America."

5.4.3 Prohibited Medications

Refer to the PSWP for a list of prohibited medications.

5.4.4 Precautionary Medications

Refer to the PSWP for a list of precautionary medications.

6.0 CLINICAL AND LABORATORY EVALUATIONS

6.1 Schedule of Evaluations

Table 6.1a: Screening – Months 96

Evaluation	Screening[2]	Entry Day 0	Post-Entry Evaluations (*months*)[1] Visit Window ±7 days for month 1; ±30 days for all other visits																									Study Termination Visit[2]	Discontinuation Visits[2]	
			1	4	8	12[2]	16	20	24[2]	28	32	36[2]	40	44	48[2]	52	56	60[2]	64	68	72[2]	76	80	84[2]	88	92	96[2]		Prem. Treatment D/C Evals ±30 days[2]	Prem. Study D/C Evals[2]
Documentation of HIV-1	x																													
Medical history	x	x[3]	x	x	x	x	x	x	x	x	x	x	x	x	x	x	x	x	x	x	x	x	x	x	x	x	x	x	x	x
Medication history	x	x[3]	x	x	x	x	x	x	x	x	x	x	x	x	x	x	x	x	x	x	x	x	x	x	x	x	x	x	x	x
Myalgia symptom assessment		x	x	x	x	x	x	x	x	x	x	x	x	x	x	x	x	x	x	x	x	x	x	x	x	x	x	x	x	x
**COVID-19 assessment**									**x**	**x**	**x**	**x**	**x**	**x**	**x**	**x**	**x**	**x**	**x**	**x**	**x**	**x**	**x**	**x**	**x**	**x**	**x**	**x**	**x**	**x**
Calculate 10-year ASCVD risk score	x																													
CV**D** risk factor assessment	x																											**x[16]**	**x[16]**	**x[16]**
REAP & DASI questionnaires		x																										x	x	x
Screening physical exam	x																													

Evaluation	Screening[2]	Entry Day 0	Post-Entry Evaluations (*months*)[1]																								Study Termination Visit[2]	Discontinuation Visits[2]	
			Visit Window ±7 days for month 1; ±30 days for all other visits																									Prem. Treatment D/C Evals ±30 days[2]	Prem. Study D/C Evals[2]
			1	4	8	12[2]	16	20	24[2]	28	32	36[2]	40	44	48[2]	52	56	60[2]	64	68	72[2]	76	80	84[2]	88	92	96[2]			
Targeted physical exam			x			x			x			x			x			x			x			x			x	x	x	x
Height	x																													
Weight	x					x			x			x			x			x			x			x			x	x	x	x
Waist circumference	x																											x	x	x
ECG[4]		x																												
Dispense lifestyle information		x				x			x			x			x			x			x			x			x	x	x	x
Labs captured from clinical care																														
CD4+/CD8+[5]	x					x			x			x			x			x			x			x			x	x	x	x
Plasma HIV-1 RNA[6]		x				x			x			x			x			x			x			x			x	x	x	x
CBC[7]	x																											x	x	x
Serum creatinine[7]	x																											x	x	x
Calculated creatinine clearance	x																													
Fasting lipid panel[7,8]	x																													
ALT[7]	x																													
AST[10]	x																													
FIB-4[10]	x																													
Labs to be collected (drawn)																														
ALT (local lab)			x			x																								
Pregnancy testing	x	x	x	x	x	x	x	x	x	x	x	x	x	x	x	x	x	x	x	x	x	x	x	x	x	x	x	x	x	x

Evaluation	Screening[2]	Entry Day 0	Post-Entry Evaluations (*months*)[1]																									Study Termination Visit[2]	Discontinuation Visits[2]	
			Visit Window ±7 days for month 1; ±30 days for all other visits																										Prem. Treatment D/C Evals ±30 days[2]	Prem. Study D/C Evals[2]
			1	4	8	12[2]	16	20	24[2]	28	32	36[2]	40	44	48[2]	52	56	60[2]	64	68	72[2]	76	80	84[2]	88	92	96[2]			
Fasting lipid panel (stored)[9]		x				x			x			x			x			x			x			x			x	x	x	x
Fasting glucose[9]		x				x			x			x			x			x			x			x			x	x	x	x
Fasting plasma/serum for biomarkers – stored[9]		x				x			x			**x**			x			**x**			**x**			**x**			**x**	x	x	x
Urine albumin/creatinine[11]		x				x			x						x															
Whole blood for genetic testing[12]		x																												
Safety labs if indicated[13]			x	x	x	x	x	x	x	x	x	x	x	x	x	x	x	x	x	x	x	x	x	x	x	x	x	x	x	x
Dispense study drug		x		x	x	x	x	x	x	x	x	x	x	x	x	x	x	x	x	x	x	x	x	x	x	x	x			
Medication adherence			x	x	x	x	x	x	x	x	x	x	x	x	x	x	x	x	x	x	x	x	x	x	x	x	x		x	x
Vital status and endpoint follow-up[14]																												x		x
Endpoint assessments[15]			x	x	x	x	x	x	x	x	x	x	x	x	x	x	x	x	x	x	x	x	x	x	x	x	x	x	x	x

1 For the purposes of the study, 1 month equals 30 days.

[2] If a participant is not fasting for a visit for which fasting blood is obtained, reschedule the appointment within 30 days of the original appointment date. See section 6.2.3.

[3] Update any changes from the screening assessment (medical and medication history).

[4] ECG available to site PI, all ECGs will be sent for central read and storage until end of study, no specific ECG findings are exclusionary, except findings which in the judgment of PI render the participant clinically unstable as per clinical read, central readings will not be available to sites, see MOPS.

[5] At the screening visit, CD4+/CD8+ from clinical care within 180 days prior to entry will be used; if not available, this will be drawn as part of the study. For post-entry CD4+/CD8+, the most recent values obtained since the last annual visit will be used. At discontinuation visits, CD4+/CD8+ from clinical care within 180 days of that visit will be used.

[6] For the entry visit, an HIV-1 RNA from clinical care from the prior 180 days will be used; if not available, do not collect. For post-entry evaluations, the most recent viral load obtained from clinical care since the last annual visit will be used. At discontinuation visits, plasma HIV-1 RNA from clinical care within 180 days of that visit will be used.

[7] At the screening visit, results of CBC, creatinine, lipid panel, and ALT (and when needed per footnote #10, AST) will be obtained from clinical care if available within 90 days prior to entry; if not available, draw as part of the study. At discontinuation visits, CBC and creatinine from clinical care within 180 days of that visit will be used.

[8] At the screening visit, direct LDL must be determined if triglyceride 400-500 mg/dL.

[9] Fasting lipid panels, glucose, and plasma/serum for biomarkers from entry visit forward will be performed centrally. Participants may choose to opt out of these entry and post-entry labs.

[10] AST is only required for persons with known chronic active HCV and/or HBV in order to calculate FIB-4 at screening. Use results from clinical care if available within 90 days prior to entry; if not available, draw as part of the study.

[11] The urine for creatinine/albumin is required of all participants at entry. At months 12, 24, and 48, collect only for participants at ACTG sites who enrolled in REPRIEVE (A5332) prior to 01/01/2018.

[12] Whole blood for genetic testing will be drawn at ACTG sites only.

[13] Additional safety labs will be checked at the discretion of site investigator based on participant symptoms (see section 7.1).

[14] Please refer to section 6.3.10 for details.

[15] Please refer to section 6.4 for details.

[16] Hormonal/reproductive health assessments relevant to CVD risk will be determined at study termination/discontinuation visits.

Table 6.1b: Months 100–120

Evaluation	Post-Entry Evaluations (*months*)[1]						Study Termination Visit[2]	Discontinuation Visits[2]	
	Visit Window ±7 days for month 1; ±30 days for all other visits							Prem. Treatment D/C Evals ±30 days[2]	Prem. Study D/C Evals[2]
	100	104	108[2]	112	116	120[2]			
Medical history	x	x	x	x	x	x	x	x	x
Medication history	x	x	x	x	x	x	x	x	x
Myalgia symptom assessment	x	x	x	x	x	x	x	x	x
COVID-19 assessment	x	x	x	x	x	x	x	x	x
CV risk factor assessment							x[10]	x[10]	x[10]
REAP & DASI questionnaires							x	x	x
Targeted physical exam			x			x	x	x	x
Weight			x			x	x	x	x
Waist circumference							x	x	x
Dispense lifestyle information			x			x	x	x	x
Labs captured from clinical care									
CD4+/CD8+[3]			x			x	x	x	x
Plasma HIV-1 RNA[4]			x			x	x	x	x
CBC[5]							x	x	x
Serum creatinine[5]							x	x	x
Labs to be collected (drawn)									
Pregnancy testing	x	x	x	x	x	x	x	x	x
Fasting lipid panel (stored)[6]			x			x	x	x	x
Fasting glucose[6]			x			x	x	x	x
Fasting plasma/serum for biomarkers – stored[6]			x			x	x	x	x
Safety labs if indicated[7]	x	x	x	x	x	x	x	x	x

Evaluation	Post-Entry Evaluations (*months*)[1]						Study Termination Visit[2]	Discontinuation Visits[2]	
	Visit Window ±7 days for month 1; ±30 days for all other visits							Prem. Treatment D/C Evals ±30 days[2]	Prem. Study D/C Evals[2]
	100	104	108	112	116	120			
Dispense study drug	x	x	x	x	x	x			
Medication adherence	x	x	x	x	x				
Vital status and endpoint follow-up[8]							x		x
Endpoint assessments[9]	x	x	x	x	x	x	x	x	x

1 For the purposes of the study, 1 month equals 30 days.

2 If a participant is not fasting for a visit for which fasting blood is obtained, reschedule the appointment within 30 days of the original appointment date. See section 6.2.3.

3 For post-entry CD4+/CD8+, the most recent values obtained since the last annual visit will be used. At discontinuation visits, CD4+/CD8+ from clinical care within 180 days of that visit will be used.

4 For post-entry evaluations, the most recent viral load obtained from clinical care since the last annual visit will be used. At discontinuation visits, plasma HIV-1 RNA from clinical care within 180 days of that visit will be used.

5 At discontinuation visits, CBC and creatinine from clinical care within 180 days of that visit will be used.

6 Fasting lipid panels, glucose, and plasma/serum for biomarkers will be performed centrally. Participants may choose to opt out of these post-entry labs.

7 Additional safety labs will be checked at the discretion of site investigator based on participant symptoms (see section 7.1).

8 Please refer to section 6.3.10 for details.

9 Please refer to section 6.4 for details.

10 Hormonal/reproductive health assessments relevant to CVD risk will be determined at study termination/discontinuation visits.

6.2 Timing of Evaluations

NOTE: For the purposes of the study, 1 month = 30 days.

6.2.1 Screening Evaluations

- Screening evaluations must occur prior to randomization and any study treatment or intervention.
- Screening evaluations to determine eligibility must be completed within 90 days prior to entry unless otherwise specified.
- In addition to data being collected on participants who enroll into the study, demographic, clinical, and laboratory data on screening failures will be captured in a Screening Outcome form in the EDC.

6.2.2 Entry Evaluations

- Entry evaluations will normally occur at least 24 hours after screening evaluations unless otherwise specified. In certain circumstances, screening and entry visits may occur sequentially in real time as long as all screening criteria are confirmed prior to entry.
- Entry evaluations must occur after randomization and be completed before initiating study treatment.
- Participants must begin treatment within 72 hours after randomization.

  NOTE: For participants coenrolled in the Mechanistic Substudy (A5333s) and other specified approved REPRIEVE (A5332) ancillary studies with ancillary study procedures, treatment must be held until after the entry CCTA or relevant ancillary study procedure (see MOPS for more details) even if that occurs more than 72 hours but not more than 14 days after randomization.

6.2.3 Post-Entry Evaluations

On-Study Evaluations
All on-study evaluations must be scheduled as per section 6.1 with a ±7-day window for the month 1 visit and a ±30-day window for all other visits.

Participants who miss visits should be contacted to make up the visit and to permit determination of whether a major event occurred. See section 6.4 for additional considerations for endpoint assessments in the event of missed visits.

Return Fasting Visit (as needed)
This visit is only required for participants who are in a non-fasting state for visits that require fasted assessments. Participants should return for fasted assessments within 30 days for a fasting evaluation.

Study Termination Evaluation
The study termination evaluation will be completed as the participant's final on-study visit. **Please follow the Schedule of Evaluations (SOE) regarding data collection for the Study Termination Visit.**

Since REPRIEVE is an endpoint-driven trial, the study termination will be when the target number of endpoints has been reached or when determined by the DSMB at a later date. The study team will notify all sites of when to begin conducting the Study Termination Visit.

Event-Driven Evaluations
Evaluations must be scheduled as per section 7.0.

6.2.4 Discontinuation Evaluations

Evaluations for Registered or Randomized Participants Who Do Not Start Study Treatment
For participants who withdraw from the study before initiating study treatment, complete all eCRFs for the period up to and including the entry visit.

Premature Treatment Discontinuation Evaluations
Participants who discontinue the study treatment before the end of the study will have the premature treatment discontinuation evaluations completed within 30 days after stopping the study drug. At this visit, study drug will not be dispensed but adherence assessment will be performed for these individuals. After completion of the premature treatment discontinuation evaluations and until the study termination visit, participants will follow the clinical assessment and laboratory schedules as per section 6.1. Medication adherence will be assessed.

Premature Study Discontinuation Evaluations
Participants who prematurely discontinue from the study will have the premature study discontinuation evaluations performed as per section 6.1 prior to being taken off study.

NOTE: Sites will capture and document reasons for premature discontinuation (eg, related to AE), and whether premature discontinuation pertains to consent for follow-up and/or treatment.

For participants who prematurely discontinue from the study for reasons other than withdrawal of consent, sites will attempt to obtain information regarding vital status and endpoints annually (see section 6.3.10).

6.3 Instructions for Evaluations and Data Collection

Each study site and laboratory involved in this study will comply with the DAIDS policy on Requirements for DAIDS Funded and/or Sponsored Laboratories in Clinical

Trials Policy, which is available at https://www.niaid.nih.gov/sites/default/files/laboratorypolicy1.pdf.

All clinical and laboratory information required by this protocol is required to be maintained in the source documents (see MOPS for additional details). Sites must refer to the Source Document Guidelines on the DAIDS website for information about what must be included in the source document: https://www.niaid.nih.gov/sites/default/files/score-source-documentation-requirements.pdf.

All stated evaluations are to be recorded in the EDC unless otherwise specified. This includes events that meet the International Conference on Harmonization (ICH) definitions for a serious adverse event:

- Results in death
- Life-threatening
- Requires inpatient hospitalization or prolongation of existing hospitalization
- Results in persistent or significant disability/incapacity
- Congenital anomaly/birth defect
- Other important medical event (may not be immediately life-threatening or result in death or hospitalization but may jeopardize the patient or may require intervention to prevent one of the events listed above).

To grade diagnoses, signs and symptoms, and laboratory results, sites must refer to the DAIDS Table for Grading the Severity of Adult and Pediatric Adverse Events (DAIDS AE Grading Table), corrected Version 2.1, July 2017, is available on the DAIDS RSC website at https://rsc.niaid.nih.gov/clinical-research-sites/daids-adverse-event-grading-tables.

The protocol team and/or study monitoring entity may determine that additional source data associated with procedures or evaluations performed per protocol should be entered into eCRFs so that the data can be used for analysis or to otherwise assist with interpretation of study findings. In such cases, sites will be officially instructed to enter the additional data into eCRFs from available source documentation.

6.3.1 Documentation of HIV-1

Section 4.1.1 specifies requirements for HIV-1 documentation. HIV-1 documentation is not recorded in the EDC.

6.3.2 Medical History

The following diagnoses should be recorded in the EDC at screening and updated at entry, regardless of when the diagnosis was made:

- Hypertension
- Diabetes mellitus
- AIDS-defining events
- Any malignancy (exclusive of basal/squamous cell skin cancer)

- Prior history of dialysis or renal transplantation
- Chronic active hepatitis C
- Chronic active hepatitis B
- Venous thromboembolism (VTE)
- Pulmonary thromboembolism (PTE)
- Nadir CD4 cell count (verbal history accepted)
- **COVID-19**

Any allergies to any medications and their formulations must also be documented.

6.3.3 Medication History

A limited medication history must be performed at screening and updated at entry and post-entry visits as per Table 6.3.3-1 below. Record all modifications to ART, including participant-initiated modifications (more than 7 consecutive missed days), provider-initiated modifications, and permanent discontinuation. See Table 6.3.3-1 below and MOPS for more details.

Table 6.3.3-1: Medication History

Medication Category	Complete History or Timeframe	Record in EDC (Yes/No)
Antiretroviral therapy	Cumulative duration of protease inhibitor, thymidine analogs, abacavir, tenofovir, and overall ART duration	Yes
Current ART therapy	Current	Yes
Blinded study therapy (other than for REPRIEVE)	Current	Yes
Statin therapy	Any prior or current exposure	Yes
Nonstatin lipid lowering therapy	Current	Yes
Antidiabetic medications	Current	Yes
Aspirin therapy (ongoing regular therapy) and anticoagulant medications	Current	Yes
Antihypertensive medications*	Current	Yes
Antihepatitis medications	Current	Yes
Hormonal contraceptives or hormone replacement therapy*	Current	Yes
Testosterone therapy*	Current	Yes
**COVID-19 medications (including vaccines)***	**Any prior or current exposure**	**Yes**

*Refer to the MOPS for more information about recording these classes of drugs.

Study Treatment Modifications
The study drugs are pitavastatin and placebo for pitavastatin. Record all study drug modifications, participant-initiated and protocol-mandated modifications, inadvertent and deliberate interruptions of more than 7 consecutive days. Record any permanent discontinuation of treatment.

6.3.4 Clinical Assessments

Signs and Symptoms
At entry, all grades that occurred ONLY within the 30 days before entry are to be collected and maintained in the source documents but are not required to be recorded in the EDC at this time. If a participant develops future signs and symptoms, the signs and symptoms recorded in source documents at entry can be referred to, in order to see if they are truly incident or recurrent.

After entry, only the following signs and symptoms are to be recorded in the EDC:

- Signs and symptoms Grade ≥3.
- All signs and symptoms that led to a change in treatment (pitavastatin or placebo) regardless of grade.

NOTE: If signs and symptoms are related to a diagnosed event, record the primary event in the EDC and do not grade or record related signs and symptoms. If Grade ≥3 signs and symptoms occur independent of a related diagnosis, they should be recorded in the EDC.

Further evaluation will be required for those events that meet EAE or ICH reporting requirements.

Diagnoses
All incident diagnoses listed in section 6.3.2 will be assessed at all visits post entry; refer to section 6.4 for requirements for intervening medical history review related to primary MACE and other secondary serious non-cardiovascular events.

Assessment of Potential Myalgia Toxicity
Potential statin effects on the liver will be assessed with ALT at month 1 and month 12 visits; potential effects on muscle will be assessed by a myalgia symptom assessment at entry and onward.

Cardiovascular Risk Assessment Tool
The ACC/AHA 2013 10-year ASCVD risk score should be performed as part of screening. MOPS section 2.1, Atherosclerotic Cardiovascular Disease Risk Assessment Tool, provides detailed instructions concerning access and use of the 10-year ASCVD risk score calculator. It is important to use only the 10-Year ASCVD Risk Score, not the “risk with optimal risk factors” or the “lifetime ASCVD Risk calculator” results.

NOTE: For the purposes of calculation of the 10-year ASCVD risk score using the Pooled Cohort Equations:

- For participants whose values of HDL cholesterol, total cholesterol, and/or systolic blood pressure fall below or above the acceptable calculator bounds for those parameters, values at the lower or upper bounds, respectively, will be entered.
- Participants of mixed race will be asked to identify themselves as predominantly African American or predominantly other, and the race of predominant identification will be entered; as per calculator guidelines, non-African American race is entered as White or other race.
- Participants will be asked to report sex at birth, and this sex will be entered.
- *Only* participants currently on 1 or more antihypertensive medications will be counted as undergoing treatment for high blood pressure.
- *Only* participants who report current active smoking will be counted as smokers.

  NOTE: When calculating the ASCVD risk score, the use of e-cigarettes is not considered as current active smoking. Please mark “no” for persons who report only the use of e-cigarettes.

Cardiovascular Risk Factor Assessment
An assessment of general cardiovascular risk factors, current smoking, alcohol use, substance use, and family history of premature CVD. will be assessed at screen. **Hormonal/reproductive health assessments relevant to CVD risk will be assessed at screening and study termination visits.**

Diet and Functional Capacity Assessment
Diet (Rapid Eating and Activity Assessment for Patients [(REAP] questionnaire), functional capacity (Duke Activity Status Index [DASI] questionnaire), will be completed at entry, and **study termination** visits.

Screening Physical Exam
A screening physical examination is to include auscultation of the chest, cardiac exam, examination of the lower extremities for edema, and vital signs.

Targeted Physical Exam
A targeted physical examination is to include vital signs, and is to be driven by any previously identified or new signs or symptoms, including muscle aches, pains, tenderness, weakness, malaise, or fever. A targeted physical exam will also be driven by any diagnoses that the participant has experienced since the last visit. The targeted physical exam will occur at month 1 and each annual visit.

Height
Measurement of height will occur at screening.

Weight
Measurement of weight will occur at screening and each annual visit.

Waist Circumference
Measurement of iliac waist circumference will occur at screening and **study termination visits.** Please see MOPS for instructions on waist circumference measurement.

ECG
Resting 12-lead ECG results, including heart rate will be performed at entry. Please see MOPS for instructions.

6.3.5 Lifestyle Information

Participants will be provided information regarding healthy diet and activities in the Lipid-Lowering Diet, Activity Guide, and Smoking Cessation located on the REPRIEVE (A5332) PSWP. In addition, participants will be reminded to adhere to prescribed antiretroviral regimen and study medication. This is to be provided at entry and annual visits.

6.3.6 Laboratory Evaluations

All laboratory values collected for REPRIEVE (A5332) must be recorded in the EDC. **This includes laboratory values performed as part of toxicity management (eg, all CK assessments performed as part of management of myalgias and myopathy).**

Post entry, all Grade ≥3 laboratory values and all laboratory values regardless of grade that led to a change in treatment must be recorded as adverse events. Further evaluation will be required for those events that meet EAE or ICH reporting requirements.

Laboratory testing for screening or safety assessment must be performed in a CLIA or equivalent certified laboratory or at any network-approved non-US laboratory that operates in accordance with Good Clinical Laboratory Practices and participates in appropriate external quality assurance programs.

When labs are being captured from clinical care, the most recent lab value should be the value recorded in the EDC. Screening labs captured from clinical care should be within 90 days prior to entry unless otherwise indicated.
Fasting is defined as nothing to eat or drink except water and required prescription medications for at least 8 hours. Drinking black decaffeinated coffee without sweetener or creamer is permissible, but is not advised. Participants must be evaluated in a fasting state for those evaluations indicated as “fasting” unless otherwise specified. Participants will be asked whether they have fasted, and if not, they should be scheduled to return in a fasting state within 30 days to complete the fasting evaluations.

Participants should be instructed with the exact time beyond which they are to be fasting, such as: “Your visit is scheduled for 8:00 a.m. You should not have any food or drink by mouth except water and medication after 12 a.m.”

In order to minimize diurnal variation, fasting samples for individual participants should be obtained consistently in the morning, if possible. Participants will be encouraged to take the study drug consistently at the same time and this information will be collected in the medication adherence assessment.

<u>CD4+/CD8+</u>
Screen: Absolute CD4+/CD8+ count and percentages will be obtained from clinical care within 180 days prior to entry; if not available, draw as part of study.

Post-entry evaluations: CD4+/CD8+ obtained from clinical care since the last annual study visit. **For post-entry evaluations, all laboratories must possess a CLIA certification or equivalent (US sites) or IQA certification (non-US sites).**

NOTE: If CD8+ cell count is not available, CD4+ cell count alone is acceptable.

<u>Plasma HIV-1 RNA</u>
At all visits indicated on the Schedule of Evaluations, collect results of plasma HIV-1 RNA determinations that are obtained through clinical care. For the entry visit, a viral load from the prior 180 days should be recorded in the EDC. If not available, it will not be collected. For all post-entry evaluations, the most recent viral load obtained since the last annual study visit should be recorded in the EDC. **For post-entry evaluations, all laboratories must possess a CLIA certification or equivalent (US sites) or must be VQA certified (non-US sites).**

<u>Hematology</u>
At screening, hemoglobin, hematocrit, white blood cell count (WBC) and platelets will be captured from clinical care within 90 days prior to entry; if not available, draw as part of the study. At **study termination visits**, results must be within **180** days prior to that visit.

<u>Serum creatinine and calculated creatinine clearance/GFR</u>: at screening, creatinine result will be obtained from clinical care within 90 days prior to entry; if not available, draw as part of the study. At **study termination visits**, results must be within **180** days prior to that visit; **calculated creatinine clearance/GFR is not required at study termination visits**.

NOTE: See the A5332 MOPS for links to GFR and CrCl calculators.

<u>Fasting Lipid Panel</u>
Screen: Total cholesterol, HDL cholesterol, direct or calculated LDL cholesterol if triglycerides are ≤400 mg/dL (direct LDL must be determined if triglycerides are

>400 mg/dL and <500 mg/dL), and triglycerides will be collected from clinical care within 90 days prior to entry; if not available, draw as part of the study.

NOTE: If the screening lipid values to determine study eligibility are taken from existing clinical care assessments and are within the range specified by inclusion criterion 4.1.4, fasting status and documentation of fasting status are not required.

The screening lipid panel will be reviewed with the potential participant and shared with his or her medical provider along with the study rationale to confirm support of participant participation. Specimens from entry onward will be stored to be tested centrally. Participants may opt out of these entry and post-entry labs.

Liver Function Tests
Screen: ALT (SGPT) will be obtained from clinical care within 90 days prior to entry; if not available; draw as part of the study. Persons with HCV or HBV should also have AST (SGOT) obtained from clinical care within 90 days prior to entry to facilitate calculation of fibrosis by the FIB-4 equation.

At month 1 and month 12, ALT will be drawn as part of the study.

NOTE: Refer to the MOPS for link to the FIB-4 calculator.

Pregnancy Testing
For women with reproductive potential: serum or urine β-HCG (urine test must have a sensitivity of <25 mIU/mL) will be performed as part of the study at each visit and whenever pregnancy is suspected. Record pregnancy and pregnancy outcome. Refer to the MOPS for guidance.

Fasting glucose at entry and onward will be performed as part of the study. Specimens will be tested centrally. Participants may opt out of these entry and post-entry labs.

NOTE: Fasting is defined as above (see 6.3.6 for Fasting Lipid Panel).

Urine Albumin/Creatinine Ratio
Urine for urine albumin/creatinine ratio will be collected from all participants at entry. Specimens will be tested centrally.
ACTG sites will collect urine at subsequent visits per section 6.1 only from participants who enrolled in REPRIEVE (A5332) prior to 01/01/2018. These specimens will be tested centrally.

6.3.7 Immunologic, Hormonal, and Biomarker Studies

Additional serum and plasma will be batched and stored in the ACTG Specimen Repository for analyses by a central core laboratory. Refer to the Laboratory Processing Chart (LPC) for details. Participants may opt out of these entry and post-entry labs.

6.3.8 Stored Whole Blood for Future Genetic Studies

A single whole blood sample will be obtained from all study volunteers for human genotyping of selected polymorphisms that may predispose to CVD or alter pitavastatin levels or effectiveness. This sample will be drawn at ACTG sites only and will be collected in addition to any samples collected for participants in A5128 or A5243.

6.3.9 Medication Adherence Assessment

Site personnel will perform an assessment of adherence to study medication at every visit after the entry visit.

6.3.10 Vital Status and Endpoint Follow-up

For participants who prematurely discontinue study for reasons other than withdrawal of consent, site personnel will attempt to obtain information regarding vital status (including date last seen alive, date of death, and primary cause of death) and endpoints (see sections 6.4.1-6.4.2) from participant or other sources (such as family members, other designated contacts, or clinic records) annually from the time off study. Please see MOPS for additional information.

6.4 Endpoint Assessments

At entry and onward, a comprehensive review of the intervening medical history of the participant will be performed to capture the data for adjudication of the primary and secondary endpoints, including diagnoses and any hospitalization. Participants will be specifically asked about hospitalization, emergency room visits, urgent care visits, physician visits, and any symptoms that are suggestive of **CVD**. Information regarding potential events will be recorded in the EDC and via source documents and prepared for transmission after de-identification to the Clinical Event Committee (CEC) for adjudication, as appropriate. Participants who miss visits will be contacted to make up the visit and to permit determination of whether major event occurred, the assessment will be completed using other sources such as designated contact, physician contact, or medical records (refer to the MOPS for details). Events will be reviewed and categorized by a CEC unaware of the treatment assignment based on established definitions described below.

Non-CVD events as described below will be assessed but will not be formally adjudicated by the CEC.

6.4.1 Definitions of Major Adverse Cardiovascular Events (MACE) Components

The primary endpoint of Major Adverse Cardiovascular Events (MACE) includes the composite of Cardiovascular Death, Myocardial Infarction, Hospitalization for Unstable Angina, Coronary, Carotid or Peripheral Arterial Revascularization, Transient Ischemic Attack, Stroke, and Peripheral Arterial Ischemia.

Formal definitions for each of these components and additional secondary and exploratory endpoints are contained within the CEC charter, and are based on the Standardized Definitions for Cardiovascular and Stroke End Point Events in Clinical Trials [Hicks 2015].

General principles guiding these definitions are described below.

CVD death: includes death resulting from an acute myocardial infarction (MI), sudden cardiac death, death due to heart failure (HF), death due to stroke, death due to cardiovascular (CV) procedures, death due to CV hemorrhage, and death due to other CV causes.

Myocardial infarction: The diagnosis of MI requires the combination of: evidence of myocardial necrosis (either changes in cardiac biomarkers or post mortem pathological findings); and supporting information derived from the clinical presentation, electrocardiographic changes, or the results of myocardial or coronary artery imaging suggesting an event consistent with coronary ischemia.

The totality of the clinical, electrocardiographic, and cardiac biomarker information will be considered to determine whether or not an MI has occurred. Specifically, timing and trends in cardiac biomarkers and electrocardiographic information will be included whenever possible, but the diagnosis can still be determined if these results are not available.

Unstable angina hospitalization: ischemic discomfort or equivalent requiring hospitalization within 24 hours with objective signs of coronary ischemia in absence of MI. ECG, angiographic and imaging criteria will be considered.

Coronary, carotid, or peripheral arterial revascularization: invasive percutaneous or surgical procedure intended to restore or improve blood flow in a coronary or peripheral artery including but not limited to angioplasty, stent, stent graft, or bypass graft.

Stroke: an acute episode of focal or global neurological dysfunction caused by brain, spinal cord, or retinal vascular injury as a result of hemorrhage or infarction.

Transient Ischemic Attack: a transient episode of focal neurological dysfunction caused by brain, spinal cord, or retinal ischemia, without stroke.

Peripheral Arterial Ischemia (PAD): Peripheral arterial ischemia hospitalization: Urgent hospitalization for insufficiency of the peripheral arterial circulation, including but not limited to, acute limb ischemia, chronic limb ischemia, amputation, or other vascular abnormality of an ischemic and noninfectious nature.

6.4.2 Causes of Death

In addition to CVD death which is a component of the primary endpoint, all deaths will be adjudicated by the CEC to determine likely cause.

6.4.3 Cardiac Events to be adjudicated but not included in REPRIEVE (A5332) MACE Definition:

- Heart Failure**, including all COVID-19-related events resulting in hospitalization** (see CEC Charter)

6.4.4 Non-CVD Events

Clinical assessments will also include assessing the participant regarding the occurrence of the following diagnoses:

- AIDS-defining events
- Non AIDS defining cancers (except squamous/basal cell of the skin)
- End stage kidney disease, requiring initiation of dialysis or renal transplantation
- End stage liver disease (incident cirrhosis or hepatic decompensation requiring hospitalization)
- Incident diabetes mellitus requiring use of diabetes medications
- Venous thromboembolism (VTE)
- Pulmonary thromboembolism (PTE)

For diagnostic criteria for these non-CVD events, refer to the MOPS. All identified events should be reported in the EDC regardless of grade. These events do not undergo CEC adjudication.

7.0 CLINICAL MANAGEMENT ISSUES

Only toxicities related to the study drug (pitavastatin and placebo for pitavastatin) are subject to the guidelines outlined in the toxicity management section. In the case of multiple toxicities or AEs, the guidelines pertaining to the most severe event should take precedence.

The grading system is located in the DAIDS Table for Grading the Severity of Adult and Pediatric Adverse Events (DAIDS AE Grading Table), corrected Version 2.1, July 2017, is available on the DAIDS RSC website at https://rsc.niaid.nih.gov/clinical-research-sites/daids-adverse-event-grading-tables.

Please refer all questions to the REPRIEVE (A5332) core protocol team via email.

7.1 Toxicity Management

7.1.1 General Reactions

Grade 1 or 2
Participants who develop a Grade 1 or 2 toxicity may continue study treatment.

Grade 3
Participants who develop a Grade 3 toxicity that is judged by the site investigator to be study drug-related should have the study drug held and the study team should be consulted. The participant should be followed closely and if the toxicity does not return to Grade ≤2 within 2 weeks, the study drug must be permanently discontinued with participant evaluations as per section 6.2.4.

If the study drug is resumed and the same Grade 3 toxicity recurs within 4 weeks of reintroduction, and the site investigator considers this AE related to the study drug, the drug must be permanently discontinued.

With a Grade 3 toxicity that is judged not related to the study drug by the site investigator, the study drug may be continued at the discretion of the site investigator in consultation with the study team.

Grade 4
Participants who develop a Grade 4 toxicity will have the study drug held and the study team should be consulted. Participants experiencing Grade 4 toxicities should be followed closely with additional clinical assessments and laboratory testing as clinically indicated.

If the investigator feels that the toxicity is clearly related to another cause and that the toxicity is not caused by the study drug, and after consultation with the study team, dosing may continue.

Otherwise, if the toxicity does not return to Grade ≤2 within 2 weeks, the study drug must be permanently discontinued with participant evaluations as per section 6.2.4.

NOTE: Direct and indirect bilirubin elevations that reach Grade 4 elevations according to the DAIDS AE Grading Table and are related to atazanavir are excluded from reporting.

7.1.2 ALT Elevations

ALT levels will be routinely evaluated at visits at month 1 and month 12. All other evaluations of ALT will be performed at the discretion of the site investigator based on participant symptoms.

Grade 3

Participants who develop *asymptomatic* >5 x ULN ALT elevations (Grade 3), study drug should be held for 1 week and the individual should be re-evaluated 1 week after drug discontinuation. If at that time the ALT elevation is ≤5 x ULN and participants remain asymptomatic, the participants are eligible to continue on study treatment at the discretion of the site investigator. If the ALT does not return to ≤5 x ULN within the 1-week period, the study drug must be permanently discontinued unless the ALT elevations are deemed not related to study drug upon further assessment as per the discretion of the PCP (ie, acute hepatitis A or other clear causation).

For any *symptomatic* (eg, fatigue, nausea and vomiting, right upper quadrant pain, rash or eosinophilia) ALT >5 x ULN (Grade 3), study drug should be held. Participants should be asked to return to the research site for repeat testing 1 week later. If repeat ALT is ≤3 x ULN and the participant is no longer symptomatic, study drug can be resumed.

Grade 4

For any ALT >10 x ULN (Grade 4), study drug should be discontinued. The participant should be brought back for repeat testing every 1 week until the ALT ≤5 x ULN.

NOTE: If the Grade 3 or 4 elevation is clearly related to another cause and not related to study drug (eg, acute hepatitis A infection), the participant should be brought back for repeat testing every 2 weeks for Grade 3 or every 1 week for Grade 4 until the ALT ≤3 x ULN. The local site investigators should contact the study team for approval before resuming study drug. Participants who permanently discontinue study drug will be followed on study, off treatment through the study termination visit with participant evaluations as per section 6.2.4.

NOTE: For those participants with ALT >3 x ULN upon repeat testing, whether symptomatic or not, AST, alkaline phosphatase, and total bilirubin or INR, should also be performed as part of the study to help determine etiology of increased LFTs. For participants on atazanavir, performance of INR rather than bilirubin is preferred. Other labs including hepatitis serologies may also be indicated and performed in the context of clinical care by PCP.
NOTE: If the participant has recurrent elevations of ALT >3 x ULN but the site investigator deems the elevation not related to study drug, the site investigator must contact the study team to discuss continuation of study drug.

Abnormal ALT determinations occurring in the course of clinical care should be repeated by the treating clinician. Persistently abnormal values which would trigger the toxicity guidelines above should be reported to the REPRIEVE (A5332) team. If the ALT abnormality is not due to another cause, eg, acute hepatitis, the REPRIEVE (A5332) site PI will follow the toxicity algorithm above.

7.1.3 Myalgias and Myopathy

Persons who present with significant myalgias (Grade ≥3, ie, muscle pain causing inability to perform usual social and functional activities) should be evaluated with a clinical assessment that includes an evaluation of CK, serum creatinine, potassium, and urinalysis. Myopathy is defined as muscle aches, soreness, tenderness, or weakness with CK >10 x ULN not related to exercise or other causes, including trauma. If the symptoms are associated with Grade ≥3 elevation in CK (10 x ULN) (See Table 7.1.3-1 below) that is not related to exercise or other cause, study medications should be permanently discontinued. Participants will be followed on study, off treatment through the study termination visit with participant evaluations as per section 6.2.4.

NOTE: Mitochondrial toxicity related to nucleoside therapy and not related to study medication is a possibility, and evaluations for lactic acidosis should be considered by the participant's primary care provider.

Table 7.1.3-1: Serum CK Toxicity Grading*

Toxicity Grade	Value
Grade 1	3 – <6 x ULN
Grade 2	6 – <10 x ULN
Grade 3	10 – <20 x ULN
Grade 4	≥20 x ULN

*Not related to exercise or other cause

7.1.4 Rhabdomyolysis

Rhabdomyolysis is defined as the presence of myopathy as per section 7.1.3 plus one or more of the following:

- Hematuria on urine dipstick in the absence of microscopic hematuria (myoglobinuria)
- Grade ≥2 hyperkalemia
- Grade ≥2 creatinine elevation

If rhabdomyolysis occurs, study medications should be permanently discontinued. The team should be consulted. Participants will be followed on study, off treatment through the study termination visit with participant evaluations as per section 6.2.4. In addition, CK will be added to the laboratory evaluations performed until it has declined to ≤1 x ULN.

7.2 Requirement for Precautionary or Prohibited Medications (see PSWP)

Participants who need to initiate therapy with erythromycin, colchicine, systemic (oral or intravenous) cyclosporine, or rifampin should be asked to hold study drug. If use of one of these precautionary medications is anticipated to be short-term, the site investigator may consider restarting study drug after use of prohibited medication is discontinued.

Participants who begin a statin medication provided through clinical care should discontinue study drug. Taking two statins can increase the risk of toxicity.

Participants who temporarily or permanently discontinue study treatment will be followed on study, off treatment through the study termination visit with participant evaluations as per section 6.2.4.

7.3 Pregnancy

If the pregnancy test is positive at entry, then the participant should not start study treatment. No further evaluations are necessary, provided that the participant did not initiate study drug.

Participants who become pregnant after study entry must discontinue study treatment immediately. These participants should be seen for a premature treatment discontinuation evaluation within 7 days. Participants will be followed on study, off treatment through the study termination visit with participant evaluations as per section 6.2.4. The core team must be notified of any pregnancies that occur in participants on study. Management of the background ART is at the discretion of the site investigator.

All pregnancies should be followed until the final outcome can be determined. In the event that the pregnancy has not been completed by the final study visit, the site should contact the participant through monthly phone calls and review of medical records, if possible, until the pregnancy outcome can be ascertained. See the MOPS for guidance on documenting pregnancy outcomes.

Pregnancies that occur on study should be reported prospectively to The Antiretroviral Pregnancy Registry. More information is available at www.apregistry.com. Phone: 800-258-4263; Fax: 800-800-1052 (Non-US sites: Fax: 44-1628-789-666 or 910-246-0637; phone: 910-679-1598.)

7.4 Unblinding Procedures

For unblinding requests, including emergency unblinding, refer to the ACTG Unblinding Participants Standard Operating Procedure (SOP) 123 at
https://member.mis.s-3.net/cms/dl/10466
Note: Unblinding is rarely allowed as study medications can most often be withdrawn in a participant experiencing adverse effects without the need for unblinding. Any decision on unblinding should be made with reference to the ACTG SOP.

In the event that emergency disclosure of treatment assignment is thought to be required, the site investigator must follow the ACTG Unblinding Participants SOP 123.

The protocol chairs and DAIDS Medical Officer will be notified of such a request through the ACTG DMC unblinding program. All site e-mails to the team should be carefully worded to prevent unblinding the team, if possible.

Unblinding of all study participants will take place after the last participant has completed the study, all data have been entered into the database and cleaned for primary and secondary endpoints, and MACE endpoint verification is complete. The time necessary to finalize the data can be up to 3 months or more after study closure.

8.0 CRITERIA FOR DISCONTINUATION

8.1 Premature and Permanent Treatment Discontinuation

- Participant refusal to continue study treatment.
- Drug-related toxicity per section 7.0.
- Clinical reasons believed life threatening by the physician, even if not addressed in section 7.0.
- Pregnancy or breast-feeding.
- Use of prohibited and some precautionary medications per section 7.0. See PSWP for precautionary and prohibited medication list for further information. Please contact the study team if you have questions.

NOTE: Participants who permanently discontinue study treatment will be followed on study, off treatment through the study termination visit with participant evaluations per section 6.2.4.

8.2 Premature Study Discontinuation

- Refusal by the participant of further study follow-up.
- Request by the participant to withdraw consent.
- Request of the primary care provider if s/he thinks the study is no longer in the best interest of the participant.
- At the discretion of the ACTG, IRB/EC, Food and Drug Administration (FDA), Office for Human Research Protections (OHRP), NHLBI, NIAID, other government agencies as part of their duties, investigator, or pharmaceutical supporter.

If a participant misses clinic visits for >1 year, it is at the discretion of the site PI whether or not to discontinue the participant from study participation. Participants who prematurely discontinue from the study will have the premature study discontinuation evaluations performed as per section 6.1 and then be taken off study.

In the event that a participant prematurely discontinues from the study, unless they have withdrawn consent, sites will attempt to obtain information regarding vital status (including date last seen alive, date of death, and primary cause of death) and MACE events from other sources (such as family members, other designated contacts, or clinic records) per section 6.2.4.

9.0 STATISTICAL CONSIDERATIONS

9.1 General Design Issues

REPRIEVE (A5332) is a prospective, randomized, double-blind, placebo-controlled, phase III study of the effect of pitavastatin on major cardiovascular events in **PWH** who do not meet current guidelines for statin therapy. The study was originally designed to enroll 6500 participants **with HIV** on stable ART and not eligible for statins as per the 2013 ACC/AHA guidelines. Enrollment was anticipated to take 30 months with study follow-up continuing for approximately 72 months after the enrollment of the first participant. Based on recommendations of the REPRIEVE (A5332) DSMB following their December 2017 review, the study sample size was increased to approximately 7500 participants with follow-up continuing for approximately 84 months after the enrollment of the first participant. Based on recommendations of the NIH and the REPRIEVE DSMB following their December 2018 review, follow-up was extended to 96 months. **Follow-up will now continue until the study reaches the targeted 288 primary MACE events or is otherwise recommended for closure by the DSMB.**

Further, a cap of approximately 2000 participants total enrollment of individuals with very low cardiovascular risk (estimated 10-year ASCVD risk score <2.5%) was implemented to ensure that the final study population reflects the targeted low-to-moderate-risk population. Subsequent to a new recommendation from the NIH, the total enrollment of individuals with low cardiovascular risk (10-year ASCVD risk score <5.0%) was capped at approximately 4200 participants, to ensure that the final study population reflects the targeted low-to-moderate risk population.

All primary analyses will be performed as intention-to-treat and include all participants as randomized.

A complete firewall will be maintained to ensure that investigators have no access to the data. All data will be kept on secure systems at Frontier Science & Technology Research Foundation (FSTRF). A complete description of the firewall procedures, data organization, and security **is** included in the final statistical analysis plan. The plans and procedures will be consistent with NHLBI and NIAID policy with respect to maintenance of data integrity.

Ancillary studies are investigations that are not part of REPRIEVE (A5332), but that propose questions and test hypotheses that are relevant to and further the goals and purposes of REPRIEVE (A5332). It is recognized that well-designed ancillary studies, consistent with the goals of REPRIEVE (A5332), can leverage the trial's resources to provide critical answers to highly relevant questions for the field. Successful ancillary studies should have secured adequate funding through peer review, through either the ACTG, NIH, or other funding sources. To protect the integrity of REPRIEVE (A5332), ancillary study proposals are reviewed and approved by the REPRIEVE Ancillary Studies Committee and the DSMB prior to implementation. In addition to meeting the standard for high scientific merit, the major criterion for approval of an ancillary study is that it does not negatively affect the conduct of the parent REPRIEVE trial.

For more details regarding the ancillary studies approved for implementation as part of REPRIEVE (A5332), please see Appendices IV and V.

9.2 Outcome Measures

For all time to event study endpoints, time will be measured from the date of randomization to the onset date of the event of interest.

9.2.1 Primary Endpoint

The primary endpoint will be time to the first event of a composite of major cardiovascular events **(primary MACE)** including:

- Atherosclerotic or other CVD death
- Nonfatal myocardial infarction
- Unstable angina hospitalization
- Coronary or peripheral arterial revascularization
- Nonfatal stroke or TIA
- Urgent PAD ischemic event (acute or chronic limb ischemia, amputation, etc.)

All primary events will be prospectively determined and adjudicated by an expert Clinical Events Committee based on standardized criteria used in prior cardiovascular trials and developed by consensus groups and the FDA [Hicks 2015] (see section 6.4.1). **Deaths adjudicated as of undetermined cause will be included as primary MACE endpoints.**

Participants discontinuing follow-up without experiencing the event will be considered censored at the time of their contact at which an assessment for primary endpoints was made; deaths from **known** non-CVD causes will be treated as competing risk events in the primary analysis. See section 9.6 for additional supportive analyses.

9.2.2 Supportive and Secondary Endpoints

9.2.2.1 Time to the first of each individual component of the primary endpoint.

For each event, participants discontinuing follow-up without experiencing the event will be considered censored; deaths from other causes will be considered competing risk events.

9.2.2.2 Time to death (all-cause mortality)

Based on independent review, death will be classified as cardiovascular event or non-cardiovascular event. Non-cardiovascular events will be further characterized as HIV-associated clinical diagnosis, non-AIDS malignancy, accidental, suicide, homicide, other sudden death of unknown etiology, or other. See Statistical Analysis Plan (SAP) for further details.

9.2.2.3 Time to death (all-cause mortality) and/or MACE

Participants discontinuing follow-up without experiencing the event (death from any cause or MACE) will be considered censored. A supportive outcome measure including data from vital status and endpoint follow-up (see section 6.3.10) will also be conducted. See SAP for further details.

9.2.2.4 Time to any (composite) or each (individual) of the following incident clinical diagnoses (including recurrent diagnoses as appropriate)

- Non AIDS-defining cancers (excluding basal cell and squamous cell carcinomas of the skin)
- AIDS-defining events (based on CDC 2014 classification)
- Initiation of dialysis or renal transplantation.
- Cirrhosis, or hepatic decompensation requiring hospitalization

For specific case definitions, see MOPS.

For each event, participants discontinuing follow-up without experiencing each event will be treated as censored; deaths from other causes will be treated as competing risk events.

9.2.2.5 Calculated fasting LDL and non-HDL cholesterol level at study entry and annually thereafter as well as change from baseline expressed as absolute change and as a percentage of baseline. For participants with triglycerides >400 mg/dL and <500 mg/dL, direct LDL will be determined and use in the statistical analysis.

9.2.2.6 Time to any of the following adverse events (including recurrent events as appropriate)

- Serious adverse event as defined by ICH criteria
- Incident Diabetes mellitus (DM)
- Grade 3 or 4 ALT
- Grade 3 or 4 myopathy
- **COVID-19 diagnosis**
- **Serious COVID-19 diagnosis (ie, a COVID-19 diagnosis that is Grade 4 or results in hospitalization or death)**

All events will be included regardless of relationship to treatment as determined by sites.

Grading will be defined per the DAIDS Table for Grading the Severity of Adult and Pediatric Adverse Events, corrected Version 2.1, July 2017. See section 11.4 for link to the document.

For COVID-19 outcomes, time will be measured from January 1, 2020.

9.2.2.7 Antibody-positive COVID-19 infection assessed cross-sectionally at specific calendar time points from samples collected annually.

9.2.3 Exploratory Endpoints

9.2.3.1 Fasting Total and HDL cholesterol and LDL-C/HDL-C ratio at study entry and annually thereafter.

9.2.3.2 Time to heart failure

9.3 Randomization and Stratification

At study entry, participants will be assigned with equal probability to one of the two treatment arms. Randomization will use permuted blocks with stratification by sex (male/female) and screening CD4+ T-cell counts (≤500 vs. >500 cells/mm^3). Additionally, to ensure balanced treatment allocation for the Mechanistic Substudy of REPRIEVE (A5333s), randomization will also be stratified by whether or not a participant has elected to participate in the mechanistic substudy (yes/no). Total enrollment of individuals with low cardiovascular risk (10-year ASCVD risk score <5.0%) was capped at approximately 4200 participants, to ensure that the final study population reflects the targeted low-to-moderate risk population. It is anticipated that the study will take 48 months to fully enroll.

9.4 Sample Size and Accrual

The original target sample size of REPRIEVE (A5332) was 6500 individuals. This was increased by approximately 1,000 participants or to approximately 7500 following the December 2017 DSMB. Sample size considerations for the original target are provided below. Additional considerations providing justification for the increased sample size and duration of follow-up are also provided.

9.4.1 Original Sample Size Considerations

The original sample size for the main study was determined to provide 90% power to detect a 30% reduction in the composite CVD endpoint with statins (statin effectiveness equating to Hazard Ratio (HR) of 0.70). This desired effect size equates to a 5-year number needed to treat of 47. The effect size is more than the 22% based on LDL reduction alone seen in the **Cholesterol Treatment Trialists'** collaboration [**Cholesterol Treatment Trialists' (CTT)** Collaboration 2010], consistent with the hypothesis of REPRIEVE (A5332), that statins will have an effect beyond LDL lowering. The HR is a more conservative effect size than was observed in JUPITER (a primary CVD prevention trial in **people without HIV**)—a**n** HR of 0.56 or 44% reduction in the composite endpoint [Ridker 2008]. This more modest clinical effect is felt justified based on the higher

absolute risk of CVD outcomes in **PWH** as well as some anticipated cross-over between the two study groups.

Assuming a fixed sample size of 6500 individuals and total follow-up of 6 years, Table 9.4.1-1 shows the power under a range of alternative scenarios. For example, under the baseline assumption of an event rate of 15/1000 PY and accrual duration of 2.5 years, the sample size of 6500 participants will ensure 90% power to detect a 30% reduction in event rates (HR = 0.70; 5-year NNT = 47). However, with slower accrual (2.75 yrs) and lower event rates (14/1000 PY) REPRIEVE (A5332) will still have adequate power at 89% and 88%, respectively. Moreover, while power will be reduced to 83% in the case of severely reduced event rates of 12/1000 PY and a slower accrual rate of 2.75 years, contingencies to extend REPRIEVE (A5332) for a total follow up of 6.5 years while remaining within the scope of the current budget, will ensure a reasonable 85% power.

Table 9.4.1-1: Power to Detect Given Hazard Ratio (HR) with Total Sample Size of 6500 Based on 2.5 Years of Accrual and 6 Years Maximal Follow-up

Design Assumptions								Power to Detect Given HR		
Control rate of MACE	5y event rates				5y NNT for given HR					
	Control	Statin (for given HR)								
		0.65	0.70	0.75	0.65	0.70	0.75	0.65	0.70	0.75
18/1000 PY	8.7%	5.7%	6.1%	6.5%	34	39	48	99%	94%	83%
15/1000 PY	7.3%	4.8%	5.1%	5.5%	41	47	57	97%	90%	76%
12/1000 PY	5.8%	3.8%	4.1%	4.4%	50	58	70	93%	83%	67%

The assumptions and justification underpinning the original REPRIEVE (A5332) study sample size are as follows:

<u>*A composite CVD event rate of 15/1000 PY in the absence of statin therapy:*</u>
Event rates for a similar composite endpoint were queried in the Partners Research Patient Data Registry (RPDR) for individuals ≥ 40 years of age, and determined for 3,213 thousand PWH and 26,309 thousand **people without HIV** matched on age/gender/race followed over 10 years from 2000-2009 (14,942 person-years for **PWH** and 106,853 person-years for non-HIV). Event rates were 21/1000 PY in **PWH** and 15/1000 PY in the non-HIV group. Further refining the Partners database query for incident MI, stroke, angina, revascularization among **PWH** ≥40 years, without diabetes mellitus and no recent history of statin use demonstrated (in alignment with the LDL independent definition of potentially eligible participants for REPRIEVE (A5332)) a MACE rate of 13/1000 PY. Since CVD death could not be obtained as part of this query, this rate likely underestimates the rate that could be expected in REPRIEVE (A5332). In JUPITER, CVD death represented 20% of all MACE. A similar adjustment would bring the HIV CVD event rate in Partners to 16.2/1000 PY. The placebo rate for MACE in JUPITER, among **people without HIV** was 13.6/1000 PY. The assumed event rate of 15/1000 PY for REPRIEVE (A5332) was felt to be a reasonable compromise between these rates.

Enrollment will be completed in 30 months: Given the total study duration of 6 years after enrollment has begun, individual participant follow-up will range from 3.5 to 6 years, with a median follow-up of 4.75 years. REPRIEVE (A5332) will be conducted at approximately 100-130 sites including the majority of all domestic ACTG sites, and selected non-US ACTG sites as well as protocol specific sites. The assumed enrollment period of 30 months requires an average enrollment rate of 2.2 participants per site per month. Given a staggered rate of site activation, actual site enrollment will need to be between 3 to 4 participants per site per month. This enrollment rate is consistent with enrollment rates observed in recent large ACTG trials and with the expectations of the study sites based on a site survey. Further, a site survey conducted in early 2014 has suggested broad availability of the target study population. As of May 29, 2014, of sites queried, a total of 153 sites have expressed an interest in participating in REPRIEVE (A5332) and estimated being able to enroll 10,551 out of an estimated pool of 46,395 eligible participants.

An annual 5% loss to follow-up rate: Since 1999, the ACTG has actively followed a large cohort of individuals on a limited visit schedule. During that time, the annual rate of lost to follow up was observed to be around 5.6%. We believe that we will be able to achieve a rate lower than this in REPRIEVE (A5332) since participants may also be receiving active treatment. Indeed, while this conservatively high estimate maintains power of study even in the event of 25% rate of loss over the duration of the study, it is desired that the observed rate will be no more than 15% over 6 years.

Estimated treatment cross-over rates of 10%: REPRIEVE (A5332) is powered to detect a statin effectiveness equating to a HR of 0.70. The influence of treatment switching (crossover) on the effectiveness of statin treatment for the prevention of MACE and impact of this crossover on the power of the REPRIEVE (A5332) study to detect a statin benefit as currently designed was broadly assessed via a simulation study. Briefly, clinical trials were simulated according to the REPRIEVE (A5332) design and analysis considerations. Simulated MACE times were accelerated and decelerated for discontinuation or initiation of statin treatment (for active and placebo groups respectively) over a range of crossover rates that were varied according to underlying MACE risk—simulated cases in the placebo group of higher underlying CVD risk as well as those in the statin group with the lowest CVD risk were assumed to have the highest rate of crossover. Average statin effectiveness over 6 years was estimated based on uncensored follow-up over >3,000,000 simulated cases. Under the base case, an overall rate of crossover of 10% resulted in an estimated statin effectiveness at the target of 0.70 for an underlying statin efficacy of 0.66. For the same statin efficacy, a 16% overall rate of crossover was associated with an estimated statin effectiveness of 0.74.

9.4.2 Design Considerations following December 2017 and 2018 DSMB Reviews

In follow-up to their December 2017 review, the REPRIEVE (A5332) DSMB recommended the following actions: 1) to cease study enrollment of patients with

10-year ASCVD risk score less than 2.5%; 2) increase study sample size by approximately 1,000 participants of higher risk population; and 3) increase the follow-up by 1 year now and consider re-evaluation of an additional follow-up extension in the future [implemented following the December 2018 DSMB Review]. These recommendations respond to the higher than anticipated enrollment of participants with very low risk and ensure that REPRIEVE (A5332) will maintain power to detect the targeted effect of interest under a broader range of scenarios for the rate of MACE in the absence of a pitavastatin (control rate). **Specifically, a total of 288 primary MACE endpoints are needed to ensure that the study will have 85% power to detect a hazard ratio of 0.7**.

9.4.3 Power Considerations for non-MACE Clinical Endpoints

Since the composite rate of serious non-MACE of interest (see section 9.2.2.4) in the absence of statin is expected to exceed the rate of MACE, REPRIEVE (A5332) will be well powered to detect statin effectiveness equating to a 25% reduction in non-MACE or higher.

If the incidence of any specific class of non-MACE in the absence of statin therapy is 5/1000 PY or higher, REPRIEVE (A5332) will have 90% power to detect a statin effect equating to a 50% reduction in the non-MACE event rate or higher. Published data from the ALLRT cohort [Overton 2013] suggest that the incidence of each of these events of interest will exceed this 5/1000 PY threshold.

9.4.4 Power Considerations for COVID-19 Objectives

In March 2020, when SARS-CoV-2 infections and COVID-19 diagnoses were emerging in REPRIEVE enrollment areas, approximately 6650 REPRIEVE participants remained in follow-up.

It was hypothesized that <u>30% of REPRIEVE participants will be antibody positive for exposure to SARS-CoV-2</u>. This rate is considered a conservative estimate based on widespread successful implementation of mitigation strategies. Recent studies from NYC and other large US urban areas indicate infection rates of up to 30%, and this is anticipated to increase further, especially in less-developed Global Burden of Disease regions that recruited into REPRIEVE. With the use of comprehensive sensitive and specific state of the art antibody testing, as proposed in this supplement, we will be well-poised to capture a significant number of cases, independent of any differences in more localized testing rates.

Allowing for 5% additional losses to follow-up, this will yield approximately 2000 cases and 4650 controls for analyses, including about 200 hospitalizations (10% of infections, ie, serious disease), and will provide ample numbers for the proposed epidemiology, pathobiology, genetic, and clinical investigations (see Table 9.4.4-1). For example, with this rate of

antibody positivity, we will be able to estimate infection risk with a precision of at least ±1.2%, and will have 90% power to show a 12% reduction in the infection risk in the pitavastatin group (RR=0.88, 30% risk in the placebo group compared to 26% risk in the pitavastatin group), and 80% power to detect a 35% relative risk reduction (RR=0.65) in serious disease (see table cells in blue font). Detectable effect sizes for antibody positivity rates of 15%-30% and power of 80%-90% are shown in the table below.

Table 9.4.4-1: Detectable Effect Sizes for Antibody Positivity Rates of 15%-30% and Power of 80%-90%

	Power	Proportion of Participants COVID-19 Antibody Positive (Control)					
		30%		20%		15%	
Precision to estimate infection risk	-	±0.012%		±0.0135%		±0.015%	
Detectable risk reduction with given power	90%	12% (RR=0.88)		15% (RR=0.85)		18% (RR=0.82)	
	85%	11% (RR=0.89)		14% (RR=0.86)		17% (RR=0.83)	
	80%	10% (RR=0.90)		13% (RR=0.87)		16% (RR=0.84)	
		Proportion of COVID-19 antibody positives experiencing serious disease					
		10%	20%	10%	20%	10%	20%
Detectable risk reduction with given power	90%	40% (0.60)	29% (0.71)	48% (0.52)	35% (0.65)	55% (0.45)	40% (0.60)
	85%	38% (0.62)	27% (0.73)	45% (0.55)	33% (0.67)	51% (0.49)	38% (0.62)
	80%	35% (0.65)	25% (0.75)	43% (0.57)	31% (0.69)	48% (0.52)	35% (0.65)
* Assumes sample size of 6650 with evaluable COVID-19 antibody results equally distributed between pitavastatin and placebo groups (see text for rationale for this number).							

9.5 Monitoring

The following is a summary of the data and safety monitoring plan for REPRIEVE (A5332). Prior to enrollment of the first participant, a detailed study monitoring and analysis plan document will be prepared that will more fully describe these data monitoring aspects including timelines and responsibilities for preparation.

Summaries of accrual rates, deaths, SAEs, and targeted AEs across regimens as well as study conduct (in terms of off-study rates, and completeness of study visits) will be reviewed on a regular basis by the protocol core team with all data pooled across study arms. Further, in line with the NHLBI Accrual Guidelines of observed against target accrual benchmarks will be reviewed by NHLBI program staff at a minimum of 25%, 50%, and 75% of the anticipated accrual period. Additional accrual reporting to NHLBI will be provided as requested. The timing and anticipated enrollment by 25%, 50%, and 75% of the accrual period are shown in Table 9.5-1.

Table 9.5-1: Accrual Benchmarks at 25%, 50%, and 75% of the Accrual Period

Recruitment Period	Time after First Patient Enrollment	Projected Accrual
25%	7.5 months	767
50%	15 months	2740
75%	22.5 months	4635

The study will undergo at least annual review by an NIH appointed DSMB for study conduct, continued feasibility, safety, and efficacy.

Unless otherwise noted, the unblinded REPRIEVE (A5332) statisticians will be responsible for all data analysis and report preparation to the DSMB. The DSMB will be appointed by NHLBI in consultation with DAIDS. For each review, the statisticians will prepare 3 summary reports: 1) a closed report containing all information broken down by masked treatment group distributed only to the DSMB; 2) a report for REPRIEVE (A5332) PIs and the NIH (NHLBI and DAIDS) team that will include MACE endpoint information pooled over both treatment groups; and 3) an open administrative report with administrative and safety information pooled over treatment groups. Specific contents of reports will be discussed with the DSMB prior to the first review. An outline of the focus of each review is provided in Table 9.5-2 below; further details and rationale are provided in the text below. These details are provided in broader detail in the Statistical Analysis Plan.

Table 9.5-2: Overview of DSMB Monitoring Focus

Focus		Timeframe
Feasibility and conduct	Site activation, enrollment, data and visit completeness, rates of loss to follow-up and cross-over	At all interim reviews occurring at least annually
Safety	Rates of adverse events by treatment group	At all interim reviews occurring at least annually
Event rate evaluation	Pooled rates of events observed to date; predicted confidence interval of the pooled event rate under a range of realistic scenarios*	At all interim reviews occurring at least annually
Formal efficacy and futility review	Treatment group comparison for the primary endpoint utilizing group sequential methods	Interim looks for efficacy are planned at approximately **50% and 75%** statistical information, **or as otherwise recommended****.
** To be conducted if the pooled rate falls below a specified target (see Statistical Analysis Plan).<br>** Statistical information for the MACE endpoint is based on the total number of expected MACE endpoints (see Statistical Analysis Plan for more details).*		

The first feasibility review will occur approximately one year after the accrual of the first participant with a focus on site activation and patient accrual and retention as well as rates of treatment crossover. Benchmarks for these aspects are provided in detail in the Statistical Analysis Plan that will be reviewed and agreed upon by the DSMB.

In addition to these aspects, pooled rates of events observed to date will be reviewed by the DSMB at all reviews to evaluate the adequacy of the sample size assumptions. Given the expected rate of accumulation of events it is anticipated that sufficient events will have been accrued to the study by the time of reviews occurring 2-2.5 years after enrollment of the first participant to allow reasonable determination of whether the underlying rate of event accumulation is inconsistent with observing the required total number of events to achieve 90% power to detect a HR of 0.70 at the study conclusion. This is illustrated by the anticipated rate of event accumulation dependent on the interim review timing shown in Table 9.5-3.

Table 9.5-3: Anticipated **D**ata and **E**vent **A**ccumulation **D**ependent of **I**nterim **R**eview Timing

Timing of review (y)	Number of participants enrolled*	Accumulated person years of follow-up*	Expected total accumulation of events under given event rate in control group (all cases assume the target HR of 0.7)		
			15/1000	12/1000	10/1000
1	508	127	2	1	1
1.5	1944	972	12	10	7
2	3609	707	34	28	21
2.5	5414	5685	72	58	43
3	6500	8125	103	82	62
3.5	6500	11375	143	115	86
*Assumes data freeze 4 months prior to DSMB review and enrollment benchmarks agreed with NHLBI.					

The event rate evaluation will be performed by a blinded REPRIEVE (A5332) statistician. In the event that the observed number of events falls short of the predetermined benchmarks providing in the statistical analysis plan, a predicted confidence interval analysis will be performed for the total expected number of MACE at trial conclusion based on accumulated data to date and a range of scenarios for accumulation of future data. These will include but not be limited to continued accumulation as observed and, under the target effect size of HR = 0.70, control MACE rate of 12/1000 PY and 15/1000 PY. Unless otherwise requested by the DSMB, these assessments along with recommendations for any study design changes will be presented only in the closed study report, and thus available only to members of the DSMB. Release of the information to the REPRIEVE (A5332) Executive Committee will be at the discretion of the DSMB.

If any of these assessments suggest that the anticipated total number of events appear substantially smaller than the rate assumed for sample size considerations or if accrual is below anticipated, consideration will be given to modifying the design of the study. Such considerations may include (but will not be limited to) extending the study duration, increasing the target sample size, and broadening the study entry criteria to include individuals with low/moderate traditional CVD risk who are willing to be randomized to statin therapy or placebo, for example, participants who have an ASCVD risk score of ≥7.5%.

Unless there are emerging feasibility or safety concerns, guidelines for stopping or modifying the trial will be guided by formal efficacy review to occur once sample size adequacy for the study has been established. **A total of four interim looks were planned originally: at 20% (if requested by DSMB), 40%, 60%, and 80%**

information. Per recommendation of NHLBI, endorsed by the DSMB at the December 2019 meeting, the timing of interim looks was changed to 50% and 75% information to allow for the best possible decision about extending study follow-up duration. Inference at each interim efficacy review will be guided by a Lan-DeMets implementation of the O'Brien-Fleming sequential stopping boundary with information measured on the cumulative number of **primary** MACE endpoints **at the time of the review**. This implementation permits early stopping only for very strong positive or negative effects and maintains most of the nominal power for **when the majority of information has accrued. For example, under the hypothesized effect size (HR=0.7), the cumulative probability that the interim result will cross a boundary at 50% information is 33% compared to a 78% probability at 75% information. T**he ultimate recommendation of all reviews—irrespective of whether boundary p-values have been achieved—will be at the discretion of the DSMB.

9.5.1 Monitoring of COVID-19-Related Outcomes

The incidence of COVID-19 diagnoses and hospitalizations (pooled over treatment groups) will be described as part of the routine (monthly) REPRIEVE adverse event monitoring reports and as targeted events at each DSMB review.

9.6 Analyses

9.6.1 General Analysis Considerations

The following sections provide a brief overview of the analysis considerations for REPRIEVE (A5332). Prior to the start of enrollment, a detailed Statistical Analysis Plan fully delineates all planned statistical analyses. **In the event of a DSMB recommendation to stop the trial for efficacy at interim review, full analysis and publication of the primary trial results will be prepared based on the frozen data for the DSMB meeting. Subsequent analyses (including a database prepared for future data-sharing requests) will be performed based on the final trial database including all final study termination visits. In the event of a DSMB recommendation to stop for futility, full analysis and publication of the primary trial results will be performed based on the final trial database including all final study termination visits.**

All major treatment comparisons between the randomized groups will be performed according to the principle of "intention-to-treat;" that is, participants will be analyzed (and endpoints attributed) according to the randomized treatment assigned regardless of subsequent changes to that treatment; as-treated analyses will also be performed. Descriptive summaries of the distribution of continuous baseline variables will be presented in terms of percentiles (eg, median, 25th and 75th percentiles), while discrete variables will be summarized in terms of frequencies and percentages. Statistical comparisons will be performed using two-sided significance tests with a 5% Type I error.

Unless otherwise noted, comparison of time to event outcomes will use methods for competing risks, notably Cox proportional hazards models for estimation of cause-specific hazard ratios and Gray's test for comparison of cumulative incidence curves.

Treatment group comparisons of continuous outcomes will use t-tests with transformation as needed. In the event of non-Normal distributions even after transformation, Wilcoxon rank sum tests will be preferred. With respect to discrete outcomes, Wilcoxon rank sum tests with be used for ordinal outcomes; Chi-squared test will be used otherwise; Fisher's exact test will be preferred in the event of small cell numbers. All testing will be stratified by sex and CD4 cell count per randomization: while the primary analyses will not be further stratified by enrollment in the Mechanistic Substudy of REPRIEVE (A5333s), sensitivity analyses will be performed including this stratification. Additional perspective regarding the interpretation of the data will be provided through extensive use of confidence intervals and graphical displays.

Given the strong plans for participant follow-up as part of the study, it is anticipated that missing data will be minimized. Unless examination of the data suggest otherwise, missing data will be assumed to be ignorable; participants lost to follow-up before experiencing a prior endpoint will be considered non-informatively censored. For all participants lost to follow-up, cardiovascular risk factors at the time of their final study visit will be described. In addition, a supportive analysis of time to death from any cause or MACE (outcome measure 9.2.2.3), including data from vital status and endpoint follow-up following premature study discontinuation will be conducted.

With the primary clinical and mechanistic hypotheses and the various secondary endpoints that have been outlined, it is recognized that there is a multiplicity of analyses to be performed, which leads to an increased probability that at least one of the comparisons could be "significant" by chance. Although the overall level of significance for all treatment comparisons will be 0.05, we will be conservative in the interpretation of our supporting analyses, taking into account the degree of significance, and looking for consistency across endpoints. Assessment will be made for any differences in major disease indices between participants in the Mechanistic Substudy of REPRIEVE (A5333s) and REPRIEVE (A5332). Such differences are unlikely, given that all participants in the Mechanistic Substudy will be eligible for REPRIEVE (A5332) and vice versa. Moreover, a subset of sites will be performing both and there will be overlap in the capacity to do both studies at selected sites. Nonetheless, we will analyze for any differences in the main and Mechanistic Substudy populations and account for any such differences in our interpretation of the Mechanistic Substudy and main study results.

9.6.2 Primary **MACE** and Supportive Analyses

The primary comparison of study arms for the primary composite endpoint will be time to event analyses and therefore based on the time from randomization to the first of any of the components of the primary composite endpoint. Deaths from non-CVD causes will be treated as competing risk events and participants completing follow-up without experiencing the event will be considered censored at the time of their last contact at which an assessment for primary endpoints was made.

A Stratified **cause-specific** Cox proportional hazards model will be the primary analytic methods used for assessing outcome differences between the two treatment groups with stratification by sex and CD4 cell count at screening as previously noted. The relative cause specific hazard of pitavastatin versus placebo for MACE will be estimated with a **repeated** 95% confidence interval and compared via a Wald test; modification of the statin effect over time (non-proportional hazards) will be evaluated with treatment by time interaction. In supportive analyses, the cumulative incidence of MACE will be estimated **using the Breslow estimators of the cause-specific hazard from the Cox model and plotted** over time by treatment group and compared via a stratified Gray test.

To complement the primary analyses, the same analytic approach will be used for evaluation of individual components of the primary MACE endpoint. In the absence of a competing risk event, treatment comparisons of all-cause mortality and a composite of MACE and all-cause mortality will use a stratified log-rank test.

Poisson regression with robust variance estimates will also be used to incorporate multiple and repeated events in evaluation of event incidence rates by treatment group and rate ratios. Sensitivity analyses will be performed that censor individuals for whom critically significant CAD was identified as a result of the Mechanistic Substudy of REPRIEVE (A5333s) CCTA evaluation; censoring will occur at the date of the apparent CCTA study findings.

9.6.3 Secondary **MACE and Lipid** Analyses

Analyses of targeted serious clinical diagnoses (see section 9.2.2.4) will use the same methods as described for the primary MACE endpoint for the composite and individual outcomes. Analyses of a further composite outcome including the primary MACE outcomes (the START endpoint) will also be performed.

Summary statistics (means and quantile distributions) will be provided to describe the distributions of LDL and non-HDL cholesterol from study entry and 12 month intervals over time. At each annual post-entry time point, the mean difference in levels between treatment groups will be estimated with 95% confidence interval. **Mixed effects models adjusted** for sex and screening CD4

cell count will be used **for repeated measurements over time** to **estimate annual** treatment group **differences**; the same analyses will be applied for other lipid fractions.

Prognostic Factors of MACE: Important secondary aims of REPRIEVE (A5332) are to evaluate whether baseline traditional risk factors and time updated HIV-specific risk factors are predictive of MACE and pitavastatin effects on MACE in the HIV population.

Targeted risk factors of interest at (or prior to) study entry and time-updated (as indicated) are as follows:

<u>At study entry/screening</u>:
- Age, sex, race
- **ASCVD risk score**
- HIV-1 RNA level
- CD4 cells count
- Nadir CD4 cell count
- Duration of ART exposure and any exposure to thymidine analogs, protease inhibitors, or abacavir
- Weight, BMI, waist circumference
- Fasting lipid (TC, HDL-C and LDL-C, HDL:LDL ratio, TG) and glucose
- Smoking status
- Systolic and diastolic BP and use of antihypertensive agents
- Presence of metabolic syndrome defined according to current guidelines at the time of the analysis.
- Self-reported level of physical activity
- Family history of heart disease

<u>Time-updated (annually unless otherwise noted)</u>:
- HIV-1 RNA level
- CD4 cells count
- Fasting lipid (TC, HDL-C and LDL-C, HDL:LDL ratio, TG) and glucose and their changes expressed as absolute change and as percent of baseline

Analyses will use stratified Cox proportional hazards models to estimate the cause-specific hazard of MACE with respect to the risk factors of interest, including the selected biomarkers representing surrogates from plaque progression as described above. In the full cohort we will include the baseline risk factors and evaluate modification of the statin effect by key subgroups (ie, race/ethnicity sex, and CD4 cell count at screening as well as HIV-related and CV risk factors including age, hypertension, LDL and non-HDL cholesterol at entry, BMI, metabolic syndrome (as defined by current NCEP guidelines at the time of the analysis) and smoking) by interaction terms in these models.

This approach will investigate associations with LDL and non-HDL levels and changes from baseline as a time-updated covariate. The same approach to analysis will be used to assess the effects of other longitudinal outcomes. With respect to HIV-1 RNA levels over time, it is of particular interest to examine whether there is evidence of the modification of the effect of statins according to whether participants maintain full suppression of HIV-1 RNA levels. This will be examined in a 12-month landmarked Cox proportional hazards models assessing an interaction between continued HIV-1 RNA suppression (<**400** copies/mL) over the first 12 months of the study as well as time-updated analysis. The analysis approach for assessment of effect of selected biomarkers for MACE will be determined based on the sampling approach to biomarker testing. Depending on power consideration, sampling may involve testing of the entire REPRIEVE (A5332) study population or a restricted sampling approach such as a case-cohort sampling. In this case, the same analytic approach as described above would be utilized with appropriate weighting for the sampling fractions; an alternative would be case-control sampling with analyses performed using logistic regression.

The frequency with which serious adverse events (excluding study defined clinical endpoints) occur will be tabulated and descriptively summarized. Additional targeted and pre-specified adverse events that will be summarized will include incident diabetes, elevated liver function tests, and myositis. Statistical comparisons of the randomized arms with respect to adverse events will use chi-square or other appropriate two-sample methods depending on the nature of the event, interpreting such comparisons in the context of differences between the two randomized arms in the primary and major secondary clinical endpoints.

9.6.4 COVID-19 Analyses

All analyses will be restricted to participants who remained in REPRIEVE study follow-up as of January 1, 2020. Person years of follow-up will be measured from January 1, 2020.

The incidence of COVID-19 diagnoses and COVID-19 hospitalizations will be estimated, including the statin effect on the incidence of COVID-19 and serious COVID-19 disease via estimation of the incidence rate ratio by randomized treatment group. Log binomial regression will be utilized to assess host factors associated with COVID-19 infection as defined by a) SARS-CoV-2 antibody positivity and b) severe disease requiring hospitalization.

Analyses will be stratified by global burden of disease region and randomized treatment and control for comorbid conditions (including hypertension, diabetes, kidney disease, and obesity). Further extension of these analyses will evaluate the influence of targeted concomitant medications (such as lopinavir/ritonavir and of ACE inhibitors and ARBs), and COVID-19 vaccination on rates of SARS-CoV-2 infection.

In all analyses, death (not due to COVID-19) will be considered a censoring event. Discontinuation of randomized treatment will be ignored in primary analyses; a supportive analysis will censor at randomized treatment discontinuation and use inverse probability of censoring weights (IPCW) to estimate the statin effect when statins are being taken per protocol. Likewise, COVID-19 vaccination will be ignored in the primary analysis; a supportive analysis will censor at COVID-19 vaccination and use IPCW to estimate the statin effect in the absence of COVID-19 vaccination.

More detailed analysis plans are provided in the REPRIEVE Statistical Analysis Plan.

10.0 PHARMACOLOGY PLAN

Not applicable.

11.0 DATA COLLECTION AND MONITORING AND ADVERSE EVENT REPORTING

11.1 Records to Be Kept

Electronic case report forms (CRF) are available on the DMC website. Participants must not be identified by name on any CRFs. Participants will be identified by the patient identification number (PID), screening number (SN), and study identification number (SID) provided by the DMC upon randomization.

11.2 Role of Data Management

Instructions concerning the recording of study data on **e**CRFs will be provided by the DMC. Each CRS is responsible for recording the data in a timely fashion.

It is the responsibility of the DMC to ensure the quality of computerized data for each study. This role extends from protocol development to generation of the final study databases.

11.3 Clinical Site Monitoring and Record Availability

Monitoring visits may be conducted on-site or remotely. Remote visits may include remote source document verification using methods specified for this purpose by NIAID. Remote monitoring visits may be performed in place of, or in addition to, onsite visits to ensure the safety of study participants and data integrity [FDA, 2021]. The site will make available study documents for site monitors to review utilizing a secure platform that is HIPAA and 21 CFR Part 11 compliant. Potential platform options include: Veeva SiteVault, site-controlled SharePoint or cloud-based portal, direct access to Electronic Medical Record (EMR), and Medidata Rave Imaging Solutions. Other secure platforms that are 21

CFR Part 11 compliant may be utilized, as allowed by the DAIDS Office of Clinical Site Oversight (OCSO).

11.4 Expedited Adverse Event Reporting to DAIDS

11.4.1 Adverse Event Reporting to DAIDS

Requirements, definitions, and methods for expedited reporting of AEs are outlined in Version 2.0 of the DAIDS EAE Manual, which is available on the RSC website at https://rsc.niaid.nih.gov/clinical-research-sites/manual-expedited-reporting-adverse-events-daids.

The DAIDS Adverse Experience Reporting System (DAERS), an Internet-based reporting system, must be used for expedited AE reporting to DAIDS. In the event of system outages or technical difficulties, expedited AEs may be submitted via the DAIDS EAE Form. For questions about DAERS, please contact DAIDS-ES (now part of the NIAID Clinical Research Management System) at CRMSsupport@niaid.nih.gov. Site queries may also be sent from within the DAERS application itself.

Sites where DAERS has not been implemented will submit expedited AEs by documenting the information on the current DAIDS EAE Form. This form is available on the RSC website: https://rsc.niaid.nih.gov/clinical-research-sites/paper-eae-reporting. For questions about EAE reporting, please contact the RSC (DAIDSRSCSafetyOffice@tech-res.com).

11.4.2 Reporting Requirements for this Study

The SAE Reporting Category, as defined in Version 2.0 of the DAIDS EAE Manual, will be used for this study with certain exceptions as noted below. Unless the site investigator considers the following events to be study drug-related, DO NOT REPORT them as EAEs:

- AIDS-defining events (see MOPS for a listing)
- REPRIEVE MACE (see section 9.2.1 for complete listing) and related CVD events: CVD death, MI, stroke, TIA, unstable angina, peripheral ischemia, coronary or peripheral reperfusion procedures, heart failure**, and all deaths** (because they are components of the primary endpoint or additional adjudicated CVD endpoint). See MOPS for details about reporting CVD Endpoints).
- **COVID-19 hospitalizations.**

NOTE: COVID-19 is a novel disease that has emerged since protocol version 5.0 was distributed. Given our evolving understanding of this viral infection, and the likelihood of subclinical cardiac injury during COVID-19 hospitalization, the requirement for reporting COVID-19 infection has been outlined in greater detail in the MOPS.

The study agents for which expedited reporting are required are pitavastatin and placebo for pitavastatin.

11.4.3 Grading Severity of Events

The Division of AIDS Table for Grading the Severity of Adult and Pediatric Adverse Events (DAIDS AE Grading Table), corrected Version 2.1, July 2017, is available on the DAIDS RSC website at https://rsc.niaid.nih.gov/clinical-research-sites/daids-adverse-event-grading-tables.

11.4.4 Expedited AE Reporting Period

The expedited AE reporting period for this study is the entire study duration for an individual participant (from study enrollment until study completion or discontinuation of the participant from study participation for any reason).

After the protocol-defined AE reporting period, unless otherwise noted, only SUSARs (suspected unexpected serious adverse reactions) as defined in Version 2.0 of the EAE Manual, will be reported to DAIDS if the study staff become aware of the events on a passive basis (from publicly available information).

12.0 PARTICIPANTS

12.1 Institutional Review Board (IRB) Review and Informed Consent

This protocol and the informed consent document (Appendix I) and any subsequent modifications will be reviewed and approved by the IRB/EC responsible for oversight of the study. A signed consent form will be obtained from the participant (or legal representative). The consent form will describe the purpose of the study, the procedures to be followed, and the risks and benefits of participation. A copy of the consent form will be given to the participant or legal representative, and this fact will be documented in the participant's record. Risks, including potential risks of pitavastatin, and protection against risk are described in the accompanying sample informed consent form.

12.2 Participant Confidentiality

All laboratory specimens, evaluation forms, reports, and other records that leave the site will be identified by coded number only to maintain participant confidentiality. All records will be kept locked. All computer entry and networking programs will be done with coded numbers only. Clinical information will not be released without written permission of the participant, except as necessary for monitoring by the ACTG, IRB/EC, FDA, NHLBI, NIAID, OHRP, and other local, US, and international regulatory entities as part of their duties, or the industry supporters or designee.

12.3 Study Discontinuation

The study may be discontinued at any time by the ACTG, IRB/EC, FDA, NIAID, NHLBI, OHRP, or the industry supporter, or other government agencies as part of their duties to ensure that research participants are protected.

12.4 Women and Minorities

REPRIEVE (A5332) will aim to recruit women and minority participants commensurate with the population demographic for HIV in the US. The prevalence of HIV among women in the US is approximately 22%, while over half of **PWH** in the US are minorities (CDC 2014).

13.0 PUBLICATION OF RESEARCH FINDINGS

Publication of the results of this trial will be governed by NHLBI and NIAID policies.

14.0 BIOHAZARD CONTAINMENT

All dangerous goods and materials, including diagnostic specimens and infectious substances, must be transported using packaging mandated by CFR 42 Part 72. Please refer to instructions detailed in the International Air Transport Association (IATA) Dangerous Goods Regulations.

15.0 STUDY GOVERNANCE

- There is an Executive Committee, a DSMB, and an External Advisory Board.
- There are various other operational committees to ensure that the trial progresses smoothly.
- There is a Clinical Events Committee (CEC) charter.

16.0 REFERENCES

Aberg JA, **Gallant JE, Ghanem KG,** et al. Primary care guidelines for the management of persons infected with HIV: 2013 update by the HIV Medicine Association of the Infectious Diseases Society of America. Clin Infect Dis 2014;58(1):1-10.

Aberg JA, **Kaplan JE, Libman H,** et al. HIV Medicine Association of the Infectious Diseases Society of America. Primary care guidelines for the management of persons infected with human immunodeficiency virus: 2009 update by the HIV Medicine Association of the Infectious Diseases Society of America. Clin Infect Dis 2009;49(5):651-81.

Aberg JA, **Sponseller CA, Ward DJ,** et al. Pitavastatin versus pravastatin in adults with HIV-1 infection and dyslipidaemia (INTREPID): 12 week and 52 week results of a phase 4, multicentre, randomised, double-blind, superiority trial. Lancet HIV 2017;4(7):e284-e294.

Ahmed MH, **Al-Atta A, Mahir A Hamad MA**. The safety and effectiveness of statins as treatment for HIV-dyslipidemia: the evidence so far and the future challenges. Expert Opin Pharmacother 2012;13(13):1901-9.

Amarenco P, **Callahan 3rd A, Campese VM,** et al. Effect of high-dose atorvastatin on renal function in subjects with stroke or transient ischemic attack in the SPARCL Trial. Stroke 2014;45(10):2974-82.

Amet T, **Nonaka M, Dewan MZ,** et al. Statin-induced inhibition of HIV-1 release from latently infected U1 cells reveals a critical role for protein prenylation in HIV-1 replication. Microbes Infect 2008;10(5):471-80.

Antiretroviral Therapy Cohort Collaboration. Causes of death in HIV-1-infected patients treated with antiretroviral therapy, 1996-2006: collaborative analysis of 13 HIV cohort studies. Clin Infect Dis 2010;50(10):1387-96.

Aviram M, **Dankner G, Cogan U, Hochgraf E, Brook JG**. Lovastatin inhibits low-density lipoprotein oxidation and alters its fluidity and uptake by macrophages: in vitro and in vivo studies. Metabolism 1992;41(3):229-35.

Borges AH, Dubrow S, Silverberg MJ, et al. Factors contributing to risk for cancer among HIV-infected individuals, and evidence that earlier combination antiretroviral therapy will alter this risk. Curr Opin HIV AIDS 2014;9(1):34-40.

Brenchley JM, **Price DA, Schacker TW**, et al. Microbial translocation is a cause of systemic immune activation in chronic HIV infection. Nat Med 2006;12(12):1365-71.

Brett SJ, Myles P, Lim WS, et al. Pre-admission statin use and in-hospital severity of 2009 pandemic influenza A(H1N1) disease. PLoS One 2011;6(4):e18120.

Bu DX, **Tarrio M, Grabie N,** et al. Statin-induced Kruppel-like factor 2 expression in human and mouse T cells reduces inflammatory and pathogenic responses. J Clin Invest 2010;120(6):1961-70.

Burdo TH, **Lo J, Abbara S**, et al. Soluble CD163, a novel marker of activated macrophages, is elevated and associated with noncalcified coronary plaque in HIV-infected patients. J Infect Dis 2011;204(8):1227-36.

Burgstahler C, **Reimann A, Beck T**, et al. Influence of a lipid-lowering therapy on calcified and noncalcified coronary plaques monitored by multislice detector computed tomography: results of the New Age II Pilot Study. Invest Radiol 2007;42(3):189-95.

Calfee CS, Delucchi KL, Sinha P, et al.; Irish Critical Care Trials Group. Acute respiratory distress syndrome subphenotypes and differential response to Simvastatin: secondary analysis of a randomized controlled trial. Lancet Respir Med 2018;6(9):691-8.

Calza L, **Trapani F, Bartoletti M,** et al. Statin therapy decreases serum levels of high-sensitivity C-reactive protein and tumor necrosis factor-α in HIV-infected patients treated with ritonavir-boosted protease inhibitors. HIV Clin Trials 2012;13(3):153-61.

Carlberg M, **Dricu A, Blegen H,** et al. Mevalonic acid is limiting for N-linked glycosylation and translocation of the insulin-like growth factor-1 receptor to the cell surface. Evidence for a new link between 3-hydroxy-3-methylglutaryl-coenzyme a reductase and cell growth. J Biol Chem 1996;271(29):17453-62.

Centers for Disease Control and Prevention. Who’s at Risk for HIV. Accessed September 8, 2014 from http://www.cdc.gov/hiv/risk/.

Chauvin B, **Drouot S, Barrail-Tran A, Taburet AM**. Drug-drug interactions between HMG-CoA reductase inhibitors (statins) and antiviral protease inhibitors. Clin Pharmacokinet 2013;52(10):815-31.

Cholesterol Treatment Trialists’ (CTT) Collaboration; Baigent C, **Blackwell L, Emberson J**, et al. Efficacy and safety of more intensive lowering of LDL cholesterol: a meta-analysis of data from 170,000 participants in 26 randomised trials. Lancet 2010;376(9753):1670-81.

Colhoun HM, **Betteridge DJ, Durrington PN,** et al. Effects of atorvastatin on kidney outcomes and cardiovascular disease in patients with diabetes: an analysis from the Collaborative Atorvastatin Diabetes Study (CARDS). Am J Kidney Dis 2009;54(5):810-9.

Crum-Cianflone N, **Hullsiek KH, Marconi V,** et al. Trends in the incidence of cancers among HIV-infected persons and the impact of antiretroviral therapy: a 20-year cohort study. AIDS 2009;23(1):41-50.

Currier JS, **Taylor A, Boyd F,** et al. Coronary heart disease in HIV-infected individuals. J Acquir Immune Defic Syndr 2003;33(4):506-12.

Data Collection on Adverse Events of Anti-HIV Drugs (D:A:D) Study Group; Smith C, **Sabin CA, Lundgren JD**, et al. Factors associated with specific causes of death amongst HIV-positive individuals in the D:A:D Study. AIDS 2010;24(10):1537-48.

deFilippi C, Toribio M, Wong LP, et al. Differential Plasma protein regulation and statin effects in HIV-infected and non-HIV-infected patients utilizing a proteomics approach protein regulation and statin effects in HIV. J Infect Dis 2020;226(6):929-39.

De Wit S, **Delforge M, Necsoi CV, Clumeck N**. Downregulation of CD38 activation markers by atorvastatin in HIV patients with undetectable viral load. AIDS 2011;25(10):1332-3.

Deeken JF, **Tjen-A-Looi A, Rudek MA,** et al. The rising challenge of non-AIDS-defining cancers in HIV-infected patients. Clin Infect Dis 2012;55(9):1228-35.

Deeks SG. HIV infection, inflammation, immunosenescence, and aging. Ann Rev Med 2011;62:141-55.

del Real G, **Jiminez-Baranda S, Mira E,** et al. Statins inhibit HIV-1 infection by down-regulating Rho activity. J Exp Med 2004;200(4):541-7.

Dolan SE, **Hadigan C, Killilea KM,** et al. Increased cardiovascular disease risk indices in HIV-infected women. J Acquir Immune Defic Syndr 2005;39(1):44-54.

Douglas K, **O'Malley PG, Jackson JL**. Meta-analysis: the effect of statins on albuminuria. Ann Intern Med 2006;145(2):117-24.

Downs JR, **Clearfield M, Weis S,** et al. Primary prevention of acute coronary events with lovastatin in men and women with average cholesterol levels: results of AFCAPS/TexCAPS. Air Force/Texas Coronary Atherosclerosis Prevention Study. JAMA 1998;279(20):1615-22.

Drechsler H, **Ayers C, Cutrell J,** et al. Current use of statins reduces risk of HIV rebound on suppressive HAART. PLoS One 2017;12(3):e0172175.

Durand M, **Sheehy O, Baril JG,** et al. Association between HIV infection, antiretroviral therapy, and risk of acute myocardial infarction: a cohort and nested case-control study using Quebec's public health insurance database. J Acquir Immune Defic Syndr 2011;57(3):245-53.

Eckard AR, **Jiang Y, Debanne SM,** et al. Effects of 24 weeks of statin therapy on systemic and vascular inflammation in HIV-infected subjects receiving antiretroviral therapy. J Infect Dis 2014;209(8):1156-64.

Ekstedt M, **Franzen LE, Mathiesen UL,** et al. Statins in non-alcoholic fatty liver disease and chronically elevated liver enzymes: a histopathological follow-up study. J Hepatol 2007;47(1):135-41.

Eriksson M, **Budinski D, Hounslow N**. Comparative efficacy of pitavastatin and simvastatin in high-risk patients: a randomized controlled trial. Adv Ther 2011**a**;28(9):811-23.

Eriksson M, **Budinski D, Hounslow N**. Long-term efficacy of pitavastatin versus simvastatin. Adv Ther 2011**b**;28(9):799-810.

FDA. FDA Guidance on Conduct of Clinical Trials of Medical Products During the COVID-19 Public Health Emergency: Guidance for Industry, Investigators, and Institutional Review Boards. March 2020; Updated on January 27, 2021. Accessed at: https://www.fda.gov/media/136238/download.

Foster T, **Budoff MJ, Saab S,** et al. Atorvastatin and antioxidants for the treatment of nonalcoholic fatty liver disease: the St Francis Heart Study randomized clinical trial. Am J Gastroenterol 2011;106(1):71-7.

Freiberg MS, **Chang CCH, Kuller LH,** et al. HIV infection and the risk of acute myocardial infarction. JAMA Intern Med 2013;173(8):614-22.

French AL, **Gawel SH, Hershow R,** et al. Trends in mortality and causes of death among women with HIV in the United States: a 10-year study. J Acquir Immune Defic Syndr 2009;51(4):399-406.

Friis-Moller N, **Sabin CA, Weber R,** et al. Combination antiretroviral therapy and the risk of myocardial infarction. N Engl J Med 2003;349(21):1993-2003.

Funderburg NT, **Jiang Y, Debanne SM,** et al. Rosuvastatin reduces vascular inflammation and T cell and monocyte activation in HIV-infected subjects on antiretroviral therapy. J Acquir Immune Defic Syndr 2015;68(4):396-404.

Funderburg NT, **Jiang Y, Debanne SM,** et al. Rosuvastatin treatment reduces markers of monocyte activation in HIV-infected subjects on antiretroviral therapy. Clin Infect Dis 2014;58(4):588-95.

Fujino M, **Miura S, Matsuo Y,** et al. Pitavastatin-induced downregulation of CCR2 and CCR5 in monocytes is associated with the arrest of cell-cycle in S phase. Atherosclerosis 2006;187(2):301-8.

Galli L, **Spagnuolo V, Poli A,** et al. Use of statins and risk of AIDS-defining and non-AIDS defining malignancies among HIV-1 infected patients on antiretroviral therapy. AIDS 2014;28(16):2407-15.

Ganesan A, **Crum-Cianflone N, Higgins J,** et al. High dose atorvastatin decreases cellular markers of immune activation without affecting HIV-1 RNA levels: results of a double-blind randomized placebo controlled clinical trial. J Infect Dis 2011;203(6):756-64.

Gerber JG, **Rosenkranz SL, Fichtenbaum CJ,** et al. Effect of efavirenz on the pharmacokinetics of simvastatin, atorvastatin, and pravastatin: results of AIDS Clinical Trials Group 5108 Study. J Acquir Immune Defic Syndr 2005;39(3):307-12.

Giguere JF, Tremblay MJ. Statin compounds reduce human immunodeficiency virus type 1 replication by preventing the interaction between virion-associated host intercellular adhesion molecule 1 and its natural cell surface ligand LFA-1. J Virol 2004;78(21):12062-5.

Gilbert C, **Bergeron M, Methot S,** et al. Statins could be used to control replication of some viruses, including HIV-1. Viral Immunol 2005;18(3):474-89.

Giroux LM, **Davignon J, Naruszewicz M.** Simvastatin inhibits the oxidation of low-density lipoproteins by activated human monocyte-derived macrophages. Biochim Biophys Acta 1993;1165(3):335-8.

Gomez-Domínguez, **Gisbert JP, Moreno-Monteagudo JA,** et al. A pilot study of atorvastatin treatment in dyslipid, non-alcoholic fatty liver patients. Aliment Pharmacol Ther 2006;23(11):1643-7.

Gotto AM Jr, Moon J. Pitavastatin for the treatment of primary hyperlipidemia and mixed dyslipidemia. Expert Rev Cardiovasc Ther 2010;8(8):1079-90.

Greene M, **Justice AC, Lampiris HW, Valcour V.** Management of human immunodeficiency virus infection in advanced age. JAMA 2013;309(13):1397-405.

Greenwood J, Mason JC. Statins and the vascular endothelial inflammatory response. Trends Immunol 2007;28(2):88-98.

Grinspoon SK, **Grunfeld C, Kotler DP,** et al. State of the science conference: Initiative to decrease cardiovascular risk and increase quality of care for patients living with HIV/AIDS: executive summary. Circulation 2008;118(2):198-210.

Guan J, Sun X, Liang Y, et al. Atorvastatin attenuates Coxsackie virus B3m-induced viral myocarditis in mice. J Cardiovasc Pharmacol 2010,56:540-7.

Gumprecht J, **Gosho M, Budinski D, Hounslow N.** Comparative long-term efficacy and tolerability of pitavastatin 4 mg and atorvastatin 20-40 mg in patients with type 2 diabetes mellitus and combined (mixed) dyslipidaemia. Diabetes Obes Metab 2011;13(11):1047-55.

Guo H, **Shi Y, Liu L,** et al. Rosuvastatin inhibits MMP-2 expression and limits the progression of atherosclerosis in LDLR-deficient mice. Arch Med Res 2009;40(5):345-51.

Han J, **Zhou X, Yokoyama T,** et al. Pitavastatin downregulates expression of the macrophage type B scavenger receptor, CD36. Circulation 2004;109(6):790-6.

Han KH, **Ryu J, Hong KH,** et al. HMG-CoA reductase inhibition reduces monocyte CC chemokine receptor 2 expression and monocyte chemoattractant protein-1-mediated monocyte recruitment in vivo. Circulation 2005;111(11):1439-47.

Hicks, KA, **Tcheng JE, Bozkurt B,** et al.; **Japan-ACS Investigators.** 2014 ACC/AHA key data elements and definitions for cardiovascular endpoint events in clinical trials: a report of the American College of Cardiology/ American Heart Association Task Force on Clinical Data Standards (Writing Committee to Develop Cardiovascular Endpoints Data Standards). J Am Coll Cardiol 2015;66(4):403-69.

Hiro T, **Kimura T, Morimoto T,** et al. Effect of intensive statin therapy on regression of coronary atherosclerosis in patients with acute coronary syndrome: a multicenter randomized trial evaluated by volumetric intravascular ultrasound using pitavastatin versus atorvastatin (JAPAN-ACS [Japan assessment of pitavastatin and atorvastatin in acute coronary syndrome] study). J Am Coll Cardiol 2009;54(4):293-302.

Hou ZH, **Lu B, Gao Y**, et al. Prognostic value of coronary CT angiography and calcium score for major adverse cardiac events in outpatients. JACC Cardiovasc Imaging 2012;5(10):990-9.

Hsue PY, **Deeks SG, Hunt PW**. Immunologic basis of cardiovascular disease in HIV-infected adults. J Infect Dis 2012;205 Suppl 3:S375-S382.

Hulten E, **Mitchell J, Scally J, Gibbs B, Villines TC**. HIV positivity, protease inhibitor exposure and subclinical atherosclerosis: a systematic review and meta-analysis of observational studies. Heart 2009;95(22):1826-35.

Hyogo H, **Yamagishi S, Maeda S, Kimura Y, Ishitobi T, Chayama K.** Atorvastatin improves disease activity of nonalcoholic steatohepatitis partly through its tumor necrosis factor-α-lowering property. Dig Liver Dis 2012;44(6):492-6.

Inoue K, **Motoyama S, Sarai M,** et al. Serial coronary CT angiography-verified changes in plaque characteristics as an end point: evaluation of effect of statin intervention. JACC Cardiovasc Imaging 2010;3(7):691-8.

Jacobson TA, **Ito MK, Maki KC,** et al. National Lipid Association recommendations for patient-centered management of dyslipidemia: part 1 - executive summary. J Clin Lipidol 2014;8(5):473-88.

Kitagawa T, **Yamamoto H, Horiguchi J,** et al. Characterization of noncalcified coronary plaques and identification of culprit lesions in patients with acute coronary syndrome by 64-slice computed tomography. JACC Cardiovasc Imaging 2009;2(2):153-60.

Klein D, **Hurley LB, Quesenberry CP Jr, Sidney S.** Do protease inhibitors increase the risk for coronary heart disease in patients with HIV-1 infection? J Acquir Immune Defic Syndr 2002;30(5):471-7.

Kodama K, **Komatsu S, Ueda Y,** et al. Stabilization and regression of coronary plaques treated with pitavastatin proven by angioscopy and intravascular ultrasound--the TOGETHAR trial. Circ J 2010;74(9):1922-8.

Koenen RR, Weber C. Therapeutic targeting of chemokine interactions in atherosclerosis. Nat Rev Drug Discov 2010;9(2):141-53.

Kuller LH, Tracy R, Belloso W, et al.; **INSIGHT SMART Study Group.** Inflammatory and coagulation biomarkers and mortality in patients with HIV infection. PLoS Med 2008;5(10):e203.

Kwak B, **Mulhaupt F, Myit S, Mach F.** Statins as a newly recognized type of immunomodulator. Nat Med 2000;6(12):1399-402.

Lang S, **Mary-Krause M, Cotte L,** et al. Increased risk of myocardial infarction in HIV-infected patients in France, relative to the general population. AIDS 2010;24(8):1228-30.

Lederman MM, Funderburg NT, Sekaly RP, Klatt NR, Hunt PW. Residual immune dysregulation syndrome in treated HIV infection. Adv Immunol 2013,119:51-83.

Lewden C, **May T, Rosenthal E,** et al.; **ANRS EN19 Mortalité Study Group and Mortavic.** Changes in causes of death among adults infected by HIV between 2000 and 2005: The "Mortalité 2000 and 2005" surveys (ANRS EN19 and Mortavic). J Acquir Immune Defic Syndr 2008;48(5):590-8.

Libby P, **Ridker PM, Hansson GK.** Progress and challenges in translating the biology of atherosclerosis. Nature 2011;473(7347):317-25.

Libby P, **Ridker PM, Maseri A.** Inflammation and atherosclerosis. Circulation 2002;105(9):1135-43.

Lichtenstein K, **Hart RLD, Wood KC,** et al.; HIV Outpatient Study Investigators. Statin use is associated with incident diabetes mellitus among patients in the HIV Outpatient Study. JAIDS 2015;69(3):306-11.

Longenecker CT, **Hileman CO, Funderburg NT, McComsey GA.** Rosuvastatin preserves renal function and lowers cystatin C in HIV-infected subjects on antiretroviral therapy: The SATURN-HIV Trial. Clin Infect Dis 2014;59(8):1148-56.

Madjid M, Safavi-Naeini P, Solomon SD, Vardeny O. Potential effects of coronaviruses on the cardiovascular system: a review. JAMA Cardiol 2020;5(7):831-40.

Malvestutto CD, **Ma Q, Morse GD, Underberg JA, Aberg JA.** Lack of pharmacokinetic interactions between pitavastatin and efavirenz or darunavir/ritonavir. J Acquir Immune Defic Syndr 2014;67(4):390-6.

Marin B, **Thiebaut R, Bucher HC,** et al. Non-AIDS-defining deaths and immunodeficiency in the era of combination antiretroviral therapy. AIDS 2009;23(13):1743-53.

Maruyama T, **Takada M, Nishibori Y,** et al. Comparison of preventive effect on cardiovascular events with different statins. The CIRCLE study. Circ J 2011;75(8):1951-9.

Mira E, **Leon B, Barber DF,** et al. Statins induce regulatory T cell recruitment via a CCL1 dependent pathway. J Immunol 2008;181(5):3524-34.

Miyaki T, **Nojiri S, Shinkai N**, et al. Pitavastatin inhibits hepatic steatosis and fibrosis in non-alcoholic steatohepatitis model rats. Hepatol Res 2011;41(4):375-85.

Moncunill G, **Negredo E, Bosch L,** et al. Evaluation of the anti-HIV activity of statins. AIDS 2005;19(15):1697-700.

Montecucco F, **Burger F, Pelli G,** et al. Statins inhibit C-reactive protein-induced chemokine secretion, ICAM-1 upregulation and chemotaxis in adherent human monocytes. Rheumatology (Oxford) 2009;48(3):233-42.

Moore RD, **Bartlett JG, Gallant JE.** Association between use of HMG CoA reductase inhibitors and mortality in HIV-infected patients. PLoS ONE 2011;6(7):e21843.

Mulhaupt F, **Matter CM, Kwak BR,** et al. Statins (HMG-CoA reductase inhibitors) reduce CD40 expression in human vascular cells. Cardiovasc Res 2003;59(3):755-66.

Nabatov AA, **Pollakis G, Linneman T,** et al. Statins disrupt CCR5 and RANTES expression levels in CD4(+) T lymphocytes in vitro and preferentially decrease infection of R5 versus X4 HIV-1. PLoS ONE 2007;2(5):e470.

Nakamura T, **Obata JE, Kitta Y,** et al. Rapid stabilization of vulnerable carotid plaque within 1 month of pitavastatin treatment in patients with acute coronary syndrome. J Cardiovasc Pharmacol 2008;51(4):365-71.

Nayor M, Vasan RS. Recent update to the US Cholesterol Treatment Guidelines: a comparison with international guidelines. Circulation 2016;133(18):1795-806.

Negredo E, **Clotet B, Puig J,** et al. The effect of atorvastatin treatment on HIV-1-infected patients interrupting antiretroviral therapy. AIDS 2006;20(4):619-21.

Neuhaus J, **Angus B, Kowalska JD,** et al.; INSIGHT SMART and ESPRIT Study Groups. Risk of all-cause mortality associated with nonfatal AIDS and serious non-AIDS events among adults infected with HIV. AIDS 2010;24(5):697-706.

Nikolic D, **Banach M, Nikfar S,** et al. A meta-analysis of the role of statins on renal outcomes in patients with chronic kidney disease. Is the duration of therapy important? Int J Cardiol 2013;168(6):5437-47.

Obel N, **Thomsen HF, Kronborg G,** et al. Ischemic heart disease in HIV-infected and HIV-uninfected individuals: a population-based cohort study. Clin Infect Dis 2007;44(12):1625-31.

Overton ET, **Kitch D, Benson CA,** et al. Effect of statin therapy in reducing the risk of serious non-AIDS-defining events and nonaccidental death. Clin Infect Dis 2013;56(10):1471-9.

Packard RR, **Lichtman AH, Libby P.** Innate and adaptive immunity in atherosclerosis. Semin Immunopathol 2009;31(1):5-22.

Palella FJ, Jr., **Baker RK, Moorman AC,** et al.; **HIV Outpatient Study Investigators.** Mortality in the highly active antiretroviral therapy era: changing causes of death and disease in the HIV outpatient study. J Acquir Immune Defic Syndr 2006;43(1):27-34.

Palmer SC, **Navaneethan SD, Craig JC,** et al. HMG CoA reductase inhibitors (statins) for people with chronic kidney disease not requiring dialysis. Cochrane Database Syst Rev 2014;5:CD007784.

Paton NI, **Goodall RL, Dunn DT,** et al. Effects of hydroxychloroquine on immune activation and disease progression among HIV-infected patients not receiving antiretroviral therapy: a randomized controlled trial. JAMA 2012;308(4):353-61.

Rasmussen LD, **Kronborg G, Larsen CS,** et al. Statin therapy and mortality in HIV-infected individuals; a Danish nationwide population-based cohort study. PLoS ONE 2013;8(3):e52828.

Ridker PM, **Danielson E, Fonseca FA,** et al.; **JUPITER Study Group.** Rosuvastatin to prevent vascular events in men and women with elevated C-reactive protein. N Engl J Med 2008;359(21):2195-207.

Romano M, **Diomede L, Sironi M,** et al. Inhibition of monocyte chemotactic protein-1 synthesis by statins. Lab Invest 2000;80(7):1095-100.

Rominger A, **Saam T, Wolpers S,** et al. 18F-FDG PET/CT identifies patients at risk for future vascular events in an otherwise asymptomatic cohort with neoplastic disease. J Nucl Med 2009;50(10):1611-20.

Ross AC, **Armentrout R, O'Riordan MA, Storer N,** et al. Endothelial activation markers are linked to HIV status and are independent of antiretroviral therapy and lipoatrophy. J Acquir Immune Defic Syndr 2008;49(5):499-506.

Ross R. Atherosclerosis--an inflammatory disease. N Engl J Med 1999;340(2):115-26.

Sackoff JE, **Hanna DB, Pfeiffer MR, Torian LV.** Causes of death among persons with AIDS in the era of highly active antiretroviral therapy: New York City. Ann Intern Med 2006;145(6):397-406.

Sacks FM, **Pfeffer MA, Moye LA,** et al. The effect of pravastatin on coronary events after myocardial infarction in patients with average cholesterol levels. Cholesterol and Recurrent Events Trial investigators. N Engl J Med 1996;335(14):1001-9.

Saghazadeh A, Rezaei N. Immune-epidemiological parameters of the novel coronavirus - a perspective. Expert Rev Clin Immunol 2020;16(5):465-70.

Samineni D, **Desai PB, Sallans L, Fichtenbaum CJ**. Steady-state pharmacokinetic interactions of darunavir/ritonavir with lipid-lowering agent rosuvastatin. J Clin Pharmacol 2012;52(6):922-31.

Scandinavian Simvastatin Survival Study (4S). Randomised trial of cholesterol lowering in 4444 patients with coronary heart disease: the Scandinavian Simvastatin Survival Study (4S). Lancet 1994;344(8934):1383-9.

Shepherd J. The West of Scotland Coronary Prevention Study: a trial of cholesterol reduction in Scottish men. Am J Cardiol 1995;76(9):113C-117C.

Shimojima M, **Kawashiri MA, Nitta Y,** et al. Rapid changes in plaque composition and morphology after intensive lipid lowering therapy: study with serial coronary CT angiography. Am J Cardiovasc Dis 2012;2(2):84-8.

Silverberg MJ, **Leyden W, Hurley L,** et al. Response to newly prescribed lipid-lowering therapy in patients with and without HIV infection. Ann Intern Med 2009;150(5):301-13.

Simon TG, **King LY, Zheng H, Chung RT**. Statin use is associated with a reduced risk of fibrosis progression in chronic hepatitis C. J Hepatol 2015;62(1):18-23.

Singh P, **Kohr D, Kaps M, Blaes F.** Influence of statins on MHC class I expression. Ann N Y Acad Sci 2009;1173:746-51.

Singh S, **Willig JH, Mugavero MJ,** et al. Comparative effectiveness and toxicity of statins among HIV-infected patients. Clin Infect Dis 2011;52(3):387-95.

Spagnuolo V, **Galli L, Poli A,** et al. Association of statins and antiretroviral drugs with the onset of type 2 diabetes among HIV-1-infected patients. BMC Infect Dis 2017;17(1)1:43.

Stein JH, **Brown TT, Ribaudo HJ,** et al. Ultrasonographic measures of cardiovascular disease risk in antiretroviral treatment-naive individuals with HIV infection. AIDS 2013;27(6):929-37.

Stone NJ, **Robinson JG, Lichtenstein AH,** et al. **American College of Cardiology/American Heart Association Task Force on Practice Guidelines.** 2013 ACC/AHA Guidelines on the treatment of blood cholesterol to reduce atherosclerotic cardiovascular risk in adults: a report of the American College of Cardiology/American Heart Association Task Force on Practice Guidelines. J Am Coll Cardiol 2014;63(25 Pt B):2889-934.

Strategies for Management of Antiretroviral Therapy (SMART) Study Group; El-Sadr WM, Lundgren JD, Neaton JD, et al. CD4+ count-guided interruption of antiretroviral treatment. N Engl J Med 2006;355(22):2283-96.

Subramanian S, **Tawakol A, Burdo TH,** et al. Arterial inflammation in patients with HIV. JAMA 2012;308(4):379-86.

Tarantino G, **Finelli C, Colao A,** et al. Are hepatic steatosis and carotid intima media thickness associated in obese patients with normal or slightly elevated gamma-glutamyl-transferase? J Transl Med 2012;10:50.

Tawakol A, **Fayad ZA, Mogg R,** et al. Intensification of statin therapy results in a rapid reduction in atherosclerotic inflammation: results of a multicenter fluorodeoxyglucose-positron emission tomography/computed tomography feasibility study. J Am Coll Cardiol 2013;62(10):909-17.

Tenorio AR, **Zheng Y, Bosch RJ,** et al. Soluble markers of inflammation and coagulation but not T-cell activation predict non-AIDS-defining morbid events during suppressive antiretroviral treatment. J Infect Dis 2014;210(8):1248-59.

Teramoto T, **Shimano H, Yokote K, Urashima M.** New evidence on pitavastatin: efficacy and safety in clinical studies. Expert Opin Pharmacother 2010;11(5):817-28.

Toribio M, Fitch KV, Sanchez L, et al. Effects of pitavastatin and pravastatin on markers of immune activation and arterial inflammation in HIV. AIDS 2017,31(6):797-806.

Triant VA, **Lee H, Hadigan C, Grinspoon SK,** et al. Increased acute myocardial infarction rates and cardiovascular risk factors among patients with human immunodeficiency virus disease. J Clin Endocrinol Metab 2007;92(7):2506-12.

Triant VA, **Regan S, Lee H,** et al. Association of immunologic and virologic factors with myocardial infarction rates in a US healthcare system. J Acquir Immune Defic Syndr 2010;55(5):615-9.

Tseng ZH, **Secemsky EA, Dowdy D,** et al. Sudden cardiac death in patients with human immunodeficiency virus infection. J Am Coll Cardiol 2012;59(21):1891-6.

Vasankari T, **Ahotupa M, Toikka J,** et al. Oxidized LDL and thickness of carotid intima-media are associated with coronary atherosclerosis in middle-aged men: lower levels of oxidized LDL with statin therapy. Atherosclerosis 2001;155(2):403-12.

Veillard, NR, **Braunersreuther V, Arnaud C,** et al. Simvastatin modulates chemokine and chemokine receptor expression by geranylgeranyl isoprenoid pathway in human endothelial cells and macrophages. Atherosclerosis 2006;188(1):51-8.

Wada N, **Jacobson LP, Cohen M,** et al. Cause-specific life expectancies after 35 years of age for human immunodeficiency syndrome-infected and human immunodeficiency syndrome-negative individuals followed simultaneously in long-term cohort studies, 1984-2008. Am J Epidemiol 2013;177(2):116-25.

Waehre T, **Damas JK, Gullestad L,** et al. Hydroxymethylglutaryl coenzyme a reductase inhibitors down-regulate chemokines and chemokine receptors in patients with coronary artery disease. J Am Coll Cardiol 2003;41:1460-7.

Walker PGT, Whittaker C, Watson OJ, et al. The impact of COVID-19 and strategies for mitigation and suppression in low- and middle-income countries. Science 2020;369(6502):413-22.

Wang W, **Zhao C, Zhou J,** et al. Simvastatin ameliorates liver fibrosis via mediating nitric oxide synthase in rats with non-alcoholic steatohepatitis-related liver fibrosis. PLoS One 2013;8(10):e76538.

Weber R, **Ruppik M, Rickenbach M,** et al. Swiss HIV Cohort Study (SHCS). Decreasing mortality and changing patterns of causes of death in the Swiss HIV Cohort Study. HIV Med 2013;14(4):195-207.

Willig JH, **Jackson DA, Westfall AO,** et al. Clinical inertia in the management of low-density lipoprotein abnormalities in an HIV clinic. Clin Infect Dis 2008;46(8):1315-8.

Wong WW, **Dimitroulakos J, Minden MD, Penn LZ**. HMG-CoA reductase inhibitors and the malignant cell: the statin family of drugs as triggers of tumor-specific apoptosis. Leukemia 2002;16(4):508-19.

WHO. World Health Organization. HIV Epidemiology 2012. http://www.who.int/hiv/data/en/. Site accessed June 30, 2013.

Xu Z, Peng C, Shi Y, et al. Nelfinavir was predicted to be a potential inhibitor of 2019-nCov main protease by an integrative approach combining homology modelling, molecular docking and binding free energy calculation. bioRxiv 2020.01.27.921627; doi: https://doi.org/10.1101/2020.01.27.921627.

Yamakawa T, **Takano T, Tanaka S,** et al. Influence of pitavastatin on glucose tolerance in patients with type 2 diabetes mellitus. J Atheroscler Thromb 2008;15(5):269-75.

Yarasheski KE, **Laciny E, Overton ET,** et al. 18FDG PET-CT imaging detects arterial inflammation and early atherosclerosis in HIV-infected adults with cardiovascular disease risk factors. J Inflamm (Lond) 2012;9(1):26.

Yasui Y, **Suzuki R, Miyamoto S,** et al. A lipophilic statin, pitavastatin, suppresses inflammation-associated mouse colon carcinogenesis. Int J Cancer 2007;121(10):2331-9.

Yokote K, **Saito Y;** CHIBA. Influence of statins on glucose tolerance in patients with type 2 diabetes mellitus: subanalysis of the collaborative study on hypercholesterolemia drug intervention and their benefits for atherosclerosis prevention (CHIBA study). J Atheroscler Thromb 2009;16(3):297-98.

Zanni MV, Abbara S, Lo J, Wai B, Hark D, Marmarelis E, Grinspoon SK. Increased coronary atherosclerotic plaque vulnerability by coronary computed tomography angiography in HIV-infected men. AIDS 2013;27(8):1263-72.

Zanni MV, Grinspoon SK. HIV-specific immune dysregulation and atherosclerosis. Curr HIV/AIDS Rep 2012;9(3):200-205.

Zheng C, **Azcutia V, Aikawa E,** et al. Statins suppress apolipoprotein CIII-induced vascular endothelial cell activation and monocyte adhesion. Eur Heart J 2013;34(8):615-24.

Zheng YY, Ma YT, Zhang JY, Xie X. COVID-19 and the cardiovascular system. Nat Rev Cardiol 2020;17:259260.

Zineh I, **Luo X, Welder GJ,** et al. Modulatory effects of atorvastatin on endothelial cell-derived chemokines, cytokines, and angiogenic factors. Pharmacotherapy 2006;26(3):333-40.

APPENDIX I: SAMPLE INFORMED CONSENT

For the REPRIEVE (A5332) Protocol, **FINAL Version 6.0**, **16May2022**

Randomized Trial to Prevent Vascular Events in HIV (The REPRIEVE Study)

INTRODUCTION

You are being asked to take part in this research study because you are **living** with the human immunodeficiency virus (HIV), the virus that causes AIDS, and you are taking HIV medications.

This study is sponsored by the National Institutes of Health (NIH). The doctor in charge of this study at this site is: (insert name of Principal Investigator). Before you decide if you want to be a part of this study, we want you to know about the study.

WHY IS THIS STUDY BEING DONE?

Since people started taking HIV medications, illness from AIDS has decreased, but other serious diseases, like heart disease, have increased. HIV causes inflammation (irritation) inside the body that cannot be felt but can be measured. These tests will be described later in this consent form. Inflammation may contribute to diseases such as heart disease that have become some of the leading causes of death in people with HIV **(PWH)**. HIV medications can lower inflammation somewhat, however sometimes the levels of inflammation can remain higher compared to people **without** HIV.

Statins are a group of medicines used to lower the levels of cholesterol and triglycerides (fat in the blood) that people make and to prevent heart-related disease events such as heart attacks in persons with high risk for heart attacks. Studies have shown that statins may have other benefits. For example, by decreasing levels of inflammation, statins may have an effect to protect against heart disease and its related events. In addition, statins may have some beneficial effects on some other diseases like some cancers or kidney problems.

The most recent guidelines from the American College of Cardiology and the American Heart Association (ACC/AHA) recommend the use of statins if someone is at risk of heart-related disease based on many different factors, including the use of a risk calculator based on known risk factors including gender, age, race, cholesterol levels, tobacco use, diabetes, and hypertension that estimates the 10-year risk of heart attack, stroke, or other event (the ASCVD risk score). HIV infection is not included in the risk calculator. The current risk calculators may not accurately predict the risk in **PWH**. However, HIV infection, HIV medications, and chronic inflammation may put you at higher risk for these diseases, although we do not know if you would benefit from taking a statin. You are eligible for this study because you are in a low-to-moderate-risk group for heart disease under the current guidelines, and there is no consensus about whether **PWH** in this group should take statins. Your participation in this study will help us determine if the use of statins can prevent heart-related disease among **PWH**. The results of this study may help to create guidelines for the prevention of heart disease in HIV infection.

For HIV-negative individuals with an ASCVD risk score between 7.5% and 15%, the current guidelines recommend a discussion between the health care provider and patients about the risks and benefits of statin therapy and recommend initiating statins based on available clinical trial data. However, for **PWH** with a moderate-risk for heart disease (a risk score between 7.5% and 15%), no currently available data clearly tell us whether the benefits of statin therapy outweigh the risks, including adverse effects and potential drug-drug interactions. Some health care providers may elect to treat **PWH** in this moderate-risk range with statin therapy, and this option may be available to you, rather than participating in the trial. Ultimately, the results of this trial will provide data to guide the use of statins for **PWH**.

Pitavastatin is a statin that, along with a diet, has been approved by the US Food and Drug Administration for the treatment of high cholesterol. It also lowers triglyceride levels in the blood. It has not been studied to see if it reduces heart-related disease or death. Pitavastatin was chosen because there are thought to be few interactions between pitavastatin and commonly used HIV medications.

The main purpose of this clinical trial is to see if pitavastatin can prevent heart disease and heart-related deaths in **PWH** who are taking HIV medications. We will also study the safety of pitavastatin.

HOW MANY PEOPLE WILL BE IN THIS STUDY?

About 7500 people will take part in this study.

WHAT DO I HAVE TO DO IF I AM IN THIS STUDY?

<u>Study visits</u>
If you enter the study, you will be seen in the clinic about 6 times the first year. After that, the study visits are every 4 months for the next **6-10** years **depending on when you join**. The study staff will tell you about how long each visit will be. More details about the visits and procedures are below.

<u>If you do not enter the study</u>
If you decide not to take part in this study after signing the consent form, or if you do not meet the eligibility requirements, we will still use some of your information. As part of this screening visit, some demographic (for example, age, gender, race), clinical (for example, disease condition, diagnosis), and laboratory (for example, safety tests) information is being collected from you so that AIDS Clinical Trials Group (ACTG) researchers may help determine whether there are patterns or common reasons why people do not join a study.

Study drugs

If you enter the study, you will be randomly assigned (as if by the toss of a coin) to get either pitavastatin or a placebo for pitavastatin. The placebo is a tablet that looks just like pitavastatin but does not contain any active medication. Therefore, there is a chance that if you are randomized to the placebo you will receive no treatment during your participation in the study. We use placebos in clinical studies to learn if the effects seen in the trial are truly from the study medicine or from other reasons. Neither you nor the study staff will know your assignment. You will not find out your assignment until after the entire study is over and the results of the study are known. You and your doctor can be told of the assignment at any point if it is necessary for your health.

You will take the study medicine (either pitavastatin or the placebo for pitavastatin) once a day, every day, throughout the study period, with or without food. The dose is 4 mg. We recommend that you take the study medicine at the same time each day. These drugs are provided by the study. It is very important that you take your medicines as directed. At every visit after entry, we will ask you about how you have been taking your study drugs. Antiretroviral drugs (treatment for HIV) will not be provided by the study.

Study procedures

The study staff can answer any questions you have about individual study visits and the procedures. The table below can be used as a quick reference, along with the explanations that follow.

Appendix I, Table 1: Study Procedures

Procedure	Screening[1]	Entry[2]	Month 1	Visits every 4 months (starting at month 4)	Annual visits (starting at month 12)	Final visit
Physical exam	X		X		X	X
Heart disease risk assessment	X					
Heart disease risk factors	X					
Diet and exercise questions		X				X
Dispense lifestyle information		X			X	X
Health and medicine questions	X[3]	X	X	X[5]	X[5]	X[3, 5]
Blood collected	X	X	X		X	X
Urine collected		X			X[4]	
Pregnancy test	X	X	X	X	X	X
Electrocardiogram		X				
Pills dispensed		X		X	X	

[1] Screening visit: before you can enter the study, you will need to come to the clinic to have evaluations done to make sure that you can take part in the study.

[2] Entry visit: if you meet the entry requirements, you will enroll in the study.

[3] **This will also include hormonal assessment for women.**

[4] *ACTG Sites ONLY: Urine will be collected at months 12, 24, and 48 only for people enrolled in REPRIEVE (A5332) before January 1, 2018.*
[5] ***This will include a COVID-19 assessment for all participants.***

If you leave the study early, or have to stop taking the study medication before the study is over, you will have the procedures listed in the table below.
Appendix I, Table 2: Discontinuation Study Procedures

Procedure	Stopping the study or the study treatment early
Physical exam	X
Health and medicine questions	X[1]
Pregnancy test	X
Blood collected	X
Fasting blood tests	X

[1] **This will also include hormonal assessment for women and a COVID-19 assessment for all participants.**

Explanation of study procedures

Physical exam
You will have a physical exam at screening. At other visits after entry, the extent of the exam will depend on how you are feeling at that visit. You will have vital signs taken, including, blood pressure and pulse. You will have measurements taken of your waist and height and weight. You will be asked questions about your health and medicines.

Heart disease risk assessment
At screening we will ask specific questions to assess eligibility based on cardiovascular disease risk. At screening you will also be asked about cardiovascular risk factors including your family history, smoking, alcohol use, substance use, diet, and exercise.

Lifestyle /risk reduction counseling
If you join the study, you will be given information about a healthy diet and the importance of exercise, smoking cessation, and taking your antiretroviral therapy and study medication as prescribed. We will provide this information at all annual visits.

ECG
An electrocardiogram, or ECG, will be done at entry. An ECG is an electrical tracing of your heart that can show how hard it is working. You will have to lie very still for up to 10 minutes while the ECG is being done.

Blood collected
Blood will be collected from you for different tests if they are not available as part of your routine medical care or for safety reasons. These include routine tests to evaluate your blood counts, liver, and kidney function.

At screen we will use the results of your liver function tests done as part of routine care by your medical provider. At month 1 and month 12 we will collect blood from you to evaluate your liver

function. This test is required as part of your participation in the study. Approximately 1 teaspoon of blood at each of these visits will be collected for this test.

At screen and the end of study visit we will use the results of your CBC (blood count) and kidney function done as part of routine care by your medical provider.
At screen and annual visits we will use the results of your CD4 T-cell count (how many infection fighting cells are in your blood) done as part of routine care by your medical provider.

At entry and annual visits we will use the results of your HIV viral load (how much HIV is in your blood) done as part of your routine care by your medical provider.

At screen we will use the results of your cholesterol (fat found in your blood) levels done as part of routine care by your medical provider.

You will be told the results of these routine tests.

At entry, all annual visits, and the end of study visit, some blood will be collected and stored for tests that will be done later on in the study or after the study is over. These tests will measure the levels of fat and sugar in your blood. Some of these tests will be used for metabolic blood tests (measures how your body uses the food that you eat). You do not need to agree to store this blood to join the study and you may change your mind about storing your blood at any time. Your blood may be stored (with usual protectors of identity) for an indefinite length of time. You will not be told of the results of the research done on your blood.

At each of these visits, approximately 2 teaspoons of your blood will be collected and stored for these purposes.

Do you agree to let us store your samples for tests to measure the levels of fat and sugar in your blood?

________ YES ________ NO _______Initials

At entry, **all** annual visits (as indicated in the Study Procedures table), and the end of study visit, some of your blood will be collected and stored for future REPRIEVE-approved research on conditions including cardiovascular disease, HIV, inflammation, cancer or statin medications. You do not need to agree to store this blood to join the study and you may change your mind about storing your blood at any time. Your blood may be stored (with the usual protectors of identity) for an indefinite length of time. You will not be told of the results of the research done on your blood.

Up to **15** teaspoons of blood will be collected at **each of these visits** for these purposes.

Do you agree to let us store your samples for future research on conditions including cardiovascular disease, HIV, inflammation, cancer, and statin medications?
________ YES ________ NO _______Initials

All ACTG sites will add to their local consent: Approximately 1 teaspoon of blood will be collected to look at genes that may affect your risk for cardiovascular disease and how statins work in your body. Genetic testing is a laboratory test that looks at differences in people's genes. Your body, like all living things, is made up of cells, and cells contain deoxyribonucleic acid, also known as "DNA." DNA is like a string of information put together in a certain order. Parts of the string make up "genes." Genes contain instructions on how to make your body work and fight disease. The testing in this study will focus on certain genes that are known to have an effect on cardiovascular disease and how your body uses statins. New genes of interest may be identified in the future and may also be looked at.

Your body's genetic makeup is unique to you, so there is a risk with genetic research that even with all of the security measures in place, someone using your samples or genetic information may still find out which information is yours. However, this risk today is very small, but it may increase with time since science and technology are developing rapidly.

ONLY ACTG sites in the US will add to their local consent: In the event that your genetic information becomes linked to your name, the US federal law called the Genetic Information Nondiscrimination Act (GINA) helps protect you. This law prohibits health insurance companies, group health plans, and most employers from denying services based on your genetic information. However, GINA does not protect against discrimination by companies that sell life insurance, disability insurance, or long-term care insurance.

All ACTG sites will add this participant opt-out to their local consent: We would like to use some of the blood we collect to look at your genes (DNA). Do you agree to this genotyping?

________ YES ________ NO ________Initials

If at a later date you change your mind and want your samples destroyed, contact the research staff. There are two ways to withdraw your permission. You could allow researchers to remove all your personal identifiers from your samples, so that they are not linked to you anymore. These samples will then become anonymous. Or, you can ask researchers to destroy your samples, so that they cannot be used for future research. However, in either case, researchers will not be able to destroy samples or information from research that is already underway.

For all Non-US sites to add to their local consent: Your samples may be shipped and stored outside of your country and may be used by researchers outside of your country.

Urine collected
Urine will be collected at entry to check for protein in your urine. The results from this test will not be known immediately; therefore we cannot make sure that you will be told the results of this test.

Only ACTG Sites will add to their local consent: For participants enrolled in RERIEVE (A5332) before January 1, 2018, urine will also be collected at months 12, 24, and 48 to check measures of kidney function.

Fasting blood tests
Before the screen, entry and all annual visits you should not eat or drink anything, including food, beverages, candy, or gum for 8 hours before your visit. You are encouraged to drink water before your visits. If you are not fasting we will ask you to return while fasting to have your blood drawn within 30 days of the study visit.

Study drugs given to you
Study drugs will be given to you at entry and every 4 months. No study drugs will be given to you at your final study visit.

Questionnaires
You will be asked questions about your diet and exercise at entry and this will be repeated the final study visit.

HOW LONG WILL I BE IN THIS STUDY?

You will be in this study about **6-10** years (**72-120** months) depending on when you join.

WHY WOULD THE DOCTOR TAKE ME OFF THIS STUDY EARLY?

The study doctor may need to take you off the study early without your permission if:
- the doctor thinks it is in your best interest
- the study is cancelled
- you are not able to attend the study visits as required by the study

The study doctor may also need to take you off the study drug without your permission if:
- you are not able to take the study drug as required by the study
- continuing the study drug may be harmful to you
- you need a treatment that you may not take while on the study
- you become pregnant

If you must stop taking the study drug before the study is over, we will ask you to continue to be part of the study and return for some study visits and procedures.

If you have to permanently stop taking the study drug, or if you leave the study, how would pitavastatin be provided?

During the study:
If you must permanently stop taking study-provided pitavastatin before your study participation is over, the study staff will discuss other options that may be of benefit to you. If you discontinue active participation in the study, study staff will contact you, your provided contacts, the medical records department, or your health care provider annually to determine if you have had any heart disease, stroke, or vascular-related disease events or procedures, or if you have died (and the cause of death since last contact).

After the study:
After you have completed your study participation, the study will not be able to continue to provide you with the pitavastatin you received on the study. If continuing to take this or a similar drug would be of benefit to you, the study staff will discuss how you may be able to obtain the drug.

WHAT ARE THE RISKS OF THE STUDY?

The drug used in this study may have side effects, some of which are listed below. Please note that these lists do not include all the side effects seen with this drug. These lists include the more serious or common side effects with a known or possible relationship. If you have questions concerning the additional study drug side effects please ask the medical staff at your site.

There is a risk of serious or life-threatening side effects when non-study medications are taken with the study drug. For your safety, you must tell the study doctor or nurse about all medications you are taking before you start the study and also before starting any new medications while on the study. Also, you must tell the study doctor or nurse before enrolling in any other clinical trials while on this study.

Risks of Pitavastatin

- Muscle problems. Pitavastatin can occasionally cause serious muscle problems that can lead to kidney problems, including kidney failure and rarely, death.
- Liver problems. Pitavastatin can occasionally cause liver problems that may rarely be serious or cause death. Your study nurse or doctor will do blood tests to check your liver before you start taking pitavastatin and while you take it.
- Be sure to let your doctor or study nurse know immediately if you have any of these problems:
- ▪ Muscle problems like weakness, tenderness, or pains that happen without a good reason, especially if you also have a fever or feel more tired than usual.
- ▪ Nausea and vomiting.
- ▪ Passing brown or dark-colored urine.
- ▪ Feeling more tired than usual.
- ▪ Noticing the skin and whites of your eyes become yellow.
- ▪ Having stomach pain.

Other problems that have been caused by pitavastatin include headaches, rash (which rarely may be severe or fatal), severe allergic reaction or swelling, constipation, gas, diarrhea, pain or numbness in arms or legs, tendon rupture, urinary tract infection, dizziness, memory impairment, and depression. All of these problems are uncommon to rare.

Risks of drawing blood
Taking blood may cause some discomfort, lightheadedness, bleeding, swelling, or bruising where the needle enters the body, and in rare cases, fainting, or infection.

Risks of fasting
Some people find fasting to be bothersome. It may make some individuals feel anxious, irritable, or hungry. Patients who are required to take their morning medications with food should wait until after the visit has been completed to take their medications.

Risks of ECG
You may experience mild irritation, slight redness and itching on your skin where the electrodes from the electrocardiogram machine are placed.

ALL ACTG sites will add to their local consent:

Genetic Testing
The results of your genetic tests are for research purposes only and no individual results will be given back to you. The results of the genetic studies will never become a part of your medical record. We will protect your confidentiality to the fullest extent. Blood samples for genetic studies will be identified in a way in order to maintain your confidentiality.

Research study results will not be given to your family members, insurance companies, employers, or third parties without your written permission and approval of the Institutional Review Board at _________.

Unknown risks
Other side effects that are not known at this time could happen during the study. All drugs have a possible risk of an allergic reaction, which if not treated right away, could become life-threatening. During the study, you will be told about any new information that may affect your decision to stay in the study. If you decide to stay in the study, you will be asked to sign an updated consent form. If you decide to leave the study early, the study staff will talk with you about your treatment options.

ARE THERE RISKS RELATED TO PREGNANCY?

Pitavastatin is unsafe for unborn babies. The risks to the unborn baby include birth defects, premature delivery, or death. If you are having sex that could lead to pregnancy, you must agree not to become pregnant.
If you can become pregnant, you must have a pregnancy test before you enter this study and at every visit (1 teaspoon of blood or a urine specimen will be collected) and at any time that

pregnancy is suspected. This test must show that you are not pregnant. If you become pregnant or think you may be pregnant at any time during the study, tell your study staff right away. The study staff will talk to you about your choices.

Because of the risk involved, you and your partner must use at least one accepted form of birth control that you discuss with the study staff. You must start an accepted form of birth control at least two weeks before you start study drug and continue to use an accepted form of birth control until at least 6 weeks after you stop the study drug. If you are having sex that could lead to pregnancy, and do not use an accepted form of birth control, your study doctor will take you off of the study drug. You may choose from the birth control methods listed below:

- Condoms (male or female), with or without a spermicidal agent
- A diaphragm or cervical cap with spermicide
- An IUD (intrauterine device)
- Tubal ligation
- Tubal micro-inserts
- Hormone-based contraceptive

If you become pregnant while on study, the study staff would like to obtain information from you about the outcome of the pregnancy (even if it is after your participation in the study ends). If you are taking anti-HIV drugs when you become pregnant, your pregnancy will be reported to an international database that collects information about pregnancies in women taking anti-HIV drugs. This report will not use your name or other information that could be used to identify you.

Breastfeeding

It is not known whether the study drug pass through the breast milk and may cause harm to your infant. Women who start breastfeeding must stop taking the provided study drug.

ARE THERE BENEFITS TO TAKING PART IN THIS STUDY?

Studies have shown statins to provide a benefit in terms of preventing heart disease in **people without** HIV with inflammation, but the effects of statins to prevent heart disease in **PWH** is not known. If you take part in this study, there may be a direct benefit to you, but no guarantee can be made. You may benefit from learning about your risk of a cardiovascular event, but it is also possible that you may receive no benefit from being in this study either because the drug may not work or because you are assigned to placebo. Information learned from this study may help others who have HIV and are at risk of cardiovascular disease.

WHAT OTHER CHOICES DO I HAVE BESIDES THIS STUDY?

Instead of being in this study, you have the choice of:

- Treatment with prescription drugs available to you
- Treatment with experimental drugs, if you qualify
- No treatment
- Continue routine medical care from your primary care provider

- Joining another trial if you qualify
- Not getting medical care

Please talk to your study doctor about these and other choices available to you. Your study doctor will explain the risks and benefits of these choices.

WHAT ABOUT CONFIDENTIALITY?

For ALL US sites: We will do everything we can to protect your privacy. In addition to the efforts of the study staff to help keep your personal information private, we have gotten a Certificate of Confidentiality from the US Federal Government. This certificate means that researchers cannot be forced to tell people who are not connected with this study, such as the court system, about your participation. Also, any publication of this study will not use your name or identify you personally.

People who may review your records include the AIDS Clinical Trials Group (ACTG), Office for Human Research Protections (OHRP) or other government agencies as part of their duties, Food and Drug Administration (FDA) (insert name of site) IRB/EC (a group that protects the rights and well-being of people in research), National Institutes of Health (NIH), other local, US, and international regulatory entities, study staff, study monitors, the drug companies supporting this study, and their designees. Having a Certificate of Confidentiality does not prevent you from releasing information about yourself and your participation in the study.

Even with the Certificate of Confidentiality, if the study staff learns of possible child abuse and/or neglect or a risk of harm to yourself or others, we will be required to tell the proper authorities.

OR

For ALL Non-US sites: Efforts will be made to keep your personal information confidential. We cannot guarantee absolute confidentiality. Your personal information may be disclosed if required by law. Any publication of this study will not use your name or identify you personally.

Your records may be reviewed by the ACTG, OHRP, FDA, (insert name of site) IRB/EC, National Institutes of Health (NIH), other local, US, and international regulatory entities, national regulatory/health agencies, study staff, study monitors, and the drug company supporting this study and its designees.

All information collected about you as part of the study will be sent securely to the ACTG statistical and data management center in the United States for combining with information from other study participants and statistical analysis of study results. Your name and other personal identifiers will not be sent. Your research site is responsible for sending your information in accordance with the laws, regulations, and policies of your country and research site. (No new procedures in data collection)

A description of this clinical trial will be available on **https://**www.ClinicalTrials.gov, as required by US law. This web site will not include information that can identify you. At most, the web site will include a summary of the results. You can search this web site at any time.

WHAT IF WE CAN NO LONGER REACH YOU DURING THE STUDY?

In the event you cannot be reached after multiple attempts to contact you, study staff may try to contact you through alternate phone numbers of family, friends, case manager, or acquaintances obtained at screening and updated at each visit. If you are unable to be reached through the alternate contacts we will attempt to obtain information about you from other sources such as family members, other designated contacts, or clinic records. The purpose of obtaining this information is to determine if you have died and the cause of death since last contact and if you have had any heart disease, stroke, or vascular-related disease events or procedures.

Contacting Your Health Care Providers
Sites to modify per local requirements for obtaining health care records: As mentioned above, we do not currently have data to tell us if **PWH** with moderate cardiovascular disease risk by the ASCVD risk calculator should be treated with statins. If you are in this moderate-risk group, with a risk score between 7.5% and 15%, we would like to inform your health care provider(s) about the rationale for the trial, but we need your permission to share this information.

Also, with your permission, for which you would need to sign a waiver, study staff may contact your health care providers regarding any clinical diagnoses you may develop during the study, including heart related diagnoses and other diagnoses, such as HIV, kidney, liver or cancer diagnoses.

Will you allow us to contact your health care provider(s) to share information regarding your cardiovascular risk score and rationale for the trial, and to provide information regarding clinical diagnoses and to let your doctor know that he/she could object to your taking part in this study since you have a chance of getting placebo instead of treatment with statins?

________ YES ________ NO _______Initials

WHAT ARE THE COSTS TO ME?

There will be no cost to you for the study drugs, the study visits, physical examinations, laboratory tests or other tests required by the study. You or your insurance company, or your health care system will be responsible for the costs of your regular medical care as well as for the costs of drugs not given by the study.

Taking part in this study may lead to added costs to you and your insurance company. In some cases, it is possible that your insurance company will not pay for these costs because you are taking part in a research study.

WILL I RECEIVE ANY PAYMENT?

You will be paid ________ at the entry visit, the month 1 visit, and annual visits thereafter for participation in the study. *(The team recommends compensation to participants of $25 at **every annual visit and study termination visit**). Sites will be reimbursed for the expense.)*

WHAT HAPPENS IF I AM INJURED?

If you are injured as a result of being in this study, you will be given immediate treatment for your injuries.

For all sites: NIH does not have a mechanism to provide direct compensation for research-related injury.

[Sites: Please modify (if necessary) and insert one of these two statements, as appropriate to your site. If your site is required to carry CTI, this must be indicated in the informed consent.]

- ***This site has clinical trials insurance. This insurance will allow the site to provide you with monetary compensation if you suffer harm as a result of participating in this research study.***
  OR
- ***The cost for this treatment will be charged to you or your insurance company. There is no program for compensation either through this institution or the NIH.]***

You will not be giving up any of your legal rights by signing this consent form.

WHAT ARE MY RIGHTS AS A RESEARCH PARTICIPANT?

Taking part in this study is completely voluntary. You may choose not to take part in this study or leave this study at any time. Your decision will not have any impact on your participation in other studies conducted by NIH and will not result in any penalty or loss of benefits to which you are otherwise entitled.

We will tell you about new information from this or other studies that may affect your health, welfare, or willingness to stay in this study. If you want the results of the study, let the study staff know.

WHAT DO I DO IF I HAVE QUESTIONS OR PROBLEMS?

For questions about this study or a research-related injury, contact:

- name of the investigator or other study staff

- telephone number of above

For questions about your rights as a research participant, contact:

- name or title of person on the Institutional Review Board (IRB/EC) or other organization appropriate for the site
- telephone number of above

SIGNATURE PAGE

If you have read this consent form (or had it explained to you), all your questions have been answered and you agree to take part in this study, please sign your name below.

Participant's Name (print)	Participant's Signature and Date
Participant's Legal Representative (print) (As appropriate)	Legal Representative's Signature and Date
Study Staff Conducting Consent Discussion (print)	Study Staff's Signature and Date
Witness's Name (print) (As appropriate)	Witness's Signature and Date

APPENDIX IA: SAMPLE INFORMED CONSENT ADDENDUM

**For the Randomized Trial to Prevent Vascular Events in HIV − (REPRIEVE) (A5332)
FINAL Version 6.0, 16May2022**

SHORT TITLE FOR THE STUDY: REPRIEVE

You are being asked to sign this consent addendum because you are participating in a research study at ______________, entitled "Randomized Trial to Prevent Vascular Events in HIV − REPRIEVE (A5332)."

The purpose of this addendum is to include information about sharing your de-identified genetic information. "De-identified" means that all information that identifies you, for example, your name, medical record number, and date of birth, has been removed.

The genetic information was obtained from the blood sample that was collected from you at the entry visit. You consented to provide this sample in the original consent.

Except for the activities described in this addendum, the terms of your original consent form remain in full effect, including that research study results will not be given to your family members, insurance companies, or employers without your written permission and approval of the Institutional Review Board/Ethics Committee at ______________________.

**Please read this consent addendum carefully and take your time making your decision. As your study doctor or study staff discusses this addendum with you, please ask them to explain any words or information that you do not clearly understand.
You do not have to sign this addendum to remain in REPRIEVE (A5332).**

SHARING OF YOUR GENETIC INFORMATION

Illness and health are affected by the genes or genetic information we have inside of us. The genetic information we collect in this study may help advance other types of research. The National Institutes of Health (NIH) has established a program to let researchers share genetic information from different studies to help us learn more from each study that is funded by the NIH. Your genetic information will be shared with other researchers through a secure, controlled-access database supported by the NIH.

When we share your genetic information with other researchers through a secure, controlled-access database supported by the NIH, including whole genome studies (such as determining your complete DNA sequence at a single time), we will remove all information that identifies you. This de-identified information may be used in other research. We do not think that there will be further risks to your privacy and confidentiality by sharing your genetic information with this controlled-access database. However, we cannot predict how genetic information will be used in the future.

Your name or other directly identifiable information will not be given to the controlled-access database supported by the NIH. There are many other safeguards in place to protect your information while it is stored in the controlled-access database and used for research. Even so, it may become possible in the future that someone can re-identify your information. Also, using the controlled-access database, researchers may publish or present combined results of their research, but they will not include your name or other information that can identify you.

There is no expected direct benefit to you from research using your genetic information. You can withdraw consent for sharing your genetic information at any time. However, we cannot get back any information that has already been shared.

Do you agree to share your genetic information?

________YES ___________NO _________Initials

OTHER
All other information that is contained in the REPRIEVE (A5332) consent that you signed also applies to this consent addendum. A copy of this signed consent will be provided for you as a reference at the time you consent to the information in this addendum.

QUESTIONS REGARDING THIS CONSENT ADDENDUM
If you have any questions, concerns, or complaints concerning this consent addendum, please contact Dr. (name) at (number) during regular business hours, and at (pager) after hours and on weekends and holidays.

For questions about your rights as a research participant, or to discuss questions, concerns, or suggestions related to the research or this consent addendum, or to obtain information or offer input about the research, please contact the _____________Institutional Review Board/Ethics Committee Office at (number).

STATEMENT OF CONSENT
"The purpose of this consent addendum has been explained to me. I have been allowed to ask questions, and my questions have been answered to my satisfaction. I have been told whom to contact if I have questions, to discuss problems, concerns, or suggestions related to the research or this addendum, or to obtain information or offer input about the research. I have read this addendum and agree to the choices I have indicated above, with the understanding that I may withdraw my consent at any time. I have been told that I will be given a signed and dated copy of this addendum."

SIGNATURE PAGE

If you have read this consent form (or had it explained to you), all your questions have been answered, and you agree to take part in this study, please sign your name below.

**Participant's Name (print)**	**Participant's Signature and Date**
**Participant's Legal Representative (print)**<br>**(As appropriate)**	**Legal Representative's Signature and Date**
**Study Staff Conducting Consent Discussion (print)**	**Study Staff's Signature and Date**
**Witness's Name (print)**<br>**(As appropriate)**	**Witness's Signature and Date**

APPENDIX II: THE MECHANISTIC SUBSTUDY OF REPRIEVE (A5333s)

Effects of Pitavastatin on Coronary Artery Disease and Inflammatory Biomarkers: Mechanistic Substudy of REPRIEVE

Principal Investigators: Steven Grinspoon, MD
Pamela Douglas, MD
Udo Hoffmann, MD, MPH
Heather Ribaudo, PhD

AIDS Clinical Trials Group Investigators: Turner Overton, MD
Carl Fichtenbaum, MD
Judith Aberg, MD
Markella Zanni, MD

Data Coordinating Center: Harvard School of Public Health

Study Funders: National Heart, Lung, and Blood Institute
National Institute of Allergy and Infectious Diseases
Office of AIDS Research, National Institutes of Health

Industry Support: Kowa Pharmaceuticals America
Gilead Sciences
ViiV Healthcare

NHLBI Program and Medical Officer: Patrice M. Desvigne-Nickens, MD

DAIDS Medical Officer: Karin L. Klingman, MD

IND Sponsor: Division of AIDS, NIAID, NIH

IND Number: 119127

FINAL Version 6.0
16May2022

SITES PARTICIPATING IN THE MECHANISTIC SUBSTUDY of REPRIEVE (A5333s)

The Mechanistic Substudy of REPRIEVE (A5333s) is open to selected sites that are participating in the REPRIEVE (A5332) study.

EXECUTIVE SUMMARY

Title	Effects of Pitavastatin on Coronary Artery Disease and Inflammatory Biomarkers: Mechanistic Substudy of REPRIEVE (A5333s)
Indication	Assess the effects of statins on critical plaque and inflammatory characteristics to understand mechanism of action in HIV
Location	Selected sites of the REPRIEVE (A5332) study
Brief Rationale	The Mechanistic Substudy of REPRIEVE (A5333s) will determine, among HIV-infected persons, potential statin effects to halt progression of non-calcified atherosclerotic plaque and to stabilize morphologic features of plaque vulnerability. Moreover, the study will identify biological factors mediating these changes – be it lipid parameters, such as LDL cholesterol, or markers of inflammation and immune activation.
Study Design and Duration	Randomized, placebo-controlled multicenter substudy of REPRIEVE (A5332) in 800 subjects, with individual subjects participating in the substudy for 2 years.
Treatment	Pitavastatin 4 mg PO daily or placebo for pitavastatin.
Primary Objective	To determine the effects of pitavastatin on the morphology and composition of non-calcified coronary atherosclerotic plaque (NCP), including the progression of plaque volume and whether these effects are modulated by markers of inflammation and immune activation.
Key Secondary Objectives	1. The effects of pitavastatin on the progression of high risk plaque features including low attenuation plaque and positive remodeling.<br>2. The effects of pitavastatin on detailed markers of immune activation, immune activation, inflammation, coagulation, and traditional CVD risk indices including detailed parameters of glucose homeostasis (insulin, glucose and related indices of insulin resistance such as HOMA-IR, HgbA1c).<br>3. The relative contributions of baseline and pitavastatin induced changes in HIV-specific immune activation and traditional risk factors, including LDL, on the presence and progression of coronary plaque and high risk morphological features in HIV.
Primary Endpoint	Noncalcified plaque volume on coronary computed tomography angiography (CCTA)
Secondary Endpoints	High risk plaque features on CCTA<br>Detailed immune phenotyping measures and inflammatory and coagulation indices as well as detailed measures of glucose homeostasis
Abbreviated Study Flow	Subjects will be coenrolled at specific sites participating in the Mechanistic Substudy of REPRIEVE (A5333s). Enrollment and treatment will be identical as in REPRIEVE (A5332), but endpoints will be collected over 2-year study duration for each enrolled patient.

1.0 HYPOTHESIS AND STUDY OBJECTIVES

1.1 Primary Mechanistic Hypothesis

Statin therapy will reduce progression of non-calcified coronary atherosclerotic plaque volume over two years as measured by serial coronary computed tomography angiography (CCTA) as compared with placebo in HIV-infected patients on ART in whom traditional CVD risk is not significantly increased. The mechanisms underlying the effect of statins will include a) reduction in non-calcified coronary atherosclerotic plaque, b) reduction in vulnerability features of non-calcified coronary atherosclerotic plaque, and c) improvement in critical indices of immune activation and inflammation.

1.2 Secondary Mechanistic Hypotheses

1.2.1 Decreases in LDL cholesterol levels associated with statin therapy will be predictive of improvement in non-calcified coronary atherosclerotic plaque burden and/or vulnerability features.

1.2.2 Statin therapy will reduce indices of general inflammation, coagulation, monocyte activation, and arterial inflammation.

1.2.3 Statin therapy will reduce levels of pro-inflammatory monocyte populations.

1.2.4 Statin therapy will reduce levels of T-cell activation and exhaustion.

1.2.5 Changes in levels of immune activation and inflammatory markers will be associated with changes in morphology and composition of non-calcified coronary atherosclerotic plaque.

1.2.6 Statin therapy will not have a clinically significant effect on glucose and insulin resistance.

1.3 Primary Mechanistic Objective

To determine the effects of pitavastatin on the morphology and composition of non-calcified coronary atherosclerotic plaque (NCP), including the progression of plaque volume and whether these effects are modulated by markers of inflammation and immune activation.

1.4 Secondary Mechanistic Objectives

1.4.1 To determine the effects of pitavastatin on the progression of high risk plaque features including low attenuation plaque and positive remodeling.

1.4.2 To determine the effects of pitavastatin on detailed markers of immune activation, including immune function (CD4, viral load), immune activation (%CD14+CD16+ monocytes, sCD163, sCD14, MCP-1 and T-cell markers),

inflammation (Lp-PLA2, hsCRP, IL-6), coagulation (D-Dimer and tissue factor) and traditional CVD risk indices including detailed parameters of glucose homeostasis (insulin, glucose and related indices of insulin resistance such as HOMA-IR, HgbA1c).

1.4.3 To determine the relative contributions of baseline and pitavastatin induced changes in HIV-specific immune activation and traditional risk factors, including LDL, on the presence and progression of coronary plaque and high risk morphological features in HIV.

1.4.4 To collect blood to enable the evaluation of the relationship of host genetics to study endpoints in subsequent ancillary studies.

2.0 INTRODUCTION

2.1 Background

HIV-infected persons face a 1.5- to 2-fold risk of myocardial infarction (MI), even after controlling for increased prevalence of traditional CVD risk factors [Triant 2007; Freiberg 2013]. This increased risk is thought to be mediated, in large part, by immune activation and inflammation as emerging major contributors to the markedly increased prevalence of high risk coronary atherosclerosis in the HIV-infected population [Hsue 2012; Zanni 2012], such that young patients without known CVD and with relatively low Framingham risk scores may still be high-risk. Traditional CVD prevention paradigms tested in the HIV-negative population fail to adequately assess and specifically target HIV-associated immune-mediated CVD risk. The proposed randomized trial of statin therapy in HIV-infected individuals with minimal traditional cardiovascular risk leverages the LDL-lowering [Silverberg 2009] and immunomodulatory properties of statins [Kwak 2000], targeting both traditional CVD risk factors (dyslipidemia), and HIV-specific immune activation. Embedded in the larger events trial is a mechanistic sub-study in which coenrolled participants will undergo coronary CT angiography for determination of atherosclerotic plaque morphology and composition, as well as detailed profiling of lipid and inflammatory/immune parameters. The mechanistic sub-study will provide several key scientific insights of crucial relevance to predicting and preventing CVD in HIV. Data from the mechanistic sub-study will highlight whether CCTA-based plaque morphology relates to CVD events in HIV, independent of lipid and inflammatory/immune parameters, and whether potential effects of statins to stabilize atherosclerotic plaque morphology – rendering plaques less likely to rupture and cause acute myocardial infarction – are mediated primarily through lipid-lowering or through immunomodulatory effects.

2.1.1 HIV-infected patients have a novel phenotype of atherosclerotic plaque linked to immune activation

HIV-infected individuals without known CVD have been shown to have more non-calcified coronary atherosclerotic plaque relative to HIV-negative controls matched on traditional cardiovascular risk factors [Burdo 2011]. The significance of non-calcified plaque – namely, the higher likelihood it has to rupture, relative to calcified plaque – is highlighted by the MACE trial of over 5000 HIV-negative outpatients. This study showed that over 3 years of follow-up, 22.7% of patients with non-calcified plaque experienced a major adverse cardiac event compared with 5.5% of patients with calcified plaque [Hou 2012]. HIV-infected individuals without known CVD also have more vulnerable atherosclerotic plaque on coronary CTA relative to HIV-negative controls matched on traditional CVD risk factors. Features used to characterize vulnerability include low CT attenuation (correlating with necrotic lipid core) and positive remodeling (reflecting eccentric plaque extension) [Zanni 2013]. Among HIV-negative individuals with suspected CVD, low attenuation and positively remodeling have been demonstrated to prospectively predict the development of acute coronary syndrome (ACS). In a large study of over 1000 patients, Motoyama et al. demonstrated that in patients without significant CAD the occurrence of either low CT attenuation or positive remodeling was accompanied by a 22 fold increase in relative risk for suffering from an adverse cardiovascular event [Motoyama 2009]. These results were confirmed in another study that demonstrated that those persons with atherosclerotic plaques characterized by two vulnerability features were far more likely than individuals with atherosclerotic plaques absent of vulnerability features to develop ACS (22.2% vs. 0.5%, respectively) [Kitagawa 2009; Motoyama 2009]. Importantly, non-calcified and vulnerable plaque in HIV-infected individuals relates to levels of soluble CD163, a monocyte/macrophage activation marker [Burdo 2011; Zanni 2013]. This latter finding suggests that immune activation in HIV may be contributing to the development of a novel atherosclerotic plaque phenotype which is prone to rupture and result in acute MI. Numerous other studies have linked immune activation markers – including monocyte activation markers LPS, sCD14, sMCP-1, and sCD163, as well as T-cell activation markers – to subclinical atherosclerosis, cardiovascular events, and mortality [Merlini 2012; Kelesidis 2012; Sandler 2011; Kaplan 2011; Liu 1997; Hunt 2011; Giorgi 1999; Burdo 2011; Zanni 2013; Subramanian 2012].

2.1.2 Statin Effects

2.1.2.1 Statins lower LDL cholesterol and dampen immune activation

In the general population, statins have long been known to potently reduce LDL cholesterol and to prevent CVD events [Sacks 1996; Shepherd 1995; Downs 1998]. In addition, statins have pleiotropic anti-inflammatory and immunomodulatory characteristics, which may also contribute to cardio-protective effects. Indeed, *in vitro,* animal, and human studies have shown that statins decrease monocyte activation – reflected in a) decreased monocyte chemotaxis and endothelial

adhesion [Montecucco 2009; Fujino 2006; Han 2005] b) reduced monocyte uptake of oxidized LDL cholesterol [Han 2004]), and c) decreased monocyte secretion of cytokines/chemokines and matrix metalloproteinases [Guo 2009; Waehre 2003]. Moreover, statins decrease T-cell activation [Kwak 2000; Singh 2009; Bu 2010] while recruiting regulatory T cells [Mira 2008], and statins also suppress endothelial cell activation [Zheng 2013; Romano 2000; Mulhaupt 2003; Veillard 2006; Zineh 2006]. In HIV-infected persons, statins, and specifically pitavastatin, effectively lower LDL cholesterol [Silverberg 2009; Eckard 2014; Funderburg 2014, 2015; Aberg 2017]. In HIV-infected persons, statins also exert immunomodulatory effects to 1) decrease monocyte activation – reflected in decreased circulating levels of sCD14 and the macrophage-derived phospholipase, Lp-PLA2 [Eckard 2014; Funderburg 2014, 2015], and 2) reduce T-cell activation [Ganesan 2011; De Wit 2011].

2.1.2.2 Statins may stabilize rupture-prone coronary atherosclerotic plaque

Findings from observational CT imaging studies and large randomized IVUS trials demonstrate that statin therapy results in regression of NCP volume and potentially stabilizes coronary atherosclerotic plaque morphology in HIV-negative populations [Shimojima 2012; Inoue 2010; Kodama 2010; Nakamura 2008]. In addition, statins have been demonstrated to reduce atherosclerotic plaque inflammation on cardiac FDG-PET [Takawol 2013], and to reduce non-calcified plaque volume [Burgstahler 2007; Hiro 2009].

2.1.2.3 The effect of statins to stabilize rupture-prone atherosclerotic plaque may be mediated through LDL lowering and/or through the exertion of anti-inflammatory effects

The JUPITER trial was a landmark study in that it showed a significant effect of statin therapy to prevent CVD events among non-HIV patients without known heart disease, with relatively low LDL cholesterol (<130 mg/dl), and with evidence of generalized inflammation (CRP >2 mg/L) [Ridker 2008]. The CVD preventive benefits seen in JUPITER, surprisingly, were found to be proportional to reductions in CRP. This observation suggests that in the general population, anti-inflammatory pleiotropy may contribute significantly to the cardioprotective effects exerted by statins [Ridker 2009]. However, event reduction was also consistent with the known magnitude of expected effect from LDL lowering. Thus, although the JUPITER trial demonstrated a marked reduction in CVD events with statins among patients with relatively low LDL cholesterol but generalized inflammation, the lack of mechanistic assessment precluded determination of how this effect was achieved. In a study of the MESA cohort, a population similar to the JUPITER trial cohort, major adverse cardiovascular events correlated better with high

risk coronary atherosclerotic features than inflammatory markers such as hsCRP [Blaha 2011]. This data highlights the importance of performing assessments of plaque volume and morphology to characterize the mechanisms by which statins have their effect. Hence, the currently proposed study will determine, in the HIV-infected population, the effects of statins to prevent CVD events and, importantly, mechanisms through which statins may achieve this effect including a reduction of these high risk morphologic features.

2.2 Rationale

The proposed REPRIEVE (A5332) study is a multi-center, prospective randomized placebo controlled trial testing the effect of statin primary preventive therapy on CVD events in HIV-infected patients on ART without significantly increased traditional cardiovascular disease risk and no prior history of CVD. The trial described in this appendix is a mechanistic substudy embedded in the larger REPRIEVE (A5332) clinical events trial. In the Mechanistic Substudy of REPRIEVE (A5333s), coenrolled participants will undergo coronary computed tomography angiography (CCTA) for determination of atherosclerotic plaque morphology, as well as detailed profiling of lipid and inflammatory/immune parameters.

The Mechanistic Substudy of REPRIEVE (A5333s) will determine, among HIV-infected persons, potential statin effects to halt progression of non-calcified atherosclerotic plaque and to stabilize morphologic features of plaque vulnerability. Moreover, the study will identify biological factors mediating these changes – be it lipid parameters, such as LDL cholesterol, or markers of inflammation and immune activation. Finally, the study will demonstrate whether presence and morphology of subclinical atherosclerotic plaque at baseline relates to CVD events independently of traditional CVD risk factors and markers of HIV-specific immune activation.

Findings from the study will have implications for predicting CVD risk in HIV-infected individuals, for whom traditional risk prediction paradigms such as those used in the 2013 ACC/AHA guidelines may fall short (for failing to factor in the contributions of HIV-specific immune activation) [Stone 2013]. Moreover, findings from the mechanistic substudy will have implications for the development of targeted CVD preventive strategies in the HIV-infected population: If statins stabilize coronary atherosclerotic plaques and prevent CVD events primarily via LDL lowering, then further LDL lowering strategies may be indicated. On the other hand, if statins stabilize coronary atherosclerotic plaques and prevent CVD events primarily through immunomodulation, then complementary immune-suppressant therapies in HIV-infected patients – such as inhibitors of monocyte activation – will need to be further explored. Overall, the mechanistic sub-study will provide new, critical knowledge about the biology of atherosclerosis in HIV.

3.0 STUDY DESIGN

A5333s is an optional mechanistic substudy of REPRIEVE (A5332). Approximately 800 HIV-infected males and females who are enrolled in REPRIEVE (A5332) will coenroll in the Mechanistic Substudy of REPRIEVE (A5333s). To ensure treatment balance in treatment assignment in the Mechanistic Substudy of REPRIEVE (A5333s), randomization in REPRIEVE (A5332) will be stratified by anticipated substudy participation. Participants will be followed for 24 months in the Mechanistic Substudy of REPRIEVE (A5333s).

4.0 SELECTION AND ENROLLMENT OF SUBJECTS

4.1 Inclusion Criteria

4.1.1 Enrollment in REPRIEVE (A5332).

4.1.2 Willingness to complete procedures required for the study.

4.1.3 Signed informed consent.

4.1.4 Glomerular filtration rate (GFR) ≥60 mL/min/1.73m^2 or creatinine clearance (CrCl) ≥60 mL/min, as per REPRIEVE (A5332)

NOTE: Results of creatinine must be obtained and GFR or CrCl must be calculated within 14 days prior to CCTA. The GFR must be ≥60 mL/min/1.73m^2 or the CrCl must be ≥60 mL/min for the subject to proceed with CCTA both at entry and at month 24.

4.2 Exclusion Criteria

4.2.1 Known allergy to iodinated contrast agent.

4.2.2 Currently symptomatic asthma.

4.2.3 Allergy to beta blockers.

4.2.4 Contraindication to beta blockers (ie, taking daily asthma medications).

4.2.5 Positive pregnancy test within 24 hours prior to study entry.

NOTE: Female subjects of reproductive potential (defined as women who have not been post-menopausal for at least 24 consecutive months, ie, who have had menses within 24 months prior to study entry, and women who have not undergone surgical sterilization, specifically hysterectomy or bilateral oophorectomy) must have a negative serum or urine pregnancy test within 24 hours prior to CCTA by any US laboratory or clinic that has a CLIA certification or its equivalent, or is using a point-of-care (POC)/CLIA-waived test, or at any network-approved non-US laboratory or clinic that operates in accordance with Good Clinical Laboratory Practices and participates in appropriate external quality assurance programs.

NOTE: Subject reported history is considered acceptable documentation of hysterectomy, bilateral oophorectomy, and menopause. Women are considered menopausal if they have not had a menses for at least 12 months and have a FSH (follicle stimulating hormone) of greater than 40 IU/L or, if FSH testing is not available, they have had amenorrhea for 24 consecutive months.

4.2.6 Any condition that prohibits the individual from completing the CCTA.

4.2.7 Body mass index (BMI) ≥40 kg/m2.

NOTE: Refer to the MOPS for the link to the BMI calculator.

4.2.8 Cardiac arrhythmia at enrollment precluding CCTA; such as atrial fibrillation with heart rate >80 beats per minute or frequent ectopic beats. Please see MOPs for scanner-specific details.

4.3 Enrollment Procedures

The Mechanistic Substudy of REPRIEVE (A5333s) will be limited to select sites participating in REPRIEVE (A5332). Prior to implementation of the substudy, each site must have the protocol and the protocol consent form approved, as appropriate, by their local IRB/EC and any other applicable regulatory entity (RE).

Site inclusion criteria for the Mechanistic Substudy of REPRIEVE (A5333s) will include the following:

- Site participating in REPRIEVE (A5332).
- Site radiology or cardiology department/facility must conduct >1000 CT/per year.
- MD oversight/supervision of CT scans.
- CT scanner must be at least 2nd generation 64-slice.
- The facility must utilize level III readers.
- The radiology/cardiology facility must be capable of all of the following
  - prospective triggering/gating
  - using low KV
  - have capacity to premedicate subjects

Once a candidate for entry has been identified, details will be carefully discussed with the subject. The subject (or when necessary, the legal representative) will be asked to read and sign the approved Mechanistic Substudy of REPRIEVE (A5333s) consent form.

Enrollment into the Mechanistic Substudy of REPRIEVE (A5333s) will occur concurrently with enrollment and randomization into REPRIEVE (A5332), ie, completion of enrollment into A5333s should occur no later than one business day after enrollment into REPRIEVE (A5332), in recognition that lab results needed to determine eligibility into A5333s may not be available on the same day. Note this does not change the

requirement that CCTA for A5333s must be performed within 14 days after randomization.

NOTE: The Mechanistic Substudy of REPRIEVE (A5333s) closed to accrual on 02/06/18.

4.4 Subject Registration

Subjects who meet enrollment criteria will be registered to the substudy according to standard DMC procedures.

5.0 STUDY TREATMENT

No medications are provided by the Mechanistic Substudy of REPRIEVE (A5333s). Study treatment will be distributed and administered as per REPRIEVE (A5332). See section 5.0 of REPRIEVE (A5332) for study treatment requirements and concomitant medications.

6.0 EVALUATIONS

6.1 Schedule of Evaluations for the Mechanistic Substudy of REPRIEVE (A5333s)

Evaluation[1]	Entry	Month 4 ±21 days	Month 24 ± 28 days	Premature Study Disc. Evaluations
Calculate BMI[3]	X		X	
REAP and DASI Questionnaires			X	X
QOL assessment	X		X	X
Pregnancy Testing	X[4]		X[4]	X
Serum Creatinine	X[5]		X[5]	X
CBC with differential	X[5]		X[5]	X
CD4+/CD8+	X[5]		X[5]	X
Plasma HIV-1 RNA	X[5]		X[5]	X
Fasting Lipid Panel		X[6]		
Fasting Plasma/Serum for Biomarkers – Planned Analysis[7]	X	X	X	X
Cryopreserved PBMCs for Flow Cytometry – Planned Analysis	X		X	X
Whole blood for RNA	X		X	X
CCTA	X		X	X[2]

[1] Subjects in the mechanistic substudy will have all evaluations as per REPRIEVE (A5332) Schedule of Evaluations; additional testing and/or unique processing instructions specific to the mechanistic substudy are listed here in section 6.1 of the mechanistic substudy.

[2] Premature Study Discontinuation before month 12 should include all evaluations listed except CCTA. Premature Study Discontinuation at month 12 or later should include all evaluations *including* the second CCTA.

[3] For BMI calculation at entry, use height and weight obtained at screen from REPRIEVE (A5332). For month 24 BMI calculation, use height obtained at screen and weight obtained at month 24 from REPRIEVE (A5332).

[4] Pregnancy testing *must* be performed and reviewed prior to CCTA for mechanistic substudy participants at entry and month 24 to ensure subject eligibility.

[5] CD4+/CD8+, HIV viral load, CBC, and serum creatinine at entry and month 24 will be performed as part of substudy. Serum creatinine results *must* be drawn and GFR or creatinine clearance must be reviewed within 14 days prior to CCTA to ensure subject eligibility. If drawn more than 14 days before CCTA, repeat creatinine must be drawn. The CBC must be drawn on the same day as PBMC.

[6] Fasting lipid panel from month 4 will be performed centrally.

[7] HgbA1c, Lp-PLA2, sCD163, sCD14, MCP-1, IL-6, D-dimer (will not be collected at month 4), hsCRP, troponin, insulin, glucose. Related indices of insulin resistance such as HOMA-IR will be calculated.

6.2 Timing of the Evaluations

6.2.1 Entry

Registration to the Mechanistic Substudy of REPRIEVE (A5333s) will occur at the entry visit and is concurrent with enrollment to the main study, ie, completion of enrollment into A5333s should occur no later than one business day after enrollment into REPRIEVE (A5332), in recognition that lab results needed to determine eligibility into A5333s may not be available on the same day. Substudy entry evaluations (including CCTA evaluations) must be completed prior to the initiation of REPRIEVE (A5332) study medications. The baseline CCTA scan should be completed within 14 days after randomization.

6.2.2 Post-entry

Month 4 substudy evaluations must be performed ± 21 days; month 24 substudy evaluations must be performed ± 28 days.

6.2.3 Discontinuation Evaluations

Premature Discontinuation of REPRIEVE (A5332) Study Treatment
Subjects who prematurely discontinue REPRIEVE (A5332) study treatment may continue participation in the substudy with all evaluations performed as per section 6.0.

Premature Discontinuation of REPRIEVE (A5332) Study Participation
Subjects who prematurely discontinue participation in the REPRIEVE (A5332) study will be discontinued from the Mechanistic Substudy of REPRIEVE (A5333s).

Premature Discontinuation of the Mechanistic Substudy of REPRIEVE (A5333s) Participation
Subjects who discontinue participation in the Mechanistic Substudy of REPRIEVE (A5333s) may continue participation in REPRIEVE (A5332).

Subjects who prematurely discontinue participation in the Mechanistic Substudy (A5333s) should complete the Premature Discontinuation of Substudy visit. Premature Study Discontinuation before month 12 should include all evaluations listed except CCTA. Premature Study Discontinuation at month 12 or later should include all evaluations *including* the second CCTA.

Subjects who become pregnant while on the Mechanistic Substudy of REPRIEVE (A5333s) must immediately be discontinued from the substudy without any further evaluations performed.

6.3 Instructions for Evaluations

6.3.1 Clinical Assessments

Targeted Physical Exam
A targeted physical examination as described in REPRIEVE (A5332) will be performed as per section 6.1 and findings will be recorded on the REPRIEVE (A5332) CRFs. Blood pressure and pulse should also be taken. Blood pressure should be recorded on the REPRIEVE (A5332) CRFs at all substudy visits. Record any changes that occur after entry on the REPRIEVE (A5332) CRF.

Calculation for Body Mass Index will be performed prior to entry and month 24 CCTA. See MOPS for link to calculator.

Cardiovascular Risk Factor Assessment
Diet, using the Rapid Eating and Activity Assessment for Patients (REAP) questionnaire and functional capacity using the Duke Activity Status Index (DASI) questionnaire will be performed at the month 24 visit.

Quality of Life Assessment using the SF-36v2 questionnaire will be performed at entry and month 24.

6.3.2 Laboratory Evaluations

Pregnancy Testing
For women with reproductive potential: serum or urine beta-HCG (urine test must have a sensitivity of 15-25 mIU/mL). A negative pregnancy test result must be obtained before the CCTA.

Serum Creatinine
For entry and month 24, serum creatinine will be performed as part of the Mechanistic Substudy of REPRIEVE (A5333s). Serum creatinine must be drawn and GFR or CrCl must be calculated within 14 days prior to CCTA. The GFR must be ≥60 mL/min/1.73m^2 or CrCl must be ≥60 mL/min for the subject to proceed with CCTA both at entry and at month 24.

<u>CBC with Differential</u>
For entry and month 24, a CBC with differential will be performed as part of the Mechanistic Substudy of REPRIEVE (A5333s). The CBC must be drawn on the same day as the PBMC collection.

<u>CD4+/CD8+ T-cell Counts</u>
CD4+/CD8+ T-cell count and percentage assays at entry and month 24 must be performed as part of the substudy at a CLIA-certified or equivalent laboratory that is certified for protocol testing by the DAIDS Immunology Quality Assurance (IQA).

<u>Plasma HIV-1 RNA</u>
HIV-1 RNA must be performed at entry and month 24 by a laboratory that possesses a CLIA certification or equivalent.

<u>Fasting Lipids</u>
Serum lipid and lipoproteins (total cholesterol, HDL cholesterol, LDL cholesterol, non–HDL, triglycerides, particle size, lipid subfractions, oxidized LDL) are collected only for the Mechanistic Substudy of REPRIEVE (A5333s) at month 4. These samples will be batched and stored in the ACTG Specimen Repository for analyses by a central laboratory.

<u>Fasting Insulin</u>
Fasting insulin samples will be batched and stored in the ACTG specimen repository for analyses by a central laboratory. HOMA-IR will be calculated from fasting insulin determined from the Mechanistic Substudy of REPRIEVE (A5333s) and fasting glucose determined from REPRIEVE (A5332).

<u>HgbA1C</u>
Samples for HgbA1C will be batched and stored in the ACTG Specimen Repository for analyses by a central laboratory.

6.3.3 Immunologic and Biomarker Studies

<u>Fasting Plasma/Serum for Biomarkers – Planned Analysis</u>
The following primary biomarker assays will be performed using frozen samples: Lp-PLA2, sCD163, sCD14, MCP-1, IL-6, D-dimer, hsCRP, troponin. These samples will be batched and stored in the ACTG Specimen Repository for analyses by a central core laboratory. Refer to the Laboratory Processing Chart (LPC) for details. Additional biomarker and coagulation marker assays may be performed.

<u>PBMCs for Monocyte and T Lymphocyte Activation – Planned Analysis</u>
PBMCs for pre-specified flow cytometric analysis on monocytes and T lymphocytes will be batched and stored in the ACTG Specimen Repository for analyses by a central core laboratory. Refer to the LPC for details.

<u>Whole Blood RNA</u>

Whole blood will be obtained and processed for RNA. Samples will be batched and stored to enable the evaluation of ancillary studies to assess RNA changes.

6.3.4 CT Angiogram and Risks of Radiation Dose

Coronary computed tomography angiography (CCTA) is performed in accordance with best practice standards as delineated in the imaging guidelines of the Society of Cardiovascular Computed Tomography [Abbara 2009] by competent and appropriately credentialed physicians. This includes the optimization of the scan protocol to limit radiation dose. Specific protocols for each CT scanner, manufacturer, and model will be provided and performed in the MOPS. In general, both retrospective and prospective ECG gated protocols are permitted. The Core Lab will conduct an initial quality assurance screen to ensure correct imaging parameters (kVp, mAs, FOV, slice thickness, slice interval, reconstruction kernel). When a data set is received by the CT core lab, quality analysis (QA) will be performed and if accepted, a case acceptance notice will be sent to the site. If the data set does not pass QA, the site will be sent a query to resolve the issue. The CRA will determine if the data set requires resubmission or if the correspondence with the site can resolve the query. If the site does not respond to the query the CRA will follow the query escalation plan. When the query is resolved, the site will be sent a case acceptance notification and the data set will be moved to the reader work list. Prior to the follow up CCTA, the Core Lab will send the site a reminder including the baseline scan parameters with a request to perform the follow up as closely as possible resembling the baseline protocol. Refer to the Mechanistic Substudy of REPRIEVE (A5333s) MOPS for detailed instructions.

Risks of Radiation Exposure from CT: CT scanning results in a measurable radiation exposure. For most patients participation in this study will be associated with an estimated cumulative radiation exposure of approximately 11 mSv with a maximum dose of about 16 mSv over two years (median radiation exposure per exam: 5.6 mSv, range 3-8 mSv).

By comparison, radiation exposure from a single stress nuclear myocardial perfusion imaging, another test commonly used to detect heart disease, is about 14 mSv. The average annual radiation exposure from natural background sources in the US is between 2 and 3.6 mSv.

The radiation exposure from this study is only 10% of the maximally allowed annual radiation exposure to radiation workers such as radiology technologists, radiologists, or workers in nuclear plants (50 mSv per year). Overall, each CT scan adds a very small theoretical risk of less than 0.05% to the 7% lifetime risk of lung cancer for men and women and to the 12% lifetime risk of breast cancer for women.

Pregnant subjects must not have a CCTA scan.

7.0 TOXICITY MANAGEMENT

See section 7.0 in protocol REPRIEVE (A5332). Specific CCTA-related events should be handled as per standard treatment guidelines, eg, for acute contrast-related events.

8.0 CRITERIA FOR SUBSTUDY DISCONTINUATION

- Subjects who discontinue participation in REPRIEVE (A5332). Discontinuation of REPRIEVE (A5332) at A5332 study closure does not constitute premature discontinuation.
- The subject refuses further participation.
- The site investigator determines further participation would be detrimental to the subject's health or well-being.
- The subject becomes pregnant.
- The subject fails to complete the entry CCTA.
- Subject develops impaired renal function defined as confirmed CrCl <60 mL/min or GFR <60 mL/min/1.73m^2 at month 24 visit.
- The subject develops new onset clinically significant asthma.
- The subject develops new onset/previously undiagnosed allergy to IV contrast.
- Body mass index (BMI) is ≥40 kg/m2 at month 24 visit.

9.0 STATISTICAL CONSIDERATION

General Design Considerations
The overarching aim of the Mechanistic Substudy of REPRIEVE (A5333s) is to better understand the modulation of critical features of coronary plaque morphology with statin therapy, including the progression of non-calcified coronary atherosclerotic plaque (NCP) volume in HIV and the biological factors mediating these effects during statin treatment in HIV.

9.1 Endpoints

9.1.1 Primary Endpoints

9.1.1.1 Evidence of non-calcified coronary atherosclerotic plaque (NCP) at study entry and 2 years.

9.1.1.2 Volume of non-calcified coronary atherosclerotic plaque (NCP) at study entry and change in NCP over 2 years (expressed as absolute change and as a percentage of baseline).

9.1.1.3 Progression of NCP where progression will be defined as follows:

a) Among subjects with evidence of NCP at entry, any progression/increase in NCP volume
b) Among subjects without evidence of NCP at entry, incident NCP.

9.1.2 Secondary and Supportive Endpoints

9.1.2.1 Number of segments with NCP

9.1.2.2 Presence and number of each of the following high-risk plaque features

- Low Hounsfield Unit attenuation by CT assessment
- Positive remodeling

9.1.2.3 Levels at study entry, 4 months, and 2 years, and changes from study entry to 4 months and 2 years in the following biomarkers

- Markers of HIV-1 disease: CD4 cell count, HIV-1 RNA level (entry to 2 years only)
- Soluble markers of monocyte activation: sCD163, sCD14, MCP-1
- Various monocyte populations including %CD14+CD16+ monocytes (entry to 2 years only)
- Markers of T-cell activation and exhaustion (entry to 2 years only)
- Markers of inflammation: Lp-PLA2, hsCRP, IL6, troponin
- Markers of coagulation: D-Dimer and tissue factor (excluding D-Dimer at month 4; D-Dimer will be available at entry and month 24 only)

9.1.2.4 Fasting lipid fractions (Total, non-HDL, and HDL cholesterol) and LDL:HDL ratio at study entry, month 4 and 2 years.

9.1.2.5 Fasting insulin, HgbA1c, and HOMA-IR at study entry, month 4, and 2 years (excluding HgbA1c at month 4; HgbA1c will be available at entry and month 24 only).

9.1.2.6 Time to the first major cardiovascular events as defined in section 9.2.1 of REPRIEVE (A5332).

9.2 Sample Size

The target sample size for the Mechanistic Substudy of REPRIEVE (A5333s) is 800 participants that will be approximately equally distributed between the study arms.

This sample size was determined to have high power to detect clinically relevant differences between the two study groups both with respect to plaque progression (among those with plaque at study entry) and rates of incident plaque (among those plaque-free at entry). Specifically, the total sample size of 800 subjects will provide 90% power to detect a 6% difference between the study groups in the percent change in NCP volume over 2 years among those with plaque at entry and 90% power to detect 13 percentage point difference in the probability of plaque development over 2 years. These effect sizes translate to a combined estimated 14 percentage point difference in the probability of NCP progression over two years and are based on the following assumptions:

- 50% of study participants will have evidence of NCP at study entry [Lo 2010]
- A SD of 20% for the percent change over 2 years among participants with evidence of plaque at entry
- An annual rate of incident plaque development of 12% among participants without plaque at entry
- 15% of participants entering the substudy will not be evaluable for study entry or 2 year NCP volume.

Together, the effects of statins acting in these two groups (those with and without evidence of NCP at study entry) will provide for a 7% lower prevalence of NCP after 2-years of statin treatment. Expectations for NCP prevalence and progression both as a whole and according to whether NCP was present at study entry are illustrated in the table below. These estimates are based on a simulation that further assumed the following:

- Average volume NCP 250mm^3 (SD=200) among participants with NCP at entry.
- Average volume NCP 40mm^3 (SD=20) at 2 years among participants without NCP at entry without statin treatment.
- Average volume NCP 20mm^3 (SD=20) at 2 years among participants without NCP at entry with statin treatment.
- NCP distributions were also assumed to follow a gamma distribution with size and shape parameters determine to provide the desire mean and standard deviations.

Table 9.2-1: Cells constituting the primary targeted group comparisons are shown in bold.

		At study entry			At two 2 years						
		NCP (%)	NCP volume (mm3)		NCP (%)	NCP volume (mm3)		Change in NCP volume (mm3)		Percent change in NCP volume (%)[2]	
			Mean (SD)	[P5, P95]		Mean (SD)	[P5, P95]	Mean (SD)	[P5, P95]	Mean (SD)	[P5, P95]
Control	Overall[1]	50%	121 (186)	[0, 509]	59%	140 (210)	[0, 573]	19 (51)	[-21, 103]	17% (33%)	[-10%, 100%]
	No plaque at entry		-	-	21%	9 (19)	[0, 51]	9 (19)	[0, 51]	21% (41%)	[0%, 100%]
	No plaque at 2 years					-	-	-	-	-	-
	Plaque at 2 years					40 (20)	[17, 79]	40 (20)	[17, 79]	100% (0%)	[100%, 100%]
	Plaque at entry		251 (198)	[26, 637]		281 (231)	[28, 730]	30 (68)	[-38, 153]	12% (20%)	[-14%, 49%]
Statin	Overall[1]	50%	120 (185)	[0, 507]	52%	127 (198)	[0, 536]	7 (45)	[-42, 76]	7% (24%)	[-17%, 60%]
	No plaque at entry		-	-	8%	2 (8)	[0, 14]	2 (8)	[0, 14]	8% (27%)	[0%, 100%]
	No plaque at 2 years					-	-	-	-	-	-
	Plaque at 2 years					23 (19)	[2,60]	23 (19)	[2,60]	100% (0%)	[100%, 100%]
	Plaque at entry		250 (197)	[26, 633]		262 (216)	[26, 682]	12 (64)	[-66, 123]	5% (20%)	[-21%, 43%]

[1] In estimation of overall means, participants without evidence of NCP are assigned a value of 0 for volume and change.

[2] In estimation of percentage change, participants without evidence of NCP at study entry with NCP at 2 years are assigned a value of 100%.

These desired statin effects on NCP volume are similar to those seen in intravascular ultrasound (IVUS) studies [Nissen 2006; Nicholls 2005]. Although no data exist to directly inform the clinical relevance of these differences, randomized comparisons of high vs. low dose statin therapy among non HIV-infected patients have demonstrated favorable effects on atherosclerotic plaque of a similar magnitude as well as on adverse cardiac events, among non HIV patients [Nissen 2006]. Further, data from the "Coronary

CT Angiography Evaluation for Clinical Outcomes (CONFIRM) registry suggest that the presence of non-obstructive CAD is predictive major adverse cardiac events independent of traditional risk factors, degree of stenosis and coronary artery calcification (HR 2-5) [Hulton 2013]. The proposed mechanistic study will further add to this body of evidence investigating, for the first time in HIV-infected population, the association of NCP (both the presence and magnitude) as well as changes in volume relate to events. For this exploratory analysis, a larger sample size will be beneficial, in order to accrue CVD events to relate to plaque morphology.

9.3 Randomization/Registration

Participants will be enrolled into the Mechanistic Substudy of REPRIEVE (A5333s) via the enrollment system at the same time as they are randomized into REPRIEVE (A5332). To ensure balanced treatment allocation in the mechanistic study, REPRIEVE (A5332) randomization will be stratified by planned mechanistic substudy participation.

9.4 Monitoring

Ongoing monitoring of accumulating data for the Mechanistic Substudy of REPRIEVE (A5333s) by the study team (pooled by treatment group) will occur for study conduct and data completeness. Since scans will be read and biomarkers tested in batch at the end of the study, data completeness focus will be for tracking of scan and specimen completeness.

Such issues will be monitored by the jointly appointed NIAID/NHLBI DSMB at the time of DSMB review of REPRIEVE (A5332).

9.5 Analysis Plan

9.5.1 General analysis considerations

The presence and extent of coronary artery disease (CAD) in this population will be described including non-obstructive or obstructive CAD, volume of NCP and calcified plaque, and Agatston score, as well as the prevalence of high risk plaque features such as positive remodeling and low CT attenuation, and levels of inflammatory, and immunomodulatory biomarkers. 95% confidence intervals will be provided for CAD characteristic for the mechanistic study subgroup as whole and by randomization.

All treatment group comparisons will be performed ITT using a 5% type error. Unless otherwise noted, analyses will be performed by subgroups defined according to the presence of NCP at study entry. Since CCTA will not be available at the time of randomization, balance by treatment arm cannot be guaranteed within these subgroups, but given the large sample size and stratification according to sex and HIV-1 disease severity relative balance between the group sample sizes is expected.

9.5.2 Analysis plan

Statin effects on coronary plaque morphology: Among participants with plaque at entry, descriptive statistics for the change and percentage change in NCP volume over 2 years will be provided by treatment group with group comparisons made with stratified t-test. Among those without NCP at entry, the prevalence of incident NCP over 2 years will be compared with stratified chi-squared test. To assess the mechanistic study population as a whole patients will be classified as progressors (any progression/increase in NCP volume OR incident NCP) or non-progressors (no progression in NCP volume OR no incident NCP); the probability of progression over two years will be compared by treatment group using a stratified chi-squared test.

The statin effect on high risk plaque features, including low HU attenuation and positive remodeling, will be assessed by comparing differences in the 2-year prevalence of high risk plaque morphology features between treatment groups using chi-squared (or Fisher's exact) tests as appropriate; these analyses will be performed overall and by subgroups defined by the presence of NCP at study entry. Exploratory analysis will be performed for additional high risk plaque features that have been described in CCTA and IVUS studies including the Napkin Ring Sign, Minimal luminal area, plaque burden, and segments with NCP. The analytic approach will be similar as described above.

Statin effects on blood biomarkers: Statin effects on the distributions of blood biomarkers belonging to distinct pathways (ie, monocyte activation, generalized inflammation, and coagulation) will be assessed via treatment group comparisons of these respective markers via t-tests; modification of statin effect on these markers by HIV-1 and traditional risk factors (including sex, age, screening CD4, duration of suppressive ART, and presence of NCP at study entry). Since the hypothesized mechanism is that sustained high levels of immune activation and inflammation precede and contribute to progression of NCP volume and high risk plaque features, these analyses will relate short term changes in these biomarkers (over 4 months) to longer term changes in NCP volume and morphology after two years.

LDL and blood biomarkers as mediators for plaque progression: In the event that both statin effects on NCP progression and biomarker changes are apparent, the association between changes in LDL and these biomarkers and NCP progression will be examined using graphical techniques and normal errors and logistic regression (for the subpopulations with and without NCP at entry respectively. A mediating effect of these biological factors will be evaluated by examination of changes in the estimated statin effect on NCP upon adjustment for these biological factors. Those biomarkers with the strongest mediating effect on plaque progression will be measured in the entire REPRIEVE (A5332) cohort to determine their association with MACE.

10.0 DATA COLLECTION AND MONITORING AND ADVERSE EVENT REPORTING

Serious adverse events (SAEs) that occur as part of participation in the Mechanistic Substudy (A5333s) are reported as expedited adverse events (EAEs) through REPRIEVE (A5332). These SAEs are reported on a CRF for the Mechanistic Substudy (A5333s); they are not reported on the CRF for REPRIEVE (A5332). There are additional non-serious AEs (ie, non-serious contrast reactions) that may occur as part of the Mechanistic Substudy (A5333s) that will also be recorded on the CRF. Please see the Mechanistic Substudy (A5333s) MOPS for more details regarding AE reporting for A5333s.

11.0 HUMAN SUBJECTS

IRB/EC review, subject confidentiality, and study discontinuation procedures will be the same as in REPRIEVE (A5332). Subjects must sign a separate informed consent form for the Mechanistic Substudy of REPRIEVE (A5333s). Risks, including potential risks of CCTA, and protection against risk are described in the accompanying sample informed consent form.

12.0 PUBLICATION OF RESEARCH FINDINGS

Publication of the results of the Mechanistic Substudy of REPRIEVE (A5333s) will be governed by NIH policies.

13.0 BIOHAZARD CONTAINMENT

Precautions and procedures will be as in REPRIEVE (A5332).

14.0 REFERENCES

Abbara S, et al. SCCT guidelines for performance of coronary computed tomographic angiography: a report of the Society of Cardiovascular Computed Tomography Guidelines Committee. J Cardiovasc Comput Tomogr 2009;3(3):190-204.

Aberg JA, et al. Pitavastatin versus pravastatin in adults with HIV-1 infection and dyslipidaemia (INTREPID): 12 week and 52 week results of a phase 4, multicentre, randomised, double-blind, superiority trial. Lancet HIV 2017;4(7):e284-e294.

Blaha MJ, et al. Associations between C-reactive protein, coronary artery calcium, and cardiovascular events: implications for the JUPITER population from MESA, a population-based cohort study. Lancet 2011;378(9792):684-92.

Bu DX, et al. Statin-induced Kruppel-like factor 2 expression in human and mouse T cells reduces inflammatory and pathogenic responses. J Clin Invest 2010;120(6):1961-70.

Burdo TH, et al. Soluble CD163, a novel marker of activated macrophages, is elevated and associated with noncalcified coronary plaque in HIV-infected patients. J Infect Dis 2011;204(8):1227-36.

Burgstahler C, et al. Influence of a lipid-lowering therapy on calcified and noncalcified coronary plaques monitored by multislice detector computed tomography: results of the New Age II Pilot Study. Invest Radiol 2007;42(3):189-95.

De Wit S, et al. Downregulation of CD38 activation markers by atorvastatin in HIV patients with undetectable viral load. AIDS 2011;25(10):1332-3.

Downs JR, et al. Primary prevention of acute coronary events with lovastatin in men and women with average cholesterol levels: results of AFCAPS/TexCAPS. Air Force/Texas Coronary Atherosclerosis Prevention Study. JAMA 1998;279(20):1615-22.

Eckard AR, et al. Effects of 24 weeks of statin therapy on systemic and vascular inflammation in HIV-infected subjects receiving antiretroviral therapy. J Infect Dis 2014;209(8):1156-64.

Freiberg MS, et al. HIV infection and the risk of acute myocardial infarction. JAMA Intern Med 2013;173(8):614-22.

Funderburg NT, et al. Rosuvastatin reduces vascular inflammation and T cell and monocyte activation in HIV-infected subjects on antiretroviral therapy. J Acquir Immune Defic Syndr 2015;68(4):396-404.

Funderburg NT, et al. Rosuvastatin treatment reduces markers of monocyte activation in HIV-infected subjects on antiretroviral therapy. Clin Infect Dis 2014;58(4):588-95.

Fujino M, et al. Pitavastatin-induced downregulation of CCR2 and CCR5 in monocytes is associated with the arrest of cell-cycle in S phase. Atherosclerosis 2006;187(2):301-8.

Ganesan A, et al. High dose atorvastatin decreases cellular markers of immune activation without affecting HIV-1 RNA levels: results of a double-blind randomized placebo controlled clinical trial. J Infect Dis 2011;203(6):756-64.

Giorgi JV, et al. Shorter survival in advanced human immunodeficiency virus type 1 infection is more closely associated with T lymphocyte activation than with plasma virus burden or virus chemokine coreceptor usage. J Infect Dis 1999;179(4):859-70.

Guo H, et al. Rosuvastatin inhibits MMP-2 expression and limits the progression of atherosclerosis in LDLR-deficient mice. Arch Med Res 2009;40(5):345-51.

Han KH, et al. HMG-CoA reductase inhibition reduces monocyte CC chemokine receptor 2 expression and monocyte chemoattractant protein-1-mediated monocyte recruitment in vivo. Circulation 2005;111(11):1439-47.

Han J, et al. Pitavastatin downregulates expression of the macrophage type B scavenger receptor, CD36. Circulation 2004;109(6):790-6.

Hiro T, et al. Effect of intensive statin therapy on regression of coronary atherosclerosis in patients with acute coronary syndrome: a multicenter randomized trial evaluated by volumetric intravascular ultrasound using pitavastatin versus atorvastatin (JAPAN-ACS [Japan assessment of pitavastatin and atorvastatin in acute coronary syndrome] study). J Am Coll Cardiol 2009;54(4):293-302.

Hou ZH, et al. Prognostic value of coronary CT angiography and calcium score for major adverse cardiac events in outpatients. JACC Cardiovasc Imaging 2012;5(10):990-9.

Hsue PY, et al. Immunologic basis of cardiovascular disease in HIV-infected adults. J Infect Dis 2012;205 Suppl 3:S375-82.

Hulten E, et al. Usefulness of coronary computed tomography angiography to predict mortality and myocardial infarction among Caucasian, African and East Asian ethnicities (from the CONFIRM [Coronary CT Angiography Evaluation for Clinical Outcomes: An International Multicenter] Registry). Am J Cardiol 2013;111(4):479-85.

Hunt PW, et al. Impact of CD8+ T-cell activation on CD4+ T-cell recovery and mortality in HIV-infected Ugandans initiating antiretroviral therapy. AIDS 2011;25(17):2123-31.

Inoue K, et al. Serial coronary CT angiography-verified changes in plaque characteristics as an end point: evaluation of effect of statin intervention. JACC Cardiovasc Imaging 2010;3(7):691-8.

Kaplan RC, et al. T cell activation and senescence predict subclinical carotid artery disease in HIV-infected women. J Infect Dis 2011;203(4):452-63.

Kelesidis T, et al. Biomarkers of microbial translocation and macrophage activation: association with progression of subclinical atherosclerosis in HIV-1 infection. J Infect Dis 2012;206(10):1558-67.

Kitagawa T, et al. Characterization of noncalcified coronary plaques and identification of culprit lesions in patients with acute coronary syndrome by 64-slice computed tomography. JACC Cardiovasc Imaging 2009;2(2):153-60.

Kodama K, et al. Stabilization and regression of coronary plaques treated with pitavastatin proven by angioscopy and intravascular ultrasound--the TOGETHAR trial. Circ J 2010;74(9):1922-8.

Kwak B, et al. Statins as a newly recognized type of immunomodulator. Nat Med 2000;6(12):1399-402.

Liu Z, et al. Elevated CD38 antigen expression on CD8+ T cells is a stronger marker for the risk of chronic HIV disease progression to AIDS and death in the Multicenter AIDS Cohort Study than CD4+ cell count, soluble immune activation markers, or combinations of HLA-DR and CD38 expression. J Acquir Immune Defic Syndr Hum Retrovirol 1997;16(2):83-92.

Lo J, et al. Increased epicardial adipose tissue volume in HIV-infected men and relationships to body composition and metabolic parameters. AIDS 2010;24(13):2127-30.

Merlini E, et al. T-cell phenotypes, apoptosis and inflammation in HIV+ patients on virologically effective cART with early atherosclerosis. PLoS One 2012;7(9):e46073.

Mira E, et al. Statins induce regulatory T cell recruitment via a CCL1 dependent pathway. J Immunol 2008;181(5):3524-34.

Montecucco F, et al. Statins inhibit C-reactive protein-induced chemokine secretion, ICAM-1 upregulation and chemotaxis in adherent human monocytes. Rheumatology (Oxford) 2009;48(3):233-42.

Motoyama S, et al. Computed tomographic angiography characteristics of atherosclerotic plaques subsequently resulting in acute coronary syndrome. J Am Coll Cardiol 2009;54(1):49-57.

Mulhaupt F, et al. Statins (HMG-CoA reductase inhibitors) reduce CD40 expression in human vascular cells. Cardiovasc Res 2003;59(3):755-66.

Nakamura T, et al. Rapid stabilization of vulnerable carotid plaque within 1 month of pitavastatin treatment in patients with acute coronary syndrome. J Cardiovasc Pharmacol 2008;51(4):365-71.

Nicholls SJ, et al. Effect of atorvastatin (80 mg/day) versus pravastatin (40 mg/day) on arterial remodeling at coronary branch points (from the REVERSAL study). Am J Cardiol 2005;96(12):1636-9.

Nissen SE, et al. ASTEROID Investigators. Effect of very high-intensity statin therapy on regression of coronary atherosclerosis: the ASTEROID trial. JAMA 2006;295(13):1556-65.

Ridker PM, et al. JUPITER Study Group. Rosuvastatin to prevent vascular events in men and women with elevated C-reactive protein. N Engl J Med 2008;359(21):2195-207.

Ridker PM, et al. Reduction in C-reactive protein and LDL cholesterol and cardiovascular event rates after initiation of rosuvastatin: a prospective study of the JUPITER trial. Lancet 2009;373(9670):1175-82.

Romano M, et al. Inhibition of monocyte chemotactic protein-1 synthesis by statins. Lab Invest 2000;80(7):1095-100.

Sacks FM, et al. The effect of pravastatin on coronary events after myocardial infarction in patients with average cholesterol levels. Cholesterol and Recurrent Events Trial investigators. N Engl J Med 1996;335(14):1001-9.

Sandler NG, et al. INSIGHT SMART Study Group. Plasma levels of soluble CD14 independently predict mortality in HIV infection. J Infect Dis 2011;203(6):780-90.

Shepherd J. The West of Scotland Coronary Prevention Study: a trial of cholesterol reduction in Scottish men. Am J Cardiol 1995;76(9):113C-117C.

Shimojima, M, et al. Rapid changes in plaque composition and morphology after intensive lipid lowering therapy: study with serial coronary CT angiography. Am J Cardiovasc Dis 2012;2(2):84-8.

Silverberg MJ, et al. Response to newly prescribed lipid-lowering therapy in patients with and without HIV infection. Ann Intern Med 2009;150(5):301-13.

Singh P, et al. Influence of statins on MHC class I expression. Ann N Y Acad Sci 2009;1173:746-51.

Stone NJ, et al. 2013 ACC/AHA guidelines on the treatment of blood cholesterol to reduce atherosclerotic cardiovascular risk in adults: A Report of the American College of Cardiology/American Heart Association Task Force on Practice Guidelines. J Am Coll Cardiol 2014;63(25 Pt B):2889-934.

Subramanian S, et al. Arterial inflammation in patients with HIV. JAMA 2012;308(4):379-86.

Tawakol A, et al. Intensification of statin therapy results in a rapid reduction in atherosclerotic inflammation: results of a multi-center fluorodeoxyglucose-positron emission tomography/computed tomography feasibility study. J Am Coll Cardiol 2013;62(10):909-17.

Triant VA, et al. Increased acute myocardial infarction rates and cardiovascular risk factors among patients with human immunodeficiency virus disease. J Clin Endocrinol Metab 2007;92(7):2506-12.

Veillard NR, et al. Simvastatin modulates chemokine and chemokine receptor expression by geranylgeranyl isoprenoid pathway in human endothelial cells and macrophages. Atherosclerosis 2006;188(1):51-8.

Waehre T, et al. Hydroxymethylglutaryl coenzyme a reductase inhibitors down-regulate chemokines and chemokine receptors in patients with coronary artery disease. J Am Coll Cardiol 2003;41(9):1460-7.

Zanni MV, et al. HIV-specific immune dysregulation and atherosclerosis. Curr HIV/AIDS Rep 2012;9(3):200-5.

Zanni MV, et al. Increased coronary atherosclerotic plaque vulnerability by coronary computed tomography angiography in HIV-infected men. AIDS 2013;27(8):1263-72.

Zheng C, et al. Statins suppress apolipoprotein CIII-induced vascular endothelial cell activation and monocyte adhesion. Eur Heart J 2013;34(8):615-24.

Zineh I, et al. Modulatory effects of atorvastatin on endothelial cell-derived chemokines, cytokines, and angiogenic factors. Pharmacotherapy 2006;26(3):333-40.

APPENDIX III: SAMPLE INFORMED CONSENT

For The Mechanistic Substudy of REPRIEVE (A5333s), **FINAL Version 6.0**, **16May2022**

Effects of Pitavastatin on Coronary Artery Disease and Inflammatory Biomarkers: Mechanistic Substudy of REPRIEVE

SHORT TITLE FOR THE STUDY: The REPRIEVE Mechanistic Substudy

INTRODUCTION

You are being asked to take part in this research substudy because you will be taking pitavastatin or the placebo for pitavastatin for REPRIEVE (A5332). This study is sponsored by the National Institutes of Health (NIH). The doctor in charge of this substudy at this site is: (insert name of Principal Investigator). Before you decide if you want to be a part of this substudy, we want you to know about the substudy.

This is a consent form. It gives you information about this substudy. The substudy staff will talk with you about this information. You are free to ask questions about this substudy at any time. If you agree to take part in this substudy, you will be asked to sign this consent form. You will get a copy to keep.

WHY IS THIS SUBSTUDY BEING DONE?

The purpose of this substudy is to learn about the effects of pitavastatin on the vessels that supply your heart with blood “coronary arteries” and the atherosclerotic plaque within the wall of these vessels (known as “hardening of the arteries”), as well as inflammatory biomarkers (blood tests that indicate the body’s immune system is active) among people with HIV (PWH).

HOW MANY PEOPLE WILL BE IN THIS SUBSTUDY?

About 800 people will take part in this study.

WHAT DO I HAVE TO DO IF I AM IN THIS SUBSTUDY?

If you agree to be in this substudy and sign this consent form, you will be asked to come in for 3 visits. Each visit will last about 2-3 hours and will occur at the same time as your main study visits whenever possible. The visits are at entry (when you join the substudy), month 4, and month 24.

Before all visits for the substudy you should not eat or drink anything, including food, beverages, candy, or gum for 8 hours before your visit. You are encouraged to drink water before your

visits. If you are not fasting, we will ask you to return to have your blood drawn within 21 days of the study visit.

The procedures described below will be done in addition to your participation in the REPRIEVE (A5332) study.

Explanation of study procedures
You will be asked to fill out a questionnaire about your quality of life at entry and month 24.

Study staff will ask you questions about your diet and physical activity at month 24.

For women capable of having children, a pregnancy test will be done immediately before the CT of your heart. This test is required as part of your participation in this study. You will be told the results of the pregnancy test. You must notify the research staff if you are pregnant, think you may be pregnant, or if you are trying to become pregnant. If do become pregnant while on the substudy, you will be taken off the substudy and will not have any more substudy tests.

At entry and month 24 we will check your kidney function, complete blood count (CBC), CD4 T-cell count (how many infection fighting cells are in your blood), and HIV viral load (how much HIV is in your blood). The test to check your kidney function is required as part of your participation in this research study. Approximately 3 teaspoons of blood will be collected at each the entry and month 24 visits for these tests.

You will be told the results of these tests.

You will have about 1-4 tablespoons of blood drawn in addition to the blood drawn for REPRIEVE (A5332) at the entry, month 4 and month 24 visits. This blood will be collected and stored for tests that will be done later on in the study or after the study is over. These tests will measure various substances in your blood related to cholesterol (fat in your blood), blood sugar, metabolic tests (how your body processes food), inflammation, and immune function (how your body reacts to infection). You do not need to agree to store this blood to join the study and you may change your mind about storing your blood at any time. You will not be told of the results of the research done on your blood.

Do you agree to let us store your samples for these tests?

________ YES ________ NO _______Initials

Approximately 1 teaspoon of blood collected will be used to look at genes that may affect your risk for cardiovascular disease. Genetic testing is a laboratory test that looks at differences in people's genes. Your body, like all living things, is made up of cells, and cells contain deoxyribonucleic acid, also known as "DNA." DNA is like a string of information put together in a certain order. Parts of the string make up "genes." For the substudy, we will do a test to look at your RNA. RNA is made from DNA and is short for ribonucleic acid. RNA is a genetic material that has a major role in making proteins. Proteins are the building blocks of your body, cells, and organs. Genes contain instructions on how to make your body work and fight disease. The testing in this study will focus on certain RNA's that are known to be related to cardiovascular

disease and effects of statins. New RNA's of interest may be identified in the future and may also be looked at. You do not need to agree to store this blood to join the study and you may change your mind about storing your blood at any time. You will not be told of the results of the research done on your blood.

Your body's genetic makeup is unique to you, so there is a risk with genetic research that even with all the security measures in place, someone using your samples or genetic information may still find out which information is yours. However, this risk today is very small, but it may increase with time since science and technology are developing rapidly.

In the event that your genetic information becomes linked to your name, the US federal law called the Genetic Information Nondiscrimination Act (GINA) helps protect you. This law prohibits health insurance companies, group health plans, and most employers from denying services based on your genetic information. However, GINA does not protect against discrimination by companies that sell life insurance, disability insurance, or long-term care insurance.

We would like to use some of the blood we collect to look at your genes (RNA). Do you agree to this genotyping?

________ YES ________ NO   _______Initials

If at a later date you change your mind and want your samples destroyed, contact the research staff. There are two ways to withdraw your permission. You could allow researchers to remove all your personal identifiers from your samples, so that they are not linked to you anymore. These samples will then become anonymous. Or, you can ask researchers to destroy your samples, so that they cannot be used for future research. However, in either case, researchers will not be able to destroy samples or information from research that is already underway.

You will have a computed tomography (CT) scan ("Cat" scan) of your heart at entry and month 24. A CT scan is a special kind of x-ray that takes pictures of the inside of the body using a small amount of radiation. A small amount of dye (intravenous contrast) will be injected into your arm during the CT scan to better see the vessels that supply your heart with blood. If your heart rate is more than 65 beats per minute, we may inject a drug called a beta-blocker into your arm via the intravenous line. A beta-blocker is used to slow down your heart rate. A low heart rate is needed in order to make the best pictures of your heart and coronary arteries. In addition, a drug called nitroglycerin will be given to you by mouth in order to obtain better images of the blood vessels of the heart. We will also check your heart rhythm with an electrocardiogram (ECG). To do this, wires with sticky pads attached will be placed on your chest before the scan.

You will be asked to lie quietly while your body is moved inside a large machine and the x-ray is taken. The CT scan takes about 15 minutes. For women, a pregnancy test will be performed prior to CT scanning. Pregnant women will not be allowed to undergo CT scanning. For all patients, a blood test for kidney function will be performed before the CT scan and patients with abnormal kidney function will not undergo CT scanning.
Because the test results are being used for research only, the results created by this study will not become part of your hospital record unless we discover an unexpected medical problem that

must be communicated to the study doctors or your primary care physician. If you are found to have a critical blockage of the vessels supplying your heart with blood or another important non-cardiac abnormality that may affect your health, we will provide the results of the CT to your study doctor. The cost of any additional testing will not be covered by the study.

Other Information
You may withdraw from this substudy at any time and still remain on the main study. If you decide to withdraw from the substudy early (before month 24) or if you decide to withdraw from the main study you will be asked to return to the clinic to have the procedures listed in the table below.

Appendix III, Table 1: Discontinuation Procedures

Procedure	Stopping the study
Fasting Blood	X
Blood Collected	X
Pregnancy Testing	X
Computed Tomography of your Heart (CT scan)	X
Quality-of-Life Assessment	X

If you leave the study before month 12, you will not have a CT scan at the discontinuation visit.

HOW LONG WILL I BE IN THIS SUBSTUDY?

You will be in this substudy for about 2 years.

WHY WOULD THE DOCTOR TAKE ME OFF THIS STUDY EARLY?

The study doctor may need to take you off the study early without your permission if:

- the doctor thinks it is in your best interest
- the study is cancelled
- you are not able to attend the study visits as required by the study
- you are unable to complete the computed tomography of your heart at the entry visit
- you become pregnant
- you have to stop participating in the main study
- your kidney function becomes abnormal during your study participation
- you develop asthma during your study participation
- you develop an allergy to the contrast dye during your study participation
- your body mass index (a measure of body fat based on your height and weight) is greater or equal to 40.

WHAT ARE THE RISKS OF THE SUBSTUDY?

The scanning on CT machines will not cause any physical discomfort other than from having to lie still on the table for the duration of the test.

Risks of Radiation Dose from CT
You will have two CTs, one at entry and one at month 24. CT results in a measurable radiation dose. For most people the dose from each CT will be approximately twice the amount you get from natural background radiation (sun and earth) each year. Your dose may be higher or lower based on your size, your heartbeat, and the CT scanner. To put the total dose from the two CTs in further context, it is similar to the radiation dose from a cardiac imaging stress test (another test commonly used to detect heart disease) and much less (approximately 10%) of the maximum allowed exposure for radiation workers such as medical technologists, radiologists, or nuclear plant technicians.

The lifetime risk of lung cancer for men and women is 7%. The lifetime risk of breast cancer in women is 12%. Each CT scan adds a very small theoretical risk of 0.05%.

Radiation Dose
Cardiac CT scanning results in a measurable radiation dose. In order to minimize risk for participants in this study we have implemented the following measures:
- Exclude subjects <40 years of age
- Exclude pregnant and breast feeding women
- Application of cardiac CT protocols to minimize radiation dose
- Review and approval of these CT protocols by the IRB/EC

Risks of Intravenous Contrast Dye
You will receive intravenous contrast dye as part of the CT scan and there is a small risk (2 out of 1,000) of an allergic reaction. More than 90% of such adverse reactions are very mild and allergic-like (itching, *rash*) and can be effectively treated with available drugs (ie, antihistamine). Severe reactions occur in 2 out of 10,000 and one death occurred in approximately 60,000 contrast dye injections.

Contrast-Induced Nephropathy (CIN)
CIN is a kidney injury caused by contrast and is usually reversible. CIN almost always occurs in people who already have abnormal kidney function. To be enrolled in this study you must have normal kidney function, and for this reason CIN is very unlikely (less than 5 out of 1,000).

Risks of IV Needle Placement
- Hemorrhage (bruise at the injection site)
- Infection (catheter related infection) at the injection site (very rare)
- Leaking of contrast agent outside of the vein at the place where the IV is inserted.
- Minor discomfort
- Bleeding

- Infection
- Bruising

Risks of Beta-blockers and Nitroglycerin

Beta-blockers and nitroglycerin are used by millions of Americans and are generally considered safe. These drugs are routinely administered prior to cardiac CT to improve the quality and interpretability of the study.

The risk of beta-blockers includes slow heart rate (bradycardia), low blood pressure (hypotension), and wheezing (bronchospasm). Allergic reactions to beta-blockers are rare. Persons with asthma treated with inhalers should not receive beta-blockers. Study staff will assess this and other reasons for you not to have beta-blockers with you prior to the CT.

The side-effects and risks of nitroglycerin are generally mild and of short duration and include low blood pressure (hypotension), high heart rate and abnormal rhythm (tachyarrhythmia), headache, lightheadedness, and visual disturbance. Persons who take erectile dysfunction medications such as Viagra, Cialis, or Levitra (sildenafil, tadalafil, or vardenafil) will need to stop these drugs at least 5 days prior to receiving nitroglycerin on the day of the cardiac CT scan. Nitroglycerin should not be given to persons with a low blood pressure. Study staff will assess this and other reasons for you not to have nitroglycerin with you prior to the CT.

Other Risks/ Additional Risks of CT Scans

- Discomfort
- Claustrophobia

Risks of Drawing Blood

Having your blood drawn may cause discomfort, bleeding, and bruising where the blood is drawn. Occasionally, there is swelling in the area where the needle enters the body and there is a small risk of infection. There is also a risk of lightheadedness, fainting, and blood clots.

Risks of Fasting

Some people find fasting to be bothersome. It may make some individuals feel anxious, irritable, or hungry. Patients who are required to take their morning medications with food should wait until after the visit has been completed to take their medications.

Genetic Testing

The results of your genetic tests are for research purposes only and no individual results will be given back to you. The results of the genetics studies will never become a part of your medical record. We will protect your confidentiality to the fullest extent. Blood samples for genetic studies will be identified in a way in order to maintain your confidentiality.
Research study results will not be given to your family members, insurance companies, employers, or third parties without your written permission and approval of the Institutional Review Board at _________.

Additional Risks
The CT scan of your heart is being done to answer research questions, not to examine you medically. This scan is not a substitute for one your doctor would order. If the radiologist thinks that there may be an abnormality in your scan, we will contact you and will help you get medical follow-up for the problem. If you have a primary care doctor, we can contact your doctor, with your permission, and help him or her get the right follow-up for you. It is possible that you could be unnecessarily worried if a problem were suspected, but not actually found.

ARE THERE BENEFITS TO TAKING PART IN THIS SUBSTUDY?

If you take part in this substudy, there may be a direct benefit to you, but no guarantee can be made. It is also possible that you may receive no benefit from being in this study. Information learned from this study may help others who have HIV.

WHAT OTHER CHOICES DO I HAVE BESIDES THIS STUDY?

Instead of being in this study you have the choice of:
- participating in REPRIEVE (A5332) only
- not participating

Please talk to your doctor about these and other choices available to you. Your doctor will explain the risks and benefits of these choices.

WHAT ABOUT CONFIDENTIALITY?

We will do everything we can to protect your privacy. In addition to the efforts of the study staff to help keep your personal information private, we have gotten a Certificate of Confidentiality from the U.S. Federal Government. This certificate means that researchers cannot be forced to tell people who are not connected with this study, such as the court system, about your participation. Also, any publication of this study will not use your name or identify you personally.

People who may review your records include the AIDS Clinical Trials Group (ACTG), OHRP, (insert name of site) IRB/EC, government agencies such as the National Institutes of Health (NIH) and Food and Drug Administration (FDA), other local, US, and international regulatory entities, study staff, study monitors, the drug company supporting this study, and its designee. Having a Certificate of Confidentiality does not prevent you from releasing information about yourself and your participation in the study.

Even with the Certificate of Confidentiality, if the study staff learns of possible child abuse and/or neglect or a risk of harm to yourself or others, we will be required to tell the proper authorities.

A description of this clinical trial will be available on www.ClinicalTrials.gov. This web site will not include information that can identify you. At most, the web site will include a summary of the results. You can search this Web site at any time.

WHAT IF WE CAN NO LONGER REACH YOU DURING YOUR STUDY PARTICIPATION?

In the event you cannot be reached after multiple attempts to contact you, study staff may try to contact you through alternate phone numbers of family, friends, case manager, or acquaintances obtained at screening and updated at each visit. If you are unable to be reached through the alternate contacts we will attempt to obtain information about you from other sources such as family members, other designated contacts, or clinic records. The purpose of obtaining this information is to determine if you have died and the cause of death since last contact.

WHAT ARE THE COSTS TO ME?

Taking part in this substudy may lead to added costs to you and your insurance company. In some cases it is possible that your insurance company will not pay for these costs because you are taking part in a research study.

WILL I RECEIVE ANY PAYMENT?

You will be paid ________ per visit for participation in the substudy. *(The team recommends compensation to participants of $25 at the entry and the month 24 visits. Sites will be reimbursed for the expense.)*

WHAT HAPPENS IF I AM INJURED?

If you are injured as a result of being in this study, you will be given immediate treatment for your injuries. The cost for this treatment will be charged to you or your insurance company. There is no program for compensation either through this institution or the National Institutes of Health. You will not be giving up any of your legal rights by signing this consent form.

WHAT ARE MY RIGHTS AS A RESEARCH PARTICIPANT?

Taking part in this study is completely voluntary. You may choose not to take part in this study or leave this study at any time. Your decision will not have any impact on your participation in other studies conducted by NIH and will not result in any penalty or loss of benefits to which you are otherwise entitled.
We will tell you about new information from this or other studies that may affect your health, welfare, or willingness to stay in this study. If you want the results of the study, let the study staff know.

WHAT DO I DO IF I HAVE QUESTIONS OR PROBLEMS?

For questions about this study or a research-related injury, contact:

- name of the investigator or other study staff
- telephone number of above

For questions about your rights as a research participant, contact:

- name or title of person on the Institutional Review Board (IRB/EC) or other organization appropriate for the site
- telephone number of above

OTHER

All other information that is contained in the main study REPRIEVE (A5332) consent that you signed also applies to this substudy consent. A copy of the signed main study consent will be provided for you as a reference at the time you consent to participate in the substudy.

SIGNATURE PAGE

If you have read this consent form (or had it explained to you), all your questions have been answered and you agree to take part in this study, please sign your name below.

_______________________ Subject's Name (print)	_______________________ Subject's Signature and Date
_______________________ Participant's Legal Representative (print) (As appropriate)	_______________________ Legal Representative's Signature and Date
_______________________ Study Staff Conducting Consent Discussion (print)	_______________________ Study Staff's Signature and Date
_______________________ Witness's Name (print) (As appropriate)	_______________________ Witness's Signature and Date

APPENDIX IV: REPRIEVE OBJECTIVES TO DECIPHER SEX-SPECIFIC MECHANISMS OF CVD RISK AND RISK REDUCTION

BACKGROUND

HIV-infected individuals face a markedly increased risk of cardiovascular disease (CVD), even when viremia is suppressed by combined antiretroviral therapy (cART) [Freiberg 2013; Triant 2007]. Mechanisms underlying HIV-associated CVD risk are incompletely understood [Zanni 2014] and specific guidelines on cardioprotective care for this population are not available [Stone 2014]. Characterizing and reducing CVD risk among HIV-infected women represents a particular challenge. HIV-infected women are less likely than HIV-infected men to be offered preventive cardiac care in clinical practice [Willig 2008]. However, data suggest women with HIV are just as likely as their male counterparts to incur an MI [Triant 2007]. Indeed, a large-scale US epidemiologic study shows that among HIV-infected women, unadjusted rates of MI modestly *exceed* those among HIV-infected men [Triant 2007]. Moreover, the same study reveals a significantly higher adjusted relative risk of MI in HIV infected-women versus uninfected females (2.89) as compared with HIV-infected men versus uninfected males (1.4) [Triant 2007]. The proposed objectives aim to identify sex-specific mechanisms of CVD risk and risk reduction in relation to adjudicated clinical CVD events in HIV. Immune activation is increased in HIV-infected women (versus HIV-infected men) [Fitch 2013] and especially among those HIV-infected women who have undergone menopause [Looby 2015]. Moreover, among women with HIV, reduced ovarian reserve has been shown to relate to subclinical atherosclerotic plaque even after controlling for traditional CVD risk factors, including age [Looby 2015]. Building on these observations, our aims interrogate immune and hormonal pathways hypothesized to contribute to CVD risk in HIV-infected women across the reproductive aging spectrum.

OBJECTIVES

<u>OBJECTIVE 1: To assess among HIV-infected individuals ages 40-75 sex-based differences in immune activation and statin-induced immunomodulation in relation to clinical CVD events</u>.
In order to achieve this objective, we will add to the REPRIEVE trial blood sample collection on female and male participants and sample processing for immune activation markers relevant to atherogenesis in HIV (beginning with monocyte activation markers including sCD14, and then exploring other immune activation markers). <u>HYPOTHESES</u>: Baseline relationships: A) Levels of immune activation markers will be higher in HIV-infected women versus men. B) High-level immune activation will relate to CVD events in HIV, and this association will be stronger in women versus men. Statin effects: C) Statins will decrease levels of immune activation markers to a greater extent in HIV-infected women versus men. D) CVD risk-reduction will be mediated in part through statin-induced immunomodulation, more so in HIV-infected women versus men.

<u>OBJECTIVE 2: To characterize among HIV-infected women ages 40-75 how menopause status and ovarian reserve relate to immune activation, statin-induced immunomodulation, and clinical CVD events</u>. In order to achieve this objective, we will add to the REPRIEVE trial blood sample collection on female participants and sample processing for hormones including anti-Müllerian hormone (AMH) and estradiol (E2). Coupling hormonal data with menstrual history, we will

categorize women as pre-menopausal and post-menopausal. We will also define the subset of pre-menopausal women with reduced ovarian reserve. HYPOTHESES: Baseline relationships: A) Post-menopausal HIV-infected women will have higher levels of immune activation markers versus pre-menopausal HIV-infected women. B) Among both pre- and post-menopausal HIV-infected women, reduced ovarian reserve (undetectable AMH) will relate to risk of first CVD event. Statin effects: C) Statins will decrease levels of immune activation markers to a greater extent in post-menopausal HIV-infected women versus pre-menopausal HIV-infected women. D) CVD risk-reduction will be mediated in part through statin-induced immunomodulation, more so in post-menopausal (vs. pre-menopausal) HIV-infected women.

SIGNIFICANCE

Overall, we will assess whether immune activation contributes uniquely to CVD risk among HIV-infected women across the reproductive aging spectrum and how statins may reduce CVD risk through effects on this pathway. Answers to these critical questions will influence the development of CVD prediction and prevention strategies tailored to the aging female HIV-infected population worldwide. Maximizing our power to elucidate sex-specific CVD mechanisms in HIV, we will also design, implement, and evaluate the effectiveness of an evidence-based education/awareness recruitment campaign to enhance female enrollment in the REPRIEVE trial. Identification of innovative strategies to recruit women to an interventional clinical trial could have far-reaching practical implications for future trial-based, sex-specific research across disciplines.

INTEGRATION OF OBJECTIVES INTO A5332

In order to address the objectives above, the REPRIEVE trial protocol has been amended to permit for additional collection of fasting blood stored for assessment of biomarkers at entry, 1 year, and at end of study. The end of study blood draw will facilitate future assessment of whether observed changes in immune markers at 1 year are sustained. Please see A5332 protocol section 6.1, Schedule of Evaluations.

STATISTICAL CONSIDERATIONS

Overview Considerations

Overall sample size assumptions: The projected sample size is 5446 persons, or approximately 80% of the total REPRIEVE study sample.

General analysis plans: As comparison groups to address the objectives listed below (eg, groups classified by sex for Objective 1 and by menopause/hormone status for Objective 2) will not have been assigned by randomization, the distributions of baseline characteristics will be compared between groups to help inform the list of characteristics to be included in covariate .adjusted models specified below. Baseline covariates anticipated to differ by sex (eg, race, hypertension, smoking, substance use, lipids, BMI) [Hatleberg 2014] will be carefully explored

and considered, particularly when they are hypothesized to be prognostic for the outcome. Where treatment effect is included in the analysis plan below (eg, statin effect), this variable will use intent-to-treat formulation.

General monitoring considerations: Safety of human participants participating in the objectives described will be monitored by the REPRIEVE DSMB as part of the REPRIEVE trial study monitoring plan.

Outcome Definition, sample size justification, and analysis plans by objective/hypothesis

Objective 1: To assess among HIV-infected individuals ages 40-75 sex-based differences in immune activation and statin-induced immunomodulation in relation to clinical CVD events.

Hypothesis A) *Levels of immune activation markers will be higher in HIV-infected women versus men.* The outcome being tested in this sub-objective is baseline (pre-treatment) immune activation, as measured by the levels of each of select immune activation markers such as sCD14. A non-parametric alternative to t-test (eg, Mann-Whitney test) will compare the pre-treatment distribution of each biomarker between groups assigned by sex. If 22% female enrollment is achieved, then the comparison group sample sizes to address this hypothesis will be 1198 women versus 4248 men (with no adjustments for losses as outcome assessed at baseline). Using these sample sizes and the standard deviations of select biomarkers [Fitch 2013], and adjusting the significance level to 0.0167 by Bonferroni to account for the biomarkers being tested, there will be 90% power to detect sex differences of 214 ng/mL for sCD14. Covariate adjusted models performed as part of the analysis of this hypothesis will use multivariable regression (linear, with log transformation if indicated based on biomarker distribution).

Hypothesis B) *High-level immune activation will relate to CVD events in HIV, and this association will be stronger in women versus men.* The primary outcome being tested in this sub-objective is the composite clinical outcome of MACE occurring at any time during follow-up. The secondary outcome being tested is the time from randomization to first MACE occurring any time during follow-up. High-level immune activation with respect to each tested biomarker will be classified by the observed highest quartile of the pre-treatment distributions. Fisher's exact test will be used for testing the main effect of immune activation. Taking the sample size of 5446 from the overall sample size assumptions above, there is an expectation of 4084 in the low immune activation group and 1362 (highest quartile) in the high immune activation group. Assuming ~6% of the study sample will experience a MACE event (from control arm incidence estimate of 15/1000 person-years and statin effect hazard ratio of 0.70; see REPRIEVE protocol section 9.0 for more details), with sample sizes as above and a Bonferroni-adjusted significance level of 0.0167, there will be 90% statistical power to detect 5% MACE rate in the low immune activation group compared with 7.8% MACE rate in the high immune activation group (ie, absolute difference of 2.8% pts in MACE rate between groups). Assessing whether the association of baseline immune activation and MACE will be higher in women versus men requires testing for an interaction effect between sex and immune activation (high vs. low) for the outcome of MACE. To assess power for interaction effect, methods described in [VanderWeele 2012] will be used under the following assumptions: 4.9% of men with low immune activation will have a qualifying MACE outcome; the main effect odds ratios (OR) for

sex and immune activation are each 2.1 (eg, 4.9% MACE in one group compared with 9.8% in the other); a multiplicative interaction OR of 1.33; association between sex and high/low immune activation of 1.33; and 22% female enrollment. Based on these assumptions and using a significance level adjusted to 0.0167, the statistical power for additive interaction effect (eg, as excess in relative risk attributed to interaction) is estimated as 80%. Testing for interaction effects and covariate adjusted models will use multivariable logistic regression (for the primary outcome) and Cox proportional hazards regression (for the secondary, time- to-event formulation outcome).

Hypothesis C) *Statins will decrease levels of immune activation markers to a greater extent in HIV-infected women versus men.* The outcomes being tested in this sub-objective are the changes from pre-treatment to 1 year of follow-up of the immune activation markers tested. For this hypothesis, statistical interactions of sex and treatment effects will be tested using linear (log transformed as indicated) models, which can also incorporate covariate adjustment. With estimates of 5% per year loss to follow-up, the effective sample size for this sub-aim is 5174 persons. For the biomarker sCD14, standard deviation of the outcome measure for this sub-aim is estimated assuming standard deviation of 1-year measurements same as baseline and intra-person correlation of 0.65. Further, assuming under additivity a 125 ng/mL effect of sex on sCD14 differences at 1 year and the same effect of statins on sCD14 differences at 1 year, there will be 80% power to detect an additive interaction effect of 220 ng/mL.

Hypothesis D) *CVD risk-reduction will be mediated in part through statin-induced immunomodulation, more so in HIV-infected women versus men*. For this sub-objective, structural equation modeling (SEM) will be employed to investigate the pathways and mechanisms leading to this clinical outcome while incorporating other prognostic factors. Specifically, the SEM model will apply an analysis of covariance structure incorporating multiple biomarker data into a single model for CVD clinical outcome. Furthermore, the model will test the covariates and directionality of effects with CVD outcome, and include temporal ordering. For this sub-objective, some of these pathways include the following: direct effect of sex on CVD, direct effect of sex on immunomodulation, direct effect of immunomodulation on CVD, sex effect on CVD through immunomodulation, direct effect of statin on CVD and on immunomodulation, and sex effect on CVD through statin effect.

Objective 2: To characterize among HIV-infected women ages 40-75 how menopause status and ovarian reserve relate to immune activation, statin-induced immunomodulation, and clinical CVD events.

The comparison groups will be formulated by combining hormonal data with data on menstrual history in order to categorize women as pre-menopausal and post-menopausal. Comparison groups will also define the subset of pre-menopausal women with reduced ovarian reserve.

Hypothesis A) *Post-menopausal HIV-infected women will have higher levels of immune activation markers versus pre-menopausal HIV-infected women.* As in Objective 1A, the outcome being tested in this sub-objective is baseline (pre-treatment) immune activation, as measured by levels of certain biomarkers such as sCD14. A non-parametric alternative to t-test (eg, Mann-Whitney test) will compare the pre-treatment distribution of each biomarker within women between groups assigned by menopause status. As per Objective 1A, 22% female

enrollment is assumed. Further assuming a 1:1 ratio between female comparison groups, sample sizes for the pre-menopausal and post-menopausal groups will be 599 each. Using these sample sizes, standard deviations for biomarker as in Objective 1A, and Bonferroni-adjusting the significance level to 0.0167, there will be 90% power to detect differences between the pre- and post-menopausal groups of 378 ng/mL for sCD14. Alternatively, if the ratio between female comparison groups is 1:2, sample sizes for the pre-menopausal and post-menopausal group will be 399 and 799, respectively. Under this contingency, the minimal detectable differences between female comparison groups for 90% power are only slightly higher at 401 ng/mL.

Hypothesis B) *Among both pre- and post-menopausal HIV-infected women, reduced ovarian reserve (undetectable AMH) will relate to risk of first CVD event.* As in Objective 1B: The primary outcome being tested in this sub-objective is the same composite clinical outcome of MACE occurring any time during follow-up. The secondary outcome being tested is the time from randomization to first MACE. Assuming 22% female enrollment in REPRIEVE and that a negligible proportion of the pre-menopausal women have reduced ovarian reserve such that the ratio of women with adequate versus reduced ovarian reserve is 1:1, then sample sizes for each group will be 599. As per Objective 1B, it is assumed that the overall rate of MACE among women will be 7%. Applying this event rate with the sample sizes above and using Fisher's exact test for analysis, there will be 90% power (at 5% significance) to show a difference of 5% points between groups (namely, 4.5% MACE in one group and 9.4% MACE in the other.) Alternatively, if a significant proportion of the pre-menopausal women have reduced ovarian reserve such that the ratio of women with adequate versus reduced ovarian reserve is 1:4, then sample sizes will be 299 for the adequate ovarian reserve group and 898 for the reduced ovarian reserve group. In this scenario, there will be 90% power to show a difference of 5.7% between groups. Covariate adjusted models for this hypothesis will use multivariable logistic regression for the primary outcome formulation, and Cox proportional hazards modeling for the time-to-initial event formulation.

Hypothesis C) *Statins will decrease levels of immune activation markers to a greater extent in post-menopausal HIV-infected women versus pre-menopausal HIV-infected women.* The outcomes being tested in this sub-objective are the changes from pre-treatment to 1 year of follow-up of each of the immune activation markers tested parallel to Objective 1C. Interactions by menopause status and treatment will be tested using linear (log transformed as indicated) models, which can also incorporate covariate adjustment. Assuming a 22% female enrollment and a 5% per year loss to follow up, the effective sample size for this analysis is 1138. Assuming a 1:1 ratio of female comparison groups, the sample sizes will be 569 for the pre- and post-menopausal groups. Using analysis assumptions from Objective 1C, and under additivity assuming a 375 ng/mL effect of menopause on sCD14 differences at 1 year as well as a similar effect of statins on sCD14 differences at 1 year, there will be 80% power to detect an additive interaction of menopause and statin of 390 ng/mL.

Hypothesis D) *CVD risk-reduction will be mediated in part through statin-induced immunomodulation, more so in post-menopausal (vs. pre-menopausal) HIV-infected women.* Parallel to Objective 1D, the SEM model will be used to incorporate all biomarker data into a single model for CVD clinical outcome, and then to test the covariate pathways, including directionality and temporal ordering. For this sub-objective, some of these pathways to be tested

include the following: direct effect of menopause status on CVD, direct effect of menopause status on immunomodulation, direct effect of immunomodulation on CVD, menopause effect on CVD through immunomodulation, direct effect of statin on CVD and on immunomodulation, and menopause effect on CVD through statin effect.

REFERENCES

Fitch KV, Srinivasa S, Abbara S, et al. Noncalcified coronary atherosclerotic plaque and immune activation in HIV-infected women. J Infect Dis 2013;208(11):1737-46.

Freiberg MS, Chang CCH, Kuller LH, et al. HIV infection and the risk of acute myocardial infarction. JAMA Intern Med 2013;173(8):614-22.

Hatleberg CI, Ryom L, El-Sadr W, et al. Gender differences in HIV-positive persons in use of cardiovascular disease-related interventions: D:A:D study. J Int AIDS Soc 2014;17(4 Suppl 3):19516.

Looby SE, Fitch KV, Srinivasa S, et al. Reduced ovarian reserve relates to monocyte activation and subclinical coronary atherosclerotic plaque in women with HIV. AIDS 2015 Published ahead of print. doi: 10.1097/QAD.0000000000000902.

Stone NJ, Robinson JG, Lichtenstein AH, et al.; American College of Cardiology/American Heart Association Task Force on Practice Guidelines. 2013 ACC/AHA guideline on the treatment of blood cholesterol to reduce atherosclerotic cardiovascular risk in adults: a report of the American College of Cardiology/American Heart Association Task Force on Practice Guidelines. J Am Coll Cardiol 2014;63(25 Pt B):2889-2934.

Triant VA, Hang Lee, Hadigan C, Grinspoon SK, et al. Increased acute myocardial infarction rates and cardiovascular risk factors among patients with human immunodeficiency virus disease. J Clin Endocrinol Metab 2007;92(7):2506-12.

VanderWeele TJ. Sample size and power calculations for additive interactions. Epidemiol Method 2012;1(1):159-88.

Willig JH, Jackson DA, Westfall AO, et al. Clinical inertia in the management of low-density lipoprotein abnormalities in an HIV clinic. Clin Infect Dis 2008;46(8):1315-18.

Zanni MV, Schouten J, Grinspoon SK, Reiss P. Risk of coronary heart disease in patients with HIV infection. Nat Rev Cardiol 2014;11(12):728-41.

APPENDIX V: REPRIEVE ANCILLARY STUDY: EFFECT OF PITAVASTATIN ON KIDNEY FUNCTION IN HIV-INFECTED PERSONS

BACKGROUND

With advances in HIV therapy, the incidence of HIV-associated nephropathy and subsequent end-stage renal disease (ESRD) have declined substantially [Lucas 2014; Lucas 2008; SMART 2006]. Still, 30-40% of HIV-infected persons are estimated to have Stage 1 or greater chronic kidney disease (CKD) [Gupta 2004; Overton 2012; Szczech 2004], and HIV infection remains an established risk factor for CKD, even after adjusting for traditional risk factors [Althoff 2015; Coresh 2003]. Black race, diabetes mellitus (DM) and hypertension (HTN) are common among HIV-infected persons and associated with an increased risk for CKD [Gathogo 2014; Jotwani 2012]. In fact, the effect of HIV may be additive to traditional risk factors like diabetes [Medapalli 2012].

This increased risk of CKD among HIV-infected individuals is likely mediated by inflammation [Mallipattu 2013]. HIV infection induces a paradoxical state of both immune suppression (low CD4+ T-cell count with increased risk of opportunistic pathogens) and immune activation [Deeks 2011]. The depletion of CD4+ T cells in the gut-associated lymphoid tissue facilitates excess microbial translocation and ensuing activation of both the innate and adaptive arms of the immune system [Brenchley 2006]. Such chronic immune activation translates into a well-characterized exhausted T-cell phenotype and a persistent pro-inflammatory milieu with elevated inflammatory cytokines and cellular immune activation markers, as well as markers of oxidative stress [Dolan 2005; Ross 2008]. Identifying adjunctive therapies that target these inflammatory pathways has the potential to prevent CKD in HIV-infected individuals.

In certain risk groups in the general population (DM, HTN, and pre-existing CVD), post-hoc analyses of randomized controlled trials (RCTs) have suggested that HMG-CoA reductase inhibitors (ie, statins) have a beneficial effect on the kidney. For example, in an RCT of atorvastatin for secondary prevention of strokes, eGFR improved with atorvastatin but not placebo, independent of baseline kidney function [Amarenco 2014]. These findings corroborate an analysis from the Collaborative Atorvastatin Diabetes Study, in which persons with diabetes and normal LDL-cholesterol were randomized to atorvastatin or placebo. Participants assigned to statin therapy demonstrated statistically significant improvement in eGFR that was most pronounced among persons with pre-existing microalbuminuria [Colhoun 2009]. These beneficial effects of statins have been shown to be most robust in persons with higher inflammatory biomarkers at baseline [Tonelli 2005]. These data suggest that the kidney protective effect of statins may be mediated through anti-inflammatory pathways, highly relevant in the setting of HIV infection. A recent Cochrane review of statins administered in the setting of CKD concluded that statins have uncertain effects on progressive CKD and additional prospective trials are warranted, particularly among persons with diabetes and other high-risk populations [Palmer 2014]. These post-hoc analyses from HIV negative individuals indicate that equipoise exists as to whether statins protect kidney function, particularly in populations with increased systemic inflammation, such as seen with HIV infection. Given the established association of CKD with CVD endpoints [Chronic Kidney Disease Prognosis Consortium 2010], interventions that prevent eGFR decline and albuminuria may also have important implications for CVD and all-cause mortality.

OBJECTIVES

OBJECTIVE 1: To evaluate the effects of pitavastatin on clinically relevant parameters of kidney function among HIV-infected individuals on ART.

OBJECTIVE 1A: To determine whether pitavastatin is associated with a lower incidence of clinically relevant eGFR decline in HIV-infected persons.

OBJECTIVE 1B: To determine whether pitavastatin is associated with a lower prevalence of albuminuria.

HYPOTHESES: 1. We hypothesize that randomization to pitavastatin versus placebo will be associated with a lower incidence of clinically relevant eGFR decline, defined as ≥30% decline from baseline, a Food and Drug Administration/National Kidney Foundation (FDA/NKA) recommended surrogate endpoint for CKD [Inker 2014]. 2. We hypothesize that randomization to pitavastatin versus placebo will be associated with a lower prevalence of albuminuria, defined as urine albumin/creatinine ratio (ACR) >30 mg/g.

OBJECTIVE 2: To assess whether the effect of pitavastatin 4 mg/daily on eGFR and albuminuria is stronger in high-risk groups defined by older age, black race, hypertension, lower CD4 cell counts, or the use of tenofovir disoproxil fumarate (TDF)-containing regimens.

HYPOTHESIS: We hypothesize the kidney protective effect of pitavastatin will be stronger in high-risk groups.

OBJECTIVE 3: To determine whether the effect of pitavastatin on kidney function is mediated through anti-inflammatory effects.

HYPOTHESIS: We hypothesize that effects of pitavastatin on eGFR and albuminuria will be mediated by systemic and vascular inflammation and oxidative stress, as measured by circulating levels of key inflammatory biomarkers.

SIGNIFICANCE

By adding collection of urine and blood longitudinally in at least 2500 participants, this ancillary study will definitively assess whether pitavastatin therapy will prevent significant decline in kidney function and the development of albuminuria (ACR >30 mg/g). This sample will further provide the opportunity to evaluate the effect of pitavastatin in sub-groups of HIV-infected participants for whom the risk of CKD has been demonstrated to be higher, including persons of older age, black race, hypertension, lower CD4 T-cell counts, and on tenofovir-containing regimens. Finally, the mechanisms of action will be assessed to determine whether the protective effect of pitavastatin is mediated through pathways involving inflammation and oxidative stress. Ultimately, the findings of this study will provide longitudinal data on the epidemiology of CKD in the setting of HIV infection, provide mechanistic insights into whether statins prevent CKD by modulating inflammation and oxidative stress, and potentially change guidelines for the prevention of CKD in this at-risk population.

INTEGRATION OF OBJECTIVES INTO A5332

This ancillary study requires only modest changes to the procedures of the parent study as it will leverage the existing study infrastructure, data collection, and trial visits to allow for additional stored blood and urine collection for a longitudinal assessment of relevant kidney function parameters. As CKD and albuminuria are independent risk factors for ASCVD, this ancillary study will provide additional insight into the primary focus of the parent trial. Furthermore, only modest changes to the parent protocol and informed consent are required to facilitate these evaluations, including collection of stored blood and urine at entry and years 1, 2, and 4 of study follow up.

STATISTICAL CONSIDERATIONS

Overview Considerations

Overall sample size assumptions: This ancillary study is projected to include at least 2500 persons, or approximately 38% of the total REPRIEVE study sample. The ancillary study will only recruit participants from the ACTG study sites.

General analysis plans: A baseline assessment of kidney function will be performed to assess baseline measures of kidney function and to assess clinical parameters associated with lower eGFR and albuminuria at baseline. For Objective 1, an intention-to-treat approach will be used based on randomization to pitavastatin or placebo for pitavastatin in the parent trial. For Objective 2, the effect of pitavastatin will be assessed in high-risk subgroups (HTN, age, race, tenofovir use) as outlined below. Objective 3 will utilize a case-control design to determine relevant mechanistic pathways through which the effects of pitavastatin are mediated.

General monitoring considerations: Safety of human participants participating in the kidney ancillary study will be monitored by the REPRIEVE DSMB as part of the REPRIEVE trial study monitoring plan.

OBJECTIVE 1: To evaluate the effects of pitavastatin on clinically relevant parameters of kidney function among HIV-infected individuals on ART.

Endpoints

For the primary analysis, we will use accepted measures of decreased eGFR. We have selected 30% decline in eGFR or ESRD for our primary outcome based on recommendations from a joint NKF-FDA workshop, which concluded that this should be considered as an alternative CKD endpoint for clinical trials [Inker 2014]. Specifically, in a pooled cohort of 850,096 individuals with eGFR ≥60 mL/min/1.73 m^2, a decline in eGFR ≥30% over 3 years of follow-up was associated with a hazard ratio of 7.0 for developing ESRD over the following decade. In secondary analysis, we will consider the more stringent endpoints of 40% decline in eGFR and development of eGFR <60 mL/min/1.73m^2. We will use the CKD Epidemiology Collaboration (CKD-EPI) creatinine equation to estimate GFR, as it has been demonstrated to be more accurate than other creatinine-based formulas in both the general population and in

HIV-infected cohorts [Matsushita 2010; Levey 2015; Inker 2012; Gagneux-Brunon 2012; Bhasin 2013]. The CKD-EPI creatinine equation had statistically significantly higher accuracy and less bias than the cystatin C equation in HIV-infected persons [Inker 2012].

Albuminuria will be quantified by ACR calculated as urinary albumin in mg/dL divided by urinary creatinine in g/dL. Albuminuria is an easily assessed marker of kidney damage, which may precede eGFR decline, and is strongly linked to adverse clinical outcomes [Gerstein 2001]. Even at levels of albuminuria >30 mg/g, persons are at increased risk for CVD events and all-cause mortality [Gerstein 2001; Wyatt 2010; Wyatt 2011]. We will use the ACR as a measure of albuminuria, as it is widely available in clinical practice and recommended by the IDSA Clinical Practice Guideline for CKD in HIV-Infected persons [Lucas 2014]. The ACR from a random urine sample is simple to obtain, correlates well with data from 24-hour urine collections, but is more practical and less prone to error than a 24-hour collection [Eknoyan 2003].

Data Analysis Plan

Aim 1A: Determine whether pitavastatin 4mg daily is associated with a lower incidence of ≥30% eGFR decline in HIV-infected persons. We will use an intention-to-treat approach for Aim 1. Descriptive statistics for participant characteristics will be presented by randomization assignment. The percent change in eGFR from baseline at study visits at 1, 2, and 4 years post-randomization, separately, will be calculated as follow-up eGFR minus baseline eGFR divided by baseline eGFR. For our primary analysis, the CKD endpoint will be a decline in eGFR ≥30% from the baseline visit or incident ESRD. The percentage of participants randomized to pitavastatin and, separately, placebo having a decline in eGFR ≥30% will be calculated at each follow-up visit. Additionally, the cumulative proportion of participants with ≥30% decline in eGFR throughout follow-up will be calculated by randomization arm and differences in these percentages of participants reaching the endpoint during follow-up will be compared using a chi-square test. We will assess these differences using logistic regression adjusted for key risk covariates as a confirmatory analysis. Additionally, we will evaluate time-to-event using interval censored regression models. Interval censored regression models are proposed as we do not know the exact date an individual reaches the CKD endpoint - only that it occurred between two visits.

Sample Size

We will enroll a sample size of at least 2500 participants. This sample size will provide adequate statistical power to achieve our aims. We anticipate an annual event rate of 2.5% based on previous reported results [Lucas 2007; Lucas 2008]. Therefore, over 4 years, we anticipate 10% of participants will meet our primary outcome of eGFR decline. As we do not know the exact incidence of eGFR decline, we provide the minimal detectable difference based on this event rate and also for 8% and 15% incidence of eGFR decline (ie, annual event rates of 2% and 3.75%, respectively). Based on sample size of 2500 participants (50% randomized to pitavastatin and 50% placebo), a two-tailed alpha error of 5% and assuming 5% of participants will be lost during follow-up, we will have 90% statistical power to detect clinically relevant differences in the cumulative incidence of eGFR decline across randomization arms. For example, if 10% of participants randomized to placebo develop our primary endpoint over the 4-year follow-up period, we will have 90% power to detect an absolute reduction in incidence of 3.6% associated with pitavastatin (10% in placebo vs. 6.4% in pitavastatin arm; **Table 1**). Given that albuminuria is anticipated to be a more frequent endpoint than ≥30% eGFR decline, we will

have 90% power to detect clinically relevant differences in the percentage of participants developing this outcome measure. Previous RCTs of pitavastatin in individuals with diabetes and persons with hyperlipidemia demonstrated a 52% and 67% reduction in measures of albuminuria, respectively [Nakamura 2005; Yagi 2011]. Although we expect to see at least a 50% reduction in albuminuria in the pitavastatin group (from 15% to 7.5%) and a modest increase in prevalence of albuminuria in the placebo group [Szczech 2010], we will be powered to detect smaller differences.

Table 1. Minimal detectable difference in incidence of CKD endpoint in REPRIEVE participants randomized to pitavastatin vs placebo.

	Proportion of control population developing CKD		
Aim 1A eGFR decline	8%	10%	15%
90% statistical power	3.2%	3.6%	4.3%
80% statistical power	2.6%	3.2%	3.5%
	Proportion of control population with albuminuria		
Aim 1B % with albuminuria	15%	20%	25%
90% statistical power	4.3%	4.9%	5.4%
80% statistical power	3.8%	4.3%	4.7%

OBJECTIVE 2: Assess whether the effect of pitavastatin on CKD is stronger in high-risk groups defined by older age, black race, hypertension, lower CD4 cell counts, or the use of TDF-containing regimens. We will divide the cohort based on the median age to evaluate differences between the groups above and below the median age. Similar dichotomous approaches will be taken for black race, hypertension (systolic blood pressure ≥140 mmHg, diastolic blood pressure ≥90 mmHg or antihypertensive medication use), and TDF-containing regimens. Within each sub-group studied, participant characteristics will be calculated by randomization assignment (pitavastatin or placebo). The percentage of participants developing ≥30% eGFR decline and separately, with ACR >30 mg/g during follow-up will be calculated for participants randomized to pitavastatin and, separately, their counterparts randomized to placebo. The statistical significance of differences within sub-groups will be calculated using interval censored regression models and chi-square tests as described in Aim 1 above. We will use multiplicative interaction terms (eg, black race * pitavastatin) to assess differences in the effect of pitavastatin on CKD across sub-groups. We will evaluate CD4 count as a continuous variable to determine if there is a threshold below which statin therapy has greater benefit for kidney function. We will model the interaction between pitavastatin versus placebo and CD4 count as a continuous variable with interval censored regression models and logistic regression for the cumulative incidence [Howard 2011].

OBJECTIVE 3: To determine whether the effect of pitavastatin on kidney function is mediated through anti-inflammatory effects. This analysis will follow the traditional case-cohort design. The case-cohort design combines the advantages of cohort studies and case-control analyses. In a case-cohort design, the comparison group is not defined on the basis of the "absence" of the outcome (≥30% eGFR decline or ACR >30 mg/g; as in a traditional "case-control" study

design). This is advantageous for the following reasons: 1) the strategy allows for studying multiple endpoints using the same "control" group (ie, the sub-cohort); 2) it bypasses a certain degree of arbitrariness unavoidable when attempting to define the comparison group intended to include only “non-cases” (ie, the inclusion of “false negatives”); 3) the strategy allows a random sample of the cohort for valid cross-sectional comparisons (eg, the association between baseline ACR and biomarkers of CKD and systemic and vascular inflammation and oxidative stress in the cohort random sample); and 4) it allows calculation of hazard ratios using survival models rather than relying on logistic regression and calculation of odds ratios.

The distribution of biomarkers at baseline and during follow-up will be graphed. For biomarkers that are not normally distributed, an appropriate transformation will be performed (eg, log transformation). Mean baseline and change in the levels of these biomarkers will be calculated for participants who experience a ≥30% decline in eGFR and the sub-cohort. Differences will be calculated using ANOVA with weighting to account for the probability of selection into the sub-cohort. Next, we will calculate the association between biomarkers of CKD, systemic and vascular inflammation and oxidative stress with CKD progression using a pseudo-likelihood, weighted Cox regression model. Using the approach of Barlow and Prentice for case-cohort analysis, we will calculate the hazard ratio for a ≥30% decline in eGFR associated with each biomarker separately. Initial models will control for age, race, and sex. Subsequent models will include further adjustment for systolic blood pressure, total and HDL-cholesterol, cigarette smoking, waist circumference, and baseline eGFR and ACR. A final model will include further adjustment for $\log_{10}$ HIV RNA load and $CD4^+$ T-cell counts.

Next, we will conduct a mediation analysis to test whether pitavastatin lowers the incidence of ≥30% eGFR decline by reductions in biomarkers assessed. To address mediation that pitavastatin will reduce the incidence of eGFR decline through improvements in systemic and vascular inflammation and oxidative stress, we will apply the regression technique described by Judd and Kenny and elaborated by MacKinnon [Judd 1981; MacKinnon 1994]. The technique, will account for each biomarker separately and for all biomarkers simultaneously (mediators) using two regression equations (**Table 2**). Y is the outcome result (eg, eGFR decline ≥30%), X_p is randomization to pitavastatin versus placebo and X_i represents the k biomarkers, our purported mediators. The total effect of pitavastatin on the outcome (measured by the coefficient, τ) is the sum of direct effect (τ') of treatment and the mediated, or indirect, effects. The assessment of the amount of change in these biomarkers mediating the effect of pitavastatin on eGFR decline will be addressed by considering two multivariable regression models (see **Table 2**). First, a regression model that estimates the decreased risk of a ≥30% eGFR decline with pitavastatin without mediating risk factors but considering other risk factors (eg, age, race, sex, hypertension) and a second Cox regression model that further makes adjustment for the mediating risk factor (ie, biomarkers of inflammation and oxidative stress). For these analyses, the parameter of interest (ie, log hazard ratio) is not the impact of pitavastatin on outcomes, but rather the difference in the estimated impact of pitavastatin between the two models (τ' - τ), which serves as a direct measure of the degree to which the effect of pitavastatin is due to improvements in these biomarkers. Whether the biomarkers “significantly” mediate the benefits of pitavastatin can be addressed by creating empirical confidence intervals around the change in its coefficient. Confidence interval of the change in the coefficient associated with the disparity will be calculated using bootstrap methods.

Table 2: Regression Equations for Mediation Analysis
A. $Y = \beta_0 + \tau X_p + \varepsilon$
B. $Y = \beta_0 + \tau' X_p + \beta_1 X_1 + \ldots + \beta_i X_i + \ldots + \beta_k X_k + \varepsilon$ for all mediators together
OR
$Y = \beta_0 + \tau' X_p + \beta_i X_i \ \varepsilon$ for each mediator separately

REFERENCES

Althoff KN, McGinnis KA, Wyatt CM, et al; Veterans Aging Cohort Study (VACS). Comparison of risk and age at diagnosis of myocardial infarction, end-stage renal disease, and non-AIDS-defining cancer in HIV-infected versus uninfected adults. Clin Infect Dis 2015;60(4):627-38.

Amarenco P, Callahan 3rd A, Campese VM, et al. Effect of high-dose atorvastatin on renal function in subjects with stroke or transient ischemic attack in the SPARCL trial. Stroke 2014;45(10):2974-82.

Bhasin B, Lau B, Atta MG, et al. HIV viremia and T-cell activation differentially affect the performance of glomerular filtration rate equations based on creatinine and cystatin C. PLoS One 2013;8(12):e82028.

Brenchley JM, Price DA, Schacker TW, et al. Microbial translocation is a cause of systemic immune activation in chronic HIV infection. Nat Med 2006;12(12):1365-71.

Chronic Kidney Disease Prognosis Consortium; Matsushita K, van der Velde M, Astor BC, et al. Association of estimated glomerular filtration rate and albuminuria with all-cause and cardiovascular mortality in general population cohorts: a collaborative meta-analysis. Lancet 2010;375(9731):2073-81.

Colhoun HM, Betteridge DJ, Durrington PN, et al. Effects of atorvastatin on kidney outcomes and cardiovascular disease in patients with diabetes: an analysis from the Collaborative Atorvastatin Diabetes Study (CARDS). Am J Kidney Dis 2009;54(5):810-9.

Coresh J, Astor BC, Greene G, Eknoyan G, Levey AS. Prevalence of chronic kidney disease and decreased kidney function in the adult US population: Third National Health and Nutrition Examination Survey. Am J Kidney Dis 2003;41(1):1-12.

Deeks SG. HIV infection, inflammation, immunosenescence, and aging. Ann Rev Med 2011;62:141-55.

Dolan SE, Hadigan C, Killilea KM, et al. Increased cardiovascular disease risk indices in HIV-infected women. J Acquir Immune Defic Syndr 2005;39(1):44-54.

Eknoyan G, Hostetter T, Bakris GL, et al. Proteinuria and other markers of chronic kidney disease: a position statement of the National Kidney Foundation (NKF) and the National Institute of Diabetes and Digestive and Kidney Diseases (NIDDK). Am J Kidney Dis 2003;42(4):617-22.

Gagneux-Brunon A, Mariat C, Delanaye P. Cystatin C in HIV-infected patients: promising but not yet ready for prime time. Nephrol Dial Transplant 2012;27(4):1305-13.

Gathogo E, Jose S, Jones R, et al. End-stage kidney disease and kidney transplantation in HIV-positive patients: an observational cohort study. J Acquir Immune Defic Syndr 2014;67(2):177-80.

Gerstein HC, Mann JF, Yi Q, et al. Albuminuria and risk of cardiovascular events, death, and heart failure in diabetic and nondiabetic individuals. JAMA 2001;286(4):421-6.

Gupta SK, Mamlin BW, Johnson CS, Dollins MD, Topf JM, Dubé MP. Prevalence of proteinuria and the development of chronic kidney disease in HIV-infected patients. Clin Nephrol 2004;61:1-6.

Howard G, Cushman M, Kissela BM, et al.; REasons for Geographic And Racial Differences in Stroke (REGARDS) Investigators. Traditional risk factors as the underlying cause of racial disparities in stroke: lessons from the half-full (empty?) glass. Stroke 2011;42(12):3369-75.

Inker LA, Wyatt A, Creamer R, et al. Performance of creatinine and cystatin C GFR estimating equations in an HIV-positive population on antiretrovirals. J Acquir Immune Defic Syndr 2012;61(3):302-9.

Inker LA, Heerspink HGL, Mondal H, et al. GFR decline as an alternative end point to kidney failure in clinical trials: a meta-analysis of treatment effects from 37 randomized trials. Am J Kidney Dis 2014;64(6):848-59.

Jotwani V, Li Y, Grunfeld C, Choi AI, Shlipaket MG. Risk factors for ESRD in HIV-infected individuals: traditional and HIV-related factors. Am J Kidney Dis 2012;59(5):628-35.

Judd PL, Kenny DJ. Process analysis: estimating mediation in treatment evaluations. Evaluation Review;1981;5(5):602-19.

Levey AS, Becker C, Inker LA. Glomerular filtration rate and albuminuria for detection and staging of acute and chronic kidney disease in adults: a systematic review. JAMA 2015;313(8):837-46.

Lucas GM, Mehta SH, Atta MG, et al. End-stage renal disease and chronic kidney disease in a cohort of African-American HIV-infected and at-risk HIV-seronegative participants followed between 1988 and 2004. AIDS 2007;21(18):2435-43.

Lucas GM, Lau B, Atta MG, Fine DM, Keruly J, Moore RD. Chronic kidney disease incidence, and progression to end-stage renal disease, in HIV-infected individuals: a tale of two races. J Infect Dis 2008;197(11):1548-57.

Lucas GM, Ross MJ, Stock PG, et al.; HIV Medicine Association of the Infectious Diseases Society of America. Clinical practice guideline for the management of chronic kidney disease in patients infected with HIV: 2014 update by the HIV Medicine Association of the Infectious Diseases Society of America. Clin Infect Dis 2014;59(9):e96-138.

MacKinnon DP. Analysis of mediating variables in prevention and intervention research. NIDA Res Monogr 1994;139:127-53.

Mallipattu SK, Liu R, Zhong Y, et al. Expression of HIV transgene aggravates kidney injury in diabetic mice. Kidney Int 2013;83(4):626-34.

Matsushita K, Selvin E, Bash LD, Astor BC, Coresh J. Risk implications of the new CKD Epidemiology Collaboration (CKD-EPI) equation compared with the MDRD Study equation for estimated GFR: the Atherosclerosis Risk in Communities (ARIC) Study. Am J Kidney Dis 2010;55(4):648-59.

Medapalli RK, Parikh CR, Gordon CR, et al. Comorbid diabetes and the risk of progressive chronic kidney disease in HIV-infected adults: data from the Veterans Aging Cohort Study. J Acquir Immune Defic Syndr 2012;60(4):393-9.

Nakamura T, Sugaya T, Kawagoe Y, Ueda Y, Osada S, Koide H. Effect of pitavastatin on urinary liver-type fatty acid-binding protein levels in patients with early diabetic nephropathy. Diabetes Care 2005;28(11):2728-32.

Overton ET, Patel P, Mondy K, et al. Cystatin C and baseline renal function among HIV-infected persons in the SUN Study. AIDS Res Hum Retroviruses 2012;28(2):148-55.

Palmer SC, Navaneethan SC, Craig JC, et al. HMG CoA reductase inhibitors (statins) for people with chronic kidney disease not requiring dialysis. Cochrane Database Syst Rev 2014;5:CD007784.

Ross AC, Armentrout R, O'Riordan MA, et al. Endothelial activation markers are linked to HIV status and are independent of antiretroviral therapy and lipoatrophy. J Acquir Immune Defic Syndr 2008;49(5):499-506.

Strategies for Management of Antiretroviral Therapy (SMART) Study Group; El-Sadr WM, Lundgren JD, Neaton JD, et al. CD4+ count-guided interruption of antiretroviral treatment. N Engl J Med 2006;355(22):2283-96.

Szczech LA, Hoover DR, Feldman JG, et al. Association between renal disease and outcomes among HIV-infected women receiving or not receiving antiretroviral therapy. Clin Infect Dis 2004;39:1199-206.

Szczech LA, Menezes P, Quinlivan EB, van der Horst C, Bartlett JA, Svetkey L. Microalbuminuria predicts overt proteinuria among patients with HIV-infection. HIV Med 2010;11(7):419-26.

Tonelli M, Sacks F, Pfeffer M, et al. Biomarkers of inflammation and progression of chronic kidney disease. Kidney Int 2005;68(1):237-45.

Wyatt CM, Hoover Dr, Shi Q, et al. Microalbuminuria is associated with all-cause and AIDS mortality in women with HIV infection. J Acquir Immune Defic Syndr 2010;55(1):73-7.

Wyatt CM, Hoover Dr, Shi Q, et al. Pre-existing albuminuria predicts AIDS and non-AIDS mortality in women initiating antiretroviral therapy. Antivir Ther 2011;16(4):591-6.

Yagi S, Akaike M, Aihara KI, et al. Effect of low-dose (1 mg/day) pitavastatin on left ventricular diastolic function and albuminuria in patients with hyperlipidemia. Am J Cardiol 2011;107(11):1644-9.

SUMMARY OF CHANGES

REPRIEVE (A5332) FINAL Version 3.0, 01/28/16

Randomized Trial to Prevent Vascular Events in HIV – REPRIEVE (A5332)

The main purpose of this amendment is to incorporate the following changes:

- Raise the 10-year ASCVD risk score exclusion criterion to >10%, as approved by the FDA and DSMB.
- Add 2 new ancillary objectives: 1) to assess the influence of sex/reproductive aging status on immune activation and statin-induced immunomodulation in relation to clinical CVD events, and 2) to determine the effects of pitavastatin on the incidence of changes in kidney function.

A summary of changes follows. New text is indicated by bold font in the protocol. Deletions, minor clarifications, and renumbering are not outlined in this summary.

1. Global changes that were made throughout the REPRIEVE (A5332) protocol document include:
   - Where appropriate, “ASCVD risk score” was changed to read “10-year ASCVD risk score” to ensure use of the correct risk calculator to determine eligibility.
   - References to “standard of care,” “routine care,” and “routine clinical care” were changed to “clinical care” for consistency except in the sample informed consent.
   - Mention of keying and entering data into OpenClinica, CRFs, or the database were changed to “record in the EDC” for consistency.

2. The National Institute of Diabetes and Digestive and Kidney Diseases (NIDDK) was added to the A5332 title page as a study funder.

3. Sites Participating in the Study was revised to include select international sites that are not part of the ACTG network.

4. Section 1.2.3, Ancillary Objectives, was added. Background, more detailed objectives and hypotheses, statistical considerations, and references related to these ancillary objectives are in new Appendices IV and V. Evaluations related to these objectives were folded into the A5332 Schedule of Events. These evaluations are additional urine collections post entry for participants at ACTG sites only, and for all participants, some additional blood/plasma banking during the study. The sample informed consent template was revised to include the additional evaluations and increase in blood volumes at the relevant visits.

5. Section 2.1.5, Rationale for the Change in 10-Year ASCVD Risk Score, was added (per Letter of Amendment (LOA) #1 dated 09/18/15).

6. Section 3.0, Study Design, was revised to change the study population to those considered low-to-moderate risk using the 2013 ACC/AHA guideline thresholds for recommending statin therapy.

7. Section 4.1, Inclusion Criteria

- 4.1.1 was changed to allow additional and less stringent methods of documenting HIV-1 infection to ease site burden (per Clarification Memo (CM) #3 dated 09/10/15).
- 4.1.3 removes the instruction that the CD4+ cell counts are obtained from standard of care (now clinical care) to avoid duplicating information in 6.3.6.
- 4.1.4 adds a note about acceptable LDL measurements for different 10-year ASCVD risk scores. The same information is in 4.2.3. The definition of fasting was removed to avoid duplicating information in section 6.0.
- 4.1.6 was clarified to allow use of pregnancy tests at clinics as well as laboratories that meet the stated requirements, and allow use of a point-of-care/CLIA-waived test. Tubal ligation and tubal micro-inserts were removed from the note because they are not considered forms of surgical sterilization (per CM #1 dated 02/02/15).
- 4.1.7 was clarified to 1) cover women of "reproductive" rather than "child-bearing" potential to be consistent with 4.1.6, 2) remove tubal ligation as a form of sterilization, 3) add tubal ligation and tubal micro-inserts as methods of contraception (per CM #2), and 4) specify that condoms may be used with or without a spermicidal agent.

8. Section 4.2, Exclusion Criteria
   - 4.2.2 corrects the LDL measurement for candidates with diabetes from >70 mg/dL to ≥70 mg/dL.
   - 4.2.3 revises the 10-year ASCVD risk score exclusion to >10% and the notes to include the LDL measurement for individuals with a risk score 7.5-10% (must be <160 mg/dL) and >10% (<70 mg/dL permitted to enroll).
   - 4.2.9 clarifies that specific immunomodulatory and immunosuppressants are exclusionary but are not limited to the list on this criterion and directs the user to the MOPS for additional information about drugs in these classes; adds a note to allow oral prednisone ≤10 mg/day.
   - 4.2.11 added bile acid sequestrants and PCSK9 inhibitors to the list of lipid-lowering agents prohibited in the 90 days prior to entry.

9. Section 4.3.2, Subject Randomization, updates the name of the screening failure form and the electronic data entry system, and specifies that participants who consent to the study but then are not randomized must have a screening outcome form recorded in the EDC.

10. Section 4.5, Coenrollment Guidelines, for emphasis underlines the statements encouraging ACTG sites to coenroll participants into A5128 and A5243.

11. Section 5.0, Study Treatment
    - 5.1.2, Administration, clarifies the timing of study treatment initiation for participants in the Mechanistic Substudy (A5333s) (per CM #1) and clarifies that participants should return all opened and not yet opened study product at each visit.
    - 5.4, Concomitant Medications, updates the name and web address of the database that sites may use for additional information about concomitant medications.
    - 5.4.2, Recommended Medications, removed the recommended prophylaxis that was specific to *Pneumocystis jiroveci* pneumonia and *Mycobacterium avium* and now refers sites to the current guidelines for the prevention and treatment of opportunistic infections in HIV-infected persons.

12. Section 6.0, Clinical and Laboratory Evaluations
    - The Schedule of Events table was updated to clarify which lab tests are to be collected from clinical care and which need to be drawn for the study, as well as to reflect the changes made to section 6.0 noted below.
    - Test results that may be obtained from clinical care are so noted throughout section 6.0.
    - 6.2.2, Entry Evaluations, now includes the clarification from 5.1.2 about the initiation of study drug for participants in the Mechanistic Substudy (A5333s).
    - Visit windows for months 4 through 72, and the Premature Treatment Discontinuation visit, were widened to ±21 days (month 1 remains at ±7 days).
    - Mention of a medication questionnaire was changed throughout section 6.0 to adherence assessment, because this assessment is done by site staff and is not a patient questionnaire.
    - 6.3, Instructions for Evaluations and Data Collection, adds a directive to refer to the MOPS for additional details about data to be maintained in source documents.
    - 6.3.4, Clinical Assessments, Signs and Symptoms, clarifies the recording of signs and symptoms before and after study entry (further revised since CM #2 dated 08/28/15). Also in this section:
        - Because the assessment of potential myalgia toxicity is done via site staff interview, myalgia symptom “questionnaire” was changed to myalgia symptom “assessment.”
        - Noted that e-cigarette use is not considered current active smoking when calculating the ASCVD risk score.
        - Waist circumference will be measured at the week 72 visit (per CM #1).
    - 6.3.6, Laboratory Evaluations, was revised for clarity. All laboratory values collected for REPRIEVE must be recorded in the EDC. A statement was added at the beginning of this subsection to cover all lab testing with respect to lab certification: All laboratory testing done for screening and safety assessment must be done by a CLIA or equivalent certified lab or at any network-approved non-US lab that operates in accordance with GCLP and participates in appropriate QA programs. Other changes in this section include:
        - At the entry visit, if a plasma HIV-1 RNA result is not available from clinical care within the required timeframe, it will not be collected.
        - At screening, if the lipid panel is not available from clinical care within the required timeframe, it may be drawn as part of the study. Also, if it is unclear or unknown if this lipid panel was done while the candidate was fasting AND the values meet the requirements for eligibility in 4.1.4, they do not need to be redone while fasting and there is no need to document fasting status (per CM #2). The definition of fasting was moved to the beginning of 6.3.6 so as to cover all fasting evaluations and not just the lipid panel.
        - From entry onwards, it was clarified that collection of stored samples for future lipid and glucose testing are optional for participants (to be consistent with the sample consent). The reference to these results being blinded until the end of the study was removed because that erroneously implied that results would be made available to sites after the study.
        - At screening, if the liver function tests are not available from clinical care within the required timeframe, they may be drawn as part of the study.
        - Clarified the circumstances under which AST and ALT have to be collected.

    - Additional urine samples for an ancillary objective, which are collected only at ACTG sites, will be stored and tested centrally as indicated in the Schedule of Events.
- 6.3.7 now includes future hormonal studies in addition to immunologic and biomarker studies. A note was added to the Schedule of Events and this section to state that participants may opt out of these tests from entry onwards, to be consistent with the sample consent.

13. Section 8.1, Premature and Permanent Treatment Discontinuation, corrects an error in the note, removing the instruction to followed participants who *temporarily* discontinue study treatment on study/off treatment.

14. Section 9.1, General Design Issues, adds an explanation of ancillary studies and notes that additional details for the 2 new ancillary objectives are in Appendices IV and V.

15. Section 11.1, Records to be Kept, clarified that electronic case report forms are available on the DMC website, changes the ACTG DMC to DMC, and adds that participants are identified by screening number as well as PID and SID.

16. Section 11.4.1, Adverse Event Reporting to DAIDS, corrects the name of DAERS to the DAIDS Adverse Experience Reporting System and updates the DAIDS-ES support email address, which is now part of the NIAID Clinical Research Management System.

17. The Table of Contents, Protocol Team Roster, Study Management, Glossary of Protocol-Specific Terms, and References sections were updated. The Schema and Executive Summary were updated to reflect changes in the protocol.

<u>APPENDIX I: Sample Informed Consent (A5332)</u>

1. For clarity throughout the consent template, boxes were drawn around portions of the consent that only apply to a subset of participating sites, eg, ACTG sites, non-US sites. If the text inside the boxes does not apply to a particular site, it may be omitted from the local consent document. Only ACTG sites need to add the new text under Urine Collected that covers samples collected at months 12, 24, and 48 (these are needed to address the kidney function ancillary objective).

2. WHY IS THIS STUDY BEING DONE?
    - Revised the third paragraph and added the fourth paragraph to include more information about the risk calculator and provide the rationale for including people with ASCVD risk scores between 7.5% and 10% to the study.

3. WHAT DO I HAVE TO DO IF I AM IN THIS STUDY?
    - The Study Procedures table was revised to add urine collection to additional time points at ACTG sites only, and to merge the rows for Blood Collected and Fasting Blood Tests.

- In the Study Procedures table and the table of early discontinuation procedures, removed the Pill Count Questionnaire row, as medication adherence is done by site staff during the pill count and not by patient questionnaire.
- Under the Explanation of Study Procedures, Blood Collected, clarified that the screening liver function tests and cholesterol level will be captured from clinical care, increased the blood volume for blood draws at entry, month 12, 24, 48, and end of study to cover future studies which will be approved by the REPRIEVE team and not the ACTG.

4. WHAT ARE THE RISKS OF THE STUDY?
   - In Risks of fasting, removed '…and not smoking or consuming caffeine…' as possibly bothersome, because smoking and consuming caffeine are not prohibited.

5. ARE THERE RISKS RELATED TO PREGNANCY?
   - Specified that male or female condoms with or without spermicidal agents and tubal micro-inserts are acceptable methods of birth control to be consistent with 4.1.7.

6. WHAT IF WE CAN NO LONGER REACH YOU DURING YOUR STUDY PARTICIPATION?
   - Revised Contacting Your Health Care Provider to request permission from the participant for the site staff to share information about the ASCVD risk score with the local health care provider, and note that the local provider may object to the patient taking part in a placebo controlled study.

7. WILL I RECEIVE ANY PAYMENT?
   - Corrected the recommended compensation statement so that month 6 now reads month 60 (per CM #1).

<u>APPENDIX II: The Mechanistic Substudy of REPRIEVE (A5333s)</u>

1. 4.2.5, Exclusion Criterion, allows use of pregnancy tests at clinics as well as laboratories that meet the stated requirements, and allows use of a point-of-care/CLIA-waived test. Tubal ligation and tubal micro-inserts were removed from the note because they are not considered forms of surgical sterilization (per CM #1 and CM #2).
2. 4.4, Subject Registration, changed ACTG data management procedures to DMC procedures.
3. 6.1, Schedule of Events, and 6.2.2, Post-entry evaluations, widened the month-4 visit window to ±21 days to match the main study. In SOE footnote 6 and 6.3.2, removed reference to the fasting lipid panel results being blinded until the end of the study because that erroneously implied that results would be made available to sites after the study.
4. 6.2.1, Entry, clarified that the baseline CCTA should be completed within 14 days after enrollment and not 14 days after the initiation of REPRIEVE (A5332) study medication.
5. 9.3, Randomization/Registration, changed ACTG enrollment system to enrollment system.

APPENDIX III, Sample Informed Consent (Mechanistic Substudy A5333s)

1. WHAT ARE THE RISKS OF THE SUBSTUDY?
   - In Risks of Drawing Blood, removed the reference to not smoking or consuming caffeine to be consistent with the protocol.

APPENDIX IV: REPRIEVE Objectives to Decipher Sex-Specific Mechanisms of CVD Risk and Risk Reduction, new in its entirety to provide details about the ancillary objective 1.2.3.1.

APPENDIX V: REPRIEVE Ancillary Study: Effect of Pitavastatin on Kidney Function in HIV-infected Persons, new in its entirety to provide details about ancillary objective 1.2.3.2.

SUMMARY OF CHANGES

REPRIEVE (A5332) FINAL Version 4.0, 03/28/18

Randomized Trial to Prevent Vascular Events in HIV – REPRIEVE (A5332)

The main purpose of this amendment is to make the changes recommended by the Data Safety and Monitoring Board (DSMB) at their December 2017 meeting.

Revisions and clarifications made in Version 3.0 Letters of Amendment (LOAs) and Clarification Memos (CMs) were incorporated, except for minor revisions, which are not listed below. Where appropriate, further revisions have been made to the information that was added to the protocol via the CMs and LOAs.

A summary of changes follows. Minor corrections were made in the protocol document for accuracy, consistency, or clarity, which are not listed below. The amendment shows additions in bold font while deletions are not marked.

1. These global changes were made throughout the protocol document where appropriate:
   - Formatting changes were made to comply with the US FDA format required for electronic submissions.
   - ‘REPRIEVE’ was changed to ‘REPRIEVE (A5332)’ for accuracy.
   - The Schedule of Events was changed to Schedule of Evaluations per the current protocol template.
   - The ‘start of study’ was changed to ‘study entry’ for consistency.
   - References to the ‘ACTG Statistics and Data Analysis Center’ were deleted because the statistical center for the trial is the Center for Biostatistics in AIDS Research.
   - The web links for these resources were updated:
     - DAIDS Protocol Registration Manual
     - ACTG Precautionary and Prohibited Medications Database
     - DAIDS Source Document Guidelines

- DAIDS Expedited Adverse Event Manual

  Note: The web link for the online ASCVD Risk Estimator was removed from 6.3.4 Clinical Assessments because the calculator was modified by the ACC/AHA. While it provides the same 10-Year ASCVD Risk Score needed to assess eligibility, it calls for the input of additional data that are not collected by REPRIEVE. For this reason, and to ensure consistency in calculating the risk score throughout the trial, the protocol now directs sites to the MOPS for information on accessing and using the 10-Year ASCVD Risk Score calculator available in a spreadsheet available on the protocol-specific website.

2. The Office of AIDS Research at the National Institutes of Health and Gilead Sciences were added to the title page of A5332 and A5333s as a study funder and industry supporter, respectively. The NHLBI Program and Medical Officer was changed to Patrice M. Desvigne-Nickens, MD.

3. To comply with DAIDS regulatory requirements, a signature page was added immediately after the title page and is to be submitted with the amendment registration packet. Also, the NHLBI and other local, US, and international regulatory entities were added to the agencies that may obtain study documents for monitoring purposes in 11.3 Clinical Site Monitoring and Record Availability and 12.2 Subject Confidentiality, as well as the Sample Informed Consents for both REPRIEVE (A5332) and the substudy A5333s.

4. The phase of the trial was changed from phase IV to phase III per DAIDS regulatory guidance. This change was made in 3.0 Study Design and 9.1 General Design Issues.

5. The Executive Summary, Protocol Team Roster, Study Management, Glossary of Protocol-Specific Terms, and References sections were updated.

6. Per the December 2017 DSMB recommendations, the sample size was increased to approximately 7500 participants, study follow-up was increased by approximately 1 year (adding study visits at months 76, 80, and 84), and the enrollment of people with 10-year ASCVD risk scores (less than 2.5%) was capped at approximately 2000 participants. These changes were made in the following protocol sections:
   - 2.1 Background
   - 3.0 Study Design
   - 5.1.3 Treatment Duration
   - 6.1 Schedule of Evaluations (SOE) (3 additional study visits)
   - 9.1 General Design Issues
   - 9.4.2 Design Considerations Following December 2017 DSMB Review (new section)
   - Appendix I Sample Informed Consent (SIC): HOW MANY PEOPLE WILL BE IN THIS STUDY?, WHAT DO I HAVE TO DO IF I AM IN THIS STUDY?, HOW LONG WILL I BE IN THIS STUDY? WILL I RECEIVE ANY PAYMENT?

7. The previous increase in the upper bound of the 10-year ASCVD risk score from 10% to 15% (LOA #1 dated 08/17/16) and the associated changes in permissible LDL cholesterol values per risk score were changed in these protocol sections:
   - 2.1.5 Rationale for the Change in 10-Year ASCVD Risk Score

- Inclusion criterion 4.1.4, first bullet
- Exclusion criterion 4.2.3
- Appendix I SIC: WHY IS THIS STUDY BEING DONE?, WHAT IF WE CAN NO LONGER REACH YOU DURING YOUR STUDY PARTICIPATION? Contacting Your Health Care Providers

8. The duration of allowable ART treatment interruptions in the 180 days before entry was changed from up to 14 days total to up to 30 days total in inclusion criterion 4.1.2 and 5.4.1 Required Medications (LOA #1).

9. Glomerular filtration rate (GFR) was added as an acceptable method to assess renal function (LOA #1) in these protocol sections:
   - Inclusion criterion 4.1.4
   - 6.3.6 Laboratory Evaluations
   - APPENDIX II: Inclusion criterion 4.1.4, 6.1 SOE, 6.3.2 Laboratory Evaluations, 8.0 Criteria for Substudy Discontinuation

10. Exclusion criterion 4.2.4 was changed to exclude active cancers within 12 months prior to entry (LOA #2 dated 04/14/17) and subsequently, the exceptions in the note were clarified.

11. Exclusion 4.2.5 was changed to exclude known decompensated cirrhosis (LOA #2).

12. The clarification that dosage equivalent to prednisone ≤10 mg/day is allowed in the 30 days prior to entry is clarified in inclusion criterion 4.2.9.

13. The list of exclusionary lipid-lowering agents in 4.2.11 was revised to remove fibrates, ezetimibe, red yeast rice, niacin, bile acid sequestrants, and omega-3 fatty acids, and add gemfibrozil (LOA #1); this amendment subsequently deletes the note about allowing niacin as part of a multivitamin, which was overlooked in LOA #1.

14. The timing of coenrollment into The Mechanistic Substudy of REPRIEVE (A5333s) was clarified (CM #3 dated 12/27/16). This change is reflected in 4.4 Coenrollment and Appendix II 4.3 Enrollment Procedures. Subsequently, the A5333s substudy closed to accrual on 02/06/2018 and a note to that effect was added to these same sections.

15. A delay of up to 14 days before initiating study treatment is allowed if coenrolling in A5333s and select ancillary studies (LOA #2). This change was added to these protocol sections:
   - 5.1.2 Administration
   - 6.2.2 Entry Evaluations
   - Appendix II: 4.3 Enrollment Procedures and 6.2.1 Entry

16. Pill counts were eliminated but medication adherence assessments were maintained (LOA #2) and those changes were made in these protocol sections:
   - 5.1.2 Administration
   - 6.1 SOE
   - 6.2.4 Discontinuation Evaluations, Premature Treatment Discontinuation Evaluations
   - 6.3.9 Medication Adherence Assessment

- Appendix I SIC: WHAT DO I HAVE TO DO IF I AM IN THIS STUDY?

17. It was clarified in 5.1.2 Administration that the drug dispensation schema is the preferred schedule and instructions on what to do if there are variations to the schema were added.

18. An instruction to contact the core team with questions and refer to the MOPS for additional information was added to 5.4 Concomitant Medications. An explanation that the ACTG Precautionary and Prohibited Medications database provides data on drug interactions with antiretrovirals was also added to this section.

19. The footnotes in the SOE were clarified to provide consistent directions with regard to when labs are collected from clinical care and when they must be drawn for the study, and to reflect other changes made to study evaluations listed elsewhere in this change summary.

20. The window for participants to return for fasting blood work was widened from 7 to 21 days (LOA #1). Changes were made in these protocol sections:
    - 6.1 SOE
    - 6.2.3 Post-Entry Evaluations, <u>Return Fasting Visit (as needed)</u>
    - 6.3.6 Laboratory Evaluations
    - Appendix I SIC: WHAT DO I HAVE TO DO IF I AM IN THIS STUDY?

21. The requirement for an AST at screening was clarified in 6.1 SOE (CM #1 dated 04/01/16, CM #2 dated 04/12/16, and CM #3 dated 12/27/16).

22. The ancillary study ‘Effect of Pitavastatin on Kidney Function in HIV-infected Persons’ was closed to new enrollees on 01/01/18 and only people enrolled in REPRIEVE at ACTG sites prior to that date will continue to have urine samples saved at months 12, 24, and 48. These protocol sections were revised:
    - 6.1 Schedule of Evaluations including footnote #11
    - 6.3.6 Laboratory Evaluations, <u>Urine Albumin/Creatinine Ratio</u>
    - Appendix I SIC: WHAT DO I HAVE TO DO IF I AM IN THIS STUDY? in the box under <u>Urine Collected</u>

23. The instructions to site staff concerning participants who discontinue the study before initiating study treatment were corrected in 6.2.4, Discontinuation Evaluations.

24. The grading tables have been updated to the DAIDS Table for Grading the Severity of Adult and Pediatric Adverse Events, corrected version 2.1, dated July 2017. This change is reflected in the following protocol sections:
    - 6.3 Instructions for Evaluations and Data Collection
    - 7.0 Clinical Management Issues
    - 9.2.2.6
    - 11.4.3 Grading Severity of Events

25. In 6.3, Medication History, Table 6.3.3-1 was annotated to direct the reader to the MOPS for information about recording antihypertensive medications, hormonal contraceptives, hormonal replacement therapy, and testosterone therapy.

26. Venous thromboembolism and pulmonary thromboembolism were added to the list of non-CVD events to be recorded. See 6.3.2 Medical History and 6.4.4 Non-CVD Events (CM #1).

27. The timing of the targeted physical exam in 6.3.4 Clinical Assessments was changed to be driven by diagnoses since the last visit instead of within the 30 days prior to the last visit. In Waist Circumference, the correction made to the measurements at screening and month 72 (CM #1) was further changed in this amendment to have the measurements done at screening and month 84.

28. In 6.3.6 Laboratory Evaluations, CD4+/CD8+, the note was clarified to state that a CD4+ cell count alone is acceptable if a CD8+ cell count is not available.

29. Text was added to 6.4 Endpoint Assessments to state that non-CVD events should also be assessed and reported in the EDC, regardless of grade, even though they are not formally adjudicated by the Clinical Event Committee.

30. The instructions on how to capture vital status for participants lost to follow-up were clarified to be consistent in 6.3.10 Vital Status Follow-up and 8.2 Premature Study Discontinuation.

31. The management of Grade 4 toxicities in 7.1.1, General Reactions, was clarified (LOA #2).

32. The name of the ACTG Unblinding SOP was revised to the current title ACTG Unblinding Participants SOP 123 in 7.4 Unblinding Procedures.

33. ‘Participant refusal to continue study treatment’ was added to the list of reasons for stopping study treatment prematurely in 8.1 Premature and Permanent Treatment Discontinuation.

34. ‘Subject judged by the investigator to be at significant risk of failing to comply with the provisions of the protocol as to cause harm to self or seriously interfere with the validity of the study results’ was removed from 8.2 Premature Study Discontinuation and Appendix II 8.0 Criteria for Substudy Discontinuation.

35. It was clarified that the clinical diagnoses of interest are incident diagnoses in the Outcome Measure subsection 9.2.2.4.

36. In the Secondary Analyses section 9.6.3, month 72 was added to the cholesterol analysis.

37. In Appendix I WHAT DO I HAVE TO DO IF I AM IN THIS STUDY?, deleted the duration of participant follow-up that duplicates the information in HOW LONG WILL I BE IN THIS STUDY?

38. In Appendix II SOE and Appendix III WHAT DO I HAVE TO DO IF I AM IN THIS SUBSTUDY?, clarified that a second CCTA is not done if the participant discontinues A5333s before month 12.

SUMMARY OF CHANGES
REPRIEVE (A5332) FINAL Version 5.0, 04/01/19
Randomized Trial to Prevent Vascular Events in HIV – REPRIEVE (A5332)

The main purpose of this amendment is to lengthen study follow-up to 96 months, as endorsed at the December 2018 DSMB meeting, and to add language to allow for the capture of vital status and endpoint data annually for participants who prematurely discontinue the study, but have not withdrawn consent.

Revisions and clarifications made in Version 4.0 Letter of Amendment (LOA) #1 were incorporated. Where appropriate, further revisions have been made to the information that was added to the protocol via the LOA.

A summary of changes follows. Sections affected by each change are indicated in brackets. Minor corrections were made in the protocol document for accuracy, consistency, or clarity, which are not listed below. The amendment shows additions in bold font while deletions are not marked.

1. The Protocol Team Roster and the Glossary of Protocol-Specific Terms were updated.

2. “Subject” was replaced with “participant” or “individual” throughout the protocol, where appropriate.

3. The previous cap on enrollment of persons with low 10-year ASCVD scores was added, per Version 4.0, LOA #1, dated 05/16/18. [Sections 2.1.7, 3.0, 9.1, 9.3]

4. Study follow-up was increased by approximately 1 year, adding study visits at months 88, 92, and 96. [Executive Summary; sections 3.0, 5.1.3, 6.1, 6.3.4, 6.3.6, 9.1, 9.4.2, 9.6.3; Appendix I, Sample Informed Consent Form, WHAT DO I HAVE TO DO IF I AM IN THIS STUDY?, HOW LONG WILL I BE IN THIS STUDY?, and WILL I RECEIVE ANY PAYMENT?]

5. Links to the DAIDS Regulatory Support Center websites have been updated. [Sections 4.3.1, 6.3, 7.0, 11.4.1, 11.4.3]

6. Additional details were added about endpoint assessments for participants who miss visits. [Sections 4.6, 6.2.3, 6.4]

7. Annual collection of vital status and endpoints was added for participants who prematurely discontinue the study, but have not withdrawn consent. [Sections 6.1, 6.2.3, 6.2.4, 6.3.10, 8.2, 9.2.1, 9.2.2, 9.6.1; Appendix I, Sample Informed Consent Form, WHY WOULD THE DOCTOR TAKE ME OFF THIS STUDY EARLY? and WHAT IF WE CAN NO LONGER REACH YOU DURING THE STUDY?]

8. Visit windows for all post-entry evaluations and for return fasting visits were increased from ±21 to ±30 days. [Sections 6.1, 6.2.3, 6.2.4, 6.3.6; Appendix I, Sample Informed Consent Form, WHAT DO I HAVE TO DO IF I AM IN THIS STUDY?]

9. It was clarified that participants should hold study drug if they need to initiate systemic (oral or intravenous) cyclosporine. [Section 7.2]

10. Study monitoring plans were updated per recommendations from the 2018 DSMB meeting. The first scheduled interim efficacy analysis will be at approximately 40% of information. [Section 9.5]

SUMMARY OF CHANGES
REPRIEVE (A5332) FINAL Version 6.0, 16May2022
Randomized Trial to Prevent Vascular Events in HIV – REPRIEVE (A5332)

This amendment is being implemented for the following reasons:

- To extend follow-up until the study reaches its target of 288 primary MACE endpoints, or otherwise recommended for closure by the DSMB
- To add COVID-19-related secondary objectives

Revisions and clarifications made in Version 5.0 Letters of Amendment (LOA) #1 and #2 and Clarification Memos (CMs) #1 and #2 were incorporated. Where appropriate, further revisions have been made to the information that was added to the protocol via the LOAs and CMs. As a reminder, Appendix IA from LOA#2 applies to ACTG sites only. To note, Appendix IIIA: Sample Informed Consent Addendum for the Mechanistic Substudy from LOA #2, was not added to Version 6.0 because the substudy is complete.

The following changes have been made for Version 6.0 of A5332. These changes, except for deletions, are noted in bold in the protocol document. Minor corrections were made in the protocol document for clarification and editorial purposes; these revisions are not reflected in this list.

1. The Protocol Team Roster was updated.

2. ViiV was added as an industry supporter.

3. “HIV-infected persons/individuals/patients/participants/population” was changed to “people with HIV” to align with the current language recommended by NIAID. Those who do not have HIV are now referred to as “people without HIV.”

4. The study follow-up period was extended (months 100-120) so that the study continues until it either reaches its target of 288 primary major adverse cardiovascular events (MACE) endpoints or the DSMB recommends closure of the study. [Executive Summary; sections 3.0, 5.1.3, 6.1b, 6.2.3, 9.1, 9.2.1, 9.4.2, 9.5, 9.6.1, 9.6.2, and 9.6.3; Appendix I: Sample Informed Consent Form, WHAT DO I HAVE TO DO IF I AM IN THIS STUDY?, HOW LONG WILL I BE IN THE STUDY?].

5. Exploring the impact of SARS-CoV-2 on the study population was added, as the COVID-19 pandemic is considered an important cardiovascular risk factor. [Sections 1.2.2, 2.1.8, 2.2, 6.1a, 6.1b, 6.3.2, 6.3.3-1, 6.4.3, 9.2.2.6, 9.2.2.7, 9.4.4, 9.5.1, 9.6.4, and 11.4.2; Appendix I: Sample Informed Consent Form].

6. The clinical assessments were updated to include hormonal/reproductive health assessments related to CVD risk and diet and functional capacity at the study termination visits [Section 6.1 and 6.3.4; Appendix I: Sample Informed Consent Form, WHAT DO I HAVE TO DO IF I AM IN THIS STUDY?].

7. The timing of some assessments and laboratory evaluations were updated [Sections 6.3.4 and 6.3.6; Appendix I: Sample Informed Consent Form, WHAT DO I HAVE TO DO IF I AM IN THIS STUDY?].

8. Window of hematology and serum creatinine evaluations at termination visits was updated to 180 days instead of 90 [Section 6.3.6].

9. Timing of compensation to participants was added. [Appendix I: Sample Informed Consent Form, WILL I RECEIVE ANY PAYMENT?].

10. New ACTG template language was added [Sections 6.3 and 6.3.6, 11.3; Appendix I: Sample Informed Consent Form, WHAT ABOUT CONFIDENTIALITY?, and WHAT HAPPENS IF I AM INJURED?].

11. The References were updated to include second and third authors and additional references [Section 16].

Effects of Pitavastatin on Coronary Artery Disease and Inflammatory Biomarkers: Mechanistic Substudy of REPRIEVE (A5333S)

CBAR Author: Heather Ribaudo

REPRIEVE Lead Investigators: Michael Lu, Steve Grinspoon, Pam Douglas

Other Investigators: Borek Foldyna, Thomas Mayrhofer

1 Background

A5333s is an optional mechanistic substudy of REPRIEVE (A5332). Approximately 800 people with HIV (PWH) who are enrolled in REPRIEVE (A5332) were targeted for Mechanistic Substudy of REPRIEVE (A5333s) enrollment.

The overarching aim of the Mechanistic Substudy of REPRIEVE (A5333s) is to better understand the modulation of critical features of coronary plaque morphology with statin therapy, including the progression of noncalcified coronary atherosclerotic plaque (NCP) volume in HIV and the biological factors mediating these effects during statin treatment in HIV. The Substudy will determine potential statin effects to halt progression of non-calcified atherosclerotic plaque and to stabilize morphologic features of plaque vulnerability. Moreover, the study will identify biological factors mediating these changes – be it lipid parameters, such as LDL cholesterol, or markers of inflammation and immune activation. Finally, the study will evaluate whether presence and morphology of subclinical atherosclerotic plaque at baseline relates to CVD events independently of traditional CVD risk factors and markers of HIV-specific immune activation.

This is the Primary Statistical Analysis Plan for the Mechanistic Substudy of REPRIEVE. It expands on details of the statistical analysis plan that were defined in Appendix II of the REPRIEVE (A5332) protocol document. Major changes from the details defined in the protocol are outlined in the version history table below. An overview of the Substudy design is provided below; details of the substudy sample size considerations are in Appendix I, copied verbatim from the protocol.

In preparation for public release of the document with publication of the data, preliminary substudy data and published work from other groups that were used as sample tables have been redacted.

Version History

Version	Changes Made	Date Finalized
(Protocol)	Original Version (Version 2.0)	Dec 19, 2014
1.0	Clarification that primary outcomes are related to noncalcified plaque; additional secondary plaque outcomes added.<br>HIV-1 RNA and CD4 moved from secondary outcomes to risk factors. HgA1C removed throughout (no longer being tested).	Mar 20, 2023
1.1	For Published release of the SAP …<br>Section 7: Sample tables of published work from other groups redacted.<br>Appendix II: Draft consort based on early preliminary data removed.	October 4, 2023

1.1 A5333S Design Overview

Enrollment into A5333s occurred concurrently with enrollment and randomization into REPRIEVE (A5332) at select US ACTG sites participating in REPRIEVE (A5332). To ensure balance in treatment assignment in the Substudy, randomization in REPRIEVE (A5332) was stratified by anticipated Substudy participation.

Substudy participants were followed for 2 years with serial coronary computed tomography angiography (CCTA) performed at study entry and 2 years. Fasting blood samples for assessment of lipids and select soluble biomarkers were drawn at entry, month 4, and year 2; PBMCs for assessment of cellular markers of immune function were drawn at entry and year 2. The Substudy also included a 2-year assessment of REAP (diet and physical activity) and DASI (physical function), as well as quality of life assessment using the SF-36 at entry and year 2.

Entry blood draws and CCTA were scheduled to occur within 14 days after randomization and prior to the start of randomized treatment. Month 4 evaluations were to be performed ± 21 days and year 2 evaluations were to be performed ±28 days. In light of scheduling concerns and to maximize the collection of follow-up CT scans, the window for year 2 data collection was extended to up to 30 months after the entry CT.

1.2 A5333S Hypotheses

1.2.1 Primary Hypothesis

Statin therapy will reduce progression of noncalcified coronary atherosclerotic plaque (NCP) volume over two years as measured by CCTA, as compared with placebo in HIV-infected persons at low to moderate traditional ASCVD risk.

1.2.2 Secondary Hypotheses

- Decreases in LDL cholesterol levels associated with statin therapy will predict improvement in NCP burden and/or plaque vulnerability features.
- Statin therapy will reduce indices of general inflammation, coagulation, monocyte activation, and arterial inflammation.
- Statin therapy will reduce levels of pro-inflammatory monocyte populations.
- Statin therapy will reduce levels of T-cell activation and exhaustion.
- Changes in levels of immune activation and inflammatory markers will be associated with changes in morphology and composition of NCP.
- Statin therapy will not have a clinically significant effect on glucose and insulin resistance.

1.3 A5333S Timeline

- The Substudy opened to enrollment on April 14, 2015; first enrollment occurred on May 6, 2015.
- Substudy enrollment closed on February 6, 2018.
- A memo was sent to Substudy sites on March 12, 2020, requesting completion of all follow-up CT scans by June 1, 2020. The Substudy did not formally close to follow-up at this time, pending a possible extension and additional follow-up CT scan.

Table of Contents

1 BACKGROUND 1
Version History 1
1.1 A5333S Design Overview 2
1.2 A5333S Hypotheses 2
1.2.1 Primary Hypothesis 2
1.2.2 Secondary Hypotheses 2
1.3 A5333S Timeline 2
TABLE OF CONTENTS 3
2 OBJECTIVES AND ESTIMANDS 5
2.1 Primary Objective 5
2.2 Secondary Objectives 5
3 OUTCOME MEASURES 6
3.1 Analysis Windows for Evaluations 6
3.2 Primary, Secondary, and Associated Supportive Outcome Measures 6
3.2.1 Change NCP volume over 2 years (Primary) 6
3.2.2 Progression of NCP over 2 years (Primary) 6
3.2.3 NCP on the 2 year CT (supportive) 6
3.2.4 NCP volume at 2 years (supportive) 7
3.2.5 Total plaque volume 7
3.2.6 Change in relative composition of coronary plaque 7
3.3 Exploratory Plaque Outcomes 7
3.3.1 Number of segments with plaque 7
3.3.2 Number of segments with noncalcified portion 7
3.3.3 Presence of vulnerable plaque features 7
3.3.4 Alternative definition of noncalcified plaque volume, using a threshold of <130 HU 7
3.3.5 Low attenuation plaque volume (<30 HU) 7
3.3.6 Stenosis 8
3.3.7 Progression of NCP over 2 years 8
3.3.8 Coronary artery calcium (CAC), Leaman, and Segment Involvement (SIS) scores 8
3.4 Markers of Immune Function 8
3.5 Markers of Glucose Homeostasis 8
3.6 Descriptive Outcomes 9
3.6.1 Time to the first major cardiovascular event 9
3.6.2 Fasting lipid fractions 9
4 ANALYSIS SETS AND HANDLING OF MISSING DATA 9
4.1 Primary (Intent-to-treat) 9
4.2 Per Protocol (Treated) 9
5 ADMINISTRATIVE DETAILS 9
5.1 Participant Enrollment 9
5.2 Participant Study and Treatment Disposition 10
5.3 CT Scan Availability 10
5.4 Consort 11
5.5 Biomarker Availability 11

6 PARTICIPANT CHARACTERISTICS AND OTHER RISK FACTORS 12

7 ANALYSIS PLAN 13

7.1 General analysis Considerations 13

7.2 Statin effects on coronary plaque morphology 13

7.3 Statin effects on markers of immune function and markers of glucose homeostasis 14

7.4 Statin effects on lipids 14

7.5 LDL and blood biomarkers as mediators for plaque progression 14

APPENDIX I SAMPLE SIZE CONSIDERATIONS 15

2 Objectives and Estimands

2.1 Primary Objective

To determine the effects of pitavastatin on the morphology and composition of coronary atherosclerotic plaque, (including the progression of noncalcified plaque (NCP) volume), and whether these effects are modulated by markers of inflammation and immune activation.

**Primary Estimand**	
Estimand description	*An estimand defines the target of estimation for a particular trial objective (i.e. "what is to be estimated"). This combines all the estimand elements outlined below into a single, succinct sentence.*
Treatment	Treatment groups
Pitavastatin 4mg per day	Pitavastatin 4mg per day or placebo
Target population	Analysis set
PWH on stable ART with low to moderate cardiovascular risk and no known current indication for statin treatment.	All randomized participants with a REPRIEVE (A5332) entry visit. Participants who were consented but never returned to the clinic for their REPRIEVE (A5332) entry visit (regardless of whether the participant was formally randomized) will be considered inadvertent enrollments and excluded.
Variable(s)	Outcome measure(s)
Change in noncalcified plaque volume (NCP)<br><br>*Change in volume of plaque and calcified plaque will be examined as secondary variables.*	*See Section 3 for more detailed outcome descriptions*<br>• Absolute change in NCP volume from entry to year 2.<br>• NCP progression defined as any increase (>0) in NCP volume or incident NCP for those without NCP at entry (based on qualitative read)<br>For sensitivity analyses,<br>• Qualitative assessment of at least one segment with NCP on the 2 year CT.<br>• Quantitative NCP volume at 2 year CT
Handling of intercurrent events	Handling of missing data
<u>Failure to start statin:</u> Included per randomized treatment (treatment policy)<br><u>Change in statin strategy:</u> Included per randomized treatment (treatment policy) *Includes discontinuation or change of statin*<br><u>Evidence of critical stenosis at treatment start:</u> Included per randomized strategy (treatment strategy)<br>Interval stent placement or bypass*: Included per randomized strategy (treatment policy strategy)<br><u>Death prior to Year 2 scan:</u> Treated as missing data (hypothetical policy)	Missing data due to missed visits or unreadable scans will be assumed missing completely at random and ignored i.e., the primary analysis will be a complete case analysis including participants with pair baseline and year 2 scans.<br>Sensitivity analyses will include multiple imputation for missing data.
Population-level summary measure	Analysis approach
Mean change NCP volume over 2 years	Linear regression (normal errors assumption).<br>In the event of extreme violation of normality, outcomes will be log transformed (giving a percentage change interpretation of the treatment effect).<br>Modeling will adjust for CD4 and sex (stratification factors), and for baseline NCP volume.
** Stented or bypassed coronary artery segments are not considered on the entry and year 2 scan as part of the read protocol for plaque volume.*	

2.2 Secondary Objectives

- To determine the effects of pitavastatin on the progression of high risk plaque features including low attenuation plaque and positive remodeling.
- To determine the effects of pitavastatin on detailed markers of immune function including, immune activation (%CD14+CD16+ monocytes, sCD163, sCD14, MCP-1 and T-cell markers), inflammation (Lp-PLA2, hsCRP, hs troponin, oxLDL IL-6), coagulation (D-Dimer) and traditional CVD risk indices

including detailed parameters of glucose homeostasis (insulin, glucose and related indices of insulin resistance such as HOMA-IR).

- To determine the relative contributions of baseline and pitavastatin induced changes in HIV-specific immune activation and traditional risk factors, including LDL, on the presence and progression of coronary plaque and high-risk morphological features in HIV.

3 Outcome Measures

3.1 Analysis Windows for Evaluations

Evaluations drawn within the windows of evaluation defined below will be included in outcome measures; evaluations outside these windows will be excluded. It is noted that analysis windows for month 24 evaluations were widened from those originally planned to accommodate scheduling complications due to COVID restrictions.

<table>
<tr><th>Evaluation</th><th>Entry*</th><th>Month 4</th><th>Year 2 (Month 24)</th></tr>
<tr><td>CT scan</td><td>Any date ≤ (First dose+14 days)</td><td>-</td><td rowspan="5">Target date – 6 months (180 days) to Target date + 12 months (360 days) i.e. Month 18 to Month 36</td></tr>
<tr><td>Fasting lipids</td><td>Any date ≤ First dose</td><td>Target date ± 60 days</td></tr>
<tr><td>Soluble biomarkers</td><td>Any date ≤ First dose</td><td>Target date ± 60 days</td></tr>
<tr><td>Flow cytometry</td><td>Any date ≤ First dose</td><td>-</td></tr>
<tr><td>Questionnaires</td><td>Any date ≤ First dose</td><td>-</td></tr>
<tr><td colspan="4">* For participants who never start treatment, evaluations ≤ 30-days after A5332 entry.
Target date = A5332 entry date + (Month*30)</td></tr>
</table>

3.2 Primary, Secondary, and Associated Supportive Outcome Measures

The primary variable of interest for the Substudy is the morphology and composition of noncalcified coronary atherosclerotic plaque. The following NCP outcome measures will be described and compared by treatment group. Corresponding outcomes at entry will be described when appropriate. For the primary analysis, a threshold of <350 HU will be used to define non-calcified plaque.

3.2.1 Change NCP volume over 2 years (Primary)

- Continuous outcome:
  - Absolute change: NCP volume at 2 years – NCP volume at entry
  - Percentage change: (NCP volume at 2 years – NCP volume at entry) / NCP volume at entry (Supportive)
- Based on quantitative read whenever available (including for participants with no evidence of NCP based on the corresponding qualitative read).
  - Participants without a quantitative read and no evidence of NCP based on the corresponding qualitative read will be assigned a value of zero for both absolute and percentage change.

3.2.2 Progression of NCP over 2 years (Primary)

- Binary outcome (Yes, No)
- Based on both quantitative and qualitative reads
  - Among participants without evidence of NCP at entry on the qualitative read, evidence of NCP at 2 years.
  - Among participants with evidence of NCP at entry on qualitative read, >0% increase in NCP volume
  - Participants with discordant outcomes based on qualitative and quantitative reads, progression will be defined by the quantitative read.

3.2.3 NCP on the 2 year CT (supportive)

- Binary outcome (Yes, No)
- Based on qualitative read
- Outcome is defined regardless of availability of an entry scan

3.2.4 NCP volume at 2 years (supportive)

- Continuous outcome
- Based on quantitative read whenever available (including for participants with no evidence of NCP on the corresponding qualitative read).
  - Participants without a quantitative read and no evidence of NCP on the corresponding qualitative read will be assigned a value of zero.
- Outcome is defined regardless of availability of an entry scan

3.2.5 Total plaque volume

- Analogous outcomes to those in Section 3.2.1-3.2.4 but defined based on total plaque volume (rather than NCP)
- Outcomes will be described for the cohort at entry and year 2, with formal comparisons by treatment group.

3.2.6 Change in relative composition of coronary plaque

- Continuous outcome
- Calculated as the ratio of NCP to overall plaque volume

3.3 Exploratory Plaque Outcomes

The following are additional CT-related outcomes that will be described for the cohort at entry and year 2, as appropriate. *Note: Not all of these analyses will be included in the primary manuscript; but will be included in the packet of primary analyses for completeness.*

3.3.1 Number of segments with plaque

- Ordinal outcome: (0, 1-2, ≥3)
- Based on qualitative read

3.3.2 Number of segments with noncalcified portion

- Ordinal outcome: (0, 1-2, ≥3)
- Based on qualitative read

3.3.3 Presence of vulnerable plaque features

- Five binary (Yes, No) outcomes based on the qualitative read
- Presence of any following vulnerable features (main outcome) and the prevalence of each feature descriptively (four outcomes)
  - Positive remodeling
  - Low attenuation plaque
  - Positive remodeling and low attenuation plaque
  - Napkin ring sign

3.3.4 Alternative definition of noncalcified plaque volume, using a threshold of <130 HU

- Continuous outcome:
  - Absolute change: NCP volume at 2 years – NCP volume at entry
  - Percentage change: (NCP volume at 2 years – NCP volume at entry) / NCP volume at entry (Supportive)
- Based on quantitative read whenever available (including for participants with no evidence of NCP based on the corresponding qualitative read).
  - Participants without a quantitative read and no evidence of NCP based on the corresponding qualitative read will be assigned a value of zero for both absolute and percentage change.

3.3.5 Low attenuation plaque volume (<30 HU)

- Continuous outcome
- Based on quantitative read whenever available.

- Participants without a quantitative read and no evidence of low attenuation plaque on the corresponding qualitative read will be assigned a value of zero

3.3.6 Stenosis

- Three outcomes based on qualitative read
- Presence of CAD or stenosis >0%
  - Binary (Yes, No)
- CAD severity
  - Ordinal (Minimal, Mild, Moderate, Severe)
    - No stenosis
    - Minimal CAD, stenosis 1-24%
    - Mild CAD, stenosis 25%-49%
    - Moderate CAD, stenosis 50%-69%
    - Severe CAD, stenosis ≥70% or ≥50% left main
- Presence of stenosis ≥50%
  - Binary (Yes, No)

3.3.7 Progression of NCP over 2 years

- Ordinal outcome (Progression, Stable, Regression)
- Based on both quantitative and qualitative reads
  - Progression: *Defined per 3.1.4*
  - Regression:
    - Based on quantitative read: >0% decrease in NCP volume over two years.
  - Stable:
    - Based on qualitative read: No evidence of NCP at entry and 2 years.
    - Based on quantitative read: Percentage change in NCP volume between -2% and +2% (including exactly -2% and +2%).
  - For participants with discordant outcomes based on qualitative and quantitative reads, progression will be defined by on the quantitative read.

3.3.8 Coronary artery calcium (CAC), Leaman, and Segment Involvement (SIS) scores

- CAC: Continuous and ordinal (0, 1-10, 11-100, 100-300, >300
- Leaman: Ordinal (0, >0-5, >=5)
- SIS: Ordinal (0, 1, 2, 3, 4, 5+)

3.4 Markers of Immune Function

Levels at entry, 4 months and 2 years, and changes from study entry to 4 months and 2 years (unless stated otherwise).

- Soluble markers of monocyte activation
  - sCD163, sCD14, MCP-1
- Monocyte populations (entry and 2 years only)
  - %CD14+CD16+ (inflammatory/intermediate) monocytes
    - Classical and non-classical subsets presented descriptively
- Markers of T-cell activation and T-cell exhaustion (entry and Year 2 only)
  - CD38+DR+ on CD4+ and CD8+
- Markers of inflammation:
  - Lp-PLA2, hsCRP, IL6, hs troponin, oxLDL
- Markers of coagulation
  - D-Dimer

Future analyses will interrogate the broader spectrum of immune phenotyping that has been performed collected PBMCs.

3.5 Markers of Glucose Homeostasis

Levels at entry, 4 months and 2 years, and changes from study entry to 4 months and 2 years.

- Fasting insulin, glucose, and HOMA-IR

3.6 Descriptive Outcomes

The following REPRIEVE outcome measures will be described by treatment group for the substudy population but since statin effects for these outcomes will be determined based on the full REPRIEVE cohort, formal treatment comparisons will not be performed.

3.6.1 Time to the first major cardiovascular event

- As defined in the REPRIEVE Primary Statistical Analysis Plan

3.6.2 Fasting lipid fractions

- Total, LDL, non-HDL, and HDL cholesterol, and LDL:HDL ratio at study entry, month 4, and 2 years
  - LDL will be reported as calculated LDL if triglycerides ≤400 mg/d; direct LDL if triglycerides >400 mg/dL and <500 mg/dL; LDL will be considered missing if triglycerides ≥500 mg/dL

4 Analysis Sets and Handling of Missing Data

4.1 Primary (Intent-to-treat)

The primary ITT sample will include all registered substudy participants who completed their REPRIEVE (A5332) entry visit. Participants registered to the substudy who failed to complete a REPRIEVE (A5332) entry visit will be considered inadvertent enrollments and excluded from all data summaries and analyses.

The primary analyses will assume all missing data are missing completely at random and be ignored. The intercurrent event of participant death during the 2 year follow-up is expected to be rare and will be handled using a hypothetical strategy – that is, outcomes unavailable due to death will be treated as missing data. Baseline and Year 2 outcomes will be described among all participants with data available at the respective time-point; analyses of change will include only participants with paired scans.

Sensitivity analyses will use multiple imputation with randomization stratification factors (sex and CD4 count) plus ASCVD risk score and BMI used to develop the model.

4.2 Per Protocol (Treated)

All analyses will be repeated within a per protocol sample which will be limited to participants who completed 24–months study follow-up on randomized treatment.

Unless the primary sensitivity analyses demonstrate a concern for informative missingness, per protocol analyses will be limited to participants with paired entry and year 2 scans available with no additional sensitivity analyses performed. Cardiovascular risk factors associated with dropping out of the per protocol analysis will be examined.

5 Administrative Details

Limited to participants in the ITT analysis set.

5.1 Participant Enrollment

Figure: Enrollment over time

- NHLBI enrollment figure

Table: Enrollment by site and sex

- Including details of all participants enrolled at substudy site

5.2 Participant Study and Treatment Disposition

Table: Participant status

	Total	Treatment Group	
		**Pitavastatin (N=XX)**	**Placebo (N=XX)**
	**Total (N=XX)**		
**Enrolled**	**XX (xx%)**	**XX (xx%)**	**XX(xx%)**
**Completed 24 mo follow-up**	**XX (xx%)**	**XX (xx%)**	**XX(xx%)**
Timing of final visit (months from enrollment)) Median (P10-P90)			
Min-Max			
**Did not complete substudy**	**XX (xx%)**	**XX (xx%)**	**XX(xx%)**
Reason for Discontinuation *Reasons from A5333S RP0002*	XX	XX	XX
….	XX	XX	XX
Timing of discontinuation Before 4 month visit*	XX (xx%)	XX (xx%)	XX(xx%)
Between 4 and 24 month visits	XX (xx%)	XX (xx%)	XX(xx%)

Figure: Time to substudy discontinuation

- KM plot by treatment group

Table: Treatment disposition

		**Total (N=XX)**	**Treatment Group: Pitavastatin (N=XX)**	**Treatment Group: Placebo (N=XX)**
Days to start of study treatment*	N	XX	XX	XX
	Median (P10-P90)	XX (XX-XX)	XX (XX-XX)	XX (XX-XX)
	Min-Max	0-X,XXX	0-X,XXX	0-X,XXX
	# missing	XX	XX	XX
	0-2	XX (XX%)	XX (XX%)	XX (XX%)
	3-14	XX (XX%)	XX (XX%)	XX (XX%)
	>14	XX (XX%)	XX (XX%)	XX (XX%)
	Will never start	XX (XX%)	XX (XX%)	XX (XX%)
Timing of treatment discontinuation	Before 4 month visit*	XX (XX%)	XX (XX%)	XX (XX%)
	Between 4 and 24 month visits	XX (XX%)	XX (XX%)	XX (XX%)
	On or after the 24 month visit	XX (XX%)	XX (XX%)	XX (XX%)
Reasons for discontinuation	*Reasons from A5332 RP0003*	XX (XX%)	XX (XX%)	XX (XX%)
	…	XX (XX%)	XX (XX%)	XX (XX%)

** Includes participants who never started treatment*

Figure: Time to treatment discontinuation

- KM plot by treatment group

5.3 CT Scan Availability

Table: CT scan availability

Table denominator is the ITT analysis set; table will be repeated in the per protocol analysis set.

		**Total (N=XX)**	**Treatment Group: Pitavastatin (N=XX)**	**Treatment Group: Placebo (N=XX)**
Evaluable Entry CT scan	Qualitative read	XX (XX%)	XX (XX%)	XX (XX%)
	Quantitative read	XX (XX%)	XX (XX%)	XX (XX%)
	Paired reads	XX (XX%)	XX (XX%)	XX (XX%)
No Entry CT scan scan		XX (XX%)	XX (XX%)	XX (XX%)

			Treatment Group	
		**Total (N=XX)**	**Pitavastatin (N=XX)**	**Placebo (N=XX)**
Reason no entry CT scan	Out of window	XX	XX	XX
	Missed visit	XX	XX	XX
	.<other reasons>	XX	XX	XX
	Image quality	XX	XX	XX
Evaluable Year 2 CT scan	Paired reads	XX (XX%)	XX (XX%)	XX (XX%)
	Qualitative read	XX (XX%)	XX (XX%)	XX (XX%)
	Quantitative read	XX (XX%)	XX (XX%)	XX (XX%)
No Year 2 CT scan		XX (XX%)	XX (XX%)	XX (XX%)
Reason no scan	Out of window	XX	XX	XX
	Premature discontinuation	XX	XX	XX
	Missed visit	XX	XX	XX
	<other reasons>	XX	XX	XX
	Image quality	XX	XX	XX
Evaluable paired scans	Qualitative read	XX (XX%)	XX (XX%)	XX (XX%)
	Quantitative read	XX (XX%)	XX (XX%)	XX (XX%)
	Paired reads	XX (XX%)	XX (XX%)	XX (XX%)

Possible <Other reasons> based on the scan tracking form might include: Refusal, Unable to place IV or other technical issue, Renal insufficiency, Contrast allergy, BMI >40.

5.4 Consort

See Appendix II for draft Consort based on scan reads. This will be expanded on to incorporate details of treatment status and analysis windows.

- Registered
  - Did not complete A5332 entry visit
- Within the ITT sample and by treatment group, Cross-tabulation of
  - Scan availability by treatment status
    - Baseline CTA completed (in versus outside of window)
    - Diagnostics baseline CTA
    - Completed follow-up CTA (in versus outside of window)
    - Diagnostic baseline and follow-up CTA
  - Treatment status
    - Completed treatment
    - Did not start treatment
    - Early treatment discontinuation

5.5 Biomarker Availability

Table: Data availability Like the table below but scaled down out of window visits/samples.

				of Expected Subjects		Total Biomarkers	
**Visit**	*[Visit windows]*		**Subjects Expected**	**Observed Visits**	**Biomarkers available**	**for day**	**for day & Baseline**
**Entry**	***TD-180***	***TD+1***	**XX**	**XX (XX%)**	**XX (XX%)**	**XX**	
Entry +	*TD+2d*	*TD+59d*	XX	XX [XX%]	XX [XX%]		
Month 4-	*TD-60d*	*TD-30d*	XX	XX [XX%]	XX [XX%]		
**Month 4**	***TD±30d***		**XX**	**XX (XX%)**	**XX (XX%)**	**XX**	**XX**
Month 4+	*TD+30d*	*TD+60d*	XX	XX [XX%]	XX [XX%]		
Month 12			XX	XX	XX		
Month 24-	*TD-180d*	*TD-30d*	XX	XX [XX%]	XX [XX%]		
**Month 24**	***TD±30d***		**XX**	**XX (XX%)**	**XX (XX%)**		
Month 24+	*TD+30d*	*TD+360d*	XX	XX [XX%]	XX [XX%]		

			of Expected Subjects		Total Biomarkers	
**Visit**	*[Visit windows]*	**Subjects Expected**	**Observed Visits**	**Biomarkers available**	**for day**	**for day & Baseline**
Month 36+		XX	XX	XX		
TD - Target date						

6 Participant Characteristics and Other Risk Factors

See JAMA Open Table 1. Statistics by treatment group as described will be given in the study report; the summaries will likely be streamlined for the manuscript.

The following defined at study entry only unless stated otherwise. The table denominator would be the ITT sample. A second table of the same information will be included for those with paired scans.

- <u>Demographic and Behavioral characteristics</u>
  - Age (years): Mean (SD) and percent by group (40-49; 50-59; 60+)
  - Sex: Percent women
  - Gender identity: Percent by group (Cisgender, Transgender spectrum, Not reported)
  - Race: Percent by group (White, Black or African American, Asian, Other)
  - Ethnicity: Percent by group (Hispanic or Latino, Not Hispanic or Latino, Unknown)
  - Other substance use (cocaine, heroin, methamphetamine)
- <u>ASCVD Risk Score at REPRIEVE Entry</u>
  - ASCVD risk score (%): Median (Q1, Q3) and percent by group (<2.5, 2.5-<5, 5-<10, 10+)
  - Smoking status: Percent by group (Current, Former, Never)
  - Use of antihypertensive medication: Percent yes
  - Systolic BP of those not on antihypertensive medication: Median (Q1-Q3) (P10, P90)
  - Total cholesterol (mg/dL) : Median (Q1-Q3) (P10, P90)
  - HDL-C (mg/dL) : Median (Q1-Q3) (P10, P90)
  - LDL calculated (mg/dL) : Median (Q1-Q3) (P10, P90)
  - Triglycerides (mg/dL) : Median (Q1-Q3) (P10, P90)
  - Family history of premature CVD: Percent yes
- <u>Other Cardiovascular / metabolic characteristics at REPRIEVE Entry</u>
  - BMI: (kg/m2) Mean (SD) and percent by group (<24.9, 25-29.9, 30+)
  - Prior statin use: Percent yes
    - Based on site report at entry
  - Diabetes: Percent yes
  - Use of anti-diabetic therapy: Percent yes
  - Use of ACE/ARBs: Percent yes
  - Use of anti-platelets/anticoagulants: Percent yes
  - Use of non-statin lipid lowering agents Percent yes
- <u>Cardiovascular / metabolic characteristics at Year 2</u>
  - BMI: (kg/m2) Mean (SD) and percent by group (<24.9, 25-29.9, 30+)
  - Prior statin use: Percent yes
    - Based on site report at entry
  - Diabetes: Percent yes
  - Use of anti-diabetic therapy: Percent yes; percent adding medication after baseline
  - Use of ACE/ARBs: Percent yes; percent adding medication after baseline
  - Use of anti-platelets/anticoagulants: Percent yes; percent adding medication after baseline
  - Use of non-statin lipid lowering agents Percent yes; percent adding medication after baseline
- <u>HIV-related health history (all based on site report at entry)</u>
  - Time since HIV diagnosis (years): Median (Q1, Q3) (P10, P90)
  - Nadir CD4 count (cells/mm3): Percent by group (<50, 50-199, 200-349, ≥350, Unknown)
  - Total ART use duration (years): Percent by group (<5, 5-10, >10, Unknown)
  - Thymidine exposure: Percent yes
  - Abacavir exposure: Percent yes
  - TDF exposure: Percent yes
  - Protease inhibitor exposure: Percent yes

- HIV-related health at REPRIEVE entry
  - CD4 count (cells/mm3): Mean (SD)
  - HIV-1 RNA (copies/ml): Percent by group (<LLQ, LLQ-400, >400)
  - ART regimen: Percent by group (NRTI+INSTI, NRTI+NNRTI, NRTI+PI, NRTI-sparing, Other NRTI containing)
  - Entry NRTI: Percent by group (No NRTI, TDF, TAF, Abacavir, Other)
  - Entry INSTI: Percent by group (No INSTI, DTG, EVG, BIC, RAL)
- HIV-related health at Year 2
  - Most recent CD4 count (cells/mm3): Mean (SD)
  - Most recent HIV-1 RNA (copies/ml): Percent by group (<LLQ, LLQ-400, >400)
  - Change in class of ART from baseline: Percent yes
  - Current ART regimen: Percent by group (NRTI+INSTI, NRTI+NNRTI, NRTI+PI, NRTI-sparing, Other NRTI containing)
  - Change in NRTI from entry: Percent yes.
  - Current NRTI: Percent by group (TDF, TAF, Abacavir, No NRTI, Other)
  - Change in INSTI from entry: Percent yes.
  - Current INSTI: Percent by group (No INSTI, DTG, EVG, BIC, RAL)

7 Analysis Plan

7.1 General analysis Considerations

Outcome distributions will be described with median, Q1, Q3, P10, P90, minimum, and maximum and shown graphically. All primary treatment group comparisons will be performed ITT using a 5% type error. For continuous outcomes, inference will be based on differences in means adjusted for baseline and presented with two-sided, 95% confidence intervals.

7.2 Statin effects on coronary plaque morphology

Outcome measures: See Sections 3.2 and 3.3

Table: Primary and secondary plaque outcomes

Among participants with plaque at entry, descriptive statistics for the change and percentage change in NCP volume over 2 years will be provided by treatment group with group comparisons made with linear regression with adjustment for baseline plaque volume and stratified by sex and CD4.

Among those without NCP at entry, the prevalence of incident NCP over 2 years will be compared with stratified chi-squared test.

To assess the mechanistic study population as a whole, the probability of NCP and total plaque progression over two years (see Section 3.2.2) will be compared by treatment group using a stratified chi-squared test.

The statin effect on high risk plaque features (any), including low attenuation and positive remodeling, will be assessed by comparing differences in the 2-year prevalence of high risk plaque morphology features between treatment groups using chi-squared (or Fisher's exact) tests as appropriate; these analyses will be performed overall and by subgroups defined by the presence of NCP at study entry.

Sample table from Testosterone study, JAMA 2017

REDACTED FOR PUBLIC RELEASE OF THE SAP

Table: Modification of statin effect on changes in NCP and total plaque volume

- By baseline value, sex, age, screening CD4, and duration of suppressive ART

Depending on their baseline prevalence, modification of the statin effect by use of cardiovascular medications may also be explored.

7.3 Statin effects on markers of immune function and markers of glucose homeostasis

Outcome measures: See Sections 3.4 and 3.5

Statin effects on the distributions of blood biomarkers belonging to distinct pathways will be assessed via treatment group comparisons of these respective markers via linear regression with adjustment for baseline and stratified by sex and CD4.

We will examine modification of statin effect on these markers by baseline value, sex, age, screening CD4, duration of suppressive ART, and presence of NCP at study entry.

Figure: Distributions of lipid fractions by treatment group over time

- Violin plots

Figure: Mean and 95% CI lipid fractions by treatment group over time

Table: Median, Q1, Q3 and Mean and 95% CI lipid fractions by treatment group over time

Sample table from Atorvastatin trial Lancet 2015

REDACTED FOR PUBLIC RELEASE OF THE SAP

7.4 Statin effects on lipids

Outcome measures: See Section 3.6.2

Figure: Distributions of lipid fractions by treatment group over time

- Violin plots

Figure: Mean and 95% CI lipid fractions by treatment group over time

Table: Mean and 95% CI lipid fractions by treatment group over time

7.5 LDL and blood biomarkers as mediators for plaque progression

In the event that both statin effects on NCP progression and biomarker changes are apparent, the association between changes in LDL and these biomarkers and NCP progression will be examined using graphical techniques and normal errors and logistic regression (for the subpopulations with and without NCP at entry respectively. A mediating effect of these biological factors will be evaluated by examination of changes in the estimated statin effect on NCP upon adjustment for these biological factors.

Since the hypothesized mechanism is that sustained high levels of immune activation and inflammation precede and contribute to progression of NCP volume and high risk plaque features, these analyses will relate short term changes in these biomarkers (over 4 months) to longer term changes in NCP volume and morphology after two years.

Appendix I Sample Size Considerations

[Copied Verbatim from the REPRIEVE A5333S Protocol, Version 5.0, Section 9.2.]

The target sample size for the Mechanistic Substudy of REPRIEVE (A5333s) is 800 participants that will be approximately equally distributed between the study arms.

This sample size was determined to have high power to detect clinically relevant differences between the two study groups both with respect to plaque progression (among those with plaque at study entry) and rates of incident plaque (among those plaque-free at entry). Specifically, the total sample size of 800 subjects will provide 90% power to detect a 6% difference between the study groups in the percent change in NCP volume over 2 years among those with plaque at entry and 90% power to detect 13 percentage point difference in the probability of plaque development over 2 years. These effect sizes translate to a combined estimated 14 percentage point difference in the probability of NCP progression over two years and are based on the following assumptions:

50% of study participants will have evidence of NCP at study entry [Lo 2010]
A SD of 20% for the percent change over 2 years among participants with evidence of plaque at entry
An annual rate of incident plaque development of 12% among participants without plaque at entry
15% of participants entering the substudy will not be evaluable for study entry or 2 year NCP volume.

Together, the effects of statins acting in these two groups (those with and without evidence of NCP at study entry) will provide for a 7% lower prevalence of NCP after 2-years of statin treatment. Expectations for NCP prevalence and progression both as a whole and according to whether NCP was present at study entry are illustrated in the table below. These estimates are based on a simulation that further assumed the following:

Average volume NCP 250mm3 (SD=200) among participants with NCP at entry.
Average volume NCP 40mm3 (SD=20) at 2 years among participants without NCP at entry without statin treatment.
Average volume NCP 20mm3 (SD=20) at 2 years among participants without NCP at entry with statin treatment.
NCP distributions were also assumed to follow a gamma distribution with size and shape parameters determine to provide the desire mean and standard deviations.

Table 9.2-1: Cells constituting the primary targeted group comparisons are shown in bold.

		At study entry			At two 2 years						
		NCP (%)	NCP volume (mm3)		NCP (%)	NCP volume (mm3)		Change in NCP volume (mm3)		Percent change in NCP volume (%)[2]	
			Mean (SD)	[P5, P95]		Mean (SD)	[P5, P95]	Mean (SD)	[P5, P95]	Mean (SD)	[P5, P95]
Control	Overall[1]	50%	121 (186)	[0, 509]	59%	140 (210)	[0, 573]	19 (51)	[-21, 103]	17% (33%)	[-10%, 100%]
	No plaque at entry		-	-	**21%**	9 (19)	[0, 51]	9 (19)	[0, 51]	21% (41%)	[0%, 100%]
	No plaque at 2 years					-	-	-	-	-	-
	Plaque at 2 years					40 (20)	[17, 79]	40 (20)	[17, 79]	100% (0%)	[100%, 100%]
	Plaque at entry		251 (198)	[26, 637]		281 (231)	[28, 730]	30 (68)	[-38, 153]	**12% (20%)**	**[-14%, 49%]**
Statin	Overall[1]	50%	120 (185)	[0, 507]	52%	127 (198)	[0, 536]	7 (45)	[-42, 76]	7% (24%)	[-17%, 60%]
	No plaque at entry		-	-	**8%**	2 (8)	[0, 14]	2 (8)	[0, 14]	8% (27%)	[0%, 100%]
	No plaque at 2 years					-	-	-	-	-	-
	Plaque at 2 years					23 (19)	[2,60]	23 (19)	[2,60]	100% (0%)	[100%, 100%]
	Plaque at entry		250 (197)	[26, 633]		262 (216)	[26, 682]	12 (64)	[-66, 123]	**5% (20%)**	**[-21%, 43%]**

1 *In estimation of overall means, participants without evidence of NCP are assigned a value of 0 for volume and change.*

2 *In estimation of percentage change, participants without evidence of NCP at study entry with NCP at 2 years are assigned a value of 100%.*

These desired statin effects on NCP volume are similar to those seen in intravascular ultrasound (IVUS) studies [Nissen 2006; Nicholls 2005]. Although no data exist to directly inform the clinical relevance of these differences, randomized comparisons of high vs. low dose statin therapy among non-HIV-infected patients have demonstrated favorable effects on atherosclerotic plaque of a similar magnitude as well as on adverse cardiac events, among non-HIV patients [Nissen 2006]. Further, data from the "Coronary CT Angiography Evaluation for Clinical Outcomes (CONFIRM) registry suggest that the presence of non-obstructive CAD is predictive major adverse cardiac events independent of traditional risk factors, degree of stenosis and coronary artery calcification (HR 2-5) [Hulten 2013]. The proposed mechanistic study will further add to this body of evidence investigating, for the first time in HIV-infected population, the association of NCP (both the presence and magnitude) as well as changes in volume relate to events. For this exploratory analysis, a larger sample size will be beneficial, in order to accrue CVD events to relate to plaque morphology.

References

Hulten E, et al. Usefulness of coronary computed tomography angiography to predict mortality and myocardial infarction among Caucasian, African and East Asian ethnicities (from the CONFIRM [Coronary CT Angiography Evaluation for Clinical Outcomes: An International Multicenter] Registry). Am J Cardiol 2013;111(4):479-85. PMID: 23211358.

Lo J, et al. Increased epicardial adipose tissue volume in HIV-infected men and relationships to body composition and metabolic parameters. AIDS 2010;24(13):2127-30. PMID: 20588167.

Nicholls SJ, et al. Effect of atorvastatin (80 mg/day) versus pravastatin (40 mg/day) on arterial remodeling at coronary branch points (from the REVERSAL study). Am J Cardiol 2005;96(12):1636-9. PMID: 16360349.

Nissen SE, et al. ASTEROID Investigators. Effect of very high-intensity statin therapy on regression of coronary atherosclerosis: the ASTEROID trial. JAMA 2006;295(13):1556-65. PMID: 16533939.
