# Supplementary material for: Effects of Pitavastatin on Coronary Artery Disease and Inflammatory Biomarkers in HIV: Mechanistic Substudy of the REPRIEVE Randomized Clinical Trial
Source: JAMA Cardiol. 2024 Feb 21;9(4):323–34. doi: 10.1001/jamacardio.2023.5661 (PMC10882511; doi:10.1001/jamacardio.2023.5661)
Supplement: Supplement 2. — eAppendix. List of REPRIEVE Mechanistic Substudy Site Investigators eMethods. eTable 1. Characteristics of Enrolled REPRIEVE Mechanistic Substudy and Overall REPRIEVE Trial Participants at Baseline* eTable 2. Baseline Characteristics of Participants Enrolled and With at Least 1 Evaluable Scan by Treatment Arm eTable 3. Sensitivity Analysis: Multiple Imputation of Changes in Coronary Artery Plaque by Treatment Arm eTable 4. Sensitivity Analysis: Per-Protocol Changes in Coronary Artery Plaque by Treatment Arm eTable 5. Coronary Artery Plaque Changes by Treatment Arm, Secondary Analysis in Subgroup With Plaque at Entry and Year 2 CT Available for Paired Analysis eTable 6. Distributions of Fasting Lipids (Entry, Month 4, and Month 24) eTable 7. Distributions of Inflammatory and Immune Biomarkers (Month 4) eFigure 1. Example Baseline and 2-Year Follow-Up Coronary CTA Demonstrating Plaque Progression on Placebo (Top Row) and Plaque Regression on Pitavastatin (Bottom Row) eFigure 2. Effect of Individual Biomarkers on Change in Noncalcified Plaque Volume eFigure 3. Effect of Treatment on Noncalcified Plaque Volume, Adjusted for Change in Individual Biomarkers eFigure 4. Effect of Treatment on Noncalcified Plaque Progression, Adjusted for Change in Individual Biomarkers eReferences. [file jamacardiol-e235661-s002.pdf]

Supplemental Online Content

Lu MT, Ribaudo H, Foldyna B, et al; REPRIEVE Trial Writing Group. Effects of pitavastatin on coronary artery disease and inflammatory biomarkers in HIV: mechanistic substudy of the REPRIEVE randomized clinical trial. *JAMA Cardiol*. Published online February 21, 2024. doi:10.1001/jamacardio.2023.5661

eAppendix. List of REPRIEVE Mechanistic Substudy Site Investigators

eMethods.

eTable 1. Characteristics of Enrolled REPRIEVE Mechanistic Substudy and Overall REPRIEVE Trial Participants at Baseline*

eTable 2. Baseline Characteristics of Participants Enrolled and With at Least 1 Evaluable Scan by Treatment Arm

eTable 3. Sensitivity Analysis: Multiple Imputation of Changes in Coronary Artery Plaque by Treatment Arm

eTable 4. Sensitivity Analysis: Per-Protocol Changes in Coronary Artery Plaque by Treatment Arm

eTable 5. Coronary Artery Plaque Changes by Treatment Arm, Secondary Analysis in Subgroup With Plaque at Entry and Year 2 CT Available for Paired Analysis

eTable 6. Distributions of Fasting Lipids (Entry, Month 4, and Month 24)

eTable 7. Distributions of Inflammatory and Immune Biomarkers (Month 4)

eFigure 1. Example Baseline and 2-Year Follow-Up Coronary CTA Demonstrating Plaque Progression on Placebo (Top Row) and Plaque Regression on Pitavastatin (Bottom Row)

eFigure 2. Effect of Individual Biomarkers on Change in Noncalcified Plaque Volume

eFigure 3. Effect of Treatment on Noncalcified Plaque Volume, Adjusted for Change in Individual Biomarkers

eFigure 4. Effect of Treatment on Noncalcified Plaque Progression, Adjusted for Change in Individual Biomarkers

eReferences.

This supplementary material has been provided by the authors to give readers additional information about their work.

© 2024 Lu MT et al. *JAMA Cardiology*.

eAppendix. List of REPRIEVE Mechanistic Substudy Site Investigators

The following investigators participated in the REPRIEVE Mechanistic Substudy, listed by clinical site with numbers of participants enrolled:

**CRS Name (n)**	**CRS Leader**
**Mechanistic Substudy (n=805)**	
Cincinnati CRS (n=58)	Dr. Carl J. Fichtenbaum
Mount Sinai Clinical and Translational Research Center CRS (n=51)	Dr. Judith A. Aberg
Harbor UCLA CRS (n=49)	Dr. Eric S Daar
Northwestern University CRS (n=44)	Dr. Babafemi Taiwo
Ohio State University CRS (n=44)	Dr. Susan L. Koletar
UCLA CARE Center CRS (n=43)	Dr. Kara W. Chew
UCSD Antiviral Research Center CRS (n=43)	Dr. Susan J. Little
Alabama CRS (n=38)	Dr. Sonya L. Heath
Case CRS (n=36)	Dr. Jeffrey M. Jacobson
Massachusetts General Hospital CRS (MGH) (n=36)	Dr. Rajesh Gandhi, Dr. Gregory Robbins
Washington University Therapeutics (WT) CRS (n=35)	Dr. Rachel M. Presti
Weill Cornell Uptown CRS (n=35)	Dr. Marshall Jay Glesby
UCSF HIV/AIDS CRS (n=33)	Dr. Annie Luetkemeyer
Penn Therapeutics CRS (n=32)	Dr. Pablo Tebas
University of Pittsburgh CRS (n=26)	Dr. Sharon A. Riddler
University of Southern California CRS (n=24)	Dr. Michael P. Dube
Puerto Rico AIDS Clinical Trials Unit CRS (n=22)	Dr. Jorge L. Santana-Bagur
Mount Sinai Clinical and Translational Research Center CRS (n=20)	Dr. Judith A. Aberg
Rush University CRS (n=18)	Dr. Beverly E. Sha
Brigham and Women's Hospital Therapeutics (BWH TCRS) CRS (n=16)	Dr. Jennifer Manne
Houston AIDS Research Team CRS (n=16)	Dr. Roberto Arduino
Johns Hopkins University CRS (n=15)	Dr. Charles W. Flexner
Vanderbilt Therapeutics (VT) CRS (n=15)	Dr. David W. Haas
Chapel Hill CRS (n=14)	Dr. David Alain Wohl
Columbia Physicians & Surgeons (P&S) CRS (n=12)	Dr. Magdalena E. Sobieszczyk
The Miriam Hospital (TMH) CRS (n=12)	Dr. Karen T. Tashima
University of Rochester Adult HIV Therapeutic Strategies Network CRS (n=11)	Dr. Sonal S. Munsiff
University of Washington AIDS CRS (n=9)	Dr. Rachel Bender Ignacio
Weill Cornell Chelsea CRS (n=6)	Dr. Kristen Marks
Greensboro CRS (n=5)	Dr. Cornelius Van Dam
New Jersey Medical School Clinical Research Center CRS (n=5)	Dr. Shobha Swaminathan
University of Colorado Hospital CRS (n=2)	Dr. Thomas B. Campbell

© 2024 Lu MT et al. *JAMA Cardiology*.

REPRIEVE Trial Leadership and Stakeholders

The study investigators thank the study participants, site staff, and study-associated personnel for their ongoing participation in the trial. In addition, we thank the following: the AIDS Clinical Trial Group (ACTG) for clinical site support; ACTG Clinical Trials Specialists for regulatory support; the data management center, Frontier Science Foundation, for data support; the Center for Biostatistics in AIDS Research for statistical support; and the Community Advisory Board for input for the community and NHLBI and NIAID team members for help with the trial.

In addition to the writing group, the following committee members contributed to the conduct of the REPRIEVE trial:

AIDS Clinical Trials Network (ACTG): B. Bastow, J. Dragavon, E. Hoffman. F. Giguel, L. Moran (ACTG Network Coordinating Center, DLH Corporation), J. Roa. (ACTG Network Coordinating Center, DLH Corporation), N. Saleh (ACTG Network Coordinating Center, DLH Corporation), A. Shahkolahi.

Biomedical Research Institute (biorepository): J. Ward.

Canadian Trials Network: E. Cherban, D. Nohynek, J. Falutz.

Center for Biostatistics and AIDS Research at Harvard T.H. Chan School of Public Health: S. Brummel, J. Granche, A. Kantor, J. Leon-Cruz, C. Moser, P. Pascuski, H.J. Ribaudo, L. Smeaton, T. Umbleja.

Clinical Coordinating Center: C. Benjamin, G. Bloomfield, T. Cadet, M. Diggs, P. Douglas, E. Fulda, S. Grinspoon, E. Grzejka, S. Looby, S. McCallum, J. Murphy, M. Zanni.

Community Advisory Board: A. Diggs, R. Ettinger, A. Hernandez, J. Jarrells, S. Selvage.

Community Scientific Subcommittee Representatives: K. Shaw, K. Starr.

Data Coordinating Center: R. Barve, B. Foldyna, S. Hedgire, U. Hoffmann, J. Karády, N. Klop-Packel, Y. Liao, M. Lu, T. Mayrhofer, N. Meyersohn, E. Norton, K. Paradis, A. Sturniolo, J. Taron.

Data Management Center (Frontier Science Foundation): F. Bone, M. Byroads, M. Green, H. Gutzman, A. Holguin, R. LeBlanc, J. Nowak, G. Pavlov, S. Siminski, H. Sprenger, D. Vlieg, K. Wood.

External Advisory Board: S. Hammer (late member), M. Hirsch, J. Manson, P. Ridker, J. Stein, R. Tracy, J. Udelson.

The European treatment network for HIV, hepatitis and global infectious diseases (NEATid): E. Martinez, T. Leaver, A. Pozniak.

Gilead Sciences, Inc.: K Melbourne, J. Rooney

Independent Data and Safety Monitoring Board: M. Budoff, B. Cheng, S. Goldkind, C. Grunfeld, R. Harrington (Chair), D. Lloyd-Jones, J. Robinson, L. Sleeper, G. Sopko (Executive Secretary), P. Volberding.

Kowa Pharmaceuticals America, Inc.: C. Sponseller

National Institutes of Health/National Heart Lung and Blood Institute: P. Bandettini, P. Desvigne-Nickens, F. Ketema, G. Sopko, Y. Rosenberg, J. Troendle.

National Institutes of Health/National Institute of Allergy and Infectious Diseases: B. Alston, K. Klingman, P. Kim, O. Alli, K. Johnson, K. Reese, M. Mishkin, B. Castillo, B. Adedeji, D. Livnat, A. Ojumu

Research Organisation (kc) ltd. (CRO for EU5332): O. Anthony, S. Bannoo, A. Duffy, C. Fletcher, M. Sanchez Grande, A. Sierto, E. Walker.

REPRIEVE Committee Membership

© 2024 Lu MT et al. *JAMA Cardiology*.

Executive Committee	
**Member Name**	**Institution**
B. Alston-Smith	National Institute of Allergy and Infectious Diseases of the National Institutes of Health
P. Bandettini	National Heart, Lung, and Blood Institute of the National Institutes of Health
G. Bloomfield	Duke University
J. Currier	University of California Los Angeles David Geffen School of Medicine
P. Desvigne-Nickens	National Heart, Lung, and Blood Institute of the National Institutes of Health
M. Diggs	Massachusetts General Hospital
P. Douglas	Duke University
K. Fitch	Massachusetts General Hospital
S. Grinspoon (Chair)	Massachusetts General Hospital
P. Kim	Division of AIDS of the National Institutes of Health
M. Lu	Massachusetts General Hospital
K. Paradis	Massachusetts General Hospital
H. Ribaudo	Harvard T.H. Chan School of Public Health
Y. Rosenberg	National Heart, Lung, and Blood Institute of the National Institutes of Health
J. Troendle	National Heart, Lung, and Blood Institute of the National Institutes of Health
**Events Oversight Committee**	
G. Bloomfield	Duke University
M. Byroads	Frontier Science Foundation
P. Douglas (Chair)	Duke University
K. Fitch	Massachusetts General Hospital
E. Gershman	Brigham and Women's Hospital
S. Grinspoon	Massachusetts General Hospital
F. Lawal	Division of AIDS of the National Institutes of Health
J. Leon-Cruz	Harvard T.H. Chan School of Public Health
R. Louis	Massachusetts General Hospital
C. Lowe	Brigham and Women's Hospital
M. Lu	Massachusetts General Hospital
E. Moy	Brigham and Women's Hospital
K. Paradis	Massachusetts General Hospital
H. Ribaudo	Harvard T.H. Chan School of Public Health
T. Umbleja	Harvard T.H. Chan School of Public Health
N. Upadhyay	Massachusetts General Hospital
S. Wiviott	Brigham and Women's Hospital
K. Wood	Frontier Science Foundation
**EU Committee**	
O. Anthony	ROKC
R. Barve	Massachusetts General Hospital

© 2024 Lu MT et al. *JAMA Cardiology*.

F. Bone	Frontier Science Foundation
S. Bannoo	ROKC
M. Diggs	Massachusetts General Hospital
A. Duffy	ROKC
K. Fitch (Chair)	Massachusetts General Hospital
C. Fletcher	ROKC
M. Green	Frontier Science Foundation
N. Klop-Packel	Massachusetts General Hospital
S. McCallum	Massachusetts General Hospital
E. Norton	Massachusetts General Hospital
J. Nowak	Frontier Science Foundation
K. Paradis	Massachusetts General Hospital
H. Ribaudo	Harvard T.H. Chan School of Public Health
M. Sanchez Grande	ROKC
A. Sierto	ROKC
S. Siminski	Frontier Science Foundation
T. Umbleja	Harvard T.H. Chan School of Public Health
E. Walker	ROKC
K. Wood	Frontier Science Foundation
D. Vlieg	Frontier Science Foundation
**Lab Committee**	
F. Bone	Frontier Science Foundation
T. Burdo	Temple University
M. Diggs	Massachusetts General Hospital
K. Fitch (Chair)	Massachusetts General Hospital
M. Green	Frontier Science Foundation
S. McCallum	Massachusetts General Hospital
L. Moran	AIDS Clinical Trials Group
K. Paradis	Massachusetts General Hospital
J. Roa	DLH Corporation
H. Sprenger	Frontier Science Foundation
D. Vlieg	Frontier Science Foundation
**Operational Leadership Committee**	
J. Aberg	Icahn School of Medicine at Mount Sinai
B. Adedeji	Division of AIDS of the National Institutes of Health
O. Alli	Division of AIDS of the National Institutes of Health
P. Bandettini	National Heart, Lung, and Blood Institute of the National Institutes of Health
G. Bloomfield	Duke University
T. Burdo	Temple University
M. Byroads	Frontier Science Foundation
B. Castillo	Division of AIDS of the National Institutes of Health

J. Currier	University of California Los Angeles David Geffen School of Medicine
P. Desvigne-Nickens	National Heart, Lung, and Blood Institute of the National Institutes of Health
M. Diggs	Massachusetts General Hospital
P. Douglas	Duke University
J. Dragavon	University of Washington
K. Easley	Division of AIDS of the National Institutes of Health
J. Falutz	Canadian HIV Trials Network
C. Fichtenbaum	University of Cincinnati College of Medicine
K. Fitch	Massachusetts General Hospital
S. Grinspoon (Chair)	Massachusetts General Hospital
E. Grzejka	Massachusetts General Hospital
E. Hoffman	University of North Carolina Chapel Hill
P. Kim	Division of AIDS of the National Institutes of Health
F. Lawal	Division of AIDS of the National Institutes of Health
Y. Liao	Massachusetts General Hospital
S. Looby	Massachusetts General Hospital
M. Lu	Massachusetts General Hospital
L. Moran	AIDS Clinical Trials Group
D. Nohynek	Canadian HIV Trials Network
K. Paradis	Massachusetts General Hospital
M. Pate	Kowa Pharmaceuticals America, Inc.
H. Ribaudo	Harvard T.H. Chan School of Public Health
J. Rooney	Gilead Sciences, Inc.
A. Shahkolahi	AIDS Clinical Trials Group
S. Siminski	Frontier Science Foundation
C. Sponseller	Kowa Pharmaceuticals America, Inc.
H. Sprenger	Frontier Science Foundation
K. Williams	Frontier Science Foundation
K. Wood	Frontier Science Foundation
M. Zanni	Massachusetts General Hospital
**Protocol Writing, Training, and Implementation Committee**	
J. Aberg	Icahn School of Medicine at Mount Sinai
O. Alli	Division of AIDS of the National Institutes of Health
P. Bandettini	National Heart, Lung, and Blood Institute of the National Institutes of Health
G. Bloomfield	Duke University
K. Borloglou	AIDS Clinical Trials Group
M. Byroads	Frontier Science Foundation
M. Clement	Duke University
P. Desvigne-Nickens	National Heart, Lung, and Blood Institute of the National Institutes of Health
M. Diggs	Massachusetts General Hospital

© 2024 Lu MT et al. *JAMA Cardiology*.

P. Douglas	Duke University
J. Dragavon	University of Washington
K. Easley	Division of AIDS of the National Institutes of Health
A. Eckard	Medical University of South Carolina
C. Fichtenbaum	University of Cincinnati College of Medicine
K. Fitch	Massachusetts General Hospital
S. Grinspoon	Massachusetts General Hospital
E. Hoffman	University of North Carolina Chapel Hill
F. Lawal	Division of AIDS of the National Institutes of Health
R. LeBlanc	Frontier Science Foundation
J. Leon-Cruz	Harvard T.H. Chan School of Public Health
M. Lu	Massachusetts General Hospital
C. Malvestutto (Chair)	Ohio State University Medical Center
L. Moran	AIDS Clinical Trials Group
E. Overton (former Chair)	University of Alabama at Birmingham
H. Ribaudo	Harvard T.H. Chan School of Public Health
A. Shahkolahi	AIDS Clinical Trials Group
K. Shaw	ACTG Community Scientific Subcommittee
V. Triant	Massachusetts General Hospital
K. Wood	Frontier Science Foundation
M. Zanni (Chair)	Massachusetts General Hospital
**Publications Committee**	
J. Aberg	Icahn School of Medicine at Mount Sinai
B. Alston-Smith	National Institute of Allergy and Infectious Diseases of the National Institutes of Health
P. Bandettini	National Heart, Lung, and Blood Institute of the National Institutes of Health
G. Bloomfield	Duke University
J. Currier	University of California Los Angeles David Geffen School of Medicine
P. Desvigne-Nickens	National Heart, Lung, and Blood Institute of the National Institutes of Health
M. Diggs	Massachusetts General Hospital
A. Diggs	REPRIEVE Community Advisory Board
P. Douglas	Duke University
C. Fichtenbaum	University of Cincinnati College of Medicine
K. Fitch	Massachusetts General Hospital
S. Grinspoon (Chair)	Massachusetts General Hospital
A. Kantor	Harvard T.H. Chan School of Public Health
F. Lawal	Division of AIDS of the National Institutes of Health
J. Leon-Cruz	Harvard T.H. Chan School of Public Health
S. Looby	Massachusetts General Hospital
M. Lu	Massachusetts General Hospital
C. Malvestutto	Ohio State University Medical Center

© 2024 Lu MT et al. *JAMA Cardiology*.

J. Manne-Goehler	Brigham and Women's Hospital
S. McCallum	Massachusetts General Hospital
L. Moran	AIDS Clinical Trials Group
K. Paradis	Massachusetts General Hospital
H. Ribaudo	Harvard T.H. Chan School of Public Health
J. Rooney	Gilead Sciences, Inc.
C. Sponseller	Kowa Pharmaceuticals America, Inc.
K. Starr	ACTG Community Scientific Subcommittee
T. Umbleja	Harvard T.H. Chan School of Public Health
M. Waclawiw	National Heart, Lung, and Blood Institute of the National Institutes of Health
M. Zanni (Chair)	Massachusetts General Hospital
**Site Selection, Performance, and Close Out Committee**	
J. Aberg	Icahn School of Medicine at Mount Sinai
B. Adedeji	Division of AIDS of the National Institutes of Health
O. Alli	Division of AIDS of the National Institutes of Health
P. Bandettini	National Heart, Lung, and Blood Institute of the National Institutes of Health
R. Barnett	Division of AIDS of the National Institutes of Health
J. Baum	Case Western Reserve
M. Byroads	Frontier Science Foundation
B. Castillo	Division of AIDS of the National Institutes of Health
C. Coates	Division of AIDS of the National Institutes of Health
S. Cordoso	Instituto de Pesquisa Clinica Evandro Chagas
C. Costanza	New Jersey Medical School
S. Davila	Puerto Rico AIDS Clinical Trials Unit
P. Desvigne-Nickens	National Heart, Lung, and Blood Institute of the National Institutes of Health
M. Diggs	Massachusetts General Hospital
J. Dushyantha	Jackson Memorial Hospital
K. Easley	Division of AIDS of the National Institutes of Health
C. Fichtenbaum (Chair)	University of Cincinnati College of Medicine
K. Fitch	Massachusetts General Hospital
T. Greenfield	National Institute of Allergy and Infectious Diseases of the National Institutes of Health
H. Gutzman	Frontier Science Foundation
R. Harden	University of Illinois at Chicago Project Wish
S. Henn	Whitman-Walker Institute, Inc.
M. Humphries	Division of AIDS of the National Institutes of Health
M. Jain	University of Texas Southwestern
D. Klein	Whitman-Walker Institute, Inc.
S. Kohrs	University of Cincinnati College of Medicine
S. Koletar	Ohio State University Medical Center

© 2024 Lu MT et al. *JAMA Cardiology*.

J. Lama	Barranco Clinical Research Site
J. Landis	Division of AIDS of the National Institutes of Health
J. Leone	University of North Carolina Chapel Hill
R. Lira	Hospital Nossa Senhora da Conceicao
M. Lu	Massachusetts General Hospital
C. Malvestutto	Ohio State University Medical Center
M. Martinez	University of Texas Houston
R. Novak	University of Illinois at Chicago Project Wish
K. Reese	Division of AIDS of the National Institutes of Health
G. Robbins	Massachusetts General Hospital
B. Santos	Hospital Nossa Senhora da Conceicao
A. Shahkolahi	AIDS Clinical Trials Group
H. Sprenger	Frontier Science Foundation
J. Tucker	Division of AIDS of the National Institutes of Health
A. Wilkin	Wake Forest University
T. Wilson	University of Alabama at Birmingham
K. Wood	Frontier Science Foundation
M. Zanni	Massachusetts General Hospital
**Mechanistic Substudy CT Committee**	
M. Diggs	Massachusetts General Hospital
P. Douglas	Duke University
K. Fitch	Massachusetts General Hospital
B. Foldyna	Massachusetts General Hospital
S. Grinspoon	Massachusetts General Hospital
J. Karady	Massachusetts General Hospital
M. Lu (Chair)	Massachusetts General Hospital
T. Mayrhofer	Massachusetts General Hospital
S. McCallum	Massachusetts General Hospital
K. Paradis	Massachusetts General Hospital
H. Ribaudo	Harvard T.H. Chan School of Public Health
A. Sturniolo	Massachusetts General Hospital
T. Umbleja	Harvard T.H. Chan School of Public Health

© 2024 Lu MT et al. *JAMA Cardiology*.

Contributions to Design, Conduct and Reporting of REPRIEVE

REPRIEVE (A5332) and the Mechanistic Substudy (A5333s) were funded by the National Heart Lung and Blood Institute (NHLBI) of the NIH through cooperative UO1 and UG3/U24 grants for the Clinical (CCC) and Data (DCC) Coordinating Centers with additional support from the National Institutes of Allergy and Infectious Diseases (NIAID) and supplemental funding from the Office of AIDS Research. Kowa Pharmaceuticals America, Inc., Gilead Sciences and ViiV Healthcare also supported the study. NIAID was the regulatory sponsor and IND holder.

REPRIEVE (A5332) and the Mechanistic Substudy (A5333s) were designed by the Co-PIs (Drs. Grinspoon and Douglas, Co-Chairs of the CCC and Drs. Hoffmann and Ribaudo, Co-Chairs of the DCC) with guidance and approval from the NHLBI and NIAID. Dr. Lu replaced Dr. Hoffmann as Co-PI and Co-Chair of the DCC in 2021. The Executive Committee provided the overall leadership of the trial, Chaired by Dr. Grinspoon, with members including the other Co-PIs and representatives of the NIH. Guidance regarding protocol development and implementation was provided from the Protocol Committee and site performance evaluation overseen by the Site Selection, Performance and Close Out Committee (SSPCC).

The study protocol was approved by the Mass General Brigham IRB, the AIDS Clinical Trials Group, and participating site regulatory bodies. Informed consent was obtained in writing from each participant prior to participation in the study. Oversight was accomplished via an independent Data Safety and Monitoring Board. Coronary CT angiography (CTA) was coordinated by the REPRIEVE CT core laboratory at Massachusetts General Hospital. Data collection was overseen by Frontier Science Research Foundation (FSTRF) and monitored by the NIH Division of AIDS. Statistical analysis was accomplished according to the prespecified analysis plan by the Center for Biostatistics in AIDS Research (CBAR),

A DSMB appointed by NHLBI met every 6 months to review safety and efficacy data and advised the NIH on the status of the study. DSMB meetings and calls were organized into Open, Closed, and Executive Sessions. The open session dealt with issues relating to the general conduct and progress of the study, such as accrual, retention, and safety and all data were presented pooled over treatment group. Following the Open Session and before the Closed Session, a restricted group including only the DSMB, the REPRIEVE NIH team, the REPRIEVE CCC PIs and statisticians reviewed aggregate primary events pooled across treatment groups to discuss trial feasibility and possible sample size adjustments. During the closed session, the DSMB, NHLBI Executive Secretary (ES) and NHLBI statistician, and unblinded REPRIEVE statisticians reviewed data presented by unmasked treatment group. Efficacy data by treatment group were not reviewed until adequacy of the study sample size assumptions was determined. The DSMB closed the meeting in executive session in which only the DSMB members and NHLBI ES were present. A DSMB Charter is available on request.

Study operations were conducted by the Operational Leadership Committee and various governance committees as described below. Data management was conducted by Frontier Science Foundation, Inc. and site monitoring by the NIH Division of AIDS (DAIDS). The REPRIEVE trial leadership remained blinded until March 31, 2023 following acceptance of the DSMB recommendation by the NIH. The manuscript was written by Drs. Michael Lu and Steven Grinspoon with a writing group named as authors on the manuscript. The writing team had full access to trial data, prepared the first draft of the manuscript, and with the REPRIEVE Executive and Publications Committees, made the decision to submit the manuscript.

Blinding Status of the REPRIEVE Biostatistics Team

Blinded: Heather Ribaudo (Lead Statistician, DCC Co-PI), Sara McCallum (Statistical Programmer), Janeway Granche (Statistician)

Unblinded: Triin Umbleja (Coordinating Statistician), Jorge Leon-Cruz (Protocol Statistician), Amy Kantor (Statistician), Sean Brummel (Internal Review), Carlee Moser (Internal Review), Laura Smeaton (Internal Review)

© 2024 Lu MT et al. *JAMA Cardiology*.

Blinded members of the Biostatistics team had no access to study data linked or aggregated by treatment group assignment. All team members were unblinded on March 31, 2023 following the DSMB recommendation to stop the trial.

Funding Statement

This study is supported through NIH grants U01HL123336, U01HL123339, UM1 AI068636, UM1 AI106701, 1UG3HL164285, and 1U24HL164284 as well as funding from Kowa Pharmaceuticals America, Inc, Gilead Sciences, and ViiV Healthcare.

NIH Grants Policy Statement

The views expressed in this manuscript are those of the authors and do not necessarily represent the views of the National Heart, Lung, and Blood Institute or the National Institute of Allergy and Infectious Diseases; the National Institutes of Health; or the U.S. Department of Health and Human Services.

© 2024 Lu MT et al. *JAMA Cardiology*.

eMethods.

Abridged inclusion and exclusion criteria for the REPRIEVE Mechanistic Substudy (A5333s) and the REPRIEVE trial (A5332) are listed below, with the full version provided in the protocol.

Eligibility Criteria

REPRIEVE Mechanistic Substudy (A5333s) Inclusion Criteria:

- Enrollment in the main REPRIEVE (A5532) study.
- Willingness to complete procedures required for the study.
- Signed informed consent.
- Glomerular filtration rate (GFR) ≥60 mL/min/1.73m^2 or creatinine clearance (CrCl) ≥60 mL/min.

REPRIEVE Mechanistic Substudy (A5333s) Exclusion Criteria:

- Known allergy to iodinated contrast agent.
- Currently symptomatic asthma.
- Allergy to beta blockers.
- Contraindication to beta blockers (i.e., taking daily asthma medications).
- Positive pregnancy test within 24 hours prior to study entry.
- Any condition that prohibits the individual from completing the CCTA.
- Body mass index (BMI) ≥40 kg/m2.
- Cardiac arrythmia at enrollment precluding CCTA, such as atrial fibrillation with heart rate >80 beats per minute or frequent ectopic beats.

REPRIEVE (A5332) Inclusion Criteria:

- Documentation of HIV-1 infection by means of any <u>one</u> of the following:
  - Documentation of HIV diagnosis in the medical record by a licensed health care provider;
  - OR HIV-1 RNA detection by a licensed HIV-1 RNA assay demonstrating >1000 RNA copies/mL;
  - OR any licensed HIV screening antibody and/or HIV antibody/antigen combination assay confirmed by a second licensed HIV assay such as a HIV-1 Western blot confirmation or HIV rapid Multispot antibody differentiation assay.
- Combination antiretroviral therapy (ART) for at least 180 days prior to study entry.
- CD4+ cell count >100 cells/mm^3 obtained within 180 days prior to study entry.
- Fasting LDL cholesterol as follows:
  - If ASCVD risk score <7.5%, LDL cholesterol must be <190 mg/dL
  - If ASCVD risk score ≥7.5% and ≤10%, LDL must be <160 mg/dL
  - If ASCVD risk score >10% and ≤15%, LDL must be <130 mg/dL
  - If LDL <70 mg/dL, participant is eligible regardless of 10-year ASCVD risk score in line with the ACC/AHA 2013 Prevention Guidelines.
- Fasting triglycerides <500 mg/dL.
- Hemoglobin >8 g/dL for female participants and >9 g/dL for male participants
- Glomerular filtration rate (GFR) ≥60 mL/min/1.73m^2 or creatinine clearance (CrCl) ≥60 mL/min.
- ALT ≤2.5 x ULN.
- For persons with known chronic active hepatitis B or C, calculated FIB-4 score must be ≤3.25.
- Female participants of reproductive potential (defined as women who have not been post-menopausal for at least 24 consecutive months, i.e, who have had menses within 24 months

© 2024 Lu MT et al. *JAMA Cardiology*.

prior to study entry, and women who have not undergone surgical sterilization, specifically hysterectomy or bilateral oophorectomy) must have a negative serum or urine pregnancy test within 48 hours prior to entry.

- For women of reproductive potential, willingness to use contraceptives as described in the product information for pitavastatin. Contraceptives must be used at least two weeks before initiation of study drug and must be continued 6 weeks after cessation of study drug.
  If participating in sexual activity that could lead to pregnancy, women must use a form of contraceptive. At least one of the following methods must be used appropriately:
  - Condoms (male or female) with or without spermicidal agent
  - Diaphragm or cervical cap with spermicidal agent
  - Intrauterine device (IUD)
  - Hormone-based contraceptive
  - Tubal ligation
  - Tubal micro-inserts

  Women who are not of reproductive potential as defined above are eligible without the use of contraception.
- Men and women age ≥40 and ≤75 years of age.
- Ability and willingness of participant or legal representative to provide written informed consent.

<u>REPRIEVE (A5332) Exclusion Criteria:</u>

- Clinical ASCVD, as defined by 2013 ACC/AHA guidelines, including a previous diagnosis of any of the following:
  - AMI
  - Acute coronary syndromes
  - Stable or unstable angina
  - Coronary or other arterial revascularization
  - Stroke
  - TIA
  - Peripheral arterial disease presumed to be of atherosclerotic origin
- Current diabetes mellitus if LDL ≥70 mg/dL.
- 10-year ASCVD risk score estimated by Pooled Cohort Equations >15%
  - If LDL <70 mg/dL, participant is eligible regardless of risk score in line with the ACC/AHA 2013 Prevention Guidelines.
  - See Fasting LDL Inclusion Criteria for LDL requirements by risk score.
  - Cardiovascular Risk Assessment Tool, for detailed instructions concerning access and use of the 10-year ASCVD risk score calculator (see Section 6.3.4 of Protocol).
- Active cancer within 12 months prior to study entry.
  Exceptions:
  - Successfully treated non-melanomatous skin cancer
  - Kaposi sarcoma without visceral organ involvement
- Known decompensated cirrhosis.
- History of myositis or myopathy with active disease in the 180 days prior to study entry.
- Known untreated symptomatic thyroid disease.
- History of allergy or severe adverse reaction to statins.
- Use of specific immunosuppressants or immunomodulatory agents, including but not limited to tacrolimus, sirolimus, rapamycin, mycophenolate, cyclosporine, TNF-alpha blockers or antagonists, azathioprine, interferon, growth factors, or intravenous immunoglobulin (IVIG), in the 30 days prior to study entry. Use of oral prednisone ≤10 mg/day or equivalent dosage is allowed.
- Current use of erythromycin, colchicine, or rifampin.
- Use of any statin drugs, gemfibrozil, or PCSK9 inhibitors in the 90 days prior to study entry.
- Current use of an investigational new drug that would be contraindicated.

© 2024 Lu MT et al. *JAMA Cardiology*.

- Serious illness or trauma requiring systemic treatment or hospitalization in the 30 days prior to study entry.
- Known active or recent (not fully resolved within 30 days prior to study entry) systemic bacterial, fungal, parasitic, or viral infections (except HIV, HBV, human papillomavirus [HPV], or HCV).
- Current breastfeeding.
- Alcohol or drug use that, in the opinion of the site investigator, would interfere with completion of study procedures.
- Other medical, psychiatric, or psychological condition that, in the opinion of the site investigator, would interfere with completion of study procedures and or adherence to study drug.

Diet, Activity, and Smoking Cessation Guidance

Information on diet, activity, smoking cessation, adhering to antiretroviral therapy, and adhering to the study medications was given to participants at each annual visit. The guidance on lifestyle was based on the NHLBI's Management of Blood Cholesterol in Adults[1] and was included information on the topics listed below:

- Goals of Therapeutic Lifestyle Changes (TLC)
- Physical Activity
- TLC Diet: Daily Food Guide Food Groups
  - Fruits and Vegetables
  - Meat, Poultry, Fish, Dry Beans, Eggs, and Nuts
  - Fats and Oils
  - Breads, Cereals, Rice, Pasta, and Other Grains
  - Sweets and Snacks
- Recommendations on Quitting Smoking
- Recommendations on adhering to the HIV medications prescribed by your doctor
- Recommendations on adhering to the study medication (pitavastatin or placebo)

Handling of Missing Data

<u>Missing data for CT scan outcomes arose via the following mechanisms:</u>

- Missed visits
- Participant loss to follow-up or premature study discontinuation
- CT scan complications at the time of the visit
- Scans that were non-diagnostic for either qualitative (presence of plaque) or quantitative (plaque volume) assessment

The primary analysis used a complete case approach. In sensitivity analyses, all missing data (regardless of reason) were imputed using a multiple imputation algorithm. 50 imputed datasets were created and analyzed and the results combined using standard multiple imputation methodology.

Models for the imputation used fully conditional models using all available plaque outcomes across both time-points (including the calcium and Leaman scores) and natal sex, age, ASCVD risk score at REPRIEVE entry, race, and ethnicity as covariate effects. A two-step procedure was used that mirrored the process by which scans were read during the study.

- Step 1 first created 50 complete datasets for the qualitative assessments of the present of any plaque and any noncalcified plaque. Imputation for each data element in the imputation used an iterative process with models based on discriminant analysis with 20 burn-in iterations.

© 2024 Lu MT et al. *JAMA Cardiology*.

- In Step 2, each of the 50 imputed datasets were evaluated for participants with evidence of plaque at either time-point. Records for those with any evidence of plaque in either assessment were included in a second imputation process to complete missing data for the quantitative assessment. Missing quantitative assessments among those records with no evidence of plaque in either scan were derived as zero, consistent with the outcome derivation.
  Imputation in Step 2 used normal error regression analysis with predicted mean matching to ensure all missing outcomes were imputed within the range of the data. To ensure quantitative noncalcified plaque volume was constrained to be less that any imputed total plaque volume, imputation was performed for the percent of total plaque that is noncalcified. Log transformations were used for the total plaque, Agatson calcium score and Leaman score outcomes, a logit transformation was used for percent of noncalcified plaque. The iterative imputation was again used with 20 burn-in iterations, however, since the Step 1 imputation process had generated the framework for the 50 imputed datasets, only 1 imputation for each of the 50 input datasets was then needed.

Imputation used proc MI and proc MIANALYZE implemented in SAS 9.4M7.

© 2024 Lu MT et al. *JAMA Cardiology*.

eTable 1. Characteristics of Enrolled REPRIEVE Mechanistic Substudy and Overall REPRIEVE Trial Participants at Baseline*

**Characteristic**	**Overall REPRIEVE Trial (N = 7769)**	**REPRIEVE Mechanistic Substudy (N=804)**
Age		
Median (IQR) — yr	50 (45–55)	51 (47-55)
Distribution — no. (%)		
40–49 yr	3730 (48.0%)	346 (43%)
50–59 yr	3361 (43.3%)	395 (49%)
≥60 yr	678 (8.7%)	63 (8%)
Sex — no. (%)†		
Female	2419 (31.1%)	139 (17%)
Gender identity — no. (%)		
Cisgender	7367 (94.8%)	764 (95%)
Transgender	127 (1.6%)	16 (2%)
Not reported	275 (3.5%)	24 (3%)
Race — no. (%)‡		
Asian	1138 (14.6%)	10 (1%)
Black or African American	3208 (41.3%)	292 (36%)
White	2704 (34.8%)	425 (53%)
Other	719 (9.3%)	77 (10%)
Ethnic group in North America — no./total no. (%)§		
Hispanic or Latino	698/3918 (17.8%)	192 (24%)
Not Hispanic or Latino	3186/3918 (81.3%)	601 (75%)
Unknown	34/3918 (0.9%)	11 (1%)
Atherosclerotic Cardiovascular Disease risk score — %¶		
Median (IQR)	4.5 (2.1,7.0)	4.6 (2.6,7.0)
Distribution — no. (%)		
0 to <2.5	2156 (27.8%)	184 (23%)
2.5 to <5	2055 (26.5%)	257 (32%)
5-10	2995 (38.6%)	311 (39%)
>10	563 (7.2%)	52 (6%)
Nadir CD4 level		
Distribution — no. (%)		
<200 cells/per mm3	3801 (48.9%)	400 (50%)
200–349 cells/per mm3	2041 (26.3%)	217 (27%)
≥350 cells/per mm3	1665 (21.4%)	159 (20%)
Unknown no. of cells/per mm3	262 (3.4%)	28 (3%)
CD4 count — no. (%)		
≤500 cells/per mm3	2510 (32.3%)	277 (34%)
>500 cells/per mm3	5259 (67.7%)	527 (66%)
HIV-1 RNA — no./total no. (%)‖		
<LLOQ copies/ml	5250 (87.5%)	696 (88%)
LLOQ to <400 copies/ml	617 (10.3%)	79 (10%)
≥400 copies/ml	130 (2.2%)	17 (2%)

* Percentages may not total 100 because of rounding. HIV denotes human immunodeficiency virus, and IQR interquartile range. REPRIEVE Mechanistic Substudy participants were also enrolled in the main REPRIEVE trial.
‡ Race was reported by the participants. "Other" race includes participants who identified as native or indigenous to the enrollment region, as having more than one race, or as having an unknown race.
§ Ethnic group is reported according to the National Institutes of Health definition for participants in the United States (including Puerto Rico) and Canada only and is not applicable to other geographic regions.
¶ The 10-Year Atherosclerotic Cardiovascular Disease risk score is calculated by assessing age, sex, race, systolic blood pressure, total and high-density lipoprotein cholesterol, treatment for hypertension, smoking history, and presence of diabetes.
‖ The level of HIV-1 RNA was measured if data were available through standard care. The assays that were used for testing varied, including assays with a lower limit of quantification (LLOQ) of 20 to 400 copies per milliliter.
Abbreviations: HIV, human immunodeficiency virus; RNA, ribonucleic acid; LLOQ, lower limit of quantification; REPRIEVE, Randomize Trial to Prevent Cardiovascular Events in HIV.

© 2024 Lu MT et al. *JAMA Cardiology*.

eTable 2. Baseline Characteristics of Participants Enrolled and With at Least 1 Evaluable Scan by Treatment Arm

Characteristic		Enrolled Participants		mITT Analysis Participants	
		Pitavastatin (N=402)	Placebo (N=402)	Pitavastatin (N=386)	Placebo (N=388)
Age (years)	Mean (SD)	51 (6)	51 (6)	51 (6)	51 (6)
	40-49	161 (40%)	185 (46%)	155 (40%)	177 (46%)
	50-59	211 (52%)	184 (46%)	203 (53%)	179 (46%)
	60+	30 (7%)	33 (8%)	28 (7%)	32 (8%)
Natal sex	Female	70 (17%)	69 (17%)	64 (17%)	66 (17%)
Gender identity	Cisgender	379 (94%)	385 (96%)	366 (95%)	374 (96%)
	Transgender Spectrum	8 (2%)	8 (2%)	8 (2%)	8 (2%)
	Not reported	15 (4%)	9 (2%)	12 (3%)	6 (2%)
Race[2]	Asian	5 (1%)	5 (1%)	5 (1%)	5 (1%)
	Black or African American	137 (34%)	155 (39%)	131 (34%)	147 (38%)
	White	210 (52%)	215 (53%)	203 (53%)	209 (54%)
	Other	50 (12%)	27 (7%)	47 (12%)	27 (7%)
Ethnicity[3]	Hispanic or Latino	103 (26%)	89 (22%)	101 (26%)	85 (22%)
	Not Hispanic or Latino	294 (73%)	307 (76%)	281 (73%)	297 (77%)
	Unknown	5 (1%)	6 (1%)	4 (1%)	6 (2%)
ASCVD risk score (%)	Median (Q1,Q3)	4.5 (2.6,6.8)	4.6 (2.6,7.0)	4.4 (2.6,6.7)	4.6 (2.6,7.0)
	0-<2.5	90 (22%)	94 (23%)	87 (23%)	91 (23%)
	2.5-<5	132 (33%)	125 (31%)	127 (33%)	122 (31%)
	5-10	153 (38%)	158 (39%)	147 (38%)	150 (39%)
	>10	27 (7%)	25 (6%)	25 (6%)	25 (6%)
Coronary artery calcium score[4] in Agatston units	0	239 (59%)	239 (59%)	237 (61%)	236 (61%)
	1-100	92 (23%)	91 (23%)	91 (24%)	90 (23%)
	101-400	35 (9%)	31 (8%)	35 (9%)	31 (8%)
	>400	5 (1%)	9 (2%)	5 (1%)	9 (2%)
Smoking status	Current	97 (24%)	100 (25%)	93 (24%)	97 (25%)
	Former	138 (34%)	111 (28%)	133 (35%)	107 (28%)
	Never	166 (41%)	190 (47%)	159 (41%)	183 (47%)
Substance use[5]	Current	10 (2%)	8 (2%)	10 (3%)	8 (2%)
	Former	197 (49%)	199 (50%)	187 (49%)	191 (49%)
	Never	194 (48%)	193 (48%)	188 (49%)	187 (48%)
Use of antihypertensive medication		88 (22%)	74 (18%)	82 (21%)	72 (19%)
Systolic blood pressure (mmHg)	Mean (SD)	123 (13)	123 (13)	123 (13)	122 (13)
Total cholesterol (mg/dL)[6]	Median (Q1,Q3)	186 (163,205)	183 (160,209)	186 (164,205)	183 (160,209)
HDL-C (mg/dL)[6]	Median (Q1,Q3)	49 (40,60)	48 (39,60)	49 (41,61)	48 (39,61)
LDL-C (mg/dL)[6]	Median (Q1,Q3)	107 (87,125)	108 (88,128)	107 (87,125)	107 (87,128)

© 2024 Lu MT et al. *JAMA Cardiology*.

Characteristic		Enrolled Participants		mITT Analysis Participants	
		Pitavastatin (N=402)	Placebo (N=402)	Pitavastatin (N=386)	Placebo (N=388)
Triglycerides (mg/dL)[6]	Median (Q1,Q3)	112 (79,165)	112 (77,171)	111 (78,165)	111 (77,172)
Family history of premature CVD	Unknown	12 (3%)	11 (3%)	10 (3%)	11 (3%)
		102 (25%)	77 (19%)	98 (25%)	77 (20%)
BMI (kg/m²)	Mean (SD)	27.3 (4.6)	27.4 (4.4)	27.2 (4.5)	27.4 (4.4)
	<25	135 (34%)	134 (33%)	130 (34%)	132 (34%)
	25-29.9	167 (42%)	157 (39%)	164 (42%)	150 (39%)
	30+	100 (25%)	111 (28%)	92 (24%)	106 (27%)
Prior statin use[7]		32 (8%)	33 (8%)	30 (8%)	31 (8%)
Pre-existing diabetes mellitus		2 (<0.5%)	0 (0%)	1 (<0.5%)	0 (0%)
Use of antidiabetic medication		1 (<0.5%)	0 (0%)	1 (<0.5%)	0 (0%)
Use of ACE inhibitors or ARBs		57 (14%)	48 (12%)	54 (14%)	47 (12%)
Use of antiplatelet therapy (including aspirin)[8]		31 (8%)	28 (7%)	29 (8%)	28 (7%)
Use of non-statin lipid-lowering therapy		32 (8%)	26 (6%)	31 (8%)	26 (7%)
Time since HIV diagnosis (years)	Median (Q1,Q3)	15 (9,21)	15 (9,22)	15 (9,21)	15 (9,22)
Nadir CD4 (cells/mm³)	<50	82 (20%)	91 (23%)	80 (21%)	86 (22%)
	50-199	109 (27%)	118 (29%)	107 (28%)	114 (29%)
	200-349	115 (29%)	102 (25%)	109 (28%)	100 (26%)
	350+	80 (20%)	79 (20%)	76 (20%)	77 (20%)
	Unknown	16 (4%)	12 (3%)	14 (4%)	11 (3%)
Total ART use (years)	<5	66 (16%)	62 (15%)	63 (16%)	59 (15%)
	5-10	102 (25%)	108 (27%)	98 (25%)	107 (28%)
	10+	234 (58%)	232 (58%)	225 (58%)	222 (57%)
CD4 count (cells/mm³)	Mean (SD)	624 (268)	640 (295)	620 (266)	638 (294)
	<350	59 (15%)	58 (14%)	58 (15%)	56 (14%)
	350-499	78 (19%)	82 (20%)	74 (19%)	79 (20%)
	500+	265 (66%)	262 (65%)	254 (66%)	253 (65%)
HIV-1 RNA (copies/mL)	<LLQ	350 (88%)	346 (88%)	337 (88%)	337 (89%)
	LLQ -< 400	40 (10%)	39 (10%)	37 (10%)	36 (9%)
	400+	9 (2%)	8 (2%)	9 (2%)	7 (2%)
Entry ART regimen class	NRTI + INSTI	175 (44%)	176 (44%)	167 (43%)	172 (44%)
	NRTI + NNRTI	104 (26%)	103 (26%)	102 (26%)	102 (26%)
	NRTI + PI	77 (19%)	61 (15%)	73 (19%)	56 (14%)
	NRTI-sparing	11 (3%)	17 (4%)	10 (3%)	15 (4%)
	Other NRTI-containing	35 (9%)	45 (11%)	34 (9%)	43 (11%)
Entry NRTI	TDF	191 (48%)	210 (52%)	185 (48%)	203 (52%)
	TAF	112 (28%)	107 (27%)	109 (28%)	106 (27%)
	ABC	80 (20%)	61 (15%)	74 (19%)	57 (15%)
	No NRTI	14 (3%)	20 (5%)	13 (3%)	18 (5%)

© 2024 Lu MT et al. *JAMA Cardiology*.

Characteristic		Enrolled Participants		mITT Analysis Participants	
		**Pitavastatin (N=402)**	**Placebo (N=402)**	**Pitavastatin (N=386)**	**Placebo (N=388)**
	Other	5 (1%)	4 (1%)	5 (1%)	4 (1%)
Entry INSTI	No INSTI	187 (47%)	173 (43%)	180 (47%)	166 (43%)
	DTG	104 (26%)	99 (25%)	98 (25%)	97 (25%)
	EVG	82 (20%)	85 (21%)	79 (20%)	82 (21%)
	RAL	29 (7%)	45 (11%)	29 (8%)	43 (11%)

mITT population included participants with an evaluable entry CT, 2-year CT, or both CTs. Missing data for the remaining 30 participants are considered missing at random and ignored.
[1]All statistics are calculated out of participants with data collected. Missing data: Smoking status (n=1); Substance use (n=1); HIV-1 RNA (n=3). [2]'Other' race includes participants self-identifying as: native or indigenous to the enrollment region; more than one race (with no single race noted as predominant); or of unknown race. [3]Ethnicity is presented per NIH definition. [4]Missing coronary artery calcium score in Enrolled: Pitavastatin (n=31), Placebo (n=32). Missing coronary artery calcium score in MI analysis: Pitavastatin (n=18), Placebo (n=22). [5]Substance use includes use of cocaine, methamphetamine, and intravenous drugs. [6]Screening lipids presented. [7]Prior statin use based on site report at Entry. [8]Aspirin use in antiplatelet therapy is limited to chronic aspirin use defined as more than 60 days.
Abbreviations: mITT, modified intention to treat; ART, antiretroviral therapy; ASCVD, atherosclerotic cardiovascular disease; HDL, high density lipoprotein; LDL, low density lipoprotein; BMI, body mass index; CVD, cardiovascular disease; ACE, angiotensin-converting enzyme; ARB, angiotensin receptor blocker; HIV, human immunodeficiency virus; RNA, ribonucleic acid; NRTI, nucleoside reverse transcriptase inhibitor; NNRTI, non-nucleoside reverse transcriptase inhibitor; INSTI, integrase strand inhibitor; PI, protease inhibitor; TDF, tenofovir disoproxil fumarate; TAF, tenofovir alafenamide; ABC, abacavir; DTG, dolutegravir; EVG, elvitegravir; RAL, raltegravir; SD, standard deviation; LLQ, lower limit of quantification

© 2024 Lu MT et al. *JAMA Cardiology*.

eTable 3. Sensitivity Analysis: Multiple Imputation of Changes in Coronary Artery Plaque by Treatment Arm

	Treatment arm		Treatment effect
Outcome	Pitavastatin (N=386)	Placebo (N=388)	Estimated difference adjusted for baseline (95% CI)
**Primary**			
Noncalcified plaque volume, mm3			
Change from baseline, mean (95% CI)	-2.4 [-8.1, 3.31]	4.02 [-1.8, 9.83]	-6.5 [-14.3, 1.30]
Fold-change from baseline (95% CI)	0.94 [0.80, 1.11]	1.24 [1.06, 1.46]	0.74 [0.59, 0.93]
*Progression of NCP**	79 (20%)	114 (29%)	0.74 [0.58, 0.95]

Noncalcified plaque defined as plaque voxels with attenuation <350 HU.
[1]Within group effects present the mean with 95% confidence interval.
[2]For continuous outcomes the treatment effect is given as the mean difference between treatment groups; for binary outcomes it is a relative risk.
*n(%) with progression calculated as an average across the 50 imputations
Abbreviations: NCP, noncalcified plaque; CI, confidence interval

© 2024 Lu MT et al. *JAMA Cardiology*.

eTable 4. Sensitivity Analysis: Per-Protocol Changes in Coronary Artery Plaque by Treatment Arm

	Treatment arm		Treatment effect
Outcome	Pitavastatin (N=270)	Placebo (N=279)	Estimated difference adjusted for baseline (95% CI)
**Primary**			
*Noncalcified plaque volume, mm3**			
Change from baseline, mean (SD)	-1.5 (24.7)	3.8 (25.4)	-5.4 [-9.6, -1.0]
Fold-change from baseline (95% CI)	0.96 (0.91-1.01)	1.03 (0.99-1.06)	0.93 [0.88, 1.00]
Progression of NCP	49 (18%)	78 (28%)	0.68 [0.52, 0.90]

Noncalcified plaque defined as plaque voxels with attenuation <350 HU.
* Change in noncalcified plaque volume could be assessed in a subset of 528 participants (260 pitavastatin and 268 placebo).
[1]Within group effects present the mean with standard deviation unless otherwise noted.[2]For continuous outcomes the treatment effect is given as the mean difference between treatment groups; for binary outcomes it is a relative risk. All treatment effects and associated p-values are adjusted for baseline value.
Abbreviations: NCP, noncalcified plaque; CI, confidence interval; SD, standard deviation

© 2024 Lu MT et al. *JAMA Cardiology*.

eTable 5. Coronary Artery Plaque Changes by Treatment Arm, Secondary Analysis in Subgroup With Plaque at Entry and Year 2 CT Available for Paired Analysis

	Treatment arm[1]		Treatment effect[2]
Outcome	Pitavastatin (N=139)	Placebo (N=148)	Estimated difference adjusted for baseline (95% CI)
**Primary**			
*Noncalcified plaque volume, mm3**			
Baseline, mean (SD)	117.5 (277.2)	121.1 (132.3)	
Month 24, mean (SD)	113.8 (276.9)	126.1 (137.7)	
Change from baseline, mean (SD)	-3.7 (37.7)	5.03 (39.0)	-8.8 [-17.9, 0.36]
Fold-change from baseline (95% CI)	0.91 (0.81, 1.01)	1.03 (0.96, 1.10)	0.88 [0.77, 1.00]
Progression of NCP	51 (37%)	77 (52%)	0.71 [0.55, 0.93]
**Secondary**			
Total plaque volume, mm3			
Baseline, mean (SD)	151.3 (301.7)	153.4 (164.4)	
Month 24, mean (SD)	156.8 (310.9)	167.6 (180.7)	
Change from baseline, mean (SD)	5.52 (43.6)	14.2 (45.9)	-8.6 [-19.4, 2.23]
Progression of total plaque	73 (60%)	90 (66%)	0.92 [0.76, 1.11]
**Exploratory**			
Low attenuation plaque volume, mm3			
Baseline, mean (SD)	7.47 (24.2)	8.93 (21.2)	
Month 24, mean (SD)	5.47 (14.3)	8.75 (17.5)	
Change from baseline, mean (SD)	-2.0 (14.9)	-0.2 (11.9)	-2.4 [-4.7, -0.2]

Noncalcified plaque defined as plaque voxels with attenuation <350 HU. Total plaque includes all plaque voxels (noncalcified + calcified). Low attenuation plaque defined as <30 HU.

* Change in noncalcified plaque volume could be assessed in a subset of 270 participants (129 pitavastatin and 141 placebo).

[1]Within group effects present the mean with standard deviation unless otherwise noted.

[2]For continuous outcomes the treatment effect is given as the mean difference or relative fold change between treatment groups; for binary outcome of progression it is a relative risk. Treatment effects and associated p-values for continuous outcomes are adjusted for baseline value.

Abbreviations: NCP, noncalcified plaque; SD, standard deviation; CI, confidence interval

© 2024 Lu MT et al. *JAMA Cardiology*.

eTable 6. Distributions of Fasting Lipids (Entry, Month 4, and Month 24)

	Entry		Month 4		Year 2	
	Pitavastatin	Pitavastatin	Pitavastatin	Placebo	Pitavastatin	Placebo
**LDL Cholesterol, mg/dL**	**105 (88, 124)**	**107 (90, 129)**	**69 (53, 84)**	**105 (84, 126)**	**75 (59, 93)**	**108 (87, 128)**
Change from Baseline			-35 (-51, -19)	-4 (-16, 9)	-29 (-49, -11)	0 (-15, 16)
Change from Baseline, Mean (95% CI)			-35.5 [-38.4, -32.6]	-3.5 [-5.7, -1.3]	-28.5 [-31.9, -25.1]	-0.8 [-3.8, 2.2]
**Non-HDL Cholesterol, mg/dL**	**130 (111, 156)**	**132 (111, 161)**	**90 (74, 110)**	**129 (106, 155)**	**99 (81, 124)**	**133 (109, 159)**
Change from Baseline			-40 (-57, -20)	-2 (-17, 11)	-35 (-54, -12)	2 (-16, 18)
Change from Baseline, Mean (95% CI)			-38.7 [-41.8, -35.5]	-2.9 [-5.4, -0.5]	-31.1 [-34.8, -27.5]	-0.1 [-3.4, 3.2]

Data are reported for all of the enrolled participants with available biomarker results at each timepoint. Missing LDL values at entry were 5 for pitavastatin and 6 for placebo; at month 4, 53 for pitavastatin and 48 for placebo; at year 2, 58 for pitavastatin and 52 for placebo. Non-HDL cholesterol had similar numbers of missing values.
Data presented correspond to median (Q1, Q3) unless stated otherwise.
Abbreviations: LDL, low density lipoprotein; HDL, high density lipoprotein; CI, confidence interval

© 2024 Lu MT et al. *JAMA Cardiology*.

eTable 7. Distributions of Inflammatory and Immune Biomarkers (Month 4)

	Month 4		
	**Pitavastatin**	**Placebo**	**P-Value**
Inflammatory Markers			
**Lp-PLA2 (ng/mL)**	**112 (80.3, 145)**	**141 (104, 183)**	**<0.001**
Change from Baseline	-13.9 (-39.2, 6.63)	14.3 (-7.50, 36.2)	<0.001
Mean Fold Change [95% CI]	0.88 [0.85, 0.91]	1.11 [1.08, 1.14]	
**oxLDL (U/L)**	**56.9 (44.7, 70.5)**	**73.6 (57.7, 91.4)**	**<0.001**
Change from Baseline	3.29 (-12.2, 16.1)	16.7 (2.58, 33.7)	<0.001
Mean Fold Change [95% CI]	1.04 [1.00, 1.09]	1.33 [1.28, 1.38]	
**hs-CRP (mg/L)** [1]	**1.50 (0.70, 3.00)**	**1.80 (1.00, 3.50)**	**0.010**
Change from Baseline	-0.10 (-1.20, 0.40)	-0.10 (-0.89, 0.70)	0.07
Mean Fold Change [95% CI]	0.84 [0.74, 0.94]	0.99 [0.87, 1.12]	
Immune Markers			
**MCP-1 (pg/mL)**	**194 (151, 238)**	**185 (150, 230)**	**0.23**
Change from Baseline	1.93 (-46.1, 43.8)	-1.19 (-46.3, 44.0)	0.72
Mean Fold Change [95% CI]	1.01 [0.97, 1.05]	1.01 [0.97, 1.05]	
**sCD14 (ng/mL)**	**1675 (1416, 1998)**	**1725 (1425, 2043)**	**0.21**
Change from Baseline	-138 (-420, 156)	-88.5 (-389, 199)	0.22
Mean Fold Change [95% CI]	0.92 [0.89, 0.94]	0.95 [0.92, 0.98]	
**sCD163 (ng/mL)**	**843 (634, 1172)**	**875 (653, 1122)**	**0.96**
Change from Baseline	8.80 (-172, 220)	31.8 (-175, 220)	0.47
Mean Fold Change [95% CI]	1.02 [0.97, 1.06]	1.06 [1.01, 1.10]	
**IL-6 (pg/mL)**	**1.64 (0.96, 2.88)**	**1.57 (1.03, 2.71)**	**0.73**
Change from Baseline	0.00 (-0.64, 0.66)	-0.01 (-0.70, 0.79)	0.79
Mean Fold Change [95% CI]	1.02 [0.94, 1.11]	1.05 [0.97, 1.14]	

Data are reported for all of the enrolled participants with available biomarker results at each timepoint. Missing Lp-PLA2 values at month 4 were 54 for pitavastatin and 48 for placebo. Other biomarkers had similar numbers of missing values.
P-values test for distribution shifts between those on Pitavastatin versus Placebo, within timepoint. Data presented correspond to median (Q1, Q3) unless stated otherwise. For hs-CRP censored values below the assay limit are randomly imputed based on a uniform distribution, otherwise censored values below the assay limit are imputed as result-0.01, values above the assay limit are imputed as result+0.1.
Abbreviations: Lp-PLA2, Lipoprotein-Associated Phospholipase A2; oxLDL, oxidized low density lipoprotein; hs-CRP, high-sensitivity C-reactive protein; MCP-1, monocyte chemoattractant protein-1; sCD14, soluble CD14; sCD163, soluble CD163; IL-6, interleukin-6; CI, confidence interval

© 2024 Lu MT et al. *JAMA Cardiology*.

eFigure 1. Example Baseline and 2-Year Follow-Up Coronary CTA Demonstrating Plaque Progression on Placebo (Top Row) and Plaque Regression on Pitavastatin (Bottom Row)

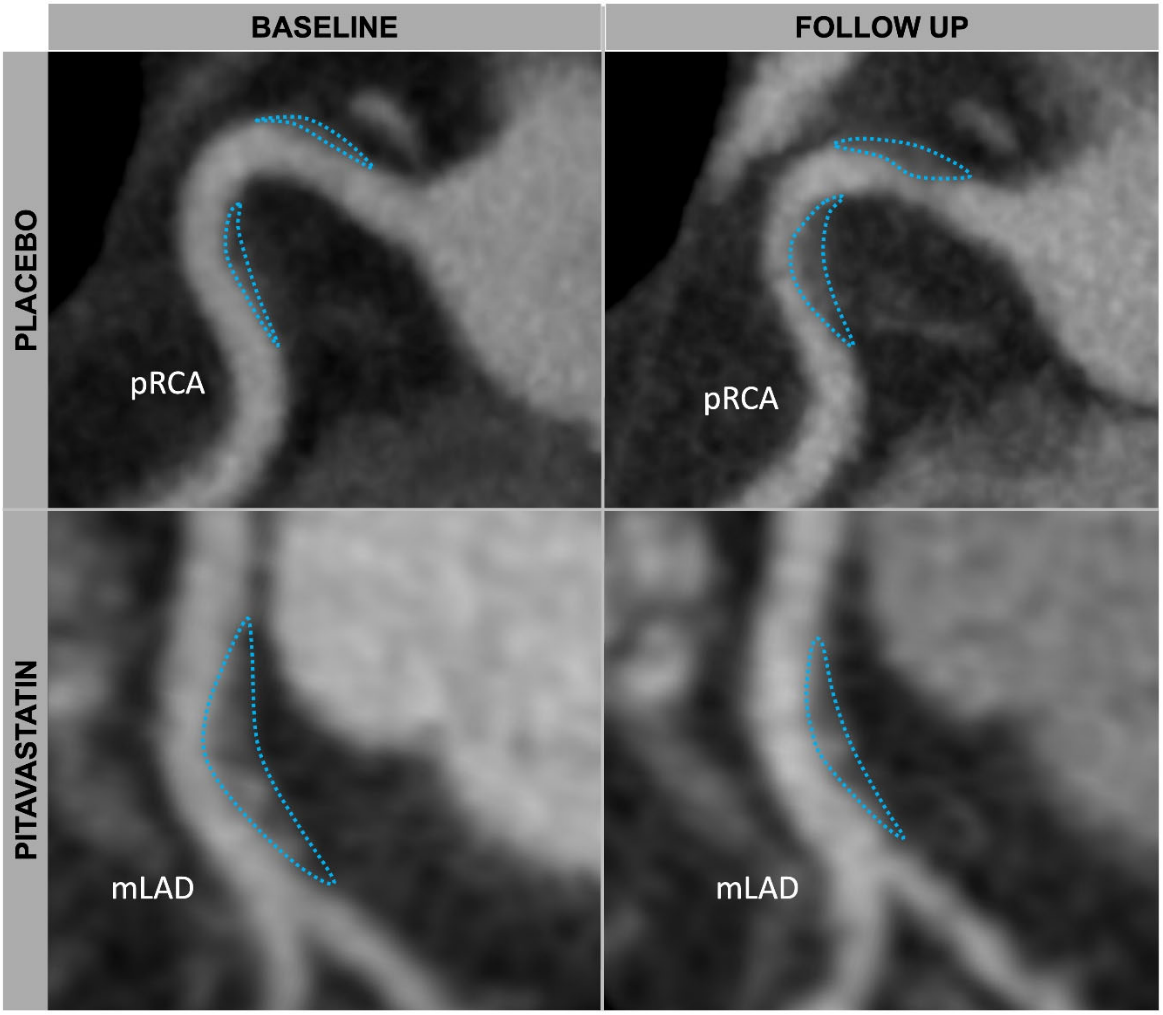

Abbreviatons: CTA, Computed tomography angiography; pRCA, proximal right coronary artery; mLAD, mid left anterior descending coronary artery

© 2024 Lu MT et al. *JAMA Cardiology*.

eFigure 2. Effect of Individual Biomarkers on Change in Noncalcified Plaque Volume

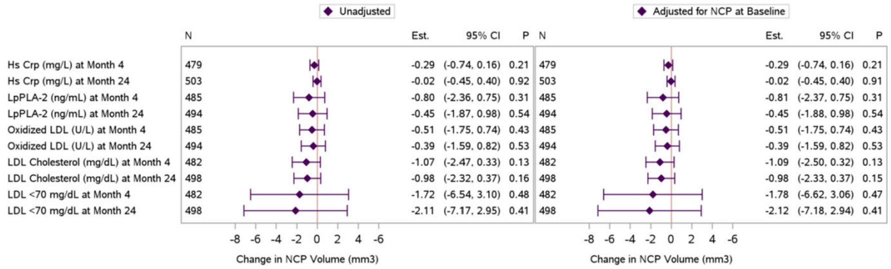

Effect size is per 25% decrease in the biomarker. The p-value tests for linearly increasing log RR.
Mechanistic analyses were limited to the per protocol population with paired biomarkers and plaque outcomes.
Abbreviations: Hs-CRP, high-sensitivity C-reactive protein; LpPLA-2, Lipoprotein-Associated Phospholipase A2; LDL, low density lipoprotein; CI, confidence interval; NCP, noncalcified plaque

© 2024 Lu MT et al. *JAMA Cardiology*.

eFigure 3. Effect of Treatment on Noncalcified Plaque Volume, Adjusted for Change in Individual Biomarkers

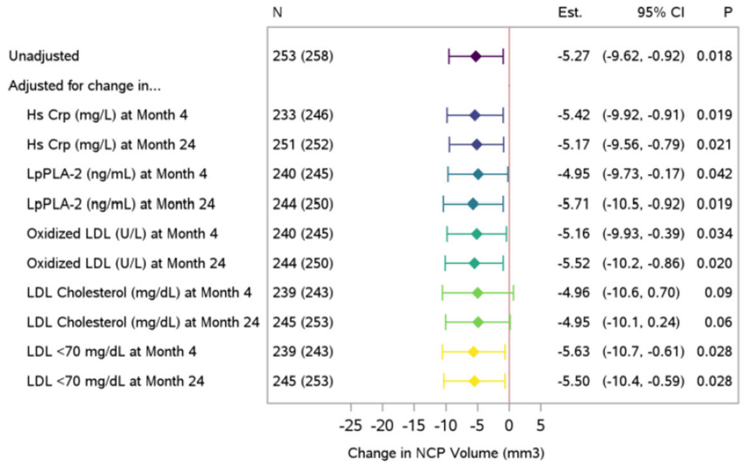

The effect of pitavastatin on noncalcified plaque volume persisted after adjustment for biomarkers.
Mechanistic analyses were limited to the per protocol population with paired biomarkers and plaque outcomes. The first N corresponds to the number in the pitavastatin arm, and the (N) to the number in the placebo arm.
Effect size is presented with placebo as reference. All analyses adjusted for noncalcified plaque at baseline.
Abbreviations: Hs-CRP, high-sensitivity C-reactive protein; LpPLA-2, Lipoprotein-Associated Phospholipase A2; LDL, low density lipoprotein; CI, confidence interval; NCP, noncalcified plaque

© 2024 Lu MT et al. *JAMA Cardiology*.

eFigure 4. Effect of Treatment on Noncalcified Plaque Progression, Adjusted for Change in Individual Biomarkers

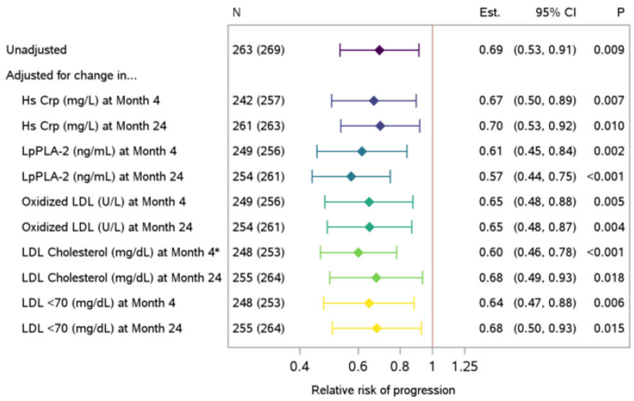

The effect of pitavastatin on noncalcified plaque volume persisted after adjustment for biomarkers.
Mechanistic analyses were limited to the per protocol population with paired biomarkers and plaque outcomes. The first N corresponds to the number in the pitavastatin arm, and the (N) to the number in the placebo arm.
Effect size is presented with placebo as reference. All analyses adjusted for noncalcified plaque at baseline. The p-value tests for a linearly increasing log RR.
Abbreviations: Hs-CRP, high-sensitivity C-reactive protein; LpPLA-2, Lipoprotein-Associated Phospholipase A2; LDL, low density lipoprotein; CI, confidence interval

© 2024 Lu MT et al. *JAMA Cardiology*.

eReferences.

1. Managing Blood Cholesterol in Adults. National Heart, Lung, and Blood Institute, 2013. (https://www.nhlbi.nih.gov/health-topics/management-blood-cholesterol-in-adults).
2. Hicks KA, Tcheng JE, Bozkurt B, et al. 2014 ACC/AHA Key Data Elements and Definitions for Cardiovascular Endpoint Events in Clinical Trials: A Report of the American College of Cardiology/American Heart Association Task Force on Clinical Data Standards (Writing Committee to Develop Cardiovascular Endpoints Data Standards). J Am Coll Cardiol 2015;66(4):403-69. (In eng). DOI: 10.1016/j.jacc.2014.12.018.

© 2024 Lu MT et al. *JAMA Cardiology*.
