# Supplementary material for: Effects of Pitavastatin on Coronary Artery Disease and Inflammatory Biomarkers in HIV: Mechanistic Substudy of the REPRIEVE Randomized Clinical Trial
Source: JAMA Cardiol. 2024 Feb 21;9(4):323–34. doi: 10.1001/jamacardio.2023.5661 (PMC10882511; doi:10.1001/jamacardio.2023.5661)
Supplement: Supplement 3. — Group Information. REPRIEVE Trial Writing Group [file jamacardiol-e235661-s003.pdf]

*Indicates required information. Only first name, last name, and suffix will appear in PubMed.

*Group Name(s): REPRIEVE Trial Writing Group							
***First Name and Middle Initial(s)**	***Last Name**	***Suffix (eg, Jr, III)**	Academic Degrees	Institution	Location (city, state/province, country)	Role or Contribution, eg, chair, principal investigator	Group (if more than 1 Group listed in the byline) and/or Subgroup (eg, Steering Committee)
Carl J.	Fichtenbaum		MD	Cincinnati CRS; University of Cincinnati College of Medicine	Cincinnati, OH, USA	CRS Leader; REPRIEVE Committee Member	Operational Leadership Committee; Protocol Writing, Training and Implementation Committee; Publications Committee; Site Selection, Performance, and Close Out Committee
Judith A.	Aberg		MD	Mount Sinai Clinical and Translational Research Center; Icahn School of Medicine at Mount Sinai	New York, NY, USA	CRS Leader; REPRIEVE Committee Member	Operational Leadership Committee; Protocol Writing, Training and Implementation Committee; Publications Committee; Site Selection, Performance, and Close Out Committee
Eric S.	Daar		MD	Harbor UCLA CRS	Torrance, CA, USA	CRS Leader	
Babafemi	Taiwo		MD	Northwestern University CRS	Evanston, IL, USA	CRS Leader	
Susan L.	Koletar		MD	Ohio State University CRS; Ohio State University Medical Center	Columbus, OH, USA	CRS Leader; REPRIEVE Committee Member	Site Selection, Performance, and Close Out Committee
Kara W.	Chew		MD	UCLA CARE Center CRS	Los Angeles, CA, USA	CRS Leader	
Susan J.	Little		MD	UCSD Antiviral Research Center CRS	San Diego, CA, USA	CRS Leader	
Sonya L.	Heath		MD	Alabama CRS	Birmingham, AL, USA	CRS Leader	
Jeffrey M.	Jacobson		MD	Case CRS	Cleveland, OH, USA	CRS Leader	
Rajesh	Gandhi		MD	Massachusetts General Hospital CRS	Boston, MA, USA	CRS Leader	

*Indicates required information. Only first name, last name, and suffix will appear in PubMed.

*First Name and Middle Initial(s)	*Last Name	*Suffix (eg, Jr, III)	Academic Degrees	Institution	Location (city, state/province, country)	Role or Contribution, eg, chair, principal investigator	Group (if more than 1 Group listed in the byline) and/or Subgroup (eg, Steering Committee)
Gregory	Robbins		MD	Massachusetts General Hospital CRS; Massachusetts General Hospital	Boston, MA, USA	CRS Leader; REPRIEVE Committee Member	Site Selection, Performance, and Close Out Committee
Rachel M.	Presti		MD	Washington University Therapeutics (WT) CRS	St. Louis, MO, USA	CRS Leader	
Marshall	Glesby		MD	Weill Cornell Uptown CRS	New York, NY, USA	CRS Leader	
Annie	Luetkemeyer		MD	UCSF HIV/AIDS CRS	San Francisco, CA, USA	CRS Leader	
Pablo	Tebas		MD	Penn Therapeutics CRS	Philadelphia, PA, USA	CRS Leader	
Sharon A.	Riddler		MD	University of Pittsburgh CRS	Pittsburgh, PA, USA	CRS Leader	
Michael P.	Dube		MD	University of Southern California CRS	Los Angeles, CA, USA	CRS Leader	
Jorge L.	Santana-Bagur		MD	Puerto Rico AIDS Clinical Trials Unit CRS	San Juan, PR	CRS Leader	
Beverly E.	Sha		MD	Rush University CRS	Chicago, IL, USA	CRS Leader	
Jennifer	Manne		MD	Brigham and Women's Hospital Therapeutics CSR	Boston, MA, USA	CRS Leader	
Roberto	Arduino		MD	Houston AIDS Research Team CRS	Houston, TX, USA	CRS Leader	
Charles W.	Flexner		MD	John Hopkins University CRS	Baltimore, MD, USA	CRS Leader	
David W.	Haas		MD	Vanderbilt Therapeutics CRS	Nashville, TN, USA	CRS Leader	
David A.	Wohl		MD	Chapel Hill CRS	Chapel Hill, NC, USA	CRS Leader	
Magdalena E.	Sobieszczyk		MD	Columbia Physicians and Surgeons (P&S) CRS	New York, NY, USA	CRS Leader	
Karen T.	Tashima		MD	The Miriam Hospital (TMH) CRS	Providence, RI, USA	CRS Leader	
Sonal S.	Munsiff		MD	University of Rochester Adult HIV Therapeutic Strategies Network CRS	Rochester, NY, USA	CRS Leader	
Rachel	Bender Ignacio		MD	University of Washington AIDS CRS	Seattle, WA, USA	CRS Leader	
Kristen	Marks		MD	Weill Cornell Chelsea CRS	New York, NY, USA	CRS Leader	
Cornelius	Van Dam		MD	Greensboro CRS	Greensboro, NC, USA	CRS Leader	
Shobha	Swaminathan		MD	New Jersey Medical School Clinical Research Center CRS	Newark, NJ, USA	CRS Leader	
Thomas B.	Campbell		MD	University of Colorado Hospital	Aurora, CO, USA	CRS Leader	

*Indicates required information. Only first name, last name, and suffix will appear in PubMed.

***First Name and Middle Initial(s)**	***Last Name**	***Suffix (eg, Jr, III)**	Academic Degrees	Institution	Location (city, state/province, country)	Role or Contribution, eg, chair, principal investigator	Group (if more than 1 Group listed in the byline) and/or Subgroup (eg, Steering Committee)
Beverly	Alston-Smith		MD	National Institute of Allergy and Infectious Diseaes of the National [illegible]	Rockville, MD, USA	REPRIEVE Committee	Executive Committee; Publications Committee
Patricia	Bandettini		MD	National Heart, Lung, and Blood Institute of the National Institutes of Health; National Institutes of Health/National Heart Lung and Blood Institute	Bethesda, MD, USA	REPRIEVE Committee	Executive Committee; Operational Leadership Committee; Protocol Writing, Training, and Implementation Committee; Publications Committee; Site Selection, Performance, and Close Out Committee
Gerald	Bloomfield		MD	Duke University; Clinical Coordinating Center	Durham, NC, USA; Boston, MA, USA	REPRIEVE Committee	Executive Committee; Events Oversight Committee; Operational Leadership Committee; Protocol Writing, Training, and Implementation Committee; Publications Committee
Judith	Currier		MD, MPH	University of California Los Angeles David Geffen School of Medicine	Los Angeles, CA, USA	REPRIEVE Committee	Executive Committee; Operational Leadership Committee; Publications Committee

*Indicates required information. Only first name, last name, and suffix will appear in PubMed.

***First Name and Middle Initial(s)**	***Last Name**	***Suffix (eg, Jr, III)**	Academic Degrees	Institution	Location (city, state/province, country)	Role or Contribution, eg, chair, principal investigator	Group (if more than 1 Group listed in the byline) and/or Subgroup (eg, Steering Committee)
Patrice	Desvigne-Nickens		MD	National Heart, Lung, and Blood Institute of the National Institutes of Health; National Institutes of Health/National Heart Lung and Blood Institute	Bethesda, MD, USA	REPRIEVE Committee	Executive Committee; Operational Leadership Committee; Protocol Writing, Training, and Implementation Committee; Publications Committee; Site Selection, Performance, and Close Out Committee
Marissa	Diggs			Massachusetts General Hospital; Clinical Coordinating Center	Boston, MA, USA	REPRIEVE Committee	Executive Committee; EU Committee; Lab Committee; Operational Leadership Committee; Protocol Training, Writing, and Implementation Committee; Publications Committee; Site Selection, Performance, and Close Out Committee; Mechanistic Substudy CT Committee
Pamela S.	Douglas		MD	Duke University; Clinical Coordinating Center	Durham, NC, USA	REPRIEVE Committee; REPRIEVE Committee Chair (Events Oversight Committee)	Executive Committee; Events Oversight Committee; Operational Leadership Committee; Protocol Writing, Training, and Implementation Committee; Publications Committee; Mechanistic Substudy CT Committee

Supplemental Online Content: Nonauthor Collaborators

*Indicates required information. Only first name, last name, and suffix will appear in PubMed.

***First Name and Middle Initial(s)**	***Last Name**	***Suffix (eg, Jr, III)**	Academic Degrees	Institution	Location (city, state/province, country)	Role or Contribution, eg, chair, principal investigator	Group (if more than 1 Group listed in the byline) and/or Subgroup (eg, Steering Committee)
Kathleen V.	Fitch		NP	Massachusetts General Hospital	Boston, MA, USA	REPRIEVE Committee; REPRIEVE Committee Chair (EU Committee)	Executive Committee; Events Oversight Committee; EU Committee; Lab Committee; Operational Leadership Committee; Protocol Writing, Training, and Implementation Committee; Publications Committee; Site Selection, Performance, and Close Out Committee; Mechanistic Substudy CT Committee
Steven K.	Grinspoon		MD	Massachusetts General Hospital; Clinical Coordinating Center	Boston, MA, USA	REPRIEVE Committee Chair (Executive and Operational Leadership Committees); REPRIEVE Committee	Executive Committee; Events Oversight Committee; Operational Leadership Committee; Protocol Writing, Performance, and Close Out Committee; Publications Committee; Mechanistic Substudy CT Committee
Peter	Kim		MD	Division of AIDS of the National Institute of Health; National Institutes of Health/National Institute of Allergy and Infectious Diseases	Rockville, MD, USA	REPRIEVE Committee	Executive Committee; Operational Leadership Committee

*Indicates required information. Only first name, last name, and suffix will appear in PubMed.

***First Name and Middle Initial(s)**	***Last Name**	***Suffix (eg, Jr, III)**	Academic Degrees	Institution	Location (city, state/province, country)	Role or Contribution, eg, chair, principal investigator	Group (if more than 1 Group listed in the byline) and/or Subgroup (eg, Steering Committee)
Michael T.	Lu		MD, MPH	Massachusetts General Hospital; Data Coordinating Center	Boston, MA, USA	REPRIEVE Committee; REPRIEVE Committee Chair (Mechanistic Substudy CT Committee); Co-PI REPRIEVE Data Coordinating Center	Executive Committee; Events Oversight Committee; Operational Leadership Committee; Protocol Writing, Training, and Implementation Committee; Publications Committee; Site Selection, Performance, and Close Out Committee; Mechanistic Substudy CT Committee
Kayla	Paradis			Massachusetts General Hospital; Data Coordinating Center	Boston, MA, USA	REPRIEVE Committee	Executive Committee; Events Oversight Committee; EU Committee; Lab Committee; Operational Leadership Committee; Publications Committee; Mechanistic Substudy CT Committee
Heather J.	Ribaudo		PhD	Harvard T.H. Chan School of Public Health; Center for Biostatistics and AIDS Research at Harvard T.H. Chan School of Public Health	Boston, MA, USA	REPRIEVE Committee	Executive Committee; Events Oversight Committee; EU Committee; Operational Leadership Committee; Protocol Writing Committee; Publications Committee; Mechanistic Substudy CT Committee

*Indicates required information. Only first name, last name, and suffix will appear in PubMed.

***First Name and Middle Initial(s)**	***Last Name**	***Suffix (eg, Jr, III)**	Academic Degrees	Institution	Location (city, state/province, country)	Role or Contribution, eg, chair, principal investigator	Group (if more than 1 Group listed in the byline) and/or Subgroup (eg, Steering Committee)
Yves	Rosenberg		MD, MPH	National Heart, Lung, and Blood Institute of the National Institutes of Health	Bethesda, MD, USA	REPRIEVE Committee	Executive Committee
James	Troendle		PhD	National Heart, Lung, and Blood Institute of the National Institutes of Health	Bethesda, MD, USA	REPRIEVE Committee	Executive Committee
Mark	Byroads			Frontier Science Foundation; Data Management Center	Amherst, NY, USA	REPRIEVE Committee	Events Oversight Committee; Operational Leadership Committee; Protocol Writing, Training, and Implementation Committee; Site Selection, Performance, and Close Out Committee
Elaine	Gershman			Brigham and Women's Hospital	Boston, MA, USA	REPRIEVE Committee	Events Oversight Committee
Folake	Lawal			Division of AIDS of the National Institute of Health	Rockville, MD, USA	REPRIEVE Committee	Events Oversight Committee; Operational Leadership Committee; Protocol Writing, Training, and Implementation Committee; Publications Committee
Jorge	Leon-Cruz		MS	Harvard T.H. Chan School of Public Health; Center for Biostatistics and AIDS Research at Harvard T.H. Chan School of Public Health	Boston, MA, USA	REPRIEVE Committee	Events Oversight Committee; Protocol Writing, Training, and Implementation Committee; Publications Committee

Supplemental Online Content: Nonauthor Collaborators

*Indicates required information. Only first name, last name, and suffix will appear in PubMed.

*First Name and Middle Initial(s)	*Last Name	*Suffix (eg, Jr, III)	Academic Degrees	Institution	Location (city, state/province, country)	Role or Contribution, eg, chair, principal investigator	Group (if more than 1 Group listed in the byline) and/or Subgroup (eg, Steering Committee)
Rochelle	Louis			Massachusetts General Hospital	Boston, MA, USA	REPRIEVE Committee	Events Oversight Committee
Cheryl	Lowe		RN	Brigham and Women's Hospital	Boston, MA, USA	REPRIEVE Committee	Events Oversight Committee
Eva	Moy		CRA	Brigham and Women's Hospital	Boston, MA, USA	REPRIEVE Committee	Events Oversight Committee
Triin	Umbleja		MSc	Harvard T.H. Chan School of Public Health; Center for Biostatistics and AIDS Research at Harvard T.H. Chan School of Public Health	Boston, MA, USA	REPRIEVE Committee	Events Oversight Committee; EU Committee; Publications Committee; Mechanistic Substudy CT Committee
Namrata	Upadhyay			Massachusetts General Hospital	Boston, MA, USA	REPRIEVE Committee	Events Oversight Committee
Stephen	Wiviott		MD	Brigham and Women's Hospital	Boston, MA, USA	REPRIEVE Committee	Events Oversight Committee
Kenneth	Wood			Frontier Science Foundation; Data Management Center	Amherst, NY, USA	REPRIEVE Committee	Events Oversight Committee; EU Committee; Operational Leadership Committee; Protocol Writing, Training, and Implementation Committee; Site Selection, Performance, and Close Out Committee
Oladapo	Anthony			ROKC; Research Organisation (kc) ltd. (CRO for EU5332)	London, United Kingdom	REPRIEVE Committee	EU Committee
Radhika	Barve			Massachusetts General Hospital; Data Coordinating Center	Boston, MA, USA	REPRIEVE Committee	EU Committee
Fred	Bone			Frontier Science Foundation; Data Management Center	Amherst, NY, USA	REPRIEVE Committee	EU Committee; Lab Committee

*Indicates required information. Only first name, last name, and suffix will appear in PubMed.

*First Name and Middle Initial(s)	*Last Name	*Suffix (eg, Jr, III)	Academic Degrees	Institution	Location (city, state/province, country)	Role or Contribution, eg, chair, principal investigator	Group (if more than 1 Group listed in the byline) and/or Subgroup (eg, Steering Committee)
Selina	Bannoo			ROKC; Research Organisation (kc) ltd. (CRO for EU5332)	London, United Kingdom	REPRIEVE Committee	EU Committee
Annie	Duffy			ROKC; Research Organisation (kc) ltd. (CRO for EU5332)	London, United Kingdom	REPRIEVE Committee	EU Committee
Carl	Fletcher			ROKC; Research Organisation (kc) ltd. (CRO for EU5332)	London, United Kingdom	REPRIEVE Committee	EU Committee
Madison	Green			Frontier Science Foundation; Data Management Center	Amherst, NY, USA	REPRIEVE Committee	EU Committee; Lab Committee
Nory	Klop-Packel			Massachusetts General Hospital; Data Coordinating Center	Boston, MA, USA	REPRIEVE Committee	EU Committee
Sara	McCallum		MPH	Massachusetts General Hospital; Clinical Coordinating Center	Boston, MA, USA	REPRIEVE Committee	EU Committee; Lab Committee; Publications Committee; Mechanistic Substudy CT Committee
Emilia	Norton			Massachusetts General Hospital; Data Coordinating Center	Boston, MA, USA	REPRIEVE Committee	EU Committee
Jennifer	Nowak			Frontier Science Foundation; Data Management Center	Amherst, NY, USA	REPRIEVE Committee	EU Committee
Maria	Sanchez Grande			ROKC; Research Organisation (kc) ltd. (CRO for EU5332)	London, United Kingdom	REPRIEVE Committee	EU Committee
Sue	Siminski			Frontier Science Foundation; Data Management Center	Amherst, NY, USA	REPRIEVE Committee	EU Committee; Operational Leadership Committee
Eloise	Walker			ROKC; Research Organisation (kc) ltd. (CRO for EU5332)	London, United Kingdom	REPRIEVE Committee	EU Committee
David	Vlieg			Frontier Science Foundation; Data Management Center	Amherst, NY, USA	REPRIEVE Committee	EU Committee; Lab Committee
Tricia	Burdo		PhD	Temple University	Philadelphia, PA, USA	REPRIEVE Committee	Lab Committee; Operational Leadership Committee

Supplemental Online Content: Nonauthor Collaborators

*Indicates required information. Only first name, last name, and suffix will appear in PubMed.

***First Name and Middle Initial(s)**	***Last Name**	***Suffix (eg, Jr, III)**	Academic Degrees	Institution	Location (city, state/province, country)	Role or Contribution, eg, chair, principal investigator	Group (if more than 1 Group listed in the byline) and/or Subgroup (eg, Steering Committee)
Laura	Moran		MPH	AIDS Clinical Trials Group; AIDS Clinical Trials Network (ACTG Network Coordinating Center, DLH Corporation)	Boston, MA, USA	REPRIEVE Committee	Lab Committee; Operational Leadership Committee; Protocol Writing, Training, and Implementation Committee; Publications Committee
Jhoanna	Roa			DLH Corporation; AIDS Clinical Trials Network (ACTG Network Coordinating Center, DLH Corporation)	Silver Spring, MD, USA	REPRIEVE Committee	Lab Committee
Heather	Sprenger			Frontier Science Foundation; Data Management Center	Amherst, NY, USA	REPRIEVE Committee	Lab Committee; Operational Leadership Committee; Site Selection, Performance, and Close Out Committee
Bola	Adedeji			Division of AIDS of the National Institute of Health; National Institutes of Health/National Institute of Allergy and Infectious Diseases	Bethesda, MD, USA	REPRIEVE Committee	Operational Leadership Committee; Site Selection, Performance, and Close Out Committee
Oladapo	Alli		PharmD	Division of AIDS of the National Institute of Health; National Institutes of Health/National Institute of Allergy and Infectious Diseases	Bethesda, MD, USA	REPRIEVE Committee	Operational Leadership Committee; Protocol Writing, Training, and Implementation Committee; Site Selection, Performance, and Close Out Committee

*Indicates required information. Only first name, last name, and suffix will appear in PubMed.

***First Name and Middle Initial(s)**	***Last Name**	***Suffix (eg, Jr, III)**	Academic Degrees	Institution	Location (city, state/province, country)	Role or Contribution, eg, chair, principal investigator	Group (if more than 1 Group listed in the byline) and/or Subgroup (eg, Steering Committee)
Blanca	Castillo			Division of AIDS of the National Institute of Health; National Institutes of Health/National Institute of Allergy and Infectious Diseases	Bethesda, MD, USA	REPRIEVE Committee	Operational Leadership Committee; Site Selection, Performance, and Close Out Committee
Joan	Dragavon			Division of AIDS of the National Institute of Health; National Institutes of Health/National Institute of Allergy and Infectious Diseases; AIDS Clinical Trials Network (ACTG)	Seattle, WA, USA	REPRIEVE Committee	Operational Leadership Committee; Protocol Writing, Training, and Implementation Committee
Keisha	Easley		PharmD	Division of AIDS of the National Institute of Health; National Institutes of Health/National Institute of Allergy and Infectious Diseases	Bethesda, MD, USA	REPRIEVE Committee	Operational Leadership Committee; Protocol Writing, Training, and Implementation Committee; Site Selection, Performance, and Close Out Committee
Julian	Falutz		MD	Canadian HIV Trials Network	Vancouver, British Columbia, Canada	REPRIEVE Committee	Operational Leadership Committee
Ewelinka	Grzejka			Massachusetts General Hospital; Clinical Coordinating Center	Boston, MA, USA	REPRIEVE Committee	Operational Leadership Committee
Erin	Hoffman		BS	University of North Carolina Chapel Hill; AIDS Clinical Trials Network (ACTG)	Chapel Hill, NC, USA	REPRIEVE Committee	Operational Leadership Committee; Protocol Writing, Training, and Implementation Committee
Yuji	Liao			Massachusetts General Hospital; Data Coordinating Center	Boston, MA, USA	REPRIEVE Committee	Operational Leadership Committee
Sara	Looby		NP, PhD	Massachusetts General Hospital; Clinical Coordinating Center	Boston, MA, USA	REPRIEVE Committee	Operational Leadership Committee; Publications Committee

*Indicates required information. Only first name, last name, and suffix will appear in PubMed.

***First Name and Middle Initial(s)**	***Last Name**	***Suffix (eg, Jr, III)**	Academic Degrees	Institution	Location (city, state/province, country)	Role or Contribution, eg, chair, principal investigator	Group (if more than 1 Group listed in the byline) and/or Subgroup (eg, Steering Committee)
Dana	Nohynek			Canadian HIV Trials Network	Vancouver, British Columbia, Canada	REPRIEVE Committee	Operational Leadership Committee
Mary	Pate			Kowa Pharmaceuticals America, Inc.	Montgomery, AL, USA	REPRIEVE Committee	Operational Leadership Committee
James	Rooney		MD	Gilead Sciences, Inc.	Foster City, CA	REPRIEVE Committee	Operational Leadership Committee; Publications Committee
Akbar	Shahkolahi		PhD	AIDS Clinical Trials Group; AIDS Clinical Trials Network (ACTG)	Boston, MA, USA	REPRIEVE Committee	Operational Leadership Committee; Protocol Writing, Training, and Implementation Committee; Site Selection, Performance, and Close Out Committee
Craig	Sponseller		MD	Kowa Pharmaceuticals America, Inc.	Montgomery, AL, USA	REPRIEVE Committee	Operational Leadership Committee; Publications Committee
Kenneth	Williams			Frontier Science Foundation	Amherst, NY, USA	REPRIEVE Committee	Operational Leadership Committee
Markella	Zanni		MD	Massachusetts General Hospital; Clinical Coordinating Center	Boston, MA, USA	REPRIEVE Committee	Operational Leadership Committee; Protocol Writing, Training, and Implementation Committee; Publications Committee; Site Selection, Performance, and Close Out Committee
Kate	Borloglou			AIDS Clinical Trials Group	Boston, MA, USA	REPRIEVE Committee	Protocol Writing, Training, and Implementation Committee

Supplemental Online Content: Nonauthor Collaborators

*Indicates required information. Only first name, last name, and suffix will appear in PubMed.

*First Name and Middle Initial(s)	*Last Name	*Suffix (eg, Jr, III)	Academic Degrees	Institution	Location (city, state/province, country)	Role or Contribution, eg, chair, principal investigator	Group (if more than 1 Group listed in the byline) and/or Subgroup (eg, Steering Committee)
Meredith	Clement			Duke University	Durham, NC, USA	REPRIEVE Committee	Protocol Writing, Training, and Implementation Committee
Allison	Eckard		MD	Medical University of South Carolina	Charleston, SC, USA	REPRIEVE Committee	Protocol Writing, Training, and Implementation Committee
Rebecca	LeBlanc			Frontier Science Foundation	Amherst, NY, USA	REPRIEVE Committee	Protocol Writing, Training, and Implementation Committee
Carlos	Malvestutto		MD, MPH	Ohio State University Medical Center	Columbus, OH, USA	REPRIEVE Committee Chair (Protocol Committee); REPRIEVE Committee	Protocol Writing, Training, and Implementation Committee; Publications Committee; Site Selection, Performance, and Close Out Committee
Edgar T	Overton			University of Alabama at Birmingham	Birmingham, AL, USA	REPRIEVE Committee Former Chair	Protocol Writing, Training, and Implementation Committee
Karl	Shaw			ACTG Community Scientific Subcommittee; Community Scientific Subcommittee Representatives	Boston, MA, USA	REPRIEVE Committee	Protocol Writing, Training, and Implementation Committee
Virginia	Triant		MD	Massachusetts General Hospital	Boston, MA, USA	REPRIEVE Committee	Protocol Writing, Training, and Implementation Committee

Supplemental Online Content: Nonauthor Collaborators

*Indicates required information. Only first name, last name, and suffix will appear in PubMed.

***First Name and Middle Initial(s)**	***Last Name**	***Suffix (eg, Jr, III)**	Academic Degrees	Institution	Location (city, state/province, country)	Role or Contribution, eg, chair, principal investigator	Group (if more than 1 Group listed in the byline) and/or Subgroup (eg, Steering Committee)
Amy	Kantor		MS	Harvard T.H. Chan School of Public Health; Center for Biostatistics and AIDS Research at Harvard T.H. Chan School of Public Health	Boston, MA, USA	REPRIEVE Committee	Publications Committee
Jennifer M.	Manne-Goehler		MD	Brigham and Women's Hospital	Boston, MA, USA	REPRIEVE Committee	Publications Committee
Kate	Starr			ACTG Community Scientific Subcommittee; Community Scientific Subcommittee Representatives	Boston, MA, USA	REPRIEVE Committee	Publications Committee
Ronald	Barnett			Division of AIDS of the National Institute of Health	Bethesda, MD, USA	REPRIEVE Committee	Site Selection, Performance, and Close Out Committee
Jane	Baum		RN	Case Western Reserve	Cleveland, OH, USA	REPRIEVE Committee	Site Selection, Performance, and Close Out Committee
Cindy	Coates			Division of AIDS of the National Institute of Health	Boston, MA, USA	REPRIEVE Committee	Site Selection, Performance, and Close Out Committee
Sandra W.	Cordoso			Instituto de Pesquisa Clinica Evandro Chagas	Rio de Janeiro, Brazil	REPRIEVE Committee	Site Selection, Performance, and Close Out Committee
Christie Lyn	Costanza			New Jersey Medical School	Newark, NJ, USA	REPRIEVE Committee	Site Selection, Performance, and Close Out Committee
Sylvia	Davila			Puerto Rico AIDS Clinical Trials Unit	San Juan, PR	REPRIEVE Committee	Site Selection, Performance, and Close Out Committee
Dushyantha	Jayaweera			Jackson Memorial Hospital	Miami, FL, USA	REPRIEVE Committee	Site Selection, Performance, and Close Out Committee

*Indicates required information. Only first name, last name, and suffix will appear in PubMed.

***First Name and Middle Initial(s)**	***Last Name**	***Suffix (eg, Jr, III)**	Academic Degrees	Institution	Location (city, state/province, country)	Role or Contribution, eg, chair, principal investigator	Group (if more than 1 Group listed in the byline) and/or Subgroup (eg, Steering Committee)
Teri	Greenfield			National Institute of Allergy and Infectious Diseaes of the National Institutes of Health	Bethesda, MD, USA	REPRIEVE Committee	Site Selection, Performance, and Close Out Committee
Howard	Gutzman			Frontier Science Foundation; Data Management Center	Amherst, NY, USA	REPRIEVE Committee	Site Selection, Performance, and Close Out Committee
Regina	Harden			University of Illinois at Chicago Project Wish	Chicago, IL, USA	REPRIEVE Committee	Site Selection, Performance, and Close Out Committee
Sarah	Henn			Whitman-Walker Institute, Inc.	Washington, DC, USA	REPRIEVE Committee	Site Selection, Performance, and Close Out Committee
MJ	Humphries			Division of AIDS of the National Institute of Health	Bethesda, MD, USA	REPRIEVE Committee	Site Selection, Performance, and Close Out Committee
Mamta	Jain			University of Texas Southwestern	Dallas, TX, USA	REPRIEVE Committee	Site Selection, Performance, and Close Out Committee
David	Klein			Whitman-Walker Institute, Inc.	Washington, DC, USA	REPRIEVE Committee	Site Selection, Performance, and Close Out Committee
Sharon	Kohrs			University of Cincinnati College of Medicine	Cincinnati, OH, USA	REPRIEVE Committee	Site Selection, Performance, and Close Out Committee
Javier	Lama			Barranco Clinical Research Site	Lima, Peru	REPRIEVE Committee	Site Selection, Performance, and Close Out Committee
Jessica	Landis			Division of AIDS of the National Institute of Health	Bethesda, MD, USA	REPRIEVE Committee	Site Selection, Performance, and Close Out Committee
Jaclyn	Leone			University of North Carolina Chapel Hill	Chapel Hill, NC, USA	REPRIEVE Committee	Site Selection, Performance, and Close Out Committee

*Indicates required information. Only first name, last name, and suffix will appear in PubMed.

*First Name and Middle Initial(s)	*Last Name	*Suffix (eg, Jr, III)	Academic Degrees	Institution	Location (city, state/province, country)	Role or Contribution, eg, chair, principal investigator	Group (if more than 1 Group listed in the byline) and/or Subgroup (eg, Steering Committee)
Rita	Lira			Hospital Nossa Senhora da Conceicao	Porto Alegre, Brazil	REPRIEVE Committee	Site Selection, Performance, and Close Out Committee
Maria	Martinez		RN	University of Texas Houston	Houston, TX, USA	REPRIEVE Committee	Site Selection, Performance, and Close Out Committee
Richard	Novak			University of Illinois at Chicago Project Wish	Chicago, IL, USA	REPRIEVE Committee	Site Selection, Performance, and Close Out Committee
Karen	Reese			Division of AIDS of the National Institute of Health; National Institutes of Health/National Institute of Allergy and Infectious Diseases	Bethesda, MD, USA	REPRIEVE Committee	Site Selection, Performance, and Close Out Committee
Breno	Santos			Hospital Nossa Senhora da Conceicao	Porto Alegre, Brazil	REPRIEVE Committee	Site Selection, Performance, and Close Out Committee
Jenese	Tucker			Division of AIDS of the National Institute of Health	Bethesda, MD, USA	REPRIEVE Committee	Site Selection, Performance, and Close Out Committee
Aimee	Wilkin		MD	Wake Forest University	Winston-Salem, NC, USA	REPRIEVE Committee	Site Selection, Performance, and Close Out Committee
Tomeka	Wilson			University of Alabama at Birmingham	Birmingham, AL, USA	REPRIEVE Committee	Site Selection, Performance, and Close Out Committee
Borek	Foldyna		MD	Massachusetts General Hospital; Data Coordinating Center	Boston, MA, USA	REPRIEVE Committee	Mechanistic Substudy CT Committee
Julia	Karady		MD	Massachusetts General Hospital; Data Coordinating Center	Boston, MA, USA	REPRIEVE Committee	Mechanistic Substudy CT Committee
Thomas	Mayrhofer			Massachusetts General Hospital; Data Coordinating Center	Boston, MA, USA	REPRIEVE Committee	Mechanistic Substudy CT Committee

Supplemental Online Content: Nonauthor Collaborators

*Indicates required information. Only first name, last name, and suffix will appear in PubMed.

*First Name and Middle Initial(s)	*Last Name	*Suffix (eg, Jr, III)	Academic Degrees	Institution	Location (city, state/province, country)	Role or Contribution, eg, chair, principal investigator	Group (if more than 1 Group listed in the byline) and/or Subgroup (eg, Steering Committee)
Audra	Sturniolo		MS	Massachusetts General Hospital; Data Coordinating Center	Boston, MA, USA	REPRIEVE Committee	Mechanistic Substudy CT Committee
Barbara	Bastow			AIDS Clinical Trials Network (ACTG)	Boston, MA, USA		
Francoise	Giguel			AIDS Clinical Trials Network (ACTG)	Boston, MA, USA		
Nada	Saleh			AIDS Clinical Trials Network (ACTG Network Coordinating Center, DLH Corporation)	Boston, MA, USA		
John	Ward			Biomedical Research Institute (Biorepository)	Rockville, MD, USA		
Erin	Cherban			Canadian Trials Network	Vancouver, British Columbia, Canada		
Sean	Brummel			Center for Biostatistics and AIDS Research at Harvard T.H. Chan School of Public Health	Boston, MA, USA		
Janeway	Granche			Center for Biostatistics and AIDS Research at Harvard T.H. Chan School of Public Health	Boston, MA, USA		
Carlee	Moser			Center for Biostatistics and AIDS Research at Harvard T.H. Chan School of Public Health	Boston, MA, USA		
Pawel	Paczuski			Center for Biostatistics and AIDS Research at Harvard T.H. Chan School of Public Health	Boston, MA, USA		
Laura	Smeaton			Center for Biostatistics and AIDS Research at Harvard T.H. Chan School of Public Health	Boston, MA, USA		
Claire	Benjamin			Clinical Coordinating Center	Boston, MA, USA		
Tanisha	Cadet			Clinical Coordinating Center	Boston, MA, USA		
Evelynne	Fulda			Clinical Coordinating Center	Boston, MA, USA		
Jacqueline	Murphy			Clinical Coordinating Center	Boston, MA, USA		

Supplemental Online Content: Nonauthor Collaborators

*Indicates required information. Only first name, last name, and suffix will appear in PubMed.

***First Name and Middle Initial(s)**	***Last Name**	***Suffix (eg, Jr, III)**	Academic Degrees	Institution	Location (city, state/province, country)	Role or Contribution, eg, chair, principal investigator	Group (if more than 1 Group listed in the byline) and/or Subgroup (eg, Steering Committee)
Alicia	Diggs			Community Advisory Board			
Robert	Ettinger			Community Advisory Board			
Angel	Hernandez			Community Advisory Board			
Janice	Jarrells			Community Advisory Board			
Shirley	Selvage			Community Advisory Board			
Sandeep	Hedgire			Data Coordinating Center	Boston, MA, USA		
Udo	Hoffman		MD	Data Coordinating Center	Boston, MA, USA		
Nina M.	Meyersohn		MD	Data Coordinating Center	Boston, MA, USA		
Jana	Taron		MD	Data Coordinating Center	Boston, MA, USA		
Anthony	Holguin			Data Management Center (Frontier Science Foundation)	Amherst, NY, USA		
Gregory	Pavlov			Data Management Center (Frontier Science Foundation)	Amherst, NY, USA		
Scott	Hammer		MD	External Advisory Board	New York, NY, USA		
Martin	Hirsch			External Advisory Board			
JoAnn	Manson		MD, MPH, DrPH	External Advisory Board	Boston, MA, USA		
Paul	Ridker		MD	External Advisory Board	Boston, MA, USA		
James	Stein		MD	External Advisory Board	Madison, WI, USA		
Russel	Tracy		PhD	External Advisory Board	Burlington, VT, USA		
James	Udelson		MD	External Advisory Board	Boston, MA, USA		
Esteban	Martinez			The European Treatment Network for HIV, Hepatitis, and Global Infectious Diseases (NEATid)			
Tim	Leaver			The European Treatment Network for HIV, Hepatitis, and Global Infectious Diseases (NEATid)			
Anton	Pozniak			The European Treatment Network for HIV, Hepatitis, and Global Infectious Diseases (NEATid)			
Kathy	Melbourne			Gilead Sciences, Inc.			
Matthew	Budoff		MD	Independent Data and Safety Monitoring Board			

Supplemental Online Content: Nonauthor Collaborators

*Indicates required information. Only first name, last name, and suffix will appear in PubMed.

***First Name and Middle Initial(s)**	***Last Name**	***Suffix (eg, Jr, III)**	Academic Degrees	Institution	Location (city, state/province, country)	Role or Contribution, eg, chair, principal investigator	Group (if more than 1 Group listed in the byline) and/or Subgroup (eg, Steering Committee)
Ben	Cheng			Independent Data and Safety Monitoring Board			
Sara	Goldkind		MD, MA	Independent Data and Safety Monitoring Board			
Carl	Grunfeld		MD	Independent Data and Safety Monitoring Board			
Robert	Harrington		MD	Independent Data and Safety Monitoring Board		Chair	
Donald	Lloyd-Jones		MD	Independent Data and Safety Monitoring Board			
Jennifer	Robinson		MD, MPH	Independent Data and Safety Monitoring Board			
Lynn	Sleeper		ScD	Independent Data and Safety Monitoring Board			
George	Sopko		MD, MPH	Independent Data and Safety Monitoring Board; National Institutes of Health/National Heart Lung and Blood Institute	Bethesda, MD, USA	Executive Secretary	
Paul	Volberding		MD	Independent Data and Safety Monitoring Board			
Fassil	Ketema			National Institutes of Health/National Heart Lung and Blood Institute	Bethesda, MD, USA		
Karin	Klingman		MD	National Institutes of Health/National Institute of Allergy and Infectious Diseases	Rockville, MD, USA		
Keisha	Johnson			National Institutes of Health/National Institute of Allergy and Infectious Diseases	Rockville, MD, USA		
Mark	Mishkin			National Institutes of Health/National Institute of Allergy and Infectious Diseases	Rockville, MD, USA		

Supplemental Online Content: Nonauthor Collaborators

*Indicates required information. Only first name, last name, and suffix will appear in PubMed.

***First Name and Middle Initial(s)**	***Last Name**	***Suffix (eg, Jr, III)**	Academic Degrees	Institution	Location (city, state/province, country)	Role or Contribution, eg, chair, principal investigator	Group (if more than 1 Group listed in the byline) and/or Subgroup (eg, Steering Committee)
Daniella	Livnat			National Institutes of Health/National Institute of Allergy and Infectious Diseases	Rockville, MD, USA		
Akin	Ojumu			National Institutes of Health/National Institute of Allergy and Infectious Diseases	Rockville, MD, USA		
Alba	Sierto			Research Organisation (kc) ltd. (CRO for EU5332)			
