# Supplementary material for: Effects of Pitavastatin on Coronary Artery Disease and Inflammatory Biomarkers in HIV: Mechanistic Substudy of the REPRIEVE Randomized Clinical Trial
Source: JAMA Cardiol. 2024 Feb 21;9(4):323–34. doi: 10.1001/jamacardio.2023.5661 (PMC10882511; doi:10.1001/jamacardio.2023.5661)
Supplement: Supplement 4. — Data Sharing Statement [file jamacardiol-e235661-s004.pdf]

Data Sharing Statement

Lu. Effects of Pitavastatin on Coronary Artery Disease and Inflammatory Biomarkers in HIV.
JAMA Cardiol. Published February 21, 2024. doi:10.1001/jamacardio.2023.5661

Data
Data available: Yes
Data types: Deidentified participant data
How to access data: Data may be made available to the research community affiliated with an approved academic institution, by reasonable request and after review by the trial principal investigators.
When available: beginning date: 12-31-2025

Supporting Documents
Document types: Other (please specify)
Additional Information: trial protocol, including the statistical analysis plan, and any amendments as online supplements
How to access documents: in the online supplement
When available: With publication

Additional Information
Who can access the data: Data may be made available to the research community affiliated with an approved academic institution, by reasonable request and after review by the trial principal investigators.
Types of analyses: The type of analysis or purpose must be approved by trial principal investigators
Mechanisms of data availability: After approval of a proposal, signed data access agreement, and with investigator support as appropriate.
